# Global Expression Profiling in Atopic Eczema Reveals Reciprocal Expression of Inflammatory and Lipid Genes

**DOI:** 10.1371/journal.pone.0004017

**Published:** 2008-12-24

**Authors:** Annika M. Sääf, Maria Tengvall-Linder, Howard Y. Chang, Adam S. Adler, Carl-Fredrik Wahlgren, Annika Scheynius, Magnus Nordenskjöld, Maria Bradley

**Affiliations:** 1 Department of Molecular Medicine and Surgery, Karolinska Institutet, Stockholm, Sweden; 2 Department of Medicine Solna, Clinical Allergy Research Unit, Karolinska Institutet, Stockholm, Sweden; 3 Programs in Epithelial Biology and Cancer Biology, Stanford University School of Medicine, Stanford, California, United States of America; 4 Department of Medicine Solna, Dermatology Unit, Karolinska Institutet, Stockholm, Sweden; Centre for Genomic Regulation, Spain

## Abstract

**Background:**

Atopic eczema (AE) is a common chronic inflammatory skin disorder. In order to dissect the genetic background several linkage and genetic association studies have been performed. Yet very little is known about specific genes involved in this complex skin disease, and the underlying molecular mechanisms are not fully understood.

**Methodology/Findings:**

We used human DNA microarrays to identify a molecular picture of the programmed responses of the human genome to AE. The transcriptional program was analyzed in skin biopsy samples from lesional and patch-tested skin from AE patients sensitized to *Malassezia sympodialis* (*M. sympodialis*), and corresponding biopsies from healthy individuals. The most notable feature of the global gene-expression pattern observed in AE skin was a reciprocal expression of induced inflammatory genes and repressed lipid metabolism genes. The overall transcriptional response in *M. sympodialis* patch-tested AE skin was similar to the gene-expression signature identified in lesional AE skin. In the constellation of genes differentially expressed in AE skin compared to healthy control skin, we have identified several potential susceptibility genes that may play a critical role in the pathological condition of AE. Many of these genes, including genes with a role in immune responses, lipid homeostasis, and epidermal differentiation, are localized on chromosomal regions previously linked to AE.

**Conclusions/Significance:**

Through genome-wide expression profiling, we were able to discover a distinct reciprocal expression pattern of induced inflammatory genes and repressed lipid metabolism genes in skin from AE patients. We found a significant enrichment of differentially expressed genes in AE with cytobands associated to the disease, and furthermore new chromosomal regions were found that could potentially guide future region-specific linkage mapping in AE. The full data set is available at http://microarray-pubs.stanford.edu/eczema.

## Introduction

Atopic eczema (AE) (OMIM#603165) is a common skin disorder currently affecting 10–20% of children and 1–3% of adults in westernized countries [1], [2]. Patients with AE suffer from itchy, dry and inflamed skin, often in combination with other atopic manifestations such as allergic asthma and allergic rhinoconjunctivitis. Twin studies indicate a strong genetic contribution in the development of AE [3], [4], and to date four genome-wide linkage studies have been performed in Caucasian populations identifying several chromosomal regions linked to AE susceptibility [5]–[8]. However, a complete genomic picture of this complex disorder still remains to be defined, and importantly specific genes involved in the pathogenesis of AE have to be identified.

Over the past decades, much research has been focused on advancing the knowledge about the role and action of immune competent cells and inflammatory molecules in AE pathogenesis. Activated T-helper cells, eosinophils, macrophages and mast cells are often found in AE skin [9], [10]. In addition, there is an imbalance between T_H_1 and T_H_2 cells giving an increase in production of a T_H_2 cytokine profile, at least in the early phases of the disease [11]. A network of cytokines/chemokines and their receptors that is characteristically expressed in patients with AE has been identified [12], [13]. However, it is still not clear whether the inflammatory response found in AE skin is a primary basic cause of the disease, or if it is a secondary effect caused by other factors such as an impaired skin barrier.

The importance of an impaired epidermal differentiation process and skin barrier dysfunction in the pathogenesis of AE has recently been emphasized when a set of so-called “epidermal differentiation genes”, including S100A7, S100A8, loricrin and filaggrin (FLG), was found to be differentially expressed in AE patients compared to healthy control individuals [14]. In addition, genetic association studies have identified *FLG* to be a susceptibility gene for AE, further supporting the importance of epidermal differentiation genes in AE (reviewed in [15]). An impaired skin barrier function in AE patients may also be an effect of changes in the lipid composition [16], [17]. However, the precise role of various molecules and a detailed underlying mechanism to skin barrier dysfunction in AE patients is still far from understood.

AE results from a complex interplay between genetic and environmental factors. For example, it is known that allergens such as house dust mite and bacteria such as staphylococci are aggravating factors in AE [9], [18]. Members of the lipophilic *Malassezia* yeast family are part of the normal microflora on human skin. However, *Malassezia* has been associated with AE and other skin diseases such as seborrhoeic dermatitis and pityriasis versicolor [19]. *Malassezia*-specific IgE antibodies are often found in adult AE patients, but not in other allergic diseases or among healthy individuals [20]. Application of *Malassezia sympodialis* (*M. sympodialis*) extract on non-lesional skin of AE patients, referred to as an atopy patch test (APT), triggers immunological changes in the skin similar to acute lesional eczema [19], [21], [22]. It is unknown why AE patients are hypersensitive to *M. sympodialis*, nor is it known if *M. sympodialis* has a major effect on transcript levels in eczema skin.

In this study, we used cDNA microarrays representing approximately 24,500 unique genes to identify a detailed molecular picture of the programmed responses of the human genome to AE. The transcriptional program was analyzed in skin biopsy samples from lesional and patch-tested skin from AE patients sensitized to *M. sympodialis*, and corresponding biopsies from healthy individuals. A set of genes identified by the microarrays was selected for further analysis by immunohistochemistry and immunofluorence staining to confirm and explore corresponding protein levels and cell type expression in skin. We found that non-lesional skin from AE patients that was patch-tested with a PBS negative control has a predisposed genetic program different from normal healthy skin. Furthermore, we show that the global transcriptional response to *M. sympodialis* patch-test in non-lesional AE skin is very similar to the gene-signature identified in lesional AE skin. The most notable feature of the global gene-expression pattern observed in AE skin was a reciprocal expression of induced inflammatory genes and repressed lipid metabolism genes. Genes encoding key enzymes and structural proteins involved in assembly of the cornified layer also demonstrated altered expression in AE skin.

## Results

### Global portrait of altered expression profiles in AE

**Figure 1:**
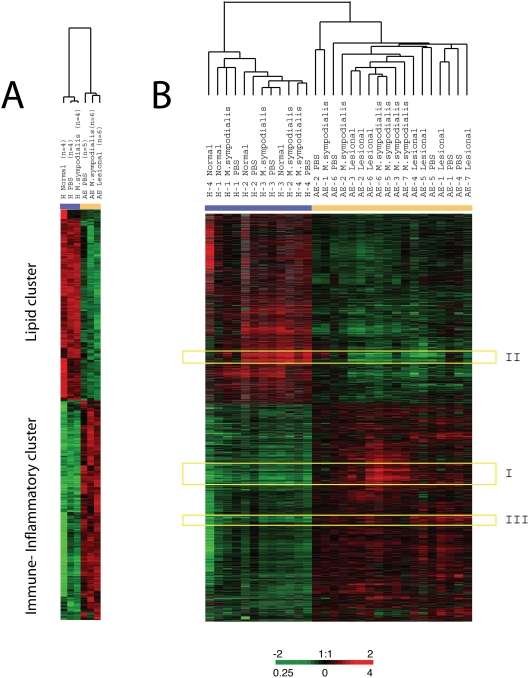
Global comparison between the gene-expression patterns underlying AE pathogenesis to those identified in normal healthy skin. (A) Thumbnail overview of ∼4000 array elements selected for differential expression in skin from AE patients and healthy individuals by a multi-group significance analysis of microarrays (SAM) approach. Hierarchical clustering analysis was performed in both the gene (row) and experiment (column) dimension. Note that each column represents a group of skin samples. The “contrast value” for each gene is shown, e.g. the standardized mean difference between the gene's expression in the class, versus its overall expression. (B) The SAM gene list of approximately 4000 array elements was used to extract patterns of gene expression from each AE patient and healthy control individual (indicated by separate numbers). Note that each column here is represented by one array experiment. Transcript levels determined by microarray analysis are shown relative to a reference pool of human mRNAs. Expression levels of each gene relative to its mean expression level over the sample set are displayed in a log2 scale. Expression levels are represented by a color bar, where red is representing the highest levels and green is representing the lowest levels of expression. Highlighted clusters (I–III) are described in detail below in Figures 2, 5 and 6. The full data set can be found at the SMD database http://microarray-pubs.stanford.edu/eczema and at the NCBI GEO database (GEO accession: GSE12511).

cDNA microarrays representing approximately 24,500 unique genes were used to identify the global gene-signature in lesional and patch-tested skin from AE patients, and control skin samples from healthy individuals. A multi-group Significance Analysis of Microarrays (SAM) approach [23] was undertaken to select a set of ∼4000 genes that were consistently differentially expressed between AE and healthy skin with a false discovery rate (FDR) <0.003. The full list of genes identified by the SAM approach to be consistently up- or down-regulated in AE skin as compared to healthy control skin is provided as Supplementary Table S1. Hierarchical clustering was used to group these genes based on similarity in expression across the samples and to group individuals on the basis of similarities in gene-expression patterns (Figure 1A). Each column in Figure 1A represents a group of skin biopsy samples and not a single array experiment. The same set of genes was next used to extract the patterns of gene-expression from each individual biopsy sample. Hierarchical clustering of these genes, in both gene and sample dimension, are displayed in Figure 1B. The clustering analysis clearly separates AE and normal healthy skin samples into two distinct branches. We found that the global transcriptional response to *M. sympodialis* extract in AE skin is very similar to that found in lesional AE skin. The healthy control group did not respond to the *M. sympodialis* or the PBS patch-test, neither on a transcriptional level nor as a visible reaction on the skin. The patients with AE also had no visible skin reaction to the PBS control patch-test. However, the gene-expression program observed in non-lesional PBS tested AE skin was more similar to the gene-signature identified in lesional AE skin than to that found in the skin of healthy individuals.

The most notable feature of the observed gene-expression program was a reciprocal expression pattern found for immune/inflammatory genes (up-regulated in AE skin), and genes involved in lipid metabolism (down-regulated in AE skin) (Figures 1A and B). Using the “gene module map method” [24] to identify significantly enriched Gene Ontology (GO) terms [25], we found that “immune response” was the most significantly enriched GO term in up-regulated AE genes (*P*<10^−73^) and “lipid metabolism” was the most significantly enriched GO term in down-regulated AE genes (*P*<10^−33^) (full list of enriched GO terms is provided in Supplementary Table S2).

The induced expression of immune and inflammatory genes, such as interleukins, cell surface antigens, and genes induced by interferon, were especially prominent in *M. sympodialis* AE skin. We also identified genes encoding structural components and enzymes with a key role in terminal keratinocyte differentiation and cornified envelope assembly that were significantly different in AE skin as compared to healthy control skin. Notably is that many of these potential AE candidate genes are localized to chromosomal regions that previously have been linked to AE (Table 1). Representative genes from different clusters in Figure 1 (Cluster I, II and III) are further described below.

**Table 1:** Enrichment of chromosomal regions.

Enriched cytoband		*P*-value	# of cytoband genes induced/repressed	Total # of genes in cytoband
***2194 genes induced in AE:***				
11q12.1	**	5.96E-15	38	210
19p13.3	**	4.10E-13	34	194
19q12		4.33E-13	32	173
6p21.2		6.87E-10	18	75
22q13.1		2.01E-09	15	54
3p21.31	**	9.71E-08	20	123
1q23.1		6.03E-07	14	70
16p11.2	**	7.23E-07	14	71
19q13.2	**	9.70E-07	18	117
16p13.3		1.09E-06	19	130
19p13.2	**	1.25E-06	18	119
11p15.5		1.49E-06	11	46
**3p25.3**	*	**3.13E-06**	**9**	**32**
9p21.1		6.50E-06	12	63
12q13.12	**	1.37E-05	10	47
6p21.1		1.84E-05	20	171
9q34.2		3.44E-05	6	17
19p13.12	**	3.56E-05	12	74
10q26.2		3.85E-05	5	11
22q12.3		4.24E-05	9	43
1q22		4.24E-05	9	43
**1q21.3**	*	**5.34E-05**	**12**	**77**
19q13.13	**	5.46E-05	7	26
11q11		7.04E-05	6	19
17p11.2		1.58E-04	8	40
3q13.33		1.75E-04	6	22
*5q32*	***	1.75E-04	6	22
Xp22.22		2.28E-04	6	23
17q11.2	**	2.80E-04	10	66
12p13.31		4.05E-04	10	69
20q13.12		4.09E-04	9	57
1p34.3		4.67E-04	9	58
17q24.3		4.88E-04	7	36
1p36.11		5.05E-04	8	47
Xq13.1		6.73E-04	4	11
*14q11.2*	***	7.00E-04	11	87
12q13.13	**	7.94E-04	10	75
5q23.1		1.51E-03	6	32
12q21.33		1.52E-03	5	22
2p14		1.52E-03	5	22
15q26.1		1.70E-03	7	44
3q24		1.78E-03	6	33
2q33.3	**	1.85E-03	4	14
8p21.2		1.88E-03	5	23
4p16.3		1.94E-03	7	45
11p15.4		2.49E-03	9	73
16q12.2	**	2.51E-03	7	47
1q25.2		2.77E-03	5	25
***1908 genes repressed in AE:***				
2q24.3		4.29E-06	6	24
12q13.11	**	8.61E-06	7	40
6q22.1		1.43E-05	4	9
6q14.1		7.22E-05	5	24
7q34		1.42E-04	4	15
14q24.3		2.10E-04	6	46
21q22.3		2.67E-04	6	48
4q28.1		7.14E-04	3	10
4q21.1		8.17E-04	4	23
1q32.2		8.17E-04	4	23
1p36.13		8.73E-04	5	40
12p12.3		9.68E-04	3	11
1p36.22		1.09E-03	5	42
7q22.3		1.27E-03	3	12
**15q21.3**	*	**1.53E-03**	**4**	**27**
10q25.1		1.53E-03	4	27
3q11.2		1.63E-03	3	13
2q14.1		1.75E-03	4	28
1p22.2		2.00E-03	4	29
3q22.1	**	2.52E-03	3	15
5q23.2		2.52E-03	3	15
12p12.1		2.52E-03	3	15

Shown are chromosomal regions (cytobands) that are enriched in 2194 genes induced or 1908 genes repressed in AE (P<0.05; FDR<0.05). Cytobands are sorted by P-value. Overlap with previously known AE genome-wide scan regions [5]–[8], [37], AE candidate gene regions [2], [36], [39]–[40], and AE regions identified by selective region specific linkage mapping [35], [38] are indicated in the second column by *, **, and ***, respectively.

### Induced expression of inflammatory and immune related genes in AE skin

**Figure 2:**
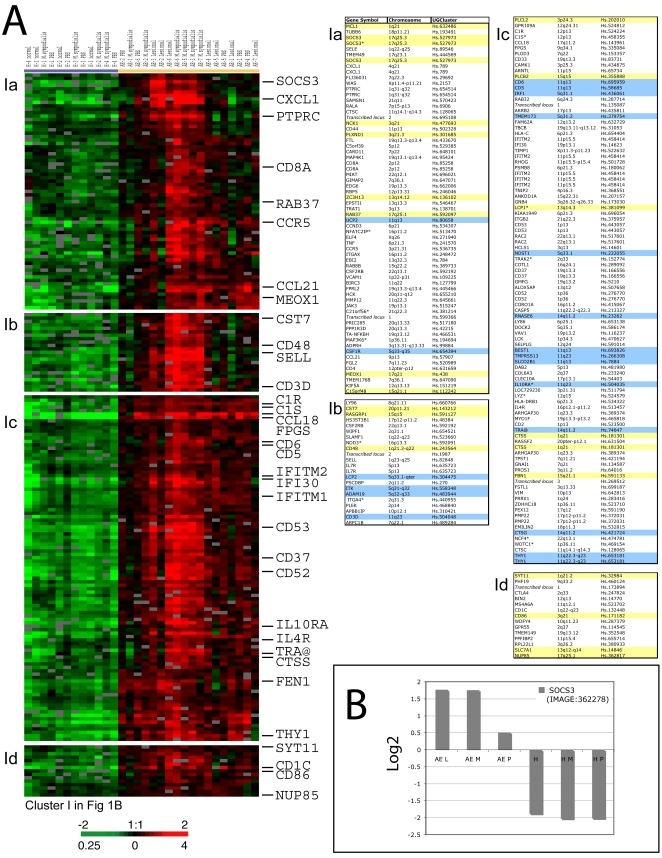
Transcriptional levels of immune and inflammatory genes in AE skin measured using DNA microarrays. (A) A zoomed in picture of the hierarchical cluster analysis showing examples of immune and inflammatory genes that was found to be coordinately over-expressed in AE skin as compared to healthy skin. A detailed list of the genes represented in this cluster is shown in the table (right). Genes localized in genome-wide linkage eczema regions or in AE associated loci are highlighted in blue and yellow, respectively. (B) The *SOCS3* gene was identifiedby the SAM approach to be significantly over-expressed in AE skin as compared to healthy skin. The “contrast value” generated by the SAM program for the *SOCS3* gene is displayed in the graph (y-axis). Each bar represents *SOCS3* transcriptional levels in a group of samples. AE patients: lesional skin (AE L n = 7), *M. sympodialis* (AE M n = 6) and PBS patch-tested skin (AE P n = 5). Healthy control individuals: normal skin (H n = 4), *M. sympodialis* (H M n = 4) and PBS patch-tested skin (H P n = 4).

The most prominently up-regulated genes in AE skin as compared to healthy control skin are genes involved in immune and inflammatory responses (Figure 2A). In general, we found the immune response cluster of genes more highly expressed in *M. sympodialis* patch-test reactive AE skin, as compared to lesional and non-involved/PBS patch tested AE skin and normal healthy skin, which supports the role of inflammatory molecules in active AE skin lesions. Among the genes identified in the “immune cluster” are chemokine family members including CCL18, CCL21 and CXCL1 (Figure 2) and members of the interleukin receptor family such as IL-2R(γ), IL-4R and IL-10RA (Figure 2). Genes encoding cell surface antigens, such as CD5, CD6, CD28, CD37, CD53 and CD86 are also among the genes over-expressed in AE skin (Figure 2). Notably is that many genes identified in the “immune cluster” are localized on chromosomal regions previously linked to AE (Figure 2 and Table 1). Furthermore, we identified a group of coordinately over-expressed genes encoding components (C1R, C1S and C1QB) and regulatory proteins (SERPING1 and CFH) of the classical pathway of complement in AE skin (Figure 2 and Supplementary Table S1). Other interesting genes identified within this cluster are *FCER1G* (1q23), encoding the high-affinity IgE receptor gamma subunit, *TRAα*, encoding the T-cell alpha locus, and *THY1*, encoding a major cell surface glycoprotein characteristic for T-cells (Figure 2 and Supplementary Table S1). *FCER1G*, TRAα and *THY1* are located on previous described AE loci (11q23.3 and 14q11.2).

### Cell specific elevated expression of the Socs3 protein in AE skin

**Figure 3:**
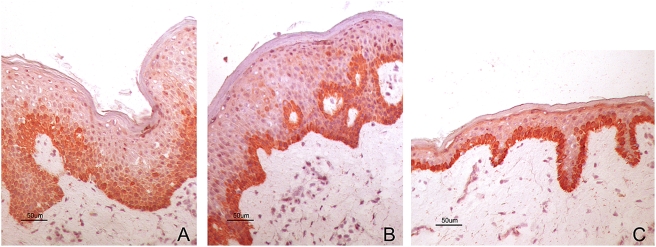
Increased expression of Socs3 on keratinocytes in lesional AE skin. Immunohistochemical staining showing Socs3 expression (A) in lesional AE skin, (B) non-lesional AE skin, and (C) normal skin from a healthy control. Sections were counterstained with hematoxylin to visualize the nucleus.

**Figure 4:**
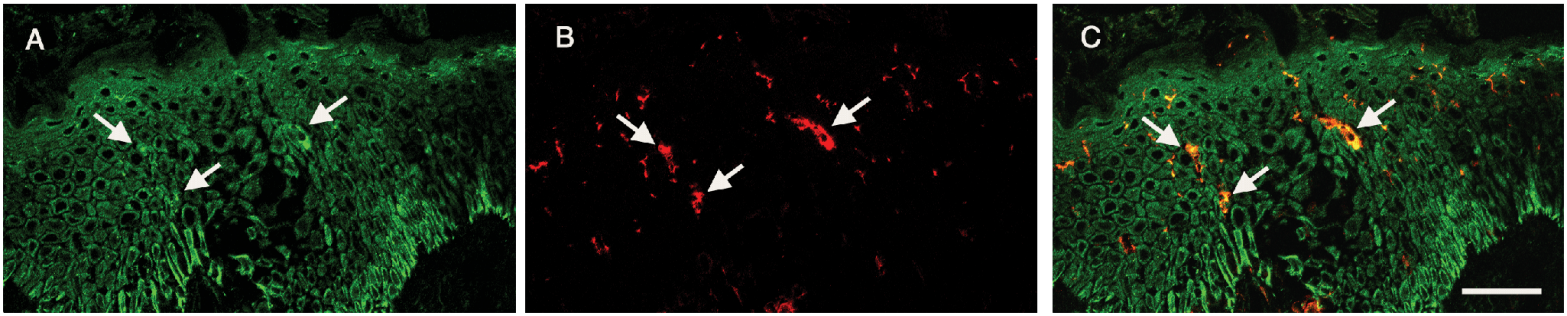
Socs3 is expressed on CD1a^+^ dendritic cells in epidermis. Double immunofluorescensce stainings of lesional AE skin showing (A) expression of Socs3 on keratinocytes and on epidermal dendritic cells, (B) CD1a expression on dendritic cells and (C) co-localisation of Socs3 and CD1a in epidermis. Dendritic cells are indicated by arrows. Scale bar = 50 µm.

*SOCS3*, located in an AE candidate chromosomal region (17q25), was among the genes more highly expressed in AE skin as compared to healthy skin (Figure 2B). To identify *in vivo* patterns of Socs3 protein expression in the skin, we used immunohistochemistry and double immunofluorescent staining methods. Socs3 expression was increased and spread to several layers in epidermis in AE lesional skin (Figure 3A), while in non-lesional AE skin (Figure 3B) and normal healthy skin (Figure 3C) it was predominately in the basal epidermal layer. We next performed double immunofluorescent staining to determine if other cell types besides keratinocytes expressed Socs3 protein in AE epidermis (Figure 4). A Socs3 antibody was co-applied with antibodies for various cell types on AE lesional skin biopsy sections: CD1a for dendritic cells, CD3 for T-cells, and CD68 for monocytes/macrophages. We found that Socs3 was expressed by CD1a^+^ dendritic cells (Figure 4A–C), but not by CD3^+^ T-cells or CD68^+^ cells in AE lesional skin (data not shown).

### Altered lipogenic gene-expression program identified in AE skin

**Figure 5:**
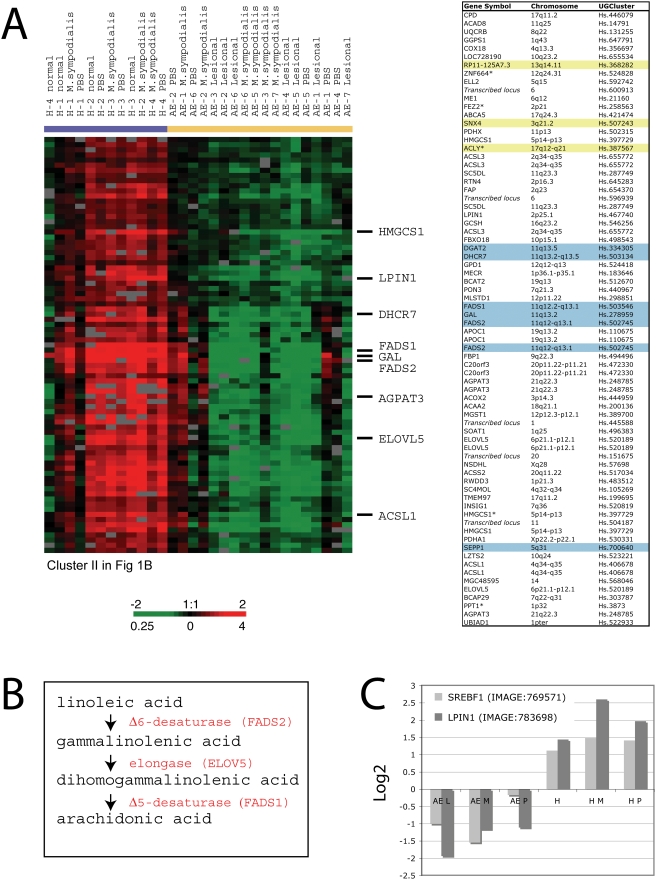
Global down-regulation of a lipogenic expression program identified in AE skin. (A) A zoomed in picture of the hierarchical cluster analysis illustrating a large set of genes involved in lipid metabolism and homeostasis that was found to be coordinately down regulated in AE skin (left). A detailed list of the genes represented in this cluster is shown in the table (right). Genes localized in genome-wide linkage eczema regions or an AE associated loci are highlighted in blue and yellow, respectively. (B) Schematic of enzymes involved in the PUFA pathway. (C) *SREBF1* and *LPIN1* transcript levels were lower in AE skin compared to healthy control skin. The “contrast values” generated by the SAM program for the *SREBF1* and the *LPIN1* gene are displayed in the graph (y-axis). Each bar represents transcriptional levels in a group of samples. AE patients: lesional skin (AE L n = 7), *M. sympodialis* (AE M n = 6) and PBS patch-tested skin (AE P n = 5). Healthy control individuals: normal skin (H n = 4), *M. sympodialis* (H M n = 4) and PBS patch-tested skin (H P n = 4).

The most prominent feature of the AE down-regulated gene cluster is a set of genes with a well-defined role in lipid homeostasis. These include genes that are dedicated to the synthesis and uptake of cholesterol and fatty acids such as ATP-Citrate lyase (*ACL1*), Acyl-CoA synthase (*ACSL1*, *ACSL3*), HMG-CoA synthetase (*HMGCS1*, *HMGCS2*) and HMG-CoA reductase (*HMGCR*) (Figure 5 and Supplementary Table S1). Furthermore, genes encoding key enzymes in the poly-unsaturated fatty acid (PUFA) pathway (*FADS1*, *FADS2* and *ELOVL5*), and an acyltransferase (*AGPAT3*) with a key role in the phospholipid pathway, were identified in the same gene cluster (Figures 5A and B).

Genes encoding eicosanoid lipid messengers and processing enzymes also showed significantly different expression levels in AE skin as compared to the healthy skin. For example, we observed that prostaglandin processing enzymes including PTGES (Prostaglandin E synthetase), PTGIS (Prostaglandin I2) and PTGER3 (Prostaglandin E receptor) were more highly expressed in AE skin as compared to healthy control skin (Supplementary Table S1). Furthermore, genes involved in arachidonic acid metabolism, e.g. genes required for leukotriene synthesis, such as ALOX5AP, ALOX12 and ALOX15B, were differentially expressed in AE skin as compared to healthy skin (Supplementary Table S1).

Among the lipid genes down-regulated in AE skin were transcripts encoding hydroxy steroid dehydrogenase family members and members of the cytochrome P450 family that are essential for steroid hormone biosynthesis [26] (data not shown). Furthermore, among the down-regulated genes in AE skin was lipin-1 (*LPIN1*; Figures 5A and C), which encodes a transcription factor with a suggested role during normal adipose tissue development [27]. The lipin-1 gene was isolated and characterized in 2001 as the gene responsible for fatty liver dystrophy (fld) in mouse [28]. Interestingly, we also found a member of the SREBP family, which is a well-described transcription factor with a major role in regulating genes involved in fatty acid and cholesterol metabolism [29], to be down-regulated in AE skin as compared to healthy skin (*SREBF1*; Figure 5C).

### Altered expression of epidermal barrier function genes in AE skin

**Figure 6:**
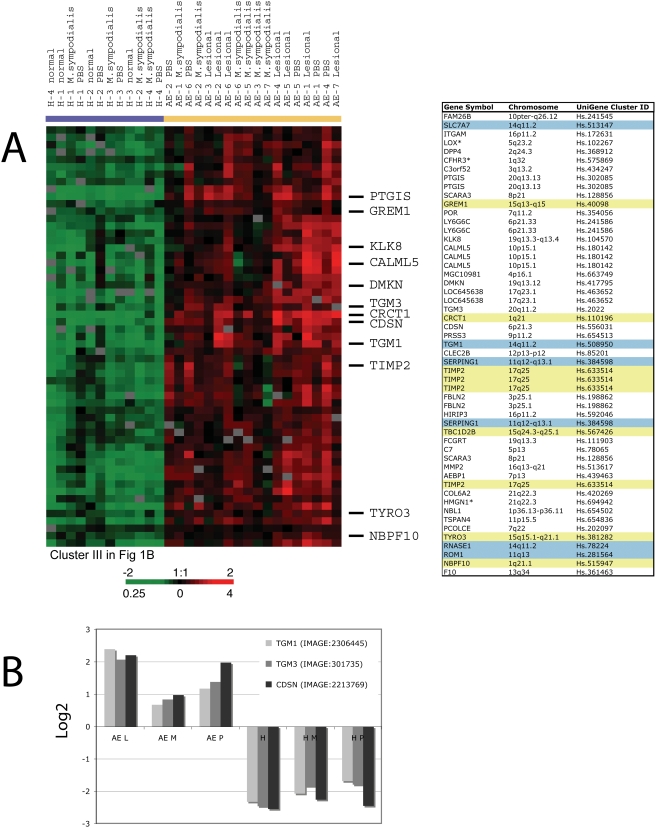
Genes involved in skin barrier function differentially expressed in skin from AE patients as compared to healthy control individuals. (A) A zoomed in picture of the hierarchical cluster analysis illustrating genes involved in skin barrier function that were found coordinately over-expressed in AE skin as compared to healthy skin (left). A detailed list of the genes represented in this cluster is shown in the table (right). Genes localized in genome-wide linkage eczema regions or an AE associated loci are highlighted in blue and yellow, respectively. (B) Members of the transglutaminase family (*TGM1*, *TGM3*) and *CDSN* were among the cornified envelope genes identified by the multi-group SAM approach to be consistently over-expressed in AE skin as compared to healthy skin. The “contrast value” generated by the SAM program for these genes is displayed in the graphs (y-axis). Each bar represents transcriptional levels in a group of samples. AE patients: lesional skin (AE L n = 7), *M. sympodialis* (AE M n = 6) and PBS patch-tested skin (AE P n = 5). Healthy control individuals: normal skin (H n = 4), *M. sympodialis* (H M n = 4) and PBS patch-tested skin (H P n = 4).

We found alterations in expression of a set of genes encoding structural components and key enzymes involved in forming the cornified envelope in AE skin. Two transcripts encoding members of the transglutaminase family (*TGM1* and *TGM3*) had consistently elevated expression in AE skin as compared to healthy skin (Figures 6A and B). Transglutaminases are key enzymes that catalyze the cross-linking of epidermal proteins during formation of the stratum corneum. A similar expression pattern, as observed for the *TGM* genes, was found for *CALML5* (alias *CLSP*), which encodes a calmodulin-like skin protein that previously has been shown to associate with the TGM3 protein [30]. Furthermore, as seen in Figure 6A, *CDSN*, which encodes a desmosomal-associated protein expressed during terminal keratinocyte differentiation [31], was co-expresed with the *TGM* transcripts. Desmosomes are cell–cell adhesion sites that provide mechanical integrity to the tissues by anchoring keratin filaments to the site of cell–cell adhesion. The hypothesis that constituents of the desmosomal cell–cell junctions may have altered expression in the epidermal layers of AE skin is further supported by consistently lower expression of *DSG2* in AE skin as compared to healthy skin (Supplementary Table S1). *DSG2* encodes a membrane spanning glycoprotein of the desmosome, and is localized to chromosome 18 in a locus previously linked to AE (18q12.1 [7]). Furthermore, we identified elevated expression of the corneum chymotryptic enzyme (SCCE/KLK7) in AE skin (Supplementary Table S1). This result is supported by recent work from Komatsu et al. (2007) that reported elevated expression of several kallikrein family members in the stratum corneum from AE patients [32], and furthermore by work from Vasilopoulos that identified genetic association between the corneum chymotryptic enzyme (SCCE/KLK7) and AE [33].

Genes encoding molecules critical for the extracellular matrix (ECM) architecture (COL6A1, COL6A2 and COL6A3) and ECM re-modeling enzymes (TIMP1 and TIMP2) were differentially expressed in AE skin (Figure 6A and Supplementary Table S1). Many of these genes with an important role in constructing the skin barrier, as well as genes encoding cell adhesion and ECM molecules, are localized on chromosomal regions previously linked or associated to AE (Figure 6 and Table 1) suggesting that they serve as potential AE candidate genes.

### Elevated expression and localization of the TGase1 protein in AE skin

**Figure 7:**
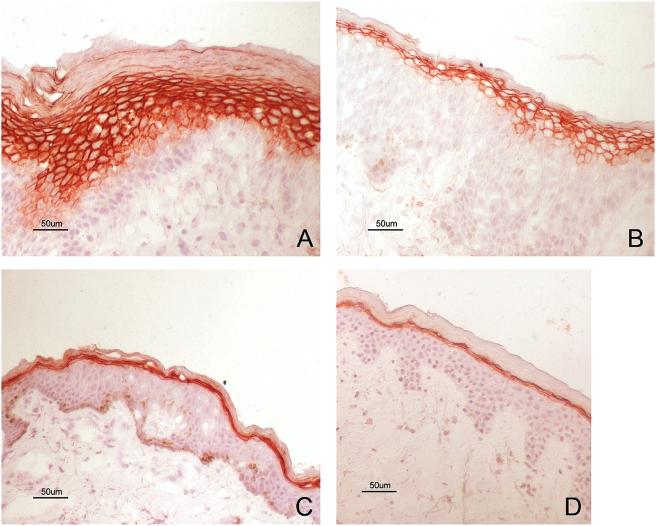
Increased TGM1 protein expression in AE skin. Immunohistochemical staining of TGM1 expression in lesional AE skin (A) and in positive APT reaction to *M. sympodialis* extract (B), non-lesional PBS patch-tested AE skin (C) and normal healthy skin (D). Sections were counterstained with hematoxylin to visualize the nucleus.

*TGM1*, which is localized in a chromosomal region (14q12) previously linked to eczema [34], was among the epidermal differentiation genes found to be over-expressed in lesional AE skin as compared to healthy skin. By immunohistochemistry, we confirmed that epidermal transglutaminase 1 (TGase1) protein expression is increased in AE skin (Figure 7). We found that both expression and the localization of TGase1 were altered in lesional AE skin. TGase1 was localized to several cell layers and spread down deep in epidermis in lesional AE skin (Figure 7A), while in non-lesional PBS patch-tested AE skin (Figure 7C) and normal healthy skin (Figure 7D) it was expressed as a sharp line in the outermost layer of epidermis. As illustrated in Figure 7, TGM1 protein levels could be induced by the application of *M. sympodialis* to non-involved AE skin.

### Enrichment of AE related chromosomal regions

We noticed that many of the genes that were found by the array experiments to be consistently differentially expressed in AE and healthy skin are localized to previously described AE susceptibility chromosomal regions (reviewed in [2]). To further test this hypothesis, we used the previously described “gene module map method” [24] to identify significantly enriched cytobands in the AE microarray data set. The set of ∼4,000 AE genes were analyzed for their enrichment in 624 gene sets composed of human cytoband regions [35]. As illustrated in Table 1, we found 70 cytobands that were significantly enriched in the AE data set (*P*<0.05; FDR<0.05). Interestingly, 20 of these significantly enriched cytobands represent chromosomal regions previously described to be linked or associated to AE either by genome-wide linkage studies, selective region-specific linkage mapping or candidate gene studies in AE [2], [34], [36]–[40]. The observation that 20 AE-linked cytobands were found in the top 70 hits is highly significant (*P*<10^−8^, hypergeometric distribution, with 624 total cytobands in the genome), whereas only 2 cytobands are expected to overlap by chance alone. Notably, we found that genes induced in AE are more enriched from AE-linked cytobands (17 of 20) than genes repressed in AE (3 of 20).

Among the enriched AE-linked cytobands identified are 1q21, 3p25.3 and 15q21.3, which all previously have been identified as AE susceptibility regions in genome-wide linkage analyses [5]–[7], [38]. The 1q21 region encodes several epidermal differentiation genes including the *FLG* gene that recently has been linked to AE susceptibility [15]. However, genes from the 3p25.3 and 15q21.3 regions have not been identified or systematically tested for a role in AE. Furthermore, among the significantly enriched cytobands were several AE gene candidate regions previously described in the literature to be associated to AE [2], including chromosomal region 2q33 (*CTLA4*), 3p21 (*TLR9*), 5q32 (the cytokine cluster), 11q12 (*FCERIβ*), 12q13 (*TIM1*), 14q11 (*MCC*), 16p11 (*IL4R*) and 19q13 (*SCCE* and *TGFB1*) (Table 1).

The microarrays also identified significantly enriched cytobands that were previously not linked to AE (Table 1). Interestingly though, some of these chromosomal regions have been linked to other diseases. For example, we found an enrichment of cytobands in the AE data set that overlap with chromosomal regions previously identified to be associated with psoriasis (1q21;PSORS4 and 6p21;PSORS1) and asthma (5q23-32, 6p21, 11p15 and 19q13). In fact, 6p21.2 was among the most significantly enriched cytobands identified in the AE data set (*P*<10^−9^, Table 1). The 6p21 region (PSORS1) contains multiple genes in tight linkage disequilibrium, including the *HLA* gene cluster and the *CDSN* gene.

All specific genes in AE susceptibility chromosomal regions have not yet been identified or systematically tested for a role in AE. Examples of genes identified by the microarrays to be consistently differentially expressed between AE and healthy skin and localized within significantly enriched cytobands are shown in Supplementary Table S3. In summary, we found that there is a significant enrichment of differentially expressed genes in AE with cytobands associated to the disease, and furthermore new chromosomal regions were found that could potentially guide future region-specific linkage mapping in AE.

## Discussion

AE is a chronic inflammatory skin disorder that results from a complex interaction of genetic and environmental factors [9]. Although several chromosomal regions have been identified that are linked to AE susceptibility, it is yet not fully understood what specific genes and mechanisms underlie the development of AE. The DNA microarray technique [41] has recently been utilized successfully in the search for new candidate genes in complex diseases. Large–scale expression profiling of chronic inflammatory skin disorders such as eczema and psoriasis have previously been performed using the Affymetrix GeneChip [12], [14], [42]–[44]. Although previous expression profiling studies have highlighted sub-groups of genes such as a set of innate immunity genes [12] and the “epidermal differentiation cluster” [14], or potential gene candidates including NELL2, CCL18, AQP3 and tenascin-C [42]–[44] that are deregulated in AE, the complete genome-wide picture of AE is still far from understood. To further advance the understanding of genes underlying the development of AE, and to test the impact allergens may have on the global expression pattern in AE skin, we used cDNA microarrays to characterize the global gene-signature in lesional skin and patch-tested skin from eczema patients and corresponding skin from healthy individuals. Our analysis provides striking evidence for global differences in the transcriptional program between skin from AE patients and healthy individuals. The most prominent feature of the gene-signature identified in AE skin was a reciprocal pattern of induced inflammatory genes and reduced lipid genes. Among the genes identified in this study are many that are localized to chromosomal regions previously associated with AE and thus are new potential AE candidate genes.

The large set of coordinately up-regulated immune and inflammatory related genes identified in AE skin includes genes encoding cytokines, chemokins and cell-surface antigens. The increased expression of inflammatory genes can be explained both by increased numbers of infiltrating cells, mainly T-cells and dendritic cells, and by activation of these cells. For example, among the most consistently highly expressed chemokines in AE skin was *CCL18* that is known from previous work to be highly expressed in AE skin by dendritic cells [42], [45]. A novel finding in our study was that components and regulatory proteins of the classical pathway of complement were coordinately over-expresed in AE skin. Complement is an essential component of the immune system and is of relevance for the destruction of invading microorganisms. However, excessive complement activation contributes to undesired tissue damage and the role of complement in other inflammatory diseases has previously been reported [46].

*SOCS3* was also among the inflammatory genes found to be more abundant in AE lesions as compared to healthy skin. This gene encodes a suppressor of cytokine signaling and is localized in an AE candidate chromosomal region (17q25). It has previously been reported that *SOCS3* is predominantly expressed in T_H_2-like cells and has an important role in regulating the onset and maintenance of T_H_2-mediated responses [47]. In contrast to the report from Seki et al. (2003), we did not find T-cells expressing Socs3 in AE skin. However, keratinocytes and CD1a^+^ dendritic cells in the skin expressed the protein in AE lesional skin. The Socs3 protein was also expressed by basal keratinocytes in normal healthy skin. Whether *SOCS3* plays an immunoregulatory role in keratinocytes, or if it might be involved in regulating keratinocyte cell proliferation and differentiation [48] needs to be further investigated. It is unknown what precise role *SOCS3* plays in different cell types in the skin. Its increased expression in AE epidermis may play a role in developing the disease, or it may be a secondary effect of the inflammatory condition of the skin. While Socs3 protein levels are found to be up-regulated in AE skin, the reverse pattern was observed in psoriatic skin [49], which is a chronic inflammatory T_H_1-related skin disease. It has been proposed that microRNAs (mir-203) acting in the *SOCS3* 3′UTR are the regulators of reduced Socs3 protein expression observed in psoriatic skin [49]. It is unclear how Socs3 expression is regulated in AE skin. However, a recent genetic study has demonstrated an association between a haplotype in the *SOCS3* 5′region and AE, which suggest that molecules acting in the 5′region may alter Socs3 expression in AE skin [50].

We demonstrate here that by applying *M. sympodialis* extract to non-lesional AE skin in *M. sympodialis* sensitized patients, a global expression signature can be induced that is remarkably similar to that identified in lesional AE skin. The *M. sympodialis* induced transcripts are dominated by immune related genes such as the IL-4 receptor, which previously has been described in AE [51]. The IL-4 signaling pathway are among the candidates considered for the treatment of allergic inflammation [52]. Data presented here supports the hypothesis that *M. sympodialis* can act as an aggravating factor in AE pathogenesis. It is still unclear, however, why AE patients can be hypersensitive to a fungi that is part of the normal human skin microflora. One hypothesis is that a defective skin barrier in AE patients allows allergens to penetrate and trigger an immunological response in the skin [1].

We found a large set of down-regulated genes in AE skin with a well-defined role in lipid homeostasis. Among these genes are classical genes encoding enzymes involved in fatty acid and cholesterol metabolism, but also genes encoding enzymes involved in poly-unsaturated fatty acid (PUFA) metabolism. The role of lipids in AE pathogenesis has previously been discussed. For example, it has been shown that ceramide levels are reduced in AE skin compared to healthy skin, and furthermore altered expression of ceramide processing enzymes has been seen in AE skin [17]. However, this is the first time that a global reduced lipogenic expression program has been demonstrated in AE skin. Coordinate down-regulation of a large set of lipid processing genes in AE skin could explain an impaired lipid balance previously observed in these patients, which may be a key factor underlying the cause of a disrupted skin barrier in AE patients. Of particular interest is the reduced expression of genes encoding PUFA processing enzymes, delta-5-desaturse (*FADS2*) and delta-6-desaturse (*FADS1*), identified here in AE skin. PUFAs are important constituents of phospholipids in cell membranes, assuring the correct environment for membrane protein function, maintaining membrane fluidity, and moreover PUFAs have been described to play a role in regulating gene transcription [53]. In addition, some PUFAs, particularly arachidonic acid, act as substrates for the synthesis of eicosanoids (i.e. prostaglandins and leukotrienes), which are involved in regulating inflammatory processes and immune cell responses. A disrupted PUFA balance may thus have a large impact on various cellular processes. Interestingly, there are a number of observations in AE patients with significantly higher levels of the first substrate of the PUFA pathway, linoleic acid, and significantly lower levels of the downstream metabolites, γ-linolenic, dihomo-γ-linolenic, and arachidonic acids in eczema [16], [54], [55]. Furthermore, levels of linoleic acid metabolites have been correlated with transepidermal water loss in children with eczema [55]. To date there has been two mutually exclusive hypotheses regarding the altered PUFA composition observed in AE patients [56]. Our results clearly supports the hypothesis that lower levels of PUFA metabolites are due to impaired desaturase enzyme activity and thus impaired synthesis of these molecules, and not that low levels of PUFA metabolites are due to increased consumption in inflammatory processes. Genetic variants in the *FADS1* and *FADS2* genes have recently been associated with fatty acid composition in phospholipids [57]. Since the *FADS* gene cluster are located on 11q12-q13.1, a chromosomal region previously linked with allergic diseases [34], it would be intriguing to test if *FADS* polymorphisms are associated with AE.

It is currently unclear what controls the well-coordinated regulation of lipid processing enzymes in AE skin. It is intriguing to speculate that members of the SREBP transcription factor family, which has a well-described role in regulating genes involved in fatty acid and cholesterol metabolism, may play a key role in the global down-regulation of lipid genes in AE patients. In fact, we found *SREBP1* to be less expressed in skin from AE patients as compared to healthy controls. Another tempting hypothesis is that nuclear receptors, such as LXR and RXR, may have an influence on the observed reciprocal gene-expression program of inflammatory and lipid genes in AE skin. Members of the liver-X-receptor family have previously been described in regulating reciprocal expression of inflammatory and lipid genes on a large scale [58].

The hypothesis that barrier dysfunction is a key factor in the pathogenesis of AE was recently strengthened by work from Palmer et al. (2006), demonstrating that filaggrin (*FLG*) is an AE susceptibility gene [59]. This study and work from others spread light on structural proteins of the cornified envelope (CE), including filaggrin and loricrin, and their role in altered barrier function in AE patients [14], [15]. We report here that *TGM* genes, which encode enzymes critical for constructing the cornified cell envelope architecture, are differentially expressed in skin from AE patients and healthy control individuals. TGM proteins catalyse the cross-linking of structural proteins of the CE and lipids. This process is important in the terminal differentiation of the epidermis, and thus critical for the formation of the stratum corneum, the outermost layer of the skin [60], [61]. Interestingly, the same loss-of-function mutation in *TGM1* and *TGM5* have been shown to cause lamellar ichthyosis, a disease characterized by excessive scaling and shedding of the outer epidermis, and peeling skin syndrome, respectively [62]–[64]. The glycine residue responsible for this loss-of function mutation (G113C) is conserved in all known TGMs and lies close to the catalytic domain of the enzyme [62]. Interestingly, all epidermal *TGM*s map in genomic regions that have previously been linked to AE susceptibility (TGM1;14q12, TGM3;20p13 and TGM5;15q15) [5], [6], [34]. It has not been investigated if *TGM*s are genetically associated to AE, nor is it known if polymorphisms or mutations near the enzymatic active site may affect TG activity in AE patients. Notably, filaggrin and loricrin, which encode two important structural proteins of the CE and identified by Sugiura et. al. (2005) to be differentially expressed in AE skin [14], were not included on the microarrays used in this study and thus the transcript levels for these genes are not reported in our patient material.

Skin barrier dysfunction could possibly also be explained by altered cell–cell adhesion function in the skin. We found genes encoding common constituents of the desmosome complex, such as *CDSN*, to be differentially expressed in AE skin. Desmosomes are cell–cell adhesion sites that provide mechanical integrity to tissues by anchoring keratin filaments to the site of cell–cell adhesion. Changes in transcriptional levels of *CDSN*, which encodes a desmosomal-associated protein expressed during terminal keratinocyte differentiation, may lead to skin disease. In fact, the *CDSN* gene has recently been associated to psoriasis (reviewed in [65]), but has not been associated with AE. The data presented here support the hypothesis that genes involved in forming the outermost protective layer of the skin, the stratum corneum, play a critical role in AE pathogenesis. Furthermore we identified altered expression of stratum corneum proteases in AE skin suggesting that not only the construction of the cornified envelope is altered in AE skin, but also that abnormal renewal and removal of corneocytes (the so called “desquamation process”) may be crucial for skin barrier dysfunction in AE skin.

Finally, we asked the questions what cytobands were enriched in the microarray data set, and if these cytobands may represent previously known AE susceptibility regions. Interestingly, we were able to discover correspondence of differentially expressed genes in AE and disease susceptibility regions. Enrichment was also found for cytobands representing chromosomal regions not previously described in the disease that could potentially guide future region-specific linkage mapping in AE.

In conclusion, we have used human DNA microarrays to identify a molecular picture of the programmed responses of the genome to AE. The most prominent feature of the global expression program identified in AE skin was a reciprocal pattern of induced immune response genes and reduced expression of lipid metabolism genes. Furthermore, we identified transglutaminases, key enzymes involved in cornified envelope assembly, to be enhanced in AE skin compared to healthy skin. Alterations in genes involved in cornified envelope formation and lipid homeostasis in AE skin support the hypothesis that skin barrier dysfunction is crucially involved in the pathogenesis of AE [66]. We believe that further understanding of these gene candidates may lead to new therapeutic strategies for AE patients in the future.

## Materials and Methods

### Subjects for microarray analysis

Seven patients with AE, recruited and investigated at the Karolinska University Hospital, Solna (Table 2), and four healthy controls were included in the study. The healthy controls had no clinical symptoms or history of allergy or skin diseases and were Phadiatop® (Phadia AB, Uppsala, Sweden) negative. Inclusion criteria for the AE patients were diagnosis according to the UK working party criteria [67]. Also, the eczema lesion had to be present in other regions than only the hands. The eczema started before the age of one year in 6/7 patients. They all had an exacerbation of symptoms lasting for more than six months before the investigation. All patients suffered from allergic symptoms in the airways, 5/7 with ongoing symptoms at the time of examination. Exclusion criteria were other skin diseases than AE, autoimmune diseases, immune deficiencies, malignant diseases, pregnancy or lactation, immunosuppressive treatment, and age below 18 or above 55 years. Systemic glucocorticoids, systemic antifungal treatment or UV therapy was not allowed for 2 months before the investigation and topical glucocorticoids were not allowed on the test sites for one week before the study. Antihistamines were withdrawn 5 days before the investigation. All participants gave their informed consent. The participant consent was written. The study was approved by the Regional Ethics Committee.

**Table 2:** Characterization of the AE patients.

No	Gender	Age (years)	Asthma/Rhinitis	SCORAD^a^	s-IgE kU/L^b^	Phadiatop^c^	*M. sympodialis* specific IgE kU/L^d^	SPT^e^ mm	APT^f^
1	M	21	+	57	5700	+	16	5	+
2	F	28	+	57	5300	+	4,8	4,5	+
3	F	34	0	24	98	0	<0,35	6	++
4	F	35	+	25	7000	+	15	5	++
5	F	22	+	22	140	+	1,6	6	+
6	M	43	+	78	4710	+	2,4	6,5	+++
7	F	31	+	48	59	+	2,6	4,5	++

a SCORAD [70].

b ImmunoCAP™ (Phadia AB, Uppsala, Sweden), reference range 1.6–122 kU/L.

c Phadiatop® (Phadia AB), serum IgE to any of 11 common aeroallergens.

d Specific serum IgE for *M. sympodialis* ATCC strain 42132, ImmunoCAP™ (m70, Phadia AB).

e SPT = skin prick test evaluated after 15 min and graded as mean diameter (mm) of the wheal. A reaction of 3 mm or more was considered positive.

f APT = atopy patch test evaluated after 48 h, + = erythema, infiltration, few papules; ++ = erythema, infiltration, papules and small vesicles; +++ = erythema, infiltration, papules and large vesicles [22].

F = female, M = male.

### Skin prick test and atopy patch test

Skin prick test (SPT) and atopy patch test (APT) were performed with extract of the yeast *M. sympodialis* prepared from strain no. 42132, American Type Culture Collection (ATCC) as previously described [68]. For SPT the protein concentration of the extract was 100 µg/mL. Histamine dihydrochloride (10 mg/mL, ALK, Hørsholm, Denmark) was used as a positive and PBS as a negative control. APT was performed on healthy individuals and non-lesional, tape-stripped skin of the back of AE patients. The *M. sympodialis* extract (20 µL, 5 mg/mL) was applied on paper discs in Finn chambers (8 mm; Epitest Ltd Oy, Tuusula, Finland). The tests were evaluated after 48 h under coded conditions (See Table 2). PBS (Phosphate Buffered Saline) was used as a negative control.

### Skin biopsy collection and RNA preparation

Skin biopsies were collected from two groups of individuals: AE patients sensitized to *M. sympodialis* (Table 2) and non-atopic healthy individuals. Punch biopsies (4 mm) were taken from *M. sympodialis* extract and PBS patch tested skin after 48 h from both AE patients and healthy controls, and from lesional skin in AE patients and normal skin from healthy control individuals. The skin biopsies were snap frozen on dry ice and stored at −80°C. Total RNA was extracted from each skin biopsy using Trizol (Invitrogen, Carlsbad, CA, USA), and total RNA was linearly amplified according to the Ambion MessageAmp procedure (Cat #1750). This amplification procedure is based on antisense RNA (aRNA) amplification first described by Van Gelder and Eberwine [69]. The amplified RNA was used as template for reverse transcription in the presence of CyDye-labeled dNTPs to generate labeled cDNA for microarray analysis. Due to technical reasons, RNA from 3 biopsies did not get further processed for microarray hybridization.

### cDNA microarrays, hybridization, data filtering and analysis

Human cDNA microarrays were used containing 41,792 elements that represents approximately 24,500 unique genes (based on Unigene clusters) manufactured in the Stanford Microarray Facility (www.microarray.org). Fluorescently labeled cDNA prepared from amplified RNA was hybridized to the array in a two-color comparative format, with AE patient- or healthy control samples labeld with Cy-5, and a reference pool of human mRNAs (Stratagene) derived from ten cell lines labeled with Cy-3.

Array images were scanned by using an Axon Scanner 4000B (Axon Instruments, Union City, CA), and data was analyzed by using GenePix 3.0 (Axon Instruments). Data was normalized and retrieved as the log_2_ ratio of fluorescence intensities of the sample (Cy5) and the reference (Cy3). We next filtered the data to exclude elements that did not have at least a 2-fold intensity over background ratio, in at least 80% of the arrays. These filtered genes were analyzed by the multi-class Significance Analysis of Microarrays (SAM) algorithm [23] to select a set of ∼4000 genes that were consistently differently expressed between skin from AE patients and control individuals, with a false discovery rate less than 0.26% (Supplementary Table S1). An overview of the skin tissue samples used for the multi-class SAM microarray analysis are shown in Supplementary Table S4. The abundance of each transcript measured in a skin biopsy specimen relative to the common reference is represented in Figures 1, 2, 5 and 6 by color; red for expression levels above the mean for that gene and green for expression levels below the mean. The multi-group SAM approach calculates a “contrast value” for each gene, e.g. the standardized mean difference between the genes expression in the class, versus its overall expression. “Contrast values” for 4102 genes are displayed in Figure 1A and the contrast for *SOCS3*, *SREBF1*, *LPIN1*, *TGM1*, *TGM3* and *CDSN*, are illustrated in the graphs in Figures 2, 5 and 6.

The full microarray data set described in this manuscript is available at the Stanford Microarray Database (SMD) http://microarray-pubs.stanford.edu/eczema and at the NCBI Gene Expression Omnibus (GEO) database http://www.ncbi.nlm.nih.gov/geo/info/linking.html (GEO accession: GSE12511).

### Identification of significantly enriched cytoband regions and Gene Ontology terms in the microarray data set

2194 genes induced and 1908 genes repressed in AE (as is illustrated in Figure 1) were analyzed for their enrichment in 624 gene sets composed of human cytoband regions [35] and 1665 gene sets composed of Gene Ontology (GO) terms [25]. Significant enrichment of AE-associated genes (*P*<0.05; corrected for multiple hypotheses using FDR) was determined using the “gene module map method” implemented in Genomica [24]. To test the overlap of cytobands discovered by altered gene expression levels by microarray versus cytobands identified by genetic studies of AE, we scored for identical cytobands between the Genomica output versus AE-linked or associated cytobands described in the literature [2], [37]–[40]. Among 70 cytobands that showed coordinate mRNA level changes in AE, 20 were previously linked or associated to AE by genetic studies (28% overlap), whereas only 2 cytobands (3% overlap) were expected by chance alone (*P*<10^−8^, hypergeometric distribution). Not all cytobands genetically linked to AE have been discovered, which might increase the pre-test probability of the overlap and decrease the significance of the above findings. We note that even assuming a pretest probability of 10% overlap (i.e. discovery of three times more genetic loci linked to AE), the overlap between microarray and genetic cytobands is highly significant at 10^−6^.

### Immunohistochemical and double immunofluorescence staining of skin biopsies

Skin biopsy specimens from an independent set of AE patients and healthy control individuals were used for immunohistochemistry. Staining results from a selected set of AE patients (n = 3) and healthy controls (n = 2) are illustrated in Figure 3, 4 and 7. The inclusion and exclusion criteria were the same for these individuals as is described above. Six µm thick cryo sections were prepared and put on glass slides, fixed in acetone and used for the ABC-ELITE (Vector Laboratories Inc. Burlingame, CA, USA) immunohistochemical staining method according to the manufacturer's instructions. The sections were incubated with rabbit anti-Socs3 antibodies (dilution 1/100 from Santa Cruz Biotechnology Inc, Santa Cruz, CA, USA) followed by a biotinylated goat-anti-rabbit secondary antibody (1/200, Vector Laboratories Inc.), or a monoclonal mouse antibody against transglutaminase 1 (1/250, Biogenesis Ltd, Poole, UK) followed by a biotinylated horse-anti-mouse secondary antibody (1/400, Vector Laboratories Inc., Inc., Burlingame, CA, USA). The specimens then were allowed to react with preformed avidin-biotin-enzyme complex (ABC-ELITE reagent, Vector Laboratories Inc.) for 30 min. The developing step was incubation with 3-amino-9-ethylcarbazole (AEC) substrate for 15 min. The slides were counterstained with Mayer's haematoxylin. Irrelevant mouse Ig or normal rabbit serum, respectively, were used as negative control and gave no staining.

For double immunofluorescence stainings, the skin sections were incubated with rabbit anti-Socs3 antibodies (1/100) from Santa Cruz Biotechnology Inc and the monoclonal antibodies against CD1a (dilution 1/10), CD3 (dilution 1/5) or CD68 (dilution 1/25), all from BD Biosciences Pharmingen, San Jose, CA, USA. Sections were next incubated with Alexa Fluor 488 goat anti-rabbit (green fluorescence, 1/500) and Alexa Fluor 546 goat anti-mouse (red fluorescence, 1/500) from Invitrogen, Eugene, OR, USA. The sections were evaluated using a Leica TCS SP2 confocal laser scanning microscope system, equipped with an inverted Leica DM IRBE microscope, an argon laser, and two HeNe lasers (Leica Microsystems, Germany). Leica confocal software was used to acquire and visualize the data. Staining was not observed when irrelevant isotype-matched mouse antibodies were used or when primary antibodies were omitted.

## Supporting Information

Table S1 Genes differentially expressed between skin from AE patients and healthy control individuals. Shown are a detailed list of ∼4,000 genes identified by the multi-group SAM approach to be consistently differentially expressed between AE and healthy skin (FDR<0.003). This set of genes is the same genes that are shown in Figure 1.
(0.74 MB PDF)

Table S2 Enriched GO terms. Shown are Gene Ontology (GO) terms significantly enriched in 2181 genes induced (Gene Set 1) or 1896 genes repressed (Gene Set 2) in AE (P<0.05; FDR<0.05).
(0.05 MB PDF)

Table S3 Genes located in enriched AE-linked chromosomal regions. Shown are differentially expressed AE genes located within disease susceptibility chromosomal regions (cytobands) that are enriched in 2194 genes induced (Gene Set 1) or 1908 genes repressed (Gene Set 2) in AE (P<0.05; FDR<0.05).
(0.06 MB PDF)

Table S4 Overview of skin biopsy samples that were used in the SAM approach to identify differentially expressed genes in AE and healthy skin. Shown are six groups of samples representing skin from atopic eczema patients (SAM group 1-3: AE-L, AE-M, AE-P) and healthy control individuals (SAM group 4-6: H-N, H-M, H-P). Corresponding microarray slide name are given for each sample using the same nomenclature as is used in the Stanford Microarray Database (SMD).
(0.05 MB PDF)
