# Supplementary material for: Global Expression Profiling in Atopic Eczema Reveals Reciprocal Expression of Inflammatory and Lipid Genes
Source: PLoS One. 2008 Dec 24;3(12):e4017. doi: 10.1371/journal.pone.0004017 (PMC2603322; doi:10.1371/journal.pone.0004017)
Supplement: Table S1 — Genes differentially expressed between skin from AE patients and healthy control individuals. Shown are a detailed list of ∼4,000 genes identified by the multi-group SAM approach to be consistently differentially expressed between AE and healthy skin (FDR<0.003). This set of genes is the same genes that are shown in Figure 1. (0.74 MB PDF) [file pone.0004017.s001.pdf]

Supplementary Table S1. Genes differentially expressed between skin from AE patients and healthy control individuals. Shown are a list of ~4,000 genes identified by a multi-group SAM approach to be consistently differentially expressed between AE and healthy skin (FDR<0.003). Six groups were used in the SAM analysis representing atopic eczmea (AE-L, AE-M, AE-P) and healthy (H-N, H-M, H-P) skin biopsy samples. The “contrast value” for each gene calculated by the SAM program are given in the table, e.g. the standardized mean difference between the genes expression in the class, versus its overall expression.

Gene ID	Gene Name	contrast-1 AE:L	contrast-2 AE:M	contrast-3 AE:P	contrast-4 H:N	contrast-5 H:M	contrast-6 H:P
IMAGE:33478	FPGS Hs.33508	2.337336877	2.85102054	2.47270515	-3.14968356	-4.16691931	-3.5568147
IMAGE:982385	CRISP3 Hs.4044	-2.1088691	-2.33120557	-1.64640924	3.89010241	2.25926439	2.56875676
IMAGE:502155	PTGIS Hs.30208	2.980389292	1.13158331	2.68863679	-3.16025985	-3.51714961	-2.85134543
IMAGE:123926	CTSK Hs.63246	2.814971321	2.01587231	2.36628467	-3.16760007	-4.14289794	-2.89362327
IMAGE:138991	COL6A3 Hs.233	2.353657571	2.59859195	1.23417695	-3.55874343	-2.72201229	-2.69033974
IMAGE:1876531	CTSG Hs.42172	1.642872788	1.77926037	2.77376731	-2.25467441	-3.42234427	-2.9233902
IMAGE:2406134	MAPKAPK3 Hs.2	2.215373296	2.08665788	2.34012824	-3.99935363	-3.04141388	-2.33743956
IMAGE:357681	NCF4 Hs.47478	1.665652497	1.79234282	2.07556607	-2.34297002	-3.02287474	-2.4156058
IMAGE:428103	CD1C Hs.1311 C	1.755770258	1.65486323	2.21933104	-2.80315254	-2.28616782	-2.80079367
IMAGE:124822	SHANK2 Hs.268	-2.347960335	-2.04257271	-1.65049441	2.93549181	2.49865206	3.21477372
IMAGE:857640	COL6A2 Hs.420	1.803825747	1.31147332	2.71574806	-2.99917919	-2.8835834	-2.18487109
IMAGE:2418774	MYBPC1 Hs.485	-2.05329443	-1.80283176	-1.43890648	2.28499061	2.497733	2.80009878
IMAGE:1912578	PTGIS Hs.30208	2.259755241	1.21038932	1.97135335	-2.5157284	-2.64733348	-2.50634665
IMAGE:376416	C1R Hs.524224	1.880238843	1.80316851	1.62222077	-3.34328684	-2.10681061	-2.10278954
IMAGE:823851	AEBP1 Hs.4394	1.984573132	1.460248	2.47426395	-2.79376058	-3.17618121	-2.29011983
IMAGE:784178	PTGER3 Hs.445	3.010122163	0.86334025	2.03877305	-3.21563832	-2.4535772	-2.68944441
IMAGE:768497	CCL18 Hs.1439	1.557692326	1.56070408	1.62244501	-1.69457389	-2.59227697	-2.41880001
IMAGE:2213769	CDSN Hs.55603	2.206490473	0.97884385	1.97795482	-2.54397458	-2.25830502	-2.44816541
IMAGE:898305	NBL1 Hs.63238	2.060587323	1.37631636	2.19393243	-3.25720693	-2.55319738	-2.08736676
IMAGE:489089	COL6A1 Hs.474	1.562189986	1.78310223	1.86926717	-2.83588862	-2.4559379	-2.06269577
IMAGE:358599	C1orf42 Hs.110	2.18222017	1.09851283	1.54253499	-2.27942359	-2.24849326	-2.32135138
IMAGE:2307518	EMILIN2 Hs.532	1.793540244	1.92440949	1.7575512	-2.49455271	-2.66357998	-2.61573091
IMAGE:299539	FGF12 Hs.5847	1.673540529	1.55141011	1.91877842	-2.94250088	-2.27811138	-2.01528672

IMAGE:1870512	TNFRSF14 Hs.51	1.374241371	1.5114805	2.39750878	-2.39089283	-2.5425168	-2.39205916
IMAGE:377346	C1S Hs.458355	1.912509355	1.54586235	1.65819675	-2.98788396	-2.34235119	-1.93006833
IMAGE:754378	PTGES Hs.1466	1.956240595	1.90354068	1.29126351	-3.73882341	-1.68711998	-1.97780791
IMAGE:246079	TRAK2 Hs.1527	1.470347722	2.41462349	0.66036895	-2.42687511	-1.95816022	-2.26788268
IMAGE:813318	NRM Hs.519993	2.447958699	2.11322424	1.56170938	-3.93869503	-2.65953121	-2.19568489
IMAGE:2119355	CRISP3 Hs.4044	-1.612583072	-1.55760262	-1.08550377	2.79804068	1.44304736	1.87107021
IMAGE:329018	GLTP Hs.381256	2.210448551	0.95727761	1.69792415	-2.82848365	-2.31203527	-1.73347551
IMAGE:49944	ACSL3 Hs.4714	-2.311069547	-0.99478278	-1.87560626	1.59801761	2.94987133	2.75539738
IMAGE:811827	NDST1 Hs.2220	1.286726183	2.36288107	0.808571	-2.55884605	-1.97185248	-1.9544261
IMAGE:2566009	TIMP2 Hs.1048	1.892475659	0.35628057	2.72959067	-2.18556571	-2.71989444	-1.87966252
IMAGE:85097	PROS1 Hs.6401	2.625571134	1.27807279	1.86794706	-2.62030525	-2.85620339	-2.71389107
IMAGE:296140	FAM89A Hs.385	3.01948362	0.92694461	0.6608797	-2.32240867	-2.50073213	-1.92260116
IMAGE:814350	IDE Hs.500546	2.854937796	0.52129052	1.5611925	-2.66067832	-2.35007677	-2.00507802
IMAGE:144849	Hs.569690 Tra	1.168311645	2.45070386	0.66476681	-2.27091134	-2.09311178	-1.89545866
IMAGE:2209734	PTGES Hs.1466	1.988474049	1.48174516	1.46766936	-3.54965042	-1.80425092	-1.68601418
IMAGE:487988	CORO1A Hs.415	1.257680564	2.05792439	0.99959274	-2.37797544	-1.90371149	-1.94121143
IMAGE:435858	RNASE6 Hs.232	1.46456101	1.94503039	1.1297626	-2.38791595	-2.26856368	-1.87011073
IMAGE:341763	CASP5 Hs.2133	1.690994638	2.22395115	1.0944745	-2.5792583	-2.63793241	-2.0233211
IMAGE:489129	FBN1 Hs.59113	1.748628064	1.44883937	1.47243976	-2.23266143	-2.28050005	-2.12358937
IMAGE:206816	CECR1 Hs.1703	1.269267068	1.96114296	1.89290314	-2.54481723	-2.50453679	-2.16238994
IMAGE:2510979	PSMB8 Hs.1800	1.611631904	1.89326931	1.1666448	-3.18767067	-1.95651286	-1.57147429
IMAGE:742685	DAB2 Hs.48198	1.842109422	1.63342854	1.42899777	-2.6382961	-2.34574618	-2.01551187
IMAGE:205185	THBD Hs.2030 T	1.718000192	1.48756613	1.34066441	-3.30240201	-1.73076967	-1.45100831
IMAGE:248829	LOX Hs.102267	1.088160764	2.06893959	1.98431121	-1.72976785	-2.7229616	-2.76331009
IMAGE:753184	SOX9 Hs.64267	-2.149140682	-1.03846456	-1.31201387	1.77826941	2.15398774	2.48916805
IMAGE:757440	IL10RA Hs.5040	1.225063485	2.18387073	1.11658529	-2.51705504	-1.85379255	-2.13828535
IMAGE:281392	DNER Hs.23407	-2.046921213	-1.17788851	-1.07566833	2.07292262	1.71026903	2.39860835
IMAGE:1609538	KIAA1324 Hs.64	-2.321842266	-0.94506443	-0.77521451	1.88927642	1.77927613	2.20082563
IMAGE:50503	ITGB2 Hs.37595	1.085216306	2.3530526	0.84951866	-2.43997549	-2.01167377	-1.76765243
IMAGE:510383	IFITM1 Hs.4584	1.461545936	1.97289848	1.18777565	-2.88042717	-2.24320323	-1.51275579
IMAGE:2306445	TGM1 Hs.50895	2.393665687	0.67532093	1.17153066	-2.32077897	-2.06754922	-1.67956506
IMAGE:85634	C1S Hs.458355	1.859880731	1.35177777	1.59482035	-2.95587684	-2.03557534	-1.81956101
IMAGE:1032509	Hs.572666 Tra	-1.943846645	-1.55728405	-0.57294426	1.67847565	2.23474055	2.05466017
IMAGE:1606893	LOC642851 Hs.	-1.809126514	-1.62521097	-1.50198755	2.53197011	2.18712846	2.30989209
IMAGE:878449	Hs.592532 3'U	1.885088846	1.456373	1.56224883	-3.44605919	-1.87221409	-1.64673052
IMAGE:133273	PMP22 Hs.3720	1.866069157	1.30913919	1.61984749	-2.5049936	-2.4861501	-1.79647818

IMAGE:783698	LPIN1 Hs.46774	-1.931630279	-1.15401904	-1.10621933	1.44028431	2.60150555	1.96945828
IMAGE:785701	RAB31 Hs.99528	2.376466481	0.96199275	0.82238236	-2.33390453	-2.16500452	-1.53675775
IMAGE:503051	LOC645638 Hs.4	1.744663944	0.95091084	1.8431674	-2.26021085	-2.15529881	-1.93181177
IMAGE:2125838	HLA-G Hs.51215	1.750325216	1.72119732	0.61258972	-2.63589854	-2.20771851	-1.12940392
IMAGE:915446	TMPRSS2 Hs.43	-2.109597094	-1.30863384	-0.75152892	2.05644694	1.73145717	2.27885344
IMAGE:740975	SLC4A11 Hs.105	-1.712242639	-1.9199901	-1.11606433	1.65659922	2.6519368	2.5348935
IMAGE:214136	SORBS2 Hs.481	-1.950704411	-1.71802338	-1.0993656	2.08807117	2.44286085	2.34636666
IMAGE:121530	URP2 Hs.180535	1.186514258	2.30203334	1.03616169	-2.47469211	-2.06243506	-1.99089633
IMAGE:1323785	THY1 Hs.643513	1.149253728	2.26830765	0.7235496	-2.29295854	-1.88200654	-1.85581399
IMAGE:1534435	TIMP2 Hs.10483	1.626124578	0.17137876	2.68737358	-2.12342314	-2.29632326	-1.63572557
IMAGE:75644	C4A Hs.534847	1.944383941	0.05663733	2.32336337	-1.89671563	-2.32038603	-1.68863447
IMAGE:322537	SLC26A2 Hs.302	-2.184763228	-1.15565919	-1.13039599	2.21394445	2.10459441	2.10508976
IMAGE:915177	AA579371 221	-1.53431836	-2.18896043	-0.89267627	1.60262431	2.38262338	2.71551584
IMAGE:509641	IFITM1 Hs.4584	1.419511491	1.80739793	1.10712438	-2.87788925	-2.04670403	-1.29967632
IMAGE:843121	CLIC1 Hs.41456	1.869518157	1.78789933	1.04468789	-2.46036066	-2.53957893	-1.79204649
IMAGE:753215	GNAI1 Hs.13458	2.213954167	1.57408647	1.21576056	-2.47264439	-2.35072365	-2.37839361
IMAGE:213502	CD53 Hs.443057	1.342887913	1.94715155	0.61351263	-1.92446334	-1.74929786	-2.02818877
IMAGE:2441080	ANPEP Hs.1239	1.495108728	1.78607088	1.06311551	-2.2543665	-2.35705902	-1.63923828
IMAGE:24707	MARCH3 Hs.132	3.159905211	0.19497352	1.30129132	-2.15895465	-2.27274734	-2.22723026
IMAGE:84786	FOXA1 Hs.16348	-1.607315565	-1.24315492	-1.23009224	2.02710386	1.55499615	2.23122102
IMAGE:1323448	CRIP1 Hs.70327	2.171239126	0.7218201	1.23897782	-1.79775852	-2.40455273	-1.68599986
IMAGE:824384	CD37 Hs.166556	1.298645697	2.01190343	1.01405945	-2.28323429	-1.95617515	-1.99398856
IMAGE:2511237	FBLN2 Hs.19886	1.8392999	0.49819192	1.99345024	-2.184243	-2.21117552	-1.60263201
IMAGE:260303	ETS2 Hs.517296	2.01686614	1.43778416	1.0894158	-2.90693923	-2.0038471	-1.63295888
IMAGE:1592837	IFITM2 Hs.1741	1.249434566	2.01400719	0.73535616	-2.59231975	-1.86493451	-1.35710359
IMAGE:796240	MICAL1 Hs.3347	1.52543564	1.51659554	1.45029478	-3.30280654	-1.70495828	-1.36815043
IMAGE:260052	HCLS1 Hs.14601	1.150617399	2.14439806	0.73560459	-2.47402569	-1.48493267	-1.90307057
IMAGE:122394	NCLN Hs.73797	1.968322328	1.24387742	1.05724076	-3.20040793	-1.62259147	-1.31685116
IMAGE:183337	HLA-DMB Hs.35	1.325013613	1.68887279	1.61236906	-3.13389398	-1.83449689	-1.56790007
IMAGE:624360	PSMB8 Hs.18000	1.429707237	1.78920787	0.97478775	-2.70572593	-1.83889264	-1.50223878
IMAGE:815130	Hs.598906 Tran	-1.968001469	-1.66429735	-1.19542081	2.32195242	2.3661316	2.25464022
IMAGE:131268	GRB14 Hs.41188	-1.92785105	-1.11570932	-1.05401	2.19138123	1.79406971	1.89740211
IMAGE:1257131	MFI2 Hs.184727	-2.118488916	-1.06054678	-0.61563075	1.90498433	1.73226849	1.90083915
IMAGE:1475574	Hs.173894 Tran	1.741412346	1.70418826	0.96880568	-2.12513662	-1.63157276	-2.62269862
IMAGE:2569190	FSTL1 Hs.59131	1.873721887	1.20257405	1.25723927	-2.31204696	-2.1797924	-1.69415363
IMAGE:343760	SH3BGRL2 Hs.3	-2.166749708	-1.68985404	-0.17047974	2.31417493	1.44947398	2.23435638

IMAGE:362278	SOCS3 Hs.5279	1.770551362	1.75753932	0.5135885	-1.88188701	-2.03091551	-2.02131913
IMAGE:1926620	THY1 Hs.643513	1.31248181	2.07074438	0.33979118	-1.63718436	-1.98657985	-1.87581405
IMAGE:122982	EGLN3 Hs.1355(	1.833184084	1.08744564	1.1029067	-1.65805204	-2.09482698	-2.00669894
IMAGE:51843	PIAS2 Hs.51484	-2.803648025	-1.57576212	-1.43687011	3.94476402	2.54050731	1.87993153
IMAGE:1913366	PRSS3 Hs.1280:	2.164772189	0.75061319	0.83171559	-2.30317463	-2.06833697	-1.04121096
IMAGE:741831	PLTP Hs.439312	1.752453793	1.13863551	1.47324133	-2.58301214	-2.06650651	-1.52866695
IMAGE:2491247	SLCO2B1 Hs.78	1.547079737	1.83658188	0.90671604	-2.13316924	-2.07677272	-1.99894552
IMAGE:486676	LCP1 Hs.381099	0.922136369	2.05038501	0.90278041	-2.24999114	-1.64341954	-1.69384691
IMAGE:80256	TIMP2 Hs.10483	1.721683911	0.5332225	2.52557715	-1.7335434	-2.7426144	-2.06317326
IMAGE:1892897	MASP1 Hs.8998	1.594075982	1.09926087	1.40119298	-1.64960653	-2.05223691	-2.08965307
IMAGE:325072	TIMP2 Hs.10483	1.203935776	1.22946259	2.00274045	-2.26240039	-2.09236156	-1.79876116
IMAGE:365085	TRPS1 Hs.2535	-1.758948966	-1.96401539	-0.66510277	1.81924605	2.21891148	2.37766746
IMAGE:2541441	SERPING1 Hs.3	1.836668917	0.0869976	2.56206457	-2.59521131	-2.14050949	-1.35235968
IMAGE:190325	PLCB4 Hs.4721(	-1.729954408	-1.72224451	-0.74154576	2.09617778	1.69574507	2.31330773
IMAGE:301735	TGM3 Hs.2022 T	2.068142124	0.8398196	1.3806178	-2.46092108	-1.83370668	-1.79308708
IMAGE:450079	AA703419 244	-1.686972052	-1.30151485	-1.05068002	1.66895066	2.20398826	1.92314146
IMAGE:159118	RHOG Hs.50172	1.508857285	2.09921174	0.89741874	-2.88901984	-2.01930123	-1.62555589
IMAGE:431655	CD37 Hs.16655(	1.398824914	1.799017	0.86437484	-2.23146595	-1.99909635	-1.64666913
IMAGE:840493	RNASE1 Hs.782	1.237636684	1.10840674	2.0022472	-1.96565062	-2.15411096	-1.90211256
IMAGE:47481	CLEC2B Hs.852(	1.702110613	1.26641725	1.39427493	-2.42618581	-2.28570524	-1.48374441
IMAGE:2326216	PADI2 Hs.33455	-1.572329936	-1.75970009	-0.39450032	1.99329809	1.72395349	1.77391887
IMAGE:625384	Hs.269512 Tran	1.788819496	1.21507181	1.08301369	-2.28783408	-1.95608612	-1.61568387
IMAGE:1555479	Hs.380686 **Tı	-1.75256585	-1.31436229	-0.95239414	2.18421292	1.88707489	1.71959709
IMAGE:856447	IFI30 Hs.14623	1.259532108	1.78882791	0.57667777	-2.29564108	-1.4326588	-1.56508735
IMAGE:381066	SERPING1 Hs.3	1.574393418	1.11669495	1.9357845	-2.14577738	-2.02767767	-2.28290813
IMAGE:755599	IFITM1 Hs.4584	1.217767845	1.72937813	1.07361602	-2.44828989	-2.07664845	-1.23780064
IMAGE:588053	TMEM45B Hs.50	2.01334744	1.33925746	0.318683	-1.71483104	-2.0009306	-1.71149946
IMAGE:796147	PLEKHH1 Hs.59	-1.818835788	-1.68150881	-0.41387512	1.62543059	2.13248406	2.00994614
IMAGE:81449	MOSC2 Hs.3690	-2.119027885	-1.4685421	-0.78903465	2.22663411	1.71708691	2.42392728
IMAGE:47142	PEX12 Hs.59119	2.006395543	1.0795533	1.65251032	-2.33206873	-2.3910496	-1.97144284
IMAGE:565050	DAZ4 Hs.70936	-1.33483134	-1.31945641	-1.00236757	1.68862527	1.75484401	1.79092181
IMAGE:50302	ASPHD2 Hs.567	2.393193138	0.21254563	1.59569919	-1.14149755	-2.21024636	-2.55148823
IMAGE:344292	HAL Hs.190783	1.82562605	0.53428211	1.41120539	-2.04047798	-1.68973729	-1.57365371
IMAGE:785707	PRC1 Hs.567385	1.622348208	1.15964449	0.92373751	-2.60515753	-1.59464137	-1.12786202
IMAGE:504226	CD53 Hs.44305	1.342358191	1.73868883	0.48350971	-1.66945738	-1.69918218	-1.8573181
IMAGE:429681	SORBS2 Hs.481	-1.559784215	-1.79735194	-0.86829246	1.60241182	2.35223494	2.16642304

IMAGE:714453	IL4R Hs.513457	1.355927194	2.09401564	0.53624574	-3.1924139	-1.20182594	-1.45098159
IMAGE:564801	SLC12A2 Hs.162	-1.851275229	-0.96778233	-0.86016796	1.79344069	1.69314128	1.81721432
IMAGE:2504698	ARRB2 Hs.4358	1.659611642	1.75039154	0.70444927	-1.84518303	-2.10716197	-2.04322135
IMAGE:307050	PPARGC1A Hs.5	-2.005283895	-1.1167292	-0.42970238	1.60529017	1.705577	1.90928045
IMAGE:430169	Hs.38218 Trans	1.166013372	2.01667614	0.85325906	-1.86325102	-2.183607	-1.79375007
IMAGE:486386	STK10 Hs.51975	1.697398256	1.4451229	1.25480991	-2.01746703	-2.18343766	-2.08138943
IMAGE:361587	ZMAT1 Hs.4965	-1.88548529	-1.02439863	-1.4256735	2.0784119	2.10801617	1.96048969
IMAGE:1516938	GPR109A Hs.524	1.62795069	1.21354947	1.33964078	-2.11423397	-2.04622967	-1.77633759
IMAGE:1911490	Hs.536075 PP6	-1.472891931	-1.7812929	-0.64849966	1.44912436	2.19774284	2.04503462
IMAGE:999166	AA550710::AI	-1.901526486	-1.32386439	-0.9056259	2.45360047	1.83339891	1.6831193
IMAGE:1635983	HAO2 Hs.35636	-1.641838034	-1.34345734	-0.47139005	1.07832467	2.32891273	1.65994323
IMAGE:611412	RPRC1 Hs.64356	1.978430586	1.01541862	1.18110714	-2.5689624	-1.98232411	-1.41587121
IMAGE:295564	RAB31 Hs.99528	1.47357658	1.09917456	1.05828592	-2.72699237	-1.65551291	-0.79947883
IMAGE:119133	MAPK8 Hs.1382	-2.026425747	-1.11809995	-0.67118601	1.87745262	1.84908714	1.8292313
IMAGE:342647	MAPKAPK3 Hs.2	1.788342796	1.05226949	1.67347075	-3.1988327	-1.66813479	-1.48578936
IMAGE:1455976	IFITM2 Hs.1741	1.388008704	1.55896752	0.92378978	-2.75560552	-1.49807194	-1.32152409
IMAGE:70692	SERPINB2 Hs.51	2.108632409	0.65597549	0.91853449	-0.99666015	-2.53971079	-1.75870903
IMAGE:897774	APRT Hs.28914	1.609126721	1.41403905	1.18525442	-2.87205182	-1.82419457	-1.3200703
IMAGE:208699	KIAA1949 Hs.10	1.410092086	1.85570204	0.79849243	-2.37301705	-1.75234605	-1.77144362
IMAGE:713696	TIMP1 Hs.52263	1.015370902	1.85129226	0.60977845	-2.3301271	-1.47927858	-1.25281213
IMAGE:768571	SOX8 Hs.24367	-2.102001045	-1.29222826	-0.83448844	1.68138347	1.8850591	2.56801193
IMAGE:1669672	THY1 Hs.643513	1.097045691	1.76891684	0.84840432	-1.93336293	-1.63911279	-1.78697349
IMAGE:162310	PMP22 Hs.3720	1.542594777	1.14996779	1.2779443	-1.9793998	-2.20770743	-1.44916699
IMAGE:278501	FYN Hs.390567	1.486684154	1.66135059	1.33975886	-2.51703011	-2.03926604	-1.84045454
IMAGE:756556	SERPING1 Hs.3	1.677385346	0.68911227	2.1075748	-2.39910898	-2.37532701	-1.40977893
IMAGE:415899	IDE Hs.500546	2.156166909	0.48993063	0.9940449	-1.94676312	-1.69640334	-1.56853597
IMAGE:665405	MYO5C Hs.4870	-1.940338396	-1.15884059	-1.05323278	2.08254478	1.67334971	2.20941496
IMAGE:823964	ITGA4 Hs.44095	0.592714966	2.64578427	-0.05537832	-1.38025478	-1.57925989	-1.82901129
IMAGE:2503571	KLF4 Hs.376206	2.219289743	0.61166932	1.23518403	-2.06097907	-2.14483792	-1.58460164
IMAGE:293925	LYZ Hs.524579	1.270347238	1.69472949	0.50522909	-2.03829673	-1.5524098	-1.48844494
IMAGE:811024	BST2 Hs.11811	1.506719358	1.65031438	0.83071817	-1.67943332	-2.02185223	-2.07266277
IMAGE:325247	LOC645638 Hs.	1.660101652	0.6432325	1.59329571	-1.80688691	-1.84676672	-1.79296724
IMAGE:796839	PDGFRL Hs.458	1.417074793	0.69776641	2.05452547	-1.7918124	-2.10235196	-1.84625427
IMAGE:290158	LOC257407 Hs.	-2.05178784	-1.26687351	-0.81728944	2.45723149	1.60294048	1.93943186
IMAGE:1604703	HLA-F Hs.51997	1.164855374	1.66126253	0.89272856	-2.52036966	-1.78921575	-1.04550214
IMAGE:450926	PLEKHQ1 Hs.45	1.507185009	1.59856444	1.10827863	-2.40082347	-1.86704456	-1.77610445

IMAGE:380884	TMEM56 Hs.483	-1.926887934	-0.94819727	-0.61809258	1.38031938	2.09515876	1.60976538
IMAGE:323704	PLA2R1 Hs.4104	-1.493765253	-1.70262671	-0.59997083	1.56671815	1.92476636	2.05306697
IMAGE:713145	CD44 Hs.50232	1.561505868	1.96152166	0.46687442	-2.47324166	-1.98616361	-1.40872905
IMAGE:472186	RAB32 Hs.2877	1.268078855	1.93811644	0.95601939	-1.70890284	-2.56167795	-1.7337364
IMAGE:79240	HUNK Hs.10943	0.999947881	1.43325322	3.064742	-2.74178498	-2.24513229	-2.49381187
IMAGE:995732	AA531182::AI	-1.853906991	-1.05368631	-0.7676703	2.15940831	1.63495011	1.5266194
IMAGE:43642	SEPT11 Hs.1281	-2.154518908	-0.56277943	-1.38262115	2.57167683	1.89027329	1.34227383
IMAGE:878468	DPH1 Hs.51385	1.845834023	1.0696316	1.0766087	-2.8695957	-1.68566897	-1.16369464
IMAGE:2417989	ZAP70 Hs.2345	0.467383872	2.83762963	-0.00841454	-1.62013792	-1.37086853	-1.95599562
IMAGE:81662	LOC340061 Hs.	1.279823406	1.82554051	0.66471555	-2.4044139	-1.68463186	-1.39989455
IMAGE:346510	NCF1 Hs.52094	1.42640605	1.63705235	0.89076259	-2.84605986	-1.7047008	-1.15788019
IMAGE:1521297	PLCB4 Hs.47210	-1.853826927	-1.1761537	-0.77439029	1.81050663	1.48384336	2.21860882
IMAGE:814123	NCF4 Hs.47478	1.115622451	1.75202596	1.29847642	-2.487004	-1.52063425	-1.91692988
IMAGE:279195	Hs.596939 Tran	-1.511048501	-1.22430582	-1.01885329	1.80361339	2.04163766	1.53134704
IMAGE:433170	SMPD2 Hs.5523	1.708365994	1.53987613	0.81170688	-2.68710032	-1.82529035	-1.37460612
IMAGE:1695257	APBB1IP Hs.310	0.811255915	2.03134231	0.90474913	-1.72195693	-1.75529966	-1.91757716
IMAGE:626502	ARPC1B Hs.489	1.020450251	2.17379047	0.35287825	-2.33483562	-1.59109286	-1.30653041
IMAGE:727988	CD52 Hs.27677	1.179839761	1.69784615	0.62607719	-1.80981345	-1.74320035	-1.54611153
IMAGE:1030498	MMP2 Hs.51361	1.188510182	0.51203057	2.13812241	-1.91075101	-1.91551495	-1.39719818
IMAGE:2450237	SOCS3 Hs.5279	1.477272094	1.6906938	0.18153251	-1.52252939	-1.76012458	-1.6962105
IMAGE:2417330	CD52 Hs.27677	1.204745784	1.57276855	0.78024657	-1.91499939	-1.62683062	-1.5997497
IMAGE:489674	ZNFX1 Hs.3717	1.581405964	1.52845118	0.99890329	-2.77701388	-1.75883771	-1.37756323
IMAGE:280982	TNIP2 Hs.36855	1.341577323	1.92443604	0.73503517	-2.49475568	-1.79578659	-1.52727173
IMAGE:47783	MYST2 Hs.2190	2.000664659	0.88056554	1.24401679	-2.1351038	-1.96778609	-1.77397641
IMAGE:2011862	CLEC10A Hs.544	1.072622792	1.6469046	0.79026757	-1.66250307	-1.71471051	-1.68991197
IMAGE:35612	COX7B Hs.5226	-1.90446009	-1.83741533	-0.55625085	2.42769823	1.94809961	1.93232885
IMAGE:149547	GNB4 Hs.27054	1.396261936	2.02236726	0.55643345	-1.93127599	-2.12193212	-1.77027748
IMAGE:1898511	HIRIP3 Hs.5920	1.943044786	1.08145	1.63533236	-2.84598622	-2.34338926	-1.39153217
IMAGE:502287	EMB Hs.561411	1.878963755	1.0223702	1.03365653	-1.71213251	-1.99307457	-1.93886451
IMAGE:2569196	MAP4 Hs.51794	1.494474994	1.36819996	1.22072319	-2.77946728	-1.92360315	-1.11684598
IMAGE:782503	FADS1 Hs.5035	-1.990464151	-0.61655947	-0.42907872	1.13824598	1.8316385	1.47699935
IMAGE:358885	FOXC1 Hs.3488	-1.817569783	-1.26769494	-0.52938344	1.4709069	1.77960857	2.03911091
IMAGE:1915749	GMFG Hs.5210	1.007947894	1.87033977	0.62784665	-1.88836415	-1.69777284	-1.51610282
IMAGE:955277	LOC653198 Hs.	-1.695719798	-1.6463161	-0.64144493	2.70230306	1.4322752	1.68028175
IMAGE:2028528	AI261207::AI	-2.14537129	-1.22383389	-1.06477401	2.5907629	1.79972403	1.99428835
IMAGE:417300	CFHR1 Hs.5758	1.856624274	0.34359847	1.6885103	-2.04542124	-1.85329098	-1.51225977

IMAGE:590759	SC4MOL Hs.105	-1.730544884	-0.90186813	-0.66945466	1.25750955	2.00514619	1.52278211
IMAGE:966761	CALML5 Hs.180	1.888208091	0.28033895	1.23712034	-1.83457965	-1.88486692	-1.07977442
IMAGE:429433	ATAD4 Hs.3682	-1.842371778	-1.13177939	-0.881713	1.98686693	1.55002735	2.02647372
IMAGE:838240	FNBP1L Hs.134	-1.880858794	-1.25515616	-0.96444509	1.77541047	2.10751418	2.02665415
IMAGE:767172	SVEP1 Hs.5223	1.377972655	0.45391462	2.14584035	-2.0038831	-1.7738421	-1.65240615
IMAGE:1609746	VMD2 Hs.59196	1.152712411	1.62097329	0.95765753	-2.17307013	-1.54333998	-1.64119036
IMAGE:1553568	HAO2 Hs.35636	-1.55741609	-1.18213153	-0.66690308	1.43408167	2.00775138	1.50111723
IMAGE:594871	RP11-125A7.3 H	-1.788160459	-1.02419447	-0.90671348	1.41421521	2.12725013	1.8104589
IMAGE:769565	RER1 Hs.52552	1.166761705	1.47125171	1.14138754	-2.60438327	-1.60935343	-1.17001785
IMAGE:127881	R08912::R090	-1.445583086	-1.55705266	-0.74224602	1.39203677	2.14715325	1.89257113
IMAGE:2298175	EGF Hs.419815	-1.678813355	-1.08539856	-1.36860849	2.41281244	1.65091615	1.79334991
IMAGE:128150	BIN2 Hs.14770	1.081891914	1.97899014	0.46227452	-1.42132868	-1.71429995	-2.0335376
IMAGE:789012	FBLN2 Hs.1988	1.613081841	-0.09192042	2.0348816	-1.87686607	-1.72793515	-1.22054291
IMAGE:181541	DNER Hs.23407	-1.793893842	-0.91636616	-0.6680445	1.80875403	1.29764083	1.79405077
IMAGE:207794	NFE2 Hs.75643	1.28249245	1.21079138	1.08609386	-0.68102581	-2.11660596	-2.29991131
IMAGE:713566	TMPRSS13 Hs.2	1.028345553	1.85783931	0.55615258	-1.66635597	-1.66225918	-1.69585286
IMAGE:1593317	PDGFRB Hs.509	1.23775455	0.32398262	2.3501856	-2.34928792	-1.79097177	-1.14007807
IMAGE:564962	DAZ1 Hs.52286	-1.191018529	-1.1656066	-1.07702786	1.72981099	1.29417784	1.85723368
IMAGE:449126	GPR153 Hs.531	1.303721167	1.12909645	1.56482669	-2.48958499	-1.54162225	-1.57405256
IMAGE:72003	Hs.135087 Tran	1.455075046	1.64738704	0.6809604	-1.90783378	-2.02950055	-1.56755929
IMAGE:203732	FGL2 Hs.52098	1.118875654	1.42045017	0.88328321	-1.58588344	-1.92591377	-1.40129555
IMAGE:827132	RAC2 Hs.51760	0.961413622	1.78895087	0.50666668	-1.68316979	-1.50229292	-1.57341738
IMAGE:810928	PLOD3 Hs.1533	1.294041227	1.58849778	1.25168081	-2.67001548	-1.9183764	-1.30001764
IMAGE:1009876	GTF2H5 Hs.356	-1.862571731	-1.16503891	-0.61568664	2.01837317	1.52895571	1.76369538
IMAGE:488202	ACOT4 Hs.4943	-2.054360095	-1.03476944	-0.51901745	1.51425067	2.15828848	1.60992697
IMAGE:2020898	PLOD3 Hs.1533	1.295210329	1.67170339	1.25756102	-2.36403334	-2.0493927	-1.60889582
IMAGE:757222	HMGCS2 Hs.598	-1.409697574	-1.50703581	-0.28678715	1.51283111	1.53147035	1.68928255
IMAGE:487118	AADAC Hs.5069	2.54157883	-0.08299853	0.46569142	-1.27294771	-1.46520295	-1.53183406
IMAGE:1762435	SLC13A1 Hs.48	-2.043585134	-1.68568819	-0.70298809	2.47377076	1.89946551	2.09940882
IMAGE:158123	ATP6V1B1 Hs.64	-2.032918896	-1.26239668	-0.31278011	1.83617362	1.55584039	1.94193449
IMAGE:50519	ARNTL Hs.6573	1.18948061	1.33071076	1.40204583	-2.07263121	-1.92143188	-1.53878125
IMAGE:345626	FABP7 Hs.2677	-1.763271609	-0.75214182	-0.52974566	1.3443153	1.64883302	1.4421539
IMAGE:45544	TAGLN2 Hs.517	1.700931403	1.33334113	0.57942069	-2.01587475	-1.90235422	-1.3574557
IMAGE:138139	LOC643790 Hs.	1.25688042	0.91761864	1.68359635	-2.27841585	-1.83172117	-1.25610701
IMAGE:795198	SLC39A1 Hs.78	1.501413964	1.60132258	0.65552106	-2.31114024	-1.63675712	-1.52560879
IMAGE:1551030	CDA Hs.466910	-1.527881325	-1.57985143	-0.10794251	1.09126771	1.61443346	2.0908261

IMAGE:995700	AA531163::AI	-1.785143378	-1.34552344	-0.5057125	2.07029785	1.5864746	1.67136841
IMAGE:1650748	FAM84B Hs.1249	-1.714399371	-1.40665107	-0.67817289	1.6862194	1.82929074	2.01378163
IMAGE:2543381	CD99 Hs.49560!	1.55849613	0.95711556	1.21665875	-2.17586622	-1.90155228	-1.21682247
IMAGE:455227	PLCL2 Hs.20201	0.861457228	2.1837566	1.09372199	-2.02374252	-2.25658219	-1.65464852
IMAGE:235903	HLA-DRB1 Hs.53	0.995370737	1.32917044	1.20393166	-2.20933648	-1.53074672	-1.25164314
IMAGE:2012021	OAS3 Hs.528634	1.771225854	1.43860474	0.33012199	-1.93280779	-1.90992102	-1.38466956
IMAGE:1609625	SELPLG Hs.5910	0.994492033	1.6397617	0.8207261	-1.79230807	-1.48815351	-1.69682664
IMAGE:309864	JUNB Hs.25292	1.633468468	1.26400333	0.71152407	-2.89398307	-0.98006775	-1.36156198
IMAGE:2578744	TUBB Hs.533059	1.087621721	1.92053569	0.95862163	-2.42610711	-1.96140206	-1.32300398
IMAGE:898122	C7 Hs.78065 Co	1.055055331	0.68684095	2.27491868	-2.48269546	-1.71782938	-1.25596793
IMAGE:1867758	Hs.634776 Tran	-1.331370145	-1.32648873	-1.4758601	1.76125189	2.08798144	1.98238011
IMAGE:1610546	FOXA1 Hs.16348	-1.749918266	-0.90878847	-0.99432709	1.56137688	1.45675543	2.21283666
IMAGE:48631	KCNAB2 Hs.440	0.948144457	1.64308828	1.24044204	-2.40801301	-1.80151659	-1.22787205
IMAGE:1010077	ACACA Hs.1605	-1.993657191	-1.00933699	-0.66147201	2.15490717	1.52850571	1.6479184
IMAGE:826218	PER3 Hs.533339	-1.824591917	-1.05510444	-1.19310695	1.78660629	1.99486301	2.02945892
IMAGE:2349964	PDGFRL Hs.4585	1.305185437	0.4535186	1.84528163	-1.4721274	-1.69034808	-1.78218262
IMAGE:2410729	RAC2 Hs.517601	0.945711058	1.80652616	0.52590213	-1.97240042	-1.42566651	-1.38766656
IMAGE:814282	Hs.36190 CDNA	-1.63813955	-0.77640113	-1.54322858	1.87955386	1.78641692	1.88487597
IMAGE:128515	SCARA3 Hs.128	1.54285267	0.83513387	1.78017792	-2.14925777	-2.19714852	-1.44579593
IMAGE:2028722	POSTN Hs.1363	0.953244549	1.4734709	1.05438527	-2.02068153	-1.51886797	-1.41850526
IMAGE:730410	LCK Hs.470627	0.781100758	2.00884032	0.35914472	-1.54690189	-1.47445763	-1.612483
IMAGE:454192	PCCB Hs.63788	-1.701661185	-1.42481003	-0.72103016	2.17712912	1.77412582	1.63973959
IMAGE:826166	THAP11 Hs.6322	1.666456468	0.96206161	1.35594771	-2.33637514	-1.90511298	-1.39622363
IMAGE:1567676	MYADM Hs.3809	1.796776267	0.54850254	1.40798273	-1.73277732	-1.95426356	-1.59085575
IMAGE:742143	CD5 Hs.58685 C	1.048404437	2.1057326	0.24372623	-1.52328891	-1.48542112	-2.0271533
IMAGE:324935	TAPBP Hs.37093	0.790344575	1.88454689	0.50470846	-2.96701887	-1.02421445	-0.65198946
IMAGE:213917	DOCK2 Hs.5861	0.793353782	1.93554372	0.58335604	-1.9257098	-1.39780688	-1.49902463
IMAGE:261194	Hs.643733 Tran	-1.637875005	-1.29576238	-0.38945386	1.8619661	1.34251544	1.68279187
IMAGE:277173	PPP1R1B Hs.286	-1.404760279	-1.28937644	-0.54043042	1.55252755	1.41406695	1.7501486
IMAGE:809707	JUNB Hs.25292	1.473070707	1.36420463	0.61127276	-2.51932946	-1.31829075	-1.18238374
IMAGE:725395	UBE2L6 Hs.4257	1.389381258	1.46158267	0.64605051	-2.22246584	-1.72164015	-1.13990304
IMAGE:429203	WDTC1 Hs.4691	1.241090609	1.3902573	0.86989504	-1.63739624	-1.90675686	-1.49023756
IMAGE:238886	SH3BGRL3 Hs.1	1.181706456	1.38863531	1.23033474	-3.16561007	-1.24117174	-0.98664926
IMAGE:745983	AA420833::AI	-1.863742159	-0.77803813	-0.43567501	1.0744305	1.88326891	1.54956478
IMAGE:983931	SEMG1 Hs.1968	-1.654387379	-1.101748	-0.21380586	1.75846589	1.39497911	1.24801538
IMAGE:725143	VTCN1 Hs.5464	-1.534483708	-1.24435598	-0.80117141	1.44353462	1.87182449	1.85436467

IMAGE:1535416	Hs.105902 Trar	1.571215453	-0.13266289	2.29530264	-1.625224	-1.62312864	-1.77860449
IMAGE:796475	FHL3 Hs.57687	1.442855042	1.68544307	1.02802951	-2.63171414	-1.94033826	-1.40543166
IMAGE:1604005	SH3KBP1 Hs.44	0.651736668	2.18641548	1.32249715	-2.77053093	-1.62745982	-1.51235891
IMAGE:2010931	ZNF414 Hs.5151	1.162579353	1.88273848	0.79860704	-2.19832546	-1.70678441	-1.66112568
IMAGE:1636885	MS4A4A Hs.325	0.995286592	1.60810217	0.62586735	-1.34537052	-1.69567442	-1.64637239
IMAGE:50926	SH3BGRL2 Hs.3	-1.442822686	-1.55456116	-0.39400904	1.9740588	1.30720929	1.70731899
IMAGE:67759	ALOX5AP Hs.50	1.00866085	1.4281975	0.85647599	-1.81467685	-1.44758228	-1.46362338
IMAGE:452466	NLGN2 Hs.2622	1.356164516	1.78359568	0.98104582	-2.95833844	-1.59980908	-1.37780006
1292541	Hs.583148 Trar	-1.208918305	-1.82666701	-0.34924884	1.30265207	1.46585038	2.22143658
IMAGE:323506	MAPK1 Hs.4318	1.152558833	1.71037995	0.95936731	-2.82858509	-1.58035028	-1.08468194
IMAGE:172785	CAMK1D Hs.156	1.803982064	0.55942605	1.55806979	-2.22882927	-1.79826859	-1.46560155
IMAGE:1671114	LOC202781 Hs.1	-1.181352669	-1.85809406	-0.94288189	2.17093416	1.82450702	1.74233128
IMAGE:130843	MAPK8IP2 Hs.55	-1.917509497	-1.33354487	-0.49038191	1.32836055	1.98916263	2.17203576
IMAGE:2017513	GARNL4 Hs.499	-1.417435757	-1.23581561	-1.47299015	1.72737809	1.97305176	2.1206849
IMAGE:1688899	TAX1BP3 Hs.129	1.727487201	1.7339289	1.53488026	-3.19735624	-2.19728175	-1.7160865
IMAGE:629907	Hs.567010 Trar	-1.479064158	-1.17852794	-0.70594162	1.96372992	1.2399615	1.66512375
IMAGE:76481	PTK9L Hs.43643	1.461821933	1.62892022	0.60962066	-2.20668177	-1.87944762	-1.31200967
IMAGE:878652	PCOLCE Hs.202	1.07046538	1.04680097	1.37389568	-1.55851886	-1.90954091	-1.42520936
IMAGE:897296	MDM4 Hs.49749	1.323236688	1.07638827	1.0942635	-3.02789235	-1.13184111	-0.80753335
IMAGE:588033	EPS8L3 Hs.4853	1.208146722	1.36509994	0.7529824	-2.28083915	-1.41339237	-1.10686648
IMAGE:321902	DSG2 Hs.41259	-1.831294088	-0.78109725	-0.68176578	1.50564905	1.63724202	1.62790317
IMAGE:810104	PLCB2 Hs.35588	1.046876348	1.48249993	1.01138109	-1.88745183	-1.42278667	-1.74805228
IMAGE:76103	ELAVL2 Hs.1661	1.299729974	0.46835958	1.50605252	-2.98663096	-1.33280783	-0.21526118
IMAGE:126858	SOAT1 Hs.49638	-1.766893435	-0.58171969	-0.777004	1.07516668	1.89334061	1.52566739
IMAGE:471196	ITM2C Hs.11157	1.089353443	1.80520791	0.99677678	-2.16886087	-2.28996407	-1.12898806
IMAGE:949938	CST3 Hs.304682	0.997818234	0.927635	1.69065276	-2.3524416	-1.52643888	-1.12261533
IMAGE:703943	CAB39L Hs.8715	2.295734256	0.24028279	0.97947332	-1.48220897	-2.08731609	-1.45884217
IMAGE:431231	CFL1 Hs.170622	1.318578788	1.11137854	1.26627188	-2.1537486	-2.01385802	-1.06016922
IMAGE:22778	R38615::T751	-1.413192171	-0.96914466	-0.89907887	1.48112178	1.83828437	1.37794768
IMAGE:1635286	ITGB4BP Hs.632	1.508801623	1.09074015	0.78350561	-2.96941986	-1.17864475	-0.73063005
IMAGE:1555384	FARP1 Hs.40391	-1.687475262	-0.98477966	-0.69926139	1.81228185	1.38523779	1.68493947
IMAGE:759948	S100B Hs.42218	1.261908052	1.18600636	1.4056748	-2.21550587	-1.61945431	-1.59400492
IMAGE:1700429	GFRA1 Hs.5919	1.20356557	1.17202445	1.26244232	-1.69243478	-1.96579114	-1.48321203
IMAGE:810504	PLP2 Hs.77422	1.482565159	1.21891721	0.83223355	-2.21344955	-1.53235901	-1.34670692
IMAGE:985356	STAC2 Hs.1450	-1.672569787	-1.1068881	-0.28705276	1.60962855	1.30318828	1.61518596
IMAGE:462412	AA699878 12	1.434300566	0.11589576	1.82026835	-1.93288621	-1.38832771	-1.279416

IMAGE:1941467	CDK6 Hs.11988	1.590214237	0.50751847	1.25116658	-1.55000648	-1.7982474	-1.3623034
IMAGE:430954	RHBDF2 Hs.464	1.536520749	1.36109985	0.89294532	-2.90025758	-1.07478383	-1.48757113
IMAGE:242698	EPB41L4B Hs.59	-1.161927859	-1.72725479	-0.68866846	1.60877981	1.6814647	1.90436503
IMAGE:853367	PRRX1 Hs.2834	1.280938631	1.2854465	1.23856469	-2.30664655	-1.75142772	-1.33970929
IMAGE:325383	NXPH3 Hs.5506	1.703592986	0.69354701	1.26006793	-1.89386515	-1.9812822	-1.29564755
IMAGE:85497	C2 Hs.408903 C	1.266097492	1.00151816	1.34411448	-1.45550799	-1.77436001	-1.85169858
IMAGE:811891	FA2H Hs.46132	-2.213452653	-0.62282712	0.1510972	0.89533096	1.60874847	1.56146873
IMAGE:82903	TAPBP Hs.37093	0.719776159	1.67353092	0.70906733	-2.74001107	-1.13368726	-0.60259645
IMAGE:512133	ADCY7 Hs.5135	1.581076716	1.31453175	0.74528296	-1.51080259	-1.99235767	-1.77185614
IMAGE:1188588	SPDEF Hs.4851	-1.720667467	-0.85566683	-0.73503749	1.66357398	1.28668084	1.83304349
IMAGE:785866	SFRP2 Hs.4810	1.478178617	0.68011205	1.51420246	-2.11806928	-1.62323633	-1.38888345
IMAGE:1635618	SAPS1 Hs.5156	1.447636129	1.14181663	1.5639081	-2.56647192	-1.79435338	-1.47823896
IMAGE:290111	HMGCS1 Hs.397	-1.392711798	-0.95060559	-0.80570382	1.20860609	1.92435073	1.38914903
IMAGE:284592	SLC43A3 Hs.99	1.270336162	1.31733609	0.93265976	-2.28484685	-1.40630974	-1.35617648
IMAGE:810331	AKT1 Hs.52562	1.661637516	1.30774823	0.61132906	-2.16276976	-1.69091737	-1.36455281
IMAGE:284341	SH3BGR Hs.473	-1.381491108	-1.6936436	-0.91265031	2.29792988	1.12276756	2.33281751
IMAGE:343821	TYRO3 Hs.3812	1.739893493	0.56805398	1.76963561	-1.6022285	-2.08405084	-1.98768637
IMAGE:23819	ABCG1 Hs.1246	1.991649746	0.75276391	0.93935452	-1.81048898	-1.86021491	-1.62010974
IMAGE:590323	VIM Hs.533317	1.442610113	1.10294101	0.87444333	-2.2149491	-1.76314688	-0.93328487
IMAGE:809910	IFITM3 Hs.3746	0.733809315	1.83652119	0.68521523	-1.97437628	-1.49450972	-1.24312879
IMAGE:489047	STS-1 Hs.44407	2.035050367	0.14970982	1.09439288	-0.71889729	-2.17390689	-1.75232719
IMAGE:137353	PEF1 Hs.470417	0.959074609	1.23259547	1.42699065	-3.02089807	-1.22591745	-0.8244279
IMAGE:124317	FOXP1 Hs.4314	-1.177604826	-1.79656285	-0.78231573	1.41352829	2.03483085	1.99078703
IMAGE:244764	Hs.642949 Tran	1.459994264	1.12685587	1.09888869	-1.94685993	-1.77843997	-1.52858617
IMAGE:2412851	VAV1 Hs.11623	0.75250525	1.80958368	0.74339811	-1.65064058	-1.22130411	-1.90043634
IMAGE:25988	EIF4G1 Hs.4337	1.788687572	1.27347164	0.31228481	-1.73477111	-1.98269302	-1.26613071
IMAGE:2057931	TACSTD1 Hs.54	-0.962441751	-1.33548368	-1.24480319	1.78977868	1.34314345	1.86997
IMAGE:136235	GSTP1 Hs.5238	1.448097286	1.03819451	0.81893802	-2.16208406	-1.56614848	-1.02487768
IMAGE:450912	SIRT2 Hs.46669	1.211597899	1.2704701	1.14856124	-2.02521171	-1.92460731	-1.20898454
IMAGE:810142	HLA-B Hs.77961	1.011206556	1.37029979	1.01048837	-2.52352772	-1.47318001	-0.83866226
IMAGE:1572980	HAO2 Hs.35636	-1.173650522	-1.26100213	-0.55851129	1.21955371	1.73676719	1.39379719
IMAGE:1509935	SERPINB7 Hs.1	1.425950343	0.193789	1.54044947	-1.17819793	-1.85671125	-1.32026166
IMAGE:46411	FARP1 Hs.40391	-1.346331048	-1.36734732	-0.76749308	1.75178785	1.43120067	1.84689538
IMAGE:739578	LYPD3 Hs.63159	1.761130624	0.51706419	1.17770636	-2.32863054	-1.85888821	-0.70190643
IMAGE:490556	TSPAN4 Hs.437	0.867352629	1.60384819	1.39722649	-2.24213052	-1.53420494	-1.67699889
IMAGE:845771	C17orf49 Hs.51	1.345810001	1.52952122	0.75823691	-2.02847053	-2.03494332	-1.19737913

IMAGE:795841	TMEM142A Hs.5	1.309493699	1.21682976	1.38035436	-2.69672267	-1.54864372	-1.26956174
IMAGE:245299	LOC388335 Hs.4	-1.082013793	-1.99976867	-0.12498381	1.26507418	1.90329454	1.61053475
IMAGE:356800	MTERFD2 Hs.15	-1.53887058	-1.06716201	-0.51038867	1.69976251	1.63608185	1.21119036
IMAGE:1154773	FCGRT Hs.1119(	1.017754061	0.84648713	1.75015083	-2.29467678	-1.66897088	-1.02040266
IMAGE:823779	PLEK Hs.468840	0.802930341	1.69277	0.55201753	-1.53160794	-1.21883662	-1.68312786
IMAGE:2450105	CCL21 Hs.57907	0.971896431	1.14573953	1.07925887	-1.51574733	-1.7911581	-1.2186221
IMAGE:703994	TA-NFKBH Hs.46	0.948471311	2.32866353	0.60982893	-2.17743293	-1.73718023	-1.76337527
IMAGE:814431	RAB30 Hs.40758	-1.397726387	-0.94451904	-1.16912327	1.47864068	1.8235448	1.67258676
*mitoch. cont. IMAC	147459	-1.73334731	-1.25971446	-0.2175829	1.5268835	1.69317738	1.5415104
IMAGE:1732444	MUC7 Hs.63194	-1.482596662	-0.97547187	-0.51991331	1.63126907	1.18510171	1.52062366
IMAGE:384795	CALML5 Hs.1801	1.756786421	0.21168419	1.03133671	-1.50350821	-1.69075045	-1.04761815
IMAGE:2333826	LY86 Hs.544738	1.171572248	1.59886059	0.63722444	-1.62485192	-1.55719793	-1.77012996
IMAGE:191603	TUBB Hs.533059	1.497673074	1.72007877	0.16986378	-2.18314112	-1.67208124	-1.18373512
IMAGE:51817	MFNG Hs.51760	0.928417975	1.72831364	0.78419887	-1.71318294	-1.85690644	-1.39525662
IMAGE:725549	SERTAD1 Hs.269	1.331290328	0.98265135	1.31477127	-2.54155835	-1.35952225	-1.21329602
IMAGE:811139	HLA-DRB1 Hs.53	0.827031822	0.88850227	1.72818852	-2.0093681	-1.42386213	-1.30030654
IMAGE:1056172	C15orf48 Hs.112	0.646145719	1.06775802	1.4489843	-2.2060615	-0.95453867	-1.22148582
IMAGE:839641	KIF5A Hs.15121	0.706530366	1.43005417	1.20398931	-2.04620168	-1.45779376	-1.205868
IMAGE:79565	FLJ22662 Hs.13	1.812214243	0.78182927	0.65883729	-1.89504546	-1.60555635	-1.21401006
IMAGE:491751	ISG20 Hs.45926	1.312262389	1.78088209	0.04588223	-1.75350671	-1.46015815	-1.48340464
IMAGE:346696	TEAD4 Hs.94865	1.277283728	1.23035848	1.07628724	-1.81724098	-1.99321067	-1.29637071
IMAGE:365642	SEC14L4 Hs.517	-1.27920473	-0.99032822	-0.65828252	0.72856816	1.93065148	1.56793294
IMAGE:810263	RHPN2 Hs.46643	-1.677394122	-0.77655101	-0.75512852	1.71628513	1.38258867	1.52595455
IMAGE:267738	C1orf27 Hs.3712	-1.503119349	-0.97218817	-1.50311935	1.57716638	2.62512339	1.38957069
IMAGE:344405	CTNNBIP1 Hs.46	1.836601265	0.11432654	1.2934502	-2.07967018	-1.22850388	-1.23503039
IMAGE:1634994	Hs.195313 **Tı	-1.80447669	-0.76122537	-0.97409933	1.67225336	1.9383458	1.45557809
IMAGE:2237353	GAL Hs.278959	-1.788112853	-0.53541895	-0.18532489	1.02797257	1.45407962	1.23490162
IMAGE:47510	AP3B2 Hs.19959	-1.522121418	-1.11743826	-1.06849983	1.3066609	2.19602272	1.79228069
IMAGE:2274786	HMOX1 Hs.5175	1.628202147	0.98177156	0.65618715	-2.09588108	-0.98408816	-1.65522526
IMAGE:1610464	EGLN3 Hs.1355(	1.639954053	0.81722097	0.87420404	-1.64249485	-1.82016678	-1.31585595
IMAGE:811849	ZNF655 Hs.521(	1.854940867	1.40426224	0.04832283	-2.59134094	-1.36421755	-0.99364971
IMAGE:84295	IL1RN Hs.81134	1.50586196	0.80032857	1.17790523	-2.01807378	-0.95323202	-1.96036154
IMAGE:151365	XLKD1 Hs.2467(	1.442612304	0.74399465	1.04695849	-1.00612467	-1.83106553	-1.75141835
IMAGE:1560977	HLX1 Hs.74870	1.391615868	1.52302677	0.97338638	-2.40094569	-1.72996447	-1.45778678
IMAGE:1589468	Hs.600894 Trar	1.965577725	0.41469422	1.18432016	-2.18957845	-1.31720648	-1.54402319
IMAGE:825343	FAM89A Hs.3853	2.300924443	0.27422847	0.95554329	-1.87832748	-1.6546363	-1.5241947

IMAGE:397488	TBX3 Hs.129895	-1.657047739	-1.07496509	-0.41332138	1.46245703	1.49987315	1.65234078
IMAGE:810010	PDGFRL Hs.4585	1.116121629	0.20263718	2.16744436	-1.36068544	-1.54011988	-1.78663834
IMAGE:785537	ELMOD1 Hs.495	1.970959909	-1.06151364	1.36329549	-0.81805553	-1.36373749	-0.88649576
IMAGE:1589138	DEFB1 Hs.3294	-1.350033451	-1.31476817	-0.83814437	1.16322803	1.7491742	2.13248066
IMAGE:272507	ME1 Hs.21160 N	-1.052554884	-1.35198249	-0.75167844	1.20265588	1.91672862	1.4270196
IMAGE:291490	CD59 Hs.27857	-1.222677315	-1.19139372	-1.1859531	1.22621607	2.01578679	1.86154507
IMAGE:1591677	C10orf26 Hs.50	1.849512536	1.04895795	0.58026963	-2.40725454	-1.47246555	-1.19332267
IMAGE:985787	RHOF Hs.52480	-1.531306631	-1.30297388	-0.28527961	1.36332197	1.65591407	1.58878425
IMAGE:838478	NCALD Hs.4924	-1.656472312	-0.76446864	-0.59779836	1.79338245	0.92092954	1.66434739
IMAGE:361899	ZNF187 Hs.1578	1.301279632	1.27729769	1.00965918	-2.65292342	-1.44303429	-1.03398225
IMAGE:768443	MGST1 Hs.3897	-1.79693782	-0.52508852	-0.43389933	1.04038313	1.66105244	1.3239781
IMAGE:307255	C1orf38 Hs.106	0.804416907	1.82118037	0.70937163	-1.74246504	-1.54353306	-1.53911235
IMAGE:985457	PIP Hs.99949 Pr	-1.466863148	-0.93755127	-0.52689051	1.76788963	1.20157328	1.29577185
IMAGE:724831	NCF1 Hs.52094	1.588758022	1.3502125	0.83733964	-2.20818184	-1.72838006	-1.51856843
IMAGE:770858	CD34 Hs.37499	1.144709177	0.16448151	1.99788075	-1.29210782	-1.38146177	-1.78756737
IMAGE:725365	GAS1 Hs.65029	1.395279191	0.89993221	1.69762608	-1.98879517	-2.31145786	-1.26459668
IMAGE:1552481	LCP2 Hs.304475	0.901474894	1.66758008	0.50596966	-1.60703148	-1.38444588	-1.49456718
IMAGE:323599	INHBB Hs.1735	-1.047164107	-1.73595628	-0.26614629	0.67807901	1.64873219	2.18055224
IMAGE:811161	HLA-E Hs.11835	1.226381554	1.23494268	0.83256968	-2.29063675	-1.6860071	-0.7560546
IMAGE:1129559	FBLN1 Hs.24601	0.945995513	-0.02398749	2.30107624	-1.65395705	-1.4530837	-1.15231659
IMAGE:1566642	AI091487 212	-1.457083579	-1.06159717	-0.9840837	2.54692519	1.68348313	0.77771743
IMAGE:824602	PYHIN1 Hs.3802	1.493116591	1.16338571	0.50441245	-1.99760378	-1.42515434	-1.19251089
IMAGE:2243051	KLK8 Hs.10457	1.749438876	0.09803914	1.15224084	-1.38294048	-1.37088343	-1.45769417
IMAGE:38816	TUBA1 Hs.7531	1.921434364	0.43066304	0.64574505	-2.11458459	-1.46723965	-0.75350319
IMAGE:725630	PEA15 Hs.51721	1.279088789	1.17605653	1.17213149	-2.61577582	-1.48978045	-1.04232608
IMAGE:842863	NDRG1 Hs.3729	1.445074616	1.16143113	0.55807089	-2.23091183	-1.4889757	-0.8874597
IMAGE:139062	FLJ25084 Hs.55	1.705594622	0.14162866	1.3962692	-1.29871116	-1.439156	-1.77830427
IMAGE:2568350	KRT17 Hs.2785	-1.633974439	-0.92767242	-0.56611416	1.30223574	1.27546435	1.9724129
IMAGE:1472405	S100A10 Hs.14	1.588640082	0.89020691	0.83075055	-2.12445751	-1.53049084	-1.10176032
IMAGE:267431	FYN Hs.390567	1.220462688	1.36244527	1.11539558	-2.02312939	-1.7320651	-1.51341192
IMAGE:1324976	CTSC Hs.12806	0.76048633	1.79414035	0.76202336	-1.24092146	-1.54640657	-1.9971412
IMAGE:755762	GRN Hs.514220	1.169142456	1.27554218	0.75933189	-2.33638534	-1.54868413	-0.73112235
IMAGE:80384	VAV1 Hs.11623	0.63818853	1.86028647	0.78984127	-1.75294156	-1.22798976	-1.75408276
IMAGE:486828	ERBB3 Hs.1186	2.342007112	0.43229903	0.3508556	-1.69435751	-1.60308469	-1.30258651
IMAGE:811108	TRIP6 Hs.53436	1.551225126	0.99410039	1.40178461	-2.82285952	-1.63323434	-1.11412517
IMAGE:431875	TRIM55 Hs.8552	-1.410341457	-1.11101696	-0.58187213	1.37274221	1.64289525	1.49374033

IMAGE:1555924	SCARA3 Hs.128	0.880283775	0.99822821	1.64776503	-2.39414847	-1.40215157	-1.08117422
IMAGE:1459964	TUBA3 Hs.5243	1.051193595	1.36083925	1.05893474	-2.44457678	-1.49628697	-1.00085393
IMAGE:50214	CD86 Hs.17118	1.456293399	1.31241787	0.77596768	-0.8389495	-1.87140324	-2.41267375
IMAGE:853938	DYNLL1 Hs.512	1.533440326	0.59608718	1.113636	-2.14324373	-1.44693845	-0.99615409
IMAGE:811955	SPRR2G Hs.490	1.062069504	1.01727328	1.00308781	-1.23252363	-1.63568963	-1.50466068
IMAGE:277414	MAF Hs.134859	1.319910244	1.13392769	0.91512469	-2.13663017	-1.50210147	-1.18593113
IMAGE:796876	NEK6 Hs.19707	1.921582909	0.40683846	0.94096322	-1.44945374	-2.13171467	-1.08766767
IMAGE:838899	TRIM2 Hs.4357	-1.645273347	-1.24112116	-0.52858328	1.69582267	1.85877005	1.43572814
IMAGE:745019	EHD1 Hs.52377	1.075385331	1.45848606	1.10272945	-2.38011684	-1.4392441	-1.35985796
IMAGE:1898064	RP11-125A7.3 H	-0.961466345	-1.2338057	-1.20596053	1.24050938	1.74987226	1.80997708
IMAGE:50768	ZDHHC18 Hs.52	1.367863547	1.40413919	1.23151716	-1.99107352	-2.08864249	-1.61768455
IMAGE:823719	LOC219854 Hs.	-1.437608211	-1.1701752	-0.10225013	1.67066811	1.32101751	1.04780217
IMAGE:377191	ZNF706 Hs.374	2.172249666	0.76126753	0.31445651	-2.2184098	-1.47457682	-1.10035982
IMAGE:859359	TP53I3 Hs.5064	1.483276527	1.2526433	0.81347919	-2.31736691	-1.42222168	-1.38114014
IMAGE:487425	CETN3 Hs.5917	1.249983798	1.1123819	1.0925864	-1.70185713	-1.68540826	-1.52201616
IMAGE:564549	BLMH Hs.37191	1.930369483	-0.39125303	1.20249345	-1.49683691	-1.29251044	-1.02244415
IMAGE:626348	TMEM139 Hs.17	-1.83980975	-0.89257288	-0.19975412	1.42436242	1.26800762	1.65589656
IMAGE:810603	C1orf198 Hs.56	1.868542135	0.49662092	1.16780699	-2.32030269	-1.73459786	-0.95260277
IMAGE:273536	Hs.633539 CDN	-1.261326457	-1.2669489	-0.57145312	1.28606034	1.96918675	1.25148234
IMAGE:283615	Hs.47068 Trans	-1.270205896	-1.86223581	-0.26891633	1.64299331	1.78612142	1.60569325
IMAGE:1609616	RASGEF1C Hs.1	-1.828918277	-0.63946328	-1.05855842	1.86826396	1.21529516	1.94221124
IMAGE:241530	EPHA2 Hs.1715	1.775750498	1.02136743	0.94040924	-2.67602961	-1.27766099	-1.41749785
IMAGE:1700163	ZNF117 Hs.2506	-1.279784451	-0.98162273	-0.95645678	1.43977873	1.76515797	1.38274504
IMAGE:32516	PPP1R14C Hs.48	1.788044551	0.15923424	1.2824186	-1.6175971	-1.82602306	-1.08032128
IMAGE:246748	BTK Hs.159494	0.914876698	1.7021713	0.68555191	-1.63212226	-1.08707216	-2.06331746
IMAGE:149910	SELL Hs.82848	0.692646542	1.874957	-0.02519854	-1.21514274	-1.31218672	-1.29257767
IMAGE:773149	SOSTDC1 Hs.54	-0.882426349	-1.75703531	-0.14435356	1.32858164	1.18177228	1.62928053
IMAGE:1434897	Hs.634838 Tran	0.895141498	1.13946708	1.37169734	-1.88730181	-1.63315598	-1.24607675
IMAGE:713530	LOC284402 Hs.	-1.225380906	-1.14169842	-0.63235103	2.34130608	0.98983395	1.00991774
IMAGE:120701	ACSBG1 Hs.250	-1.365112252	-1.10032643	-0.24176029	1.3748584	1.62126673	1.00423326
IMAGE:1635596	RASSF2 Hs.631	0.875335861	1.4131352	0.93889397	-1.96293071	-1.32123832	-1.32215502
IMAGE:1010307	Hs.377508 Tran	-1.257054146	-1.15685427	-0.87270665	2.48068584	0.90341673	1.32764336
IMAGE:450515	SLC26A2 Hs.302	-1.393655636	-0.71708961	-1.08444819	1.19141368	1.52113478	1.80912965
IMAGE:380620	PSEN2 Hs.2536	1.429152158	1.02985924	1.48517236	-1.7911154	-1.80005479	-1.95381236
IMAGE:2562939	SERPINF1 Hs.53	1.038416189	1.20917848	0.95443474	-1.92151013	-1.31684367	-1.32608163
IMAGE:809685	FAM62A Hs.632	1.036638613	1.42430337	0.8650073	-2.50293704	-1.36940187	-0.9003332

IMAGE:1557714	ADAM19 Hs.483	1.070770715	1.4978449	0.27000661	-1.34961887	-1.54580607	-1.29500675
IMAGE:209167	CSNK2A2 Hs.82	2.055703676	0.38219046	0.75460368	-1.81069627	-1.59313529	-1.19626424
IMAGE:1476320	NCF2 Hs.587558	1.076026619	1.63111322	0.26150383	-1.61881492	-1.21140511	-1.55736951
IMAGE:324513	GREM1 Hs.4009	1.787769041	0.49661331	1.21402638	-1.76337874	-1.45584892	-1.72487883
IMAGE:2119876	CCR5 Hs.536735	0.975175277	1.17085171	0.84208514	-1.24151698	-1.26932264	-1.76080729
IMAGE:1569463	ZNF511 Hs.4221	1.81104715	1.18044441	0.70031975	-2.0390256	-1.6234055	-1.70020593
IMAGE:447416	PLEKHB1 Hs.445	-1.677509413	-0.90665576	-0.64882874	1.72278579	1.08177797	1.88271992
IMAGE:2307514	MLC1 Hs.517729	-1.319097317	-1.45834221	-0.8967397	2.35084118	1.23734616	1.69889658
IMAGE:245881	Hs.596165 Tran	1.653816764	0.62064375	1.02119698	-2.13852427	-1.50669923	-1.0429635
IMAGE:192258	Hs.595974 Tran	-1.496759845	-1.18465039	-0.46310702	1.54177675	1.75085652	1.30836586
IMAGE:134948	SHRM Hs.13745	-2.019595054	-1.23064169	-0.2911803	1.77436031	1.60139745	1.86357273
IMAGE:1947396	Hs.157287 MRN	1.600493061	1.45965561	0.79462804	-2.60756967	-1.7930025	-1.18293588
IMAGE:240766	TIMP1 Hs.52263	1.270254469	1.75659664	0.30972869	-2.24736725	-1.38107026	-1.29900002
IMAGE:1877592	AI276654 853	-1.529193112	-1.30237697	-0.81393104	1.6760355	1.96878657	1.61994685
IMAGE:780937	SYT11 Hs.32984	1.185336978	1.56547757	0.99862086	-1.82979288	-1.54552598	-1.99917905
IMAGE:344262	KRTAP10-11 Hs.	-1.62105335	-0.71948996	-0.68598052	1.69039697	1.26753981	1.41035383
IMAGE:193606	SLC30A10 Hs.28	-1.810568772	-0.48542227	-0.99453186	1.38567729	1.92767196	1.37380213
IMAGE:1324230	CLDN8 Hs.16220	-0.902762453	-1.5576615	-0.48141196	1.25214434	1.43634473	1.60391181
IMAGE:772880	FLJ21127 Hs.21	1.376346404	0.44023293	1.65088372	-1.69976237	-1.70527201	-1.38343927
IMAGE:843098	BASP1 Hs.20164	1.214102602	1.32976741	0.48724062	-2.1184135	-1.2326646	-1.07377768
IMAGE:1010082	MLPH Hs.102406	-1.634079119	-1.2288123	-0.63771581	1.77930541	1.8528218	1.45935467
IMAGE:2541366	AQP3 Hs.234642	1.362658811	1.31060687	0.16053286	-1.93172701	-1.35901864	-0.91981894
IMAGE:2250736	SCGB2A1 Hs.97	-1.355071976	-1.23384997	-0.12389725	1.92155873	0.99255739	1.12413836
IMAGE:139689	Hs.504187 Tran	-1.622912006	-0.63915577	-0.54616175	0.94658609	1.85069466	1.2785231
IMAGE:897910	POSTN Hs.13634	0.767624667	1.36942855	0.86374011	-1.7453395	-1.22579658	-1.31411888
IMAGE:260138	HPSE Hs.44227	1.710136847	0.46944524	0.76033517	-1.54453973	-1.65905296	-1.01619939
IMAGE:1758590	ACSL3 Hs.47146	-1.35406945	-0.75686898	-1.17977024	1.05087767	1.68106305	1.90917974
IMAGE:366126	ZBTB5 Hs.16127	1.787609287	0.94929339	1.11695657	-2.4619319	-1.51882651	-1.52079131
IMAGE:1492468	C1orf34 Hs.1129	1.771155726	0.84497401	0.71278657	-1.98944643	-1.44808669	-1.3776447
IMAGE:1603404	KCNH2 Hs.4388	0.886766708	1.21121743	1.09062991	-1.65706213	-1.45894441	-1.39425705
IMAGE:1670631	FADS2 Hs.50274	-1.232595551	-1.25843225	-0.31201411	1.1362322	1.64750072	1.34282642
IMAGE:2578078	TUBB3 Hs.51174	1.59734699	1.22917368	0.55091155	-1.90990397	-1.58746648	-1.43104999
IMAGE:51772	ABHD5 Hs.1938	-1.444690403	-0.87752106	-0.6893796	0.75951736	2.06819849	1.51732584
IMAGE:1951183	AI338101 601	-2.042202584	-0.77974771	-0.81105322	1.86443906	1.88427376	1.49802914
IMAGE:714089	ZNF506 Hs.3519	-1.807225714	-1.38768853	-0.7699562	1.84336777	2.02394443	1.88750442
IMAGE:301995	SORBS2 Hs.481	-1.314208457	-1.47015863	-0.31612738	1.75819121	1.56778693	1.2457317

IMAGE:1665056	SLC9A8 Hs.4442	1.289975264	1.30358238	0.87904042	-2.07030155	-1.37709468	-1.54174076
IMAGE:415962	PCSK6 Hs.49849	1.644055794	0.8177812	0.85712761	-1.50896872	-1.62110598	-1.6340903
IMAGE:2015083	TCIRG1 Hs.4959	0.746127906	2.03057737	0.4734456	-2.3905082	-1.11132288	-1.25503383
IMAGE:310493	ACSL3 Hs.47146	-1.0053206	-0.84706731	-1.44169564	0.99523674	1.7528323	1.83263237
IMAGE:1631014	PPP1R3D Hs.422	0.788529671	1.92515142	0.87822227	-2.06955211	-1.85121635	-1.24753102
IMAGE:51955	FAM57A Hs.1541	1.741268741	0.70010884	0.98758796	-2.09384871	-1.57208453	-1.23061808
IMAGE:506548	RGS10 Hs.50120	1.214388561	1.7132608	0.68683257	-1.77443922	-1.77068971	-1.70488582
IMAGE:289203	FAM81A Hs.5311	-1.427019527	-1.02903597	-1.06992469	2.6926555	1.26427615	1.06455746
IMAGE:238821	PLA2G7 Hs.5848	-1.610169212	-0.90943275	-0.3460396	1.15370258	1.6716608	1.38658906
IMAGE:815575	ACTR1A Hs.1539	1.39756772	1.44113959	0.64377437	-2.45500253	-1.39293821	-1.21483819
IMAGE:206544	ARHGAP30 Hs.3	1.376703104	1.39710352	0.22290921	-1.65175504	-1.50226348	-1.28532793
IMAGE:1585510	MFI2 Hs.184727	-1.326014596	-1.02539872	-0.67390939	1.71521603	1.31407369	1.34021699
IMAGE:823718	CA6 Hs.100322	-1.481405571	-1.21535782	0.3284193	1.21609114	1.25284773	1.16568209
IMAGE:502814	TPST1 Hs.42119	1.233146722	1.34311368	1.01121341	-1.87017084	-1.84017985	-1.41805668
IMAGE:199180	PON3 Hs.44096	-1.506390046	-1.0950867	-0.16720595	1.02667544	1.53713227	1.54741485
IMAGE:142788	SERPINH1 Hs.24	0.94681797	1.15748761	1.12615384	-2.07439137	-1.33938099	-1.15037832
IMAGE:841260	SCOTIN Hs.4145	1.062728996	1.43690512	0.81327825	-2.70322893	-1.14665627	-0.91616378
IMAGE:384140	Hs.560705 CDN	-1.311875537	-1.59799289	-0.75131909	1.6352062	1.69323255	1.97551276
IMAGE:2049813	FAM26B Hs.2415	1.229441657	0.64553095	1.81225065	-1.99776671	-1.8738234	-1.2061821
IMAGE:488651	FAM46B Hs.6323	1.448330724	0.40892292	1.31877074	-2.1376262	-1.32348442	-0.97323327
IMAGE:897727	MAF1 Hs.19673	0.972811292	1.1188148	1.64442742	-2.35698117	-2.01934806	-0.81664418
IMAGE:916250	KLK3 Hs.171995	-1.070372479	-1.76376019	-0.36041324	1.69947158	1.53302945	1.46921452
IMAGE:754406	ITGAM Hs.17263	1.41521037	0.99725004	0.91834538	-1.39143885	-1.71369799	-1.66148549
IMAGE:843321	KRT7 Hs.411501	-1.192056274	-0.87118145	-0.93018796	1.46248298	1.16206662	1.63304195
IMAGE:2018808	PRCP Hs.523936	1.186394777	1.30372901	0.95618501	-2.17804709	-1.60076265	-1.1516072
IMAGE:435894	TMEM149 Hs.35	0.643365583	1.53143918	1.06904137	-1.92848806	-1.46548134	-1.20453946
IMAGE:854189	AA669383 896	1.465569823	0.7830203	0.79969798	-1.92200684	-1.55139172	-0.8991091
IMAGE:2435471	ALDH6A1 Hs.29	-1.816283551	-0.58054689	-0.6731315	0.98765266	1.57013225	1.87887512
IMAGE:1467469	RASGRP1 Hs.59	0.880219955	1.68466826	0.29630933	-1.50870012	-1.42371312	-1.28530574
IMAGE:784830	C10orf7 Hs.4128	1.406009229	1.41453014	0.3928728	-1.70135432	-1.67579158	-1.34475416
IMAGE:813757	FOLR2 Hs.43315	1.500556629	0.41676093	1.08628068	-1.04155268	-1.84850413	-1.34377038
IMAGE:255725	N27731 12849	-1.777497199	-0.42392577	-0.39252132	0.908875	1.4854845	1.3984266
IMAGE:68977	PSMB10 Hs.966	0.699497947	1.51274369	0.73982418	-2.26901703	-1.35243532	-0.62169033
IMAGE:840978	CD81 Hs.54457	1.041504574	0.93859482	1.25723392	-2.21482695	-1.59736841	-0.72949612
IMAGE:49560	CAPN1 Hs.50284	1.156157969	1.20392995	0.71180505	-2.3673931	-1.28701138	-0.77548371
IMAGE:1660303	Hs.132014 Tran	-1.159560046	-1.15956005	-0.61668926	0.90389937	1.4803233	1.86531905

IMAGE:809894	ACSS2 Hs.5170[illegible]	-1.300794266	-0.88626903	-0.6363165	0.86803888	1.85794989	1.3500018
IMAGE:588661	ZNF204 Hs.819[illegible]	0.943681453	1.63727432	0.32161912	-1.60255828	-1.41166946	-1.25922981
IMAGE:1909775	MRAS Hs.52702	-1.332273928	-1.58798602	-0.53499054	1.73270468	1.40061542	1.915808
IMAGE:825323	CKAP1 Hs.3105[illegible]	1.095276435	1.44563895	0.87019506	-2.43674935	-1.70417318	-0.75819438
IMAGE:1556638	ZNF592 Hs.793[illegible]	0.977580757	0.89831045	1.46822222	-2.26610444	-1.37612144	-1.00688871
IMAGE:825813	Hs.594539 **F[illegible]	-1.401927502	-1.11988033	-0.77952325	1.09078495	1.59061242	2.07571844
IMAGE:149745	LOC442609 Hs.[illegible]	-1.583159194	-1.3626625	-0.05054009	1.2217008	1.52738941	1.73281744
IMAGE:131887	C1orf115 Hs.51[illegible]	-1.899269593	-0.98084315	-0.02826417	1.35814438	1.33276074	1.66459421
IMAGE:743268	DDX54 Hs.5068[illegible]	1.391778163	1.03801716	1.05852505	-2.37064953	-1.36319972	-1.23400005
IMAGE:1552374	PLSCR3 Hs.534[illegible]	1.068769522	1.32416261	0.86256325	-2.61007334	-1.19122284	-0.86630608
IMAGE:814167	DPP8 Hs.59110[illegible]	0.862656925	1.70006777	0.84710501	-2.73731248	-1.40941814	-0.75623768
*mitoch. cont. IMA[illegible]	140755	-1.098515291	-0.89900761	-1.22708691	0.78073624	1.73393962	2.01546712
IMAGE:377560	CD3D Hs.50404	0.756941492	1.61826156	0.2322336	-1.10017389	-1.19306398	-1.55985871
IMAGE:1416129	CFC1 Hs.56754[illegible]	1.218039577	1.32153183	0.56065679	-2.41805723	-1.09480529	-0.99731558
IMAGE:306812	VASN Hs.37257[illegible]	1.401953043	0.44356434	1.61104733	-1.96963257	-1.43493493	-1.37751774
IMAGE:485854	SNX19 Hs.4440[illegible]	1.461441785	-0.53802169	1.73804488	-0.87823924	-1.22425928	-1.45518773
IMAGE:1861233	EIF5B Hs.15868	-0.885321542	-1.53070207	-1.59282881	1.83083214	1.78414252	2.00009677
IMAGE:2578396	TUBB6 Hs.1934[illegible]	1.64789293	1.25249378	-0.02876773	-2.09795319	-1.30645382	-0.91021339
IMAGE:1880372	PRKD1 Hs.5089[illegible]	-1.464990536	-1.57871735	-0.31364198	1.89527564	1.5847834	1.47755526
IMAGE:415584	Hs.49943 **Tr[illegible]	1.092261221	0.72214799	1.38468127	-1.50724897	-1.48430807	-1.46090835
IMAGE:841046	VSIG4 Hs.8904	1.396513821	0.412206	1.25914002	-1.01166733	-1.75219798	-1.52313943
IMAGE:261472	ORF1-FL49 Hs.5[illegible]	-1.55600277	-1.04671854	-0.48276116	1.09957744	1.77587694	1.63207904
IMAGE:1692766	SLURP1 Hs.103[illegible]	1.297799885	0.25657441	1.12907576	-0.80874365	-1.15365908	-1.78050342
IMAGE:725152	SLC43A3 Hs.99[illegible]	1.047803442	1.31006436	0.83274949	-2.0856526	-1.40002761	-1.09205836
IMAGE:624372	ST3GAL6 Hs.14[illegible]	-1.137346279	-1.68553149	-0.73044592	1.8300123	1.68354701	1.63381474
IMAGE:345329	ABHD5 Hs.1938	-1.447413506	-0.99719577	-0.32549391	0.85154673	1.74523268	1.4770019
IMAGE:2014138	ACSL1 Hs.4066[illegible]	-1.591884668	-0.67415786	-0.38722776	1.08291441	1.59140998	1.20877409
IMAGE:810133	TRIM25 Hs.528[illegible]	1.382295308	1.25485739	0.50567687	-1.8629307	-1.45165376	-1.27324067
IMAGE:249603	STK39 Hs.2762[illegible]	-1.629418302	-0.99434203	-0.61934461	1.80366577	1.30467727	1.60147822
IMAGE:1658925	FBXW8 Hs.4354	-1.425053433	-1.13343985	-0.37078561	2.10070639	0.72156142	1.47895413
IMAGE:985230	TGM4 Hs.43826	-1.242449511	-1.34039345	-0.54015519	1.33547124	1.62930305	1.58468415
IMAGE:151240	SORBS2 Hs.481	-1.170756345	-1.55216171	-0.25366929	1.88755756	0.59549332	1.91841282
IMAGE:377368	AVEN Hs.55596[illegible]	1.7977578	0.90108515	0.47703371	-2.07077755	-1.3772573	-1.19652172
IMAGE:740476	IRF1 Hs.436061	0.885526755	1.76563645	0.58269331	-2.08531663	-1.30457416	-1.31522065
IMAGE:166195	RNH1 Hs.53068	1.338113592	0.98925167	1.23016387	-3.01838197	-1.18192014	-0.82845061
IMAGE:839580	MLSTD1 Hs.298[illegible]	-1.576847346	-0.56312261	-0.33350786	0.89543491	1.47122751	1.26017734

IMAGE:767405	MAP3K6 Hs.194	0.983310009	1.10215282	0.97188282	-1.96733422	-1.22599503	-1.14971852
IMAGE:1069986	XKR6 Hs.12591	-1.59181345	-0.86274421	-0.47712292	1.50456455	1.50493805	1.26873754
IMAGE:490361	SDC2 Hs.1501 S	1.378668408	0.93028195	0.86843554	-1.98944834	-1.6879471	-0.87157451
IMAGE:52629	CAMK1 Hs.4348	0.978903195	1.30947755	1.22228258	-1.5236405	-1.60269089	-1.83409294
IMAGE:1350439	PPM1F Hs.1127	0.8622137	1.14197957	1.36186573	-2.57767945	-1.14940107	-0.98154154
IMAGE:264895	TRAFD1 Hs.514	0.866024984	1.57129216	0.77145507	-1.83422666	-1.55772986	-1.22833803
IMAGE:118399	FLJ25037 Hs.56	-1.190266313	-1.36631042	-0.94481053	1.63473867	1.89880483	1.48233477
IMAGE:162722	PLA2R1 Hs.410	-1.033907161	-1.29233287	-0.3396144	1.09053321	1.36624217	1.45710267
IMAGE:1897415	AI301734 462	-1.364826389	-1.39616114	-0.42478394	1.65442584	1.92599366	1.09204171
IMAGE:1535957	EXOC3 Hs.4814	0.999254317	1.2018868	1.0446054	-2.68600339	-1.12283851	-0.79862653
IMAGE:309929	GPR176 Hs.371	1.655322685	0.9438976	0.89719336	-1.98244901	-1.78694288	-1.25093024
IMAGE:1009945	AA224758 15	-1.434532844	-1.76654144	-0.10237046	1.58538906	1.83575396	1.50843148
IMAGE:704237	MOBKL2A Hs.86	0.867816527	1.35275378	1.1233211	-2.61460656	-1.39444185	-0.72595845
IMAGE:2430220	AI871056 187	1.201365804	0.84695721	1.06084591	-1.91105669	-1.41581731	-1.07166791
IMAGE:2385051	SOLH Hs.63221	1.620584167	0.80878336	1.01964072	-2.23731466	-1.46241389	-1.21887365
IMAGE:198453	HLA-E Hs.11835	1.149400095	0.77108145	1.33426921	-2.16127807	-1.10822617	-1.27905459
IMAGE:190887	MYD88 Hs.8211	1.794057917	1.10264181	0.07104897	-2.07026673	-1.21844208	-1.14515198
IMAGE:461488	ARRB1 Hs.5032	0.866997909	1.74946434	1.33529343	-2.15066959	-1.66531305	-1.77782752
IMAGE:510736	CHMP4C Hs.183	1.894621888	-0.59148672	1.23326872	-1.2530123	-1.24784009	-0.99543627
IMAGE:359701	Hs.99913 Beta	-1.379594746	-1.45739685	-0.03102502	1.43109871	1.68326088	1.17990908
IMAGE:726768	FLJ37543 Hs.58	1.176361374	1.3196741	0.69865229	-2.54849562	-1.21903123	-0.84984172
IMAGE:490140	DUSP14 Hs.914	2.024764567	0.30424891	0.58462924	-1.62874246	-1.33004183	-1.26552249
IMAGE:270127	NSUN7 Hs.5909	-1.26117931	-0.89523578	-1.36194637	2.37783744	1.3762659	1.18295225
IMAGE:752557	GPSM3 Hs.5200	0.841192099	1.74910984	0.17213062	-1.45785445	-0.8992585	-1.74350324
IMAGE:813630	PIM1 Hs.81170	1.850829974	0.65255662	-0.35147032	-1.37656164	-1.61495497	-0.32422537
IMAGE:289666	RAGE Hs.10411	1.393853663	1.25237044	0.59093638	-1.83704833	-1.71833506	-1.15262325
IMAGE:2015501	CALM3 Hs.5154	-1.501753404	-1.36129336	-0.21793946	1.61810664	1.92121666	1.02767118
IMAGE:954096	HMGCS1 Hs.397	-1.732433281	-0.74472636	-0.59081862	1.16979871	1.86595358	1.41851045
IMAGE:196282	CSF1R Hs.4838	1.294777757	1.24256575	1.03835879	-1.95599982	-1.01618369	-2.13178024
IMAGE:286446	ZNF626 Hs.128	-1.825377299	-1.05251348	-0.5062789	1.40350827	2.19671061	1.34946591
IMAGE:502012	MS4A4A Hs.325	0.831298695	1.2682778	0.71433775	-0.93804027	-1.44251051	-1.66173615
IMAGE:2098508	ATP2B2 Hs.268	-0.910210369	-1.53127186	-0.96312668	2.14974026	1.10001715	1.6163743
IMAGE:1536940	LOC642851 Hs.	-1.060508662	-1.29173855	-0.45738208	1.71389715	1.23403044	1.15217083
IMAGE:291955	PTPLA Hs.11406	1.114909987	-0.75418233	2.24051892	-1.05261407	-1.41760627	-0.87151979
IMAGE:223483	DUSP1 Hs.1716	1.026434671	1.48773107	0.7102451	-2.18853249	-1.25474318	-1.21577931
IMAGE:840663	HSPC159 Hs.37	2.075349693	-0.38064395	0.89741134	-0.92170561	-1.6843151	-1.05780207

IMAGE:2119167	CYP4F8 Hs.2685	-1.383676303	-0.60947146	-0.61111532	0.9066576	1.63247464	1.21448356
IMAGE:280782	CP Hs.558314 C	1.20605834	0.5789366	1.20959854	-1.16676296	-1.58779428	-1.43493336
IMAGE:23579	C9orf91 Hs.522	-2.400967665	-0.35374499	-0.78721436	1.12339901	2.03199784	1.96069008
IMAGE:2568695	UBE2L6 Hs.4257	1.146080256	1.44463384	0.42480358	-1.46274148	-1.74741651	-1.20691763
IMAGE:79710	KIAA0174 Hs.23	1.017464323	1.78473052	0.69163895	-1.70913348	-1.84927342	-1.50943406
IMAGE:71545	MAP4K1 Hs.954	0.920446185	1.62172028	0.59207109	-1.35074714	-1.41780393	-1.78478748
IMAGE:506018	CADPS2 Hs.126	-1.916747194	-0.81382047	-0.27430548	1.91431473	1.10090628	1.42351234
IMAGE:68894	FCGRT Hs.1119	1.111580016	0.53498433	1.57642577	-1.95134556	-1.81750058	-0.67153259
IMAGE:986126	PIP Hs.99949 Pr	-1.355296557	-0.67776897	-0.57801491	1.52938339	1.16936873	1.07336482
IMAGE:725709	PRSS1 Hs.5115	1.633058691	0.59583306	0.51810187	-1.52926493	-1.33979514	-1.12190489
IMAGE:768643	Hs.506325 **T	-1.098288188	-0.99362876	-1.41625351	1.24732906	1.74072354	1.92013971
IMAGE:898222	ATPAF2 Hs.5288	1.291927598	1.51069719	1.2372352	-2.46294673	-1.88385076	-1.40368369
IMAGE:811050	C19orf6 Hs.515	1.115085353	1.20344515	0.81024407	-2.02665754	-1.36948158	-1.09446172
IMAGE:433491	Hs.579591 **T	1.37568416	0.22590644	1.24908629	-1.99062649	-1.40350596	-0.56961131
IMAGE:1155709	CD46 Hs.51040	-1.139233275	-1.19479053	-0.98880131	1.29524307	1.77602704	1.66576725
IMAGE:2281706	ITK Hs.558348	0.663575501	1.67128334	0.33831694	-1.11198806	-1.33944441	-1.47375196
IMAGE:1606300	BMPR1A Hs.524	-1.376169188	-1.27361577	-0.71787392	1.50094251	1.62547166	1.74560566
IMAGE:786067	CDC25B Hs.153	0.752877804	1.47478919	0.97897093	-1.8227989	-1.50150867	-1.24090659
IMAGE:358990	LRRC56 Hs.567	1.577273404	0.6214428	1.00377504	-2.29457959	-1.64422062	-0.61399289
IMAGE:1558326	FRZB Hs.12845	-1.213033623	-1.30249453	-0.49190089	2.18900924	1.22487345	0.97428566
IMAGE:995444	ARSB Hs.14910	-1.533348641	-0.51345439	-1.13180011	2.03105636	1.42037378	1.03352455
IMAGE:1947123	AI351836 971	1.819542674	1.54905881	0.6331445	-2.61406087	-2.20833508	-1.02193691
IMAGE:1090417	MUC5B Hs.5233	-1.590866093	-1.54301471	-0.82205382	2.00357055	1.97127087	1.75354706
IMAGE:1470060	TUBA3 Hs.5243	1.399414491	1.10833307	0.80560838	-1.64283423	-1.58909612	-1.53670146
IMAGE:366950	SLPI Hs.517070	1.157491846	0.71730293	1.10067571	-1.84200986	-1.44420615	-0.9018208
IMAGE:1691326	AI128614 435	-1.529481542	-1.4315041	-0.45288235	1.53159313	1.64052099	1.83546728
IMAGE:320865	MARVELD2 Hs.4	0.560358917	2.13327587	0.26653085	-1.83662852	-1.46841786	-1.06856936
IMAGE:285760	Hs.587521 Tran	-1.171794004	-1.6439852	-0.83732524	2.2132266	1.45575323	1.60134552
IMAGE:298155	ACADM Hs.4450	-1.600325504	-0.50123144	-0.86077492	1.06229589	1.79960483	1.36640334
IMAGE:878630	NBEA Hs.49117	-1.666671717	-1.07761704	-0.11360713	1.40127452	1.27728932	1.5798782
IMAGE:1915694	Hs.604477 Tran	-1.818724018	-1.21092621	-0.54029986	2.2149364	1.67982067	1.32509309
IMAGE:1568056	TATDN3 Hs.530	-1.353829263	-0.93384696	-1.28231876	1.16083506	2.12436204	1.74921568
IMAGE:52076	OLFM1 Hs.5224	1.13933122	1.52004786	0.7854887	-1.96994797	-1.91116084	-1.08982068
IMAGE:50483	FBLN5 Hs.3327	1.167402438	0.43602437	1.56760158	-1.82744647	-1.51419762	-1.0229981
IMAGE:252382	H87106 9035	-1.400276686	-0.97974882	-0.60056098	0.77595502	1.85043935	1.69434511
IMAGE:825270	PREX1 Hs.1533	0.696197429	1.64740028	0.62995192	-1.62073065	-1.4743476	-1.20775822

IMAGE:296454	MGC7036 Hs.48	1.053052092	1.68325457	0.77404983	-1.99302852	-1.9498933	-1.12910046
IMAGE:999777	AA552459::AI	-1.676939206	-1.50989258	-0.76071379	1.92732368	1.38178569	2.42203056
IMAGE:739901	CYP51A1 Hs.417	0.846879518	1.34505583	1.21611608	-2.73031944	-1.08633761	-0.99139107
IMAGE:744939	GRAMD1C Hs.24	-0.764963149	-1.75598503	-0.6920389	1.51111494	1.71563006	1.41972589
IMAGE:824799	NOB1 Hs.27169	1.338940171	1.54775249	0.47052658	-2.35120342	-1.69712954	-0.86986426
IMAGE:82556	POR Hs.354056	1.45283696	0.09155189	1.49122627	-1.30677886	-1.8077594	-1.06607786
IMAGE:491415	CYBRD1 Hs.221	1.186259808	0.83890094	1.40990182	-1.90277567	-1.43011383	-1.46722889
IMAGE:188232	KLF4 Hs.376206	1.524942345	0.20560195	1.13601179	-2.03401273	-0.66828927	-1.31352918
IMAGE:268837	C1orf21 Hs.497	1.772217975	0.87973001	0.60229611	-1.48108616	-1.94485011	-1.30485585
IMAGE:2418870	RABAC1 Hs.114	1.028100859	0.95504375	0.99570162	-2.08270272	-1.40136574	-0.73527547
IMAGE:795543	PRDX4 Hs.8338	1.270916086	1.18737899	0.91612913	-1.70128463	-1.88555763	-1.24576176
IMAGE:510145	SGK2 Hs.47279	-1.455486516	-0.81770513	-0.26505665	0.89040792	1.51012539	1.34057498
IMAGE:2029476	CYP4F2 Hs.5584	-1.344067112	-0.96884296	-0.28495983	0.84611841	1.78735867	1.19208781
IMAGE:269606	MPG Hs.459596	1.058588535	1.37282315	1.02418812	-2.2565866	-1.42965342	-1.24111265
IMAGE:140635	C9orf150 Hs.44	0.414787279	1.80071782	0.44093691	-1.59394035	-1.35249207	-0.92799637
IMAGE:151620	TMEM54 Hs.534	1.230113278	0.65577863	0.93792161	-2.17821202	-1.17767429	-0.64535356
IMAGE:68061	ALDH1L1 Hs.434	-1.400322455	-0.87800155	-0.39692202	1.05353906	1.41780293	1.44229654
IMAGE:108377	TUBG1 Hs.2796	1.456382765	1.17397677	0.94933564	-2.43857355	-1.36992359	-1.3237117
IMAGE:1630961	PPM1F Hs.1127	0.993140838	0.88289198	1.27407126	-2.26062955	-1.35413896	-0.7918698
IMAGE:68637	LOC643008 Hs.5	1.81955709	0.10191595	0.8235285	-1.15785695	-1.74734326	-1.00641995
IMAGE:810326	PDIA3 Hs.59109	0.637310646	1.82878434	-0.11723965	-2.76189372	-0.42815613	-0.36254306
IMAGE:781362	PLEC1 Hs.43424	0.91358397	0.62254843	1.54173567	-2.35995951	-1.09274227	-0.77866642
IMAGE:376736	Hs.62927 Trans	1.219475147	-0.0276516	1.43757171	-0.94386803	-1.60583929	-1.03499263
IMAGE:1699663	OCA2 Hs.13093	-1.135067729	-1.21679961	-1.00429673	2.02289061	1.40565181	1.35462949
IMAGE:246661	WSB1 Hs.44601	-0.58788115	-0.87689635	-1.47011422	0.41738172	2.06136819	1.55605911
IMAGE:380890	FAM46C Hs.356	-1.67667087	-0.26013591	-0.85115239	1.5928168	1.32944843	1.04688542
IMAGE:824510	FAM96B Hs.982	1.47904323	1.21155633	0.52192647	-1.88326708	-1.59146319	-1.21357716
IMAGE:2236882	ZNF710 Hs.459	1.50340784	0.45412871	1.59298045	-2.28807272	-0.97807332	-1.66138436
IMAGE:1033363	AA621402 15	-1.515183062	-0.82625317	-0.82170169	1.54114163	1.69408939	1.30405045
IMAGE:156386	FLOT2 Hs.51403	0.600896281	1.52840269	0.80463811	-2.38264543	-1.15266399	-0.66443667
IMAGE:1035182	AA670190 12	0.885127353	1.58897368	0.86290063	-2.13850119	-1.39231825	-1.25895789
IMAGE:343987	DPP4 Hs.36891	1.341094219	0.39098347	1.16331439	-1.28262198	-1.54609807	-1.22353947
IMAGE:294018	N64033 4937	-0.846472651	-1.39299403	-1.00237953	1.4542129	1.64952015	1.50844138
IMAGE:530139	GNAI2 Hs.7726	1.167193236	1.73420791	0.86368683	-2.17235092	-1.85775889	-1.40160045
IMAGE:856568	C14orf92 Hs.55	1.493299963	1.27770361	0.68494044	-2.31673155	-1.70798889	-0.98796047
IMAGE:951108	ELF3 Hs.67928	-1.709597684	-1.03452119	-0.22264112	1.65304326	1.10985258	1.63158388

IMAGE:998871	KIF13A Hs.1899	-1.3878577	-1.231181	-0.3154824	1.63549121	1.37104698	1.31637285
IMAGE:51865	CA2 Hs.155097	1.272151538	1.55419444	0.31064166	-1.52288373	-1.70778948	-1.39714782
IMAGE:198045	ARG1 Hs.44093	1.950738801	-0.68983713	0.42691946	-0.44043306	-1.32295392	-0.66161485
IMAGE:742565	C12orf10 Hs.50	1.324555177	1.12871026	0.94899375	-2.36107333	-1.6266549	-0.87841212
IMAGE:726521	CTDP1 Hs.4654	1.494777419	0.98679088	0.82682916	-2.3467357	-1.28482766	-1.12432553
IMAGE:1636154	Hs.131034 Tran	-1.286962647	-0.82775021	-0.45951924	1.14564348	1.45991699	1.14090786
IMAGE:2009477	CD6 Hs.502710	0.670842484	1.54872642	0.51518187	-1.64362644	-1.0740501	-1.25565415
IMAGE:1855351	EPSTI1 Hs.5464	1.080854281	1.50270766	-0.03518821	-1.06966347	-1.41248609	-1.34920809
IMAGE:357544	SPRR4 Hs.4336	0.621847249	0.83232754	1.45875101	-1.75025813	-1.08921454	-1.16522828
IMAGE:2572015	TIMP1 Hs.5226	0.724232017	1.51029781	0.5205935	-1.64728629	-1.11787889	-1.23737144
IMAGE:739126	TSTA3 Hs.4041	0.617915	1.49031913	0.62847399	-2.21772035	-0.94472659	-0.78549674
IMAGE:855843	UQCRB Hs.1312	-1.577191827	-1.03868152	-0.5502146	0.62390104	2.13336174	1.85431549
IMAGE:826405	INPP5D Hs.2628	1.373470372	1.41313048	0.39089134	-1.52840974	-1.37968436	-1.76042134
IMAGE:1916614	FLT3LG Hs.428	0.325764822	0.7138532	2.21378956	-1.69456277	-1.53075923	-1.10134197
IMAGE:840821	SSR4 Hs.40922	0.975243319	1.28390425	0.57797855	-2.25607348	-1.29196199	-0.56315907
IMAGE:395604	JOSD1 Hs.3094	1.592430365	0.95242184	0.78475228	-2.35734107	-1.35645645	-1.08442114
IMAGE:251682	TINF2 Hs.49619	1.335931462	1.28851307	0.8332965	-2.22963531	-1.5734833	-1.1751688
IMAGE:81332	MSN Hs.87752	0.932427565	1.39514954	0.42879828	-2.15188266	-1.21134994	-0.66413091
IMAGE:1566690	AI091654 104	-1.19965484	-1.25630868	-0.51149952	0.98863122	1.35285247	1.981836
IMAGE:526282	CSK Hs.77793 *	1.117703151	1.26859224	1.10201069	-2.46953415	-1.37407933	-1.11334298
IMAGE:996112	Hs.633916 Tran	-1.3787802	-0.87991119	-0.1916335	1.5480755	1.08177131	0.99773216
IMAGE:340644	ITGB8 Hs.59217	1.452447832	1.20651957	0.47776887	-0.82539998	-1.75139519	-2.00886702
IMAGE:1636162	LOC388323 Hs.	-1.19329482	-0.96556104	-0.52686694	1.3139337	1.60954966	0.9733841
IMAGE:1087295	PREPL Hs.44434	-1.15885479	-1.27597145	-0.52853942	1.53564175	2.08157619	0.69569569
IMAGE:795790	MKRN1 Hs.4903	1.323142814	0.53319941	1.15438218	-2.01203413	-1.30647105	-0.90898589
IMAGE:298899	GBP2 Hs.38656	1.461374799	1.07490809	0.87199567	-2.11695872	-1.48607793	-1.29138227
IMAGE:1010093	COG4 Hs.20868	-1.715077322	-1.00460032	-0.40821452	2.08571532	1.34949372	1.15457558
IMAGE:281597	TSPAN33 Hs.27	0.668422042	1.5537828	0.80658596	-1.87887598	-1.51063737	-0.95202635
IMAGE:769698	Hs.536417 Tran	1.29549954	0.67727773	1.0894256	-1.94593309	-1.42154852	-0.95346629
IMAGE:2466502	PSMC4 Hs.2115	1.402838838	1.17792776	0.35555502	-2.24137649	-1.29606946	-0.77814772
IMAGE:1011676	EIF3S3 Hs.4925	1.706371449	0.96355033	0.39980216	-1.87911428	-1.2765196	-1.3490015
IMAGE:1670741	ZNF428 Hs.990	1.299743699	0.24936746	1.02426041	-0.60435867	-1.85302022	-1.14661335
IMAGE:129725	RBPSUH Hs.479	0.992633658	1.25999714	0.65445498	-2.20976668	-1.14753878	-0.83970947
IMAGE:592728	ACP6 Hs.56215	-1.457746452	-0.94796738	-0.41805691	0.90818043	1.62414403	1.59881743
IMAGE:1842541	PDE6A Hs.5673	-1.085352046	-1.05243622	-0.77265171	1.18213687	1.64172408	1.34863609
IMAGE:358217	GPC4 Hs.58367	-1.166818592	-1.39507244	-0.25881189	1.02216447	1.26982706	1.87435989

IMAGE:186307	LOC400128 Hs.4	-1.270274899	-1.22628926	-0.59553521	2.0732934	1.04930774	1.36666412
IMAGE:2284924	TBC1D9 Hs.480	-1.416139753	-0.65933542	-0.98142561	1.65764555	1.31212193	1.37022729
IMAGE:207358	SLC2A1 Hs.4737	1.6317543	0.47903724	0.6764707	-1.68031452	-1.31242609	-1.01903508
IMAGE:361097	UBE2D3 Hs.518	0.549643862	1.45969337	0.5395009	-2.12936719	-1.22495267	-0.3340621
IMAGE:229365	PTPRC Hs.19203	0.963101616	1.43311075	-0.05294753	-0.93136349	-1.15168027	-1.44509038
IMAGE:2541394	EIF3S7 Hs.5568	1.298227793	1.21644621	0.46859907	-2.30856718	-1.27266713	-0.77652553
IMAGE:756595	S100A10 Hs.143	1.603182416	0.65315516	0.57972676	-2.15283172	-1.04572824	-0.91060485
IMAGE:207288	INSIG1 Hs.5208	-1.265114376	-0.73076401	-0.6018654	0.87912263	1.57032956	1.29669713
IMAGE:81203	PON3 Hs.44096	-1.619451762	-0.66868025	-0.1900049	0.68996021	1.62436401	1.35537992
IMAGE:745057	MOBKL2A Hs.86	1.044754223	1.36503946	0.90294007	-2.4525569	-1.18622684	-1.10458187
IMAGE:1276003	CIP29 Hs.50567	-1.475167168	-1.04116056	-0.48330925	2.65666742	1.23728243	0.4846783
IMAGE:769028	MEOX1 Hs.438 M	0.951330266	0.80418901	1.2297951	-1.92563902	-1.03001878	-1.21486499
IMAGE:2130772	CLDN15 Hs.387	0.617408815	2.19220782	1.05243617	-1.95773558	-1.92184582	-1.65038876
IMAGE:704519	HMGCS1 Hs.397	-0.915357506	-0.8911472	-0.96935106	1.05143298	1.49023336	1.37977955
IMAGE:85128	C1QB Hs.8986 C	1.068859668	0.88865379	0.95749648	-1.3913809	-1.38378795	-1.35797194
IMAGE:451855	PPM1F Hs.11272	0.97531703	1.18226123	1.36506193	-2.13660633	-1.3690685	-1.43701997
IMAGE:587388	LIPH Hs.68864 L	-1.052420654	-1.42559545	-0.5441173	1.66841643	1.50932612	1.21942823
IMAGE:878600	TIA1 Hs.516075	-1.355243623	-1.43250927	-0.62062558	1.90863579	1.35588921	1.69288632
IMAGE:357298	Hs.568928 MRN	0.946542771	1.40946255	0.90445915	-1.8232606	-1.37436869	-1.46695264
IMAGE:40056	CSPG4 Hs.5130	1.234054985	1.58815736	0.33135818	-1.88600977	-1.27557955	-1.48592693
IMAGE:75581	T58362::T584	1.20654388	0.21855725	1.29758149	-2.44407861	-1.332058	0.01650804
IMAGE:783442	ALOX15B Hs.11	-1.392351141	-0.67053641	-0.37807588	0.9615204	1.50282779	1.10257798
IMAGE:37728	ANKRD6 Hs.575	-1.373602019	-1.27421254	-0.69775359	2.41461229	0.77245635	1.65684519
IMAGE:502326	ATP2B4 Hs.3435	1.400793679	0.91816826	0.60802209	-2.20272704	-1.15138492	-0.88435856
IMAGE:983891	MCF2L2 Hs.5848	-1.496442608	-1.28987257	-0.4444667	1.96837177	1.51080674	1.25587765
IMAGE:788520	LOC400948 Hs.5	1.025111395	1.40723979	0.81315751	-2.20947809	-1.28472738	-1.1707682
IMAGE:1657539	UBIAD1 Hs.5229	-1.289445992	-0.77230585	-0.92610467	0.97438536	1.61745249	1.65842074
IMAGE:1618989	ELOVL5 Hs.5201	-1.330766217	-0.70935788	-0.59842247	0.93200807	1.56319233	1.31301384
IMAGE:377692	MFAP5 Hs.5128	1.518148914	-0.40034274	1.2811187	-1.02209297	-1.15150602	-1.10450865
IMAGE:999104	PRR5 Hs.102336	-1.288419281	-0.97712269	-0.61244384	1.64188585	1.16632954	1.35565237
IMAGE:1475987	EXOSC7 Hs.115	1.453551036	0.65209271	1.10780564	-2.00320394	-1.36017343	-1.17984531
IMAGE:1839226	Hs.458901 Tran	-0.984575383	-1.22645032	-0.91669435	1.69744055	1.51473395	1.250232
IMAGE:795803	HN1 Hs.532803	1.362248193	0.80569773	1.15109512	-1.85472224	-1.66937017	-1.16669537
IMAGE:826994	PLEKHA4 Hs.94	1.16714861	1.12929843	0.79789912	-1.72986181	-1.85602907	-0.85615358
IMAGE:2249522	FAU Hs.387208	1.091130025	0.96042441	0.86645766	-2.1247581	-1.39351315	-0.64213247
IMAGE:2072912	AATF Hs.195740	1.51965304	1.1992386	0.63203687	-2.74356332	-1.17898644	-0.9458338

IMAGE:67884	T52763::AI82	-1.305302498	-1.16105352	-0.68809173	1.45138285	1.42870735	1.6795585
IMAGE:1733935	DHX8 Hs.46310	1.542537525	0.94289157	1.26864518	-2.86040342	-1.17086042	-1.28268629
IMAGE:379346	Hs.203961 Trar	-1.118050856	-1.30096986	-1.29555004	2.06485279	1.62759732	1.55551852
*mitoch. cont. IMA(	147522	-1.286400902	-1.15430557	-0.58665265	1.83807024	1.27874766	1.27755761
IMAGE:1639090	PDZD6 Hs.3914	-1.316514377	-1.40803278	-0.61561737	1.70366254	1.67062862	1.48205129
IMAGE:767991	Hs.43744 MRN/	-1.972554686	0.1021727	-0.44997787	0.4057223	1.39590786	1.56641516
IMAGE:1632487	WISP2 Hs.5921	0.844059738	-0.08634873	1.89560172	-1.16972048	-1.15812259	-1.1782256
IMAGE:1558847	AA975259 13	-0.952530301	-1.75571828	-0.69210269	1.7051396	1.70133663	1.52102499
IMAGE:744944	MYO6 Hs.14938	-0.478978749	-1.70787372	-0.9521517	1.1038283	1.80052466	1.56611536
IMAGE:222454	CD300A Hs.968	0.759592121	1.78664368	0.16458439	-1.03433723	-1.62555511	-1.36519185
IMAGE:42558	GATM Hs.75335	1.184646941	0.69936691	1.35015297	-2.32392769	-1.44276131	-0.74702299
IMAGE:1132429	TOB1 Hs.53155(	-0.88882907	-1.51092467	-0.93727962	1.33163474	1.84759777	1.59199762
IMAGE:292388	TMEM97 Hs.199	-1.359812346	-0.6838893	-0.5650671	0.92537243	1.59739967	1.24911425
IMAGE:783249	BASP1 Hs.2016	-1.269360116	-0.97173829	-0.74064369	2.35746824	1.22169082	0.70829317
IMAGE:1950782	CD1B Hs.1310 (	0.715012508	1.19766704	0.82639432	-1.63350581	-0.90952402	-1.3589824
IMAGE:417800	Hs.59203 Trans	-1.112080615	-1.6038087	-0.17340682	1.74894267	1.15623471	1.38541512
IMAGE:682418	ELF4 Hs.271940	0.86866597	1.52481539	0.70462861	-2.21113537	-1.19072653	-1.0691459
IMAGE:1702586	RCSD1 Hs.4938	0.818699549	1.70582175	0.23066426	-1.31075583	-1.31944601	-1.44491044
IMAGE:183194	CASP4 Hs.1383	1.183954078	1.17531903	0.70989008	-1.7621076	-0.83599896	-1.8281657
IMAGE:2239290	CXCL12 Hs.522	0.780471574	-0.46855991	2.19150278	-1.02109468	-1.13851045	-1.04764085
IMAGE:810728	DMKN Hs.4177	1.223210644	0.55671893	1.13661801	-1.74039952	-1.32403077	-1.0262366
IMAGE:244355	IL2RG Hs.84 Int	0.187479391	1.63306889	0.76757248	-1.95567668	-0.6370019	-1.09760943
IMAGE:186768	C2orf18 Hs.516	1.154031128	0.79397971	1.31909206	-2.27724657	-1.21794211	-1.07569266
IMAGE:725390	GSTP1 Hs.5238	1.327885192	0.71401777	0.66989605	-2.17739369	-1.14628826	-0.57654256
IMAGE:1084278	Hs.1247 Transc	1.562768131	0.91566207	0.33304424	-2.2346993	-1.27023489	-0.62901642
IMAGE:2508563	TNFAIP3 Hs.591	0.896821303	1.51557886	0.23618823	-1.81836812	-0.94648247	-1.14898495
IMAGE:1575419	FTL Hs.433670	0.915908949	1.06966578	0.85758739	-1.73825202	-1.29636683	-1.01572748
IMAGE:1573311	NFKBIE Hs.4582	0.099018007	1.94386567	0.84705325	-1.40934765	-1.34817779	-1.36561665
IMAGE:1609795	AA991624 875	0.814440374	1.5317649	0.33327207	-1.6057181	-1.32479059	-1.00538931
IMAGE:470279	CNTNAP1 Hs.40	-1.712027884	-1.082525	-0.40335634	1.84369889	1.72217075	1.13015512
IMAGE:32565	PIAS1 Hs.16245	-1.142310925	-0.89949576	-0.586392	0.14253272	1.90134517	1.75182215
IMAGE:342349	MAP3K14 Hs.40	0.484923635	1.0599207	1.36384772	-2.25695362	-0.90720401	-0.85791854
IMAGE:926239	C10orf99 Hs.29	1.670689511	0.1843178	0.37205567	-0.87848566	-1.32024188	-1.04885302
IMAGE:486102	ELK3 Hs.591015	1.666156396	1.01334324	0.32702592	-2.10953873	-1.37521028	-0.94328285
IMAGE:1000892	ZNF539 Hs.434	-1.324663708	-0.81665813	-0.37761074	0.6952778	1.68287211	1.30584629
IMAGE:1461019	ZP1 Hs.172130	-1.553382763	-1.0009782	-0.36845389	1.1692127	1.82211835	1.30077776

IMAGE:841340	TAP1 Hs.352018	0.920962846	1.29095986	0.85883989	-2.27433444	-1.21108787	-0.90601162
IMAGE:221898	LRRC39 Hs.4427	-0.495449258	-1.38781545	-1.86509145	1.87655025	2.21002159	1.06968953
IMAGE:756490	BCAT2 Hs.51267	-1.475381603	-0.56297131	-0.45475705	0.72177411	1.53009393	1.37410763
IMAGE:487764	Hs.592414 Hom	-1.231063505	-1.7696967	1.53453817	0.80880804	0.91866312	0.85549645
IMAGE:1941687	PIP5K1B Hs.534	-1.265372517	-1.40200192	-1.16227372	2.30297866	1.20197008	1.94895508
IMAGE:300276	Hs.594923 Tran	0.719708968	1.51177675	1.14558889	-1.13332787	-1.99427112	-1.65161571
IMAGE:1917430	CD33 Hs.83731	0.66954106	1.54029009	1.04416564	-1.30913485	-1.52175961	-1.78905931
IMAGE:1912458	IL12A Hs.673 In	0.369296151	0.84107264	1.84752915	-1.36847944	-1.45431655	-1.30216863
IMAGE:924152	Hs.600053 Tran	-1.626213179	-0.96203165	-0.67220698	1.4018508	1.51712198	1.80365318
IMAGE:506497	EHD2 Hs.63155	0.767734106	0.81003127	1.66631393	-1.9127314	-1.44041303	-1.09639606
IMAGE:43679	Hs.107384 MRN	-0.638811064	-1.81611047	-0.03522428	1.3356148	1.29706748	1.09373037
IMAGE:455263	PET112L Hs.119	1.052900248	1.37883576	0.45645778	-1.90725778	-1.32760002	-0.98331843
IMAGE:78845	CSF1R Hs.48382	0.979968047	1.23009517	0.97626246	-2.00928269	-1.37006893	-1.15607128
IMAGE:143039	Hs.356055 CDN	-1.28091493	-1.34572047	-0.47433526	1.20736838	1.97755725	1.34794654
IMAGE:853962	AA644695::AI	-1.513826803	-0.97645025	-0.83538891	2.66192486	0.73744508	1.38028178
IMAGE:138496	MFAP5 Hs.51284	1.540264699	0.57988048	1.08177186	-1.99285076	-1.46623315	-1.0733487
IMAGE:768329	MYCBP Hs.5915	1.552168586	0.66782767	0.54303946	-1.63390991	-1.2519052	-1.1229786
IMAGE:323238	CXCL1 Hs.789 C	0.398467617	1.57945762	0.29587931	-1.36601037	-1.08799697	-0.88272966
IMAGE:141845	FEZ2 Hs.258563	-1.130497923	-1.3591793	-0.4893431	1.07013685	1.8006468	1.47541106
IMAGE:366585	SNX8 Hs.58490	0.646809332	1.85640285	0.15277299	-1.71057217	-1.32505248	-0.91015986
IMAGE:823688	MAN1A1 Hs.102	1.077391493	0.85560757	1.38288797	-2.29203897	-1.04858133	-1.28748827
IMAGE:213651	ENC1 Hs.10492	-1.197444492	-0.70718634	-0.91140853	0.61885266	1.72895949	1.64839477
IMAGE:626716	ELL2 Hs.592742	-1.549356994	-0.90965488	-0.80928783	1.16193217	1.93122911	1.60696632
IMAGE:1435862	CD99 Hs.49560	1.091402193	0.5689498	1.63375754	-1.73326155	-1.35746901	-1.44199434
IMAGE:212640	ARHGAP4 Hs.31	0.740053221	1.85755007	0.34843486	-1.2172969	-1.55839715	-1.55625446
IMAGE:756509	NSDHL Hs.5769	-1.192034903	-0.96446271	-0.42568555	0.74039435	1.69278397	1.33367505
IMAGE:251047	MMP17 Hs.1595	1.590711721	1.05650063	0.37714539	-1.97161953	-1.30637552	-1.16425521
IMAGE:1635756	HSPC049 Hs.45	-1.044517733	-1.13127386	-0.75355102	1.64542283	1.36124089	1.19896244
IMAGE:161993	CEBPB Hs.5171	1.267686917	0.78059151	0.91306597	-2.3388769	-1.06424603	-0.81062717
IMAGE:897011	PLEKHM2 Hs.14	1.091751553	0.3131365	1.43203517	-2.6347866	-0.5512704	-0.71131905
IMAGE:110198	TRPV2 Hs.27974	0.79131599	1.4750353	0.64496074	-1.2748294	-1.42804126	-1.5028572
IMAGE:814432	CCDC34 Hs.143	-1.236354508	-0.72706349	-1.00100032	0.71192479	1.80646642	1.67798619
IMAGE:823925	PLEKHO1 Hs.43	0.558660318	1.93460429	0.13027364	-1.86626362	-1.49508973	-0.54138561
IMAGE:151477	VGLL3 Hs.43501	1.218264816	0.83901337	0.99342005	-1.00541017	-1.79485408	-1.52742809
IMAGE:345553	CLEC3B Hs.4760	1.524784341	-0.4188968	1.26130756	-0.76929931	-1.38493332	-1.08123314
IMAGE:1855450	SHROOM2 Hs.5	-1.364915043	-0.95088054	-0.55589895	0.95168915	1.69692509	1.51995282

IMAGE:48614	ELF4 Hs.271940	1.070784517	1.4165186	0.45257906	-1.62321452	-1.46960638	-1.2038576
IMAGE:503035	CPXM2 Hs.3075	1.19981162	1.44973007	1.02992748	-2.31679008	-1.55797425	-1.38695755
IMAGE:132690	PDCD4 Hs.2325	-1.502909386	-0.98157927	-0.51097316	0.87665733	1.68830022	1.80049187
IMAGE:139009	FN1 Hs.203717	1.522819903	0.56782203	0.54478317	-0.2925586	-1.92710992	-1.59727335
IMAGE:970284	LOC643980 Hs.5	-0.838349326	-0.88992869	-0.86770989	2.71328648	0.47222935	0.49153855
IMAGE:740117	IRF7 Hs.166120	0.9381888	1.23466222	0.53410651	-2.26207702	-1.0926541	-0.57217855
IMAGE:384968	Hs.59203 Trans	-1.260283514	-1.52186541	-0.10361595	1.51795398	1.28253028	1.50225906
IMAGE:1456776	NCF1 Hs.520943	1.419100608	0.6776186	0.91089384	-1.90652951	-1.350418	-1.02674861
IMAGE:154670	GABRP Hs.2622	-1.397319711	-1.24663028	-0.14910892	1.79211628	1.15247104	1.20772383
IMAGE:340864	LY6G6C Hs.2415	1.235999131	0.34240879	1.16408255	-1.25786929	-1.672463	-0.89238279
IMAGE:195117	Hs.445447 Tran	1.180352541	1.35968884	0.10379336	-1.02531474	-1.33492363	-1.57956541
IMAGE:564514	SEMA6D Hs.511	2.048123078	-0.56805005	1.1600524	-2.2451923	-0.91113876	-0.51384398
IMAGE:743901	ZNF256 Hs.2887	-0.815153198	-1.77648845	-0.42121473	1.12789343	1.8475887	1.43849875
IMAGE:137238	ANXA11 Hs.530	0.441718096	1.48332409	1.23546044	-1.68175967	-1.46475842	-1.28537073
IMAGE:310519	F10 Hs.361463	1.065400606	0.50644502	1.28620197	-1.44392065	-1.22701251	-1.29458774
IMAGE:486356	ZNF83 Hs.4672	-1.321227932	-1.18555476	-0.64587705	1.19513246	1.81508346	1.55730444
IMAGE:85450	ACOX2 Hs.4449	-1.458212929	-0.68629295	-0.31753335	0.87743934	1.53814237	1.19809379
IMAGE:951705	ZCCHC6 Hs.136	-1.401032359	-1.12903928	-0.70405008	2.1442275	1.12425344	1.40668911
IMAGE:838689	SLC44A1 Hs.573	1.637837066	0.62994032	0.61079591	-2.57590332	-1.16541095	-0.4238467
IMAGE:491762	Hs.594584 Tran	1.568622328	0.36054062	0.9744308	-1.27594841	-1.27017732	-1.56565718
IMAGE:1896493	LOC125893 Hs.4	-0.965640784	-1.43893394	-0.86200172	1.51156344	2.34023099	0.8325698
IMAGE:759173	PYGM Hs.15408	1.175921443	0.24045865	1.34003772	-1.50696945	-1.26900084	-1.023647
IMAGE:455121	CEBPG Hs.4296	1.946958318	0.82178347	0.15185178	-2.37494174	-1.25946667	-0.70851899
IMAGE:2012135	C3AR1 Hs.5911	1.336054788	0.85645983	0.61204402	-0.70790789	-1.64578248	-1.70013658
IMAGE:769901	CDIPT Hs.12154	1.091097092	0.27577025	1.72093708	-2.04969479	-1.46877441	-0.68300316
IMAGE:141627	FAM46A Hs.107	0.99495502	0.82949728	0.87486472	-1.97487805	-1.18628509	-0.66909621
IMAGE:262804	GABARAP Hs.51	1.058830634	1.19395864	0.76655376	-2.43372792	-0.94731983	-0.95632836
IMAGE:1473289	PPGB Hs.51707	1.497657094	0.81197499	0.63066039	-2.16222868	-1.02118922	-1.06935571
IMAGE:785389	CTPS Hs.473087	1.104862943	1.45032352	0.32447636	-1.86189046	-1.0048825	-1.37160219
IMAGE:145503	CDKN2D Hs.435	1.207088227	1.23297823	0.27874686	-1.81408966	-0.75280006	-1.44164354
IMAGE:783996	TSEN34 Hs.155	1.131736	1.06343617	1.11010772	-2.19133521	-1.54248685	-0.94657085
IMAGE:840530	ZNF664 Hs.5248	-1.505758931	-0.71745091	-0.92930869	1.21874855	1.75414108	1.52356099
IMAGE:1877404	LOC642775 Hs.	-1.06713034	-1.12759717	-0.51947362	1.76383308	1.16193617	1.01566403
IMAGE:365517	MPP7 Hs.49915	1.88949175	0.4913543	0.6265559	-1.7543301	-1.40404311	-1.19609074
IMAGE:770192	LGALS9 Hs.813	0.692571082	1.78640351	0.49298413	-1.66666063	-1.14971243	-1.51831897
IMAGE:141115	CSF2RB Hs.592	0.803444985	1.45817097	0.1432977	-1.3532933	-1.13018604	-1.08806672

IMAGE:759164	NEK4 Hs.63192	0.869720284	1.34203341	0.92566274	-2.41726662	-1.2098358	-0.84760655
IMAGE:245860	Hs.593064 Trar	-0.837631943	-0.95977149	-1.12533843	0.9830955	1.23682587	1.88285683
IMAGE:202740	PEX11A Hs.3103	-1.401164036	-0.76723803	-0.4462755	1.12291634	1.40038395	1.28714717
IMAGE:62260	LOC440341 Hs.5	-0.977592585	-1.06715034	-0.76346814	2.39636004	0.82842091	0.79666861
IMAGE:789382	NOTCH4 Hs.436	-1.316615218	-1.52901386	0.1000837	0.70462333	1.94715537	1.49156029
IMAGE:129986	R19275 10699	-1.136762911	-0.87650199	-0.99070604	0.95950285	1.69949846	1.59927858
IMAGE:342720	SNRK Hs.47605	1.495279416	0.83743918	0.65669488	-1.59700108	-1.5128838	-1.21006161
IMAGE:487766	MS4A6A Hs.523	0.916146628	1.27558816	0.47461987	-1.31149861	-1.26858656	-1.30079184
IMAGE:773437	PPIG Hs.470544	-0.709604623	-0.18252554	-2.13467031	1.07978008	1.63614133	1.29061171
IMAGE:857319	EIF3S4 Hs.5290	1.04032702	0.99109452	0.87016421	-2.531897	-1.05492206	-0.54801852
IMAGE:433253	FBP1 Hs.494496	-1.271634813	-0.53169362	-0.70592091	0.75428219	1.47323214	1.35987945
IMAGE:1573665	SH3RF1 Hs.3018	-1.294387967	-1.61096287	-0.36756444	1.48945044	1.23136332	2.09666804
IMAGE:325079	SRA1 Hs.642625	1.105487771	1.27631643	0.40598935	-2.31490665	-1.0741511	-0.69113525
IMAGE:1699118	LOC644488 Hs.1	-0.887940641	-0.89233776	-0.61004293	2.24093691	0.60067537	0.59135898
IMAGE:1631649	CSNK2B Hs.735	1.588255128	0.33731847	0.77358819	-2.31079424	-1.06208345	-0.48246796
IMAGE:266643	Hs.556072 Full-	-1.210049075	-1.70917146	0.25612293	1.24578904	1.76986754	1.04302057
IMAGE:384866	Hs.206112 Trar	1.601299576	0.76333336	0.94981035	-1.85663882	-1.44773688	-1.42983666
IMAGE:200402	FAM83D Hs.472	0.587395997	1.92533415	-0.22549183	-1.15138119	-0.93984232	-1.39600691
IMAGE:28298	Hs.106554 Trar	-0.922687589	-1.17848485	-1.14146883	1.8053653	1.30498609	1.4682433
IMAGE:341840	ADAMTS14 Hs.3	0.652611923	1.71274789	0.22444848	-0.53197703	-1.89027624	-1.40634706
IMAGE:770835	BCKDHB Hs.436	-1.185042379	-0.91908391	-0.71439201	0.92594005	1.44938843	1.67385097
IMAGE:1506912	ARL1 Hs.372616	-1.142264581	-1.69141797	-0.35987526	1.7124075	1.41623489	1.57172551
IMAGE:1441751	SYTL2 Hs.36952	1.058189941	1.29022697	0.37429132	-2.1561336	-1.23103858	-0.60331734
IMAGE:814620	FNBP1 Hs.18940	1.032244974	1.31649694	0.72197673	-1.96131363	-1.5060287	-0.95824145
IMAGE:204539	CCR2 Hs.395 Ch	0.942199048	1.35026016	0.55046038	-1.39652341	-1.17031561	-1.55992527
IMAGE:840770	LTBP2 Hs.51277	-0.918713097	-1.14399259	-0.61683857	0.91534362	1.5634915	1.38627163
IMAGE:1017720	AA552851 644	0.690707791	0.41065935	1.62144271	-1.03122487	-1.32162293	-1.3260063
IMAGE:565083	RGS16 Hs.4132	0.775823434	1.17860397	0.7714094	-1.76734945	-1.26663255	-0.86192087
IMAGE:1571901	TBC1D2B Hs.56	1.022916713	0.89760977	1.03795355	-1.9522373	-1.31177956	-0.91421479
IMAGE:346360	LAT Hs.632179	0.814211096	1.76106569	-0.04571914	-1.52711972	-1.23349132	-1.04515521
IMAGE:110788	LSP1 Hs.56729	1.012965031	0.51253346	1.45517229	-2.09629813	-0.80583128	-1.20508369
IMAGE:810209	C15orf20 Hs.11	-1.200803264	-0.60956171	-1.25826195	1.34382543	1.22932442	1.71522505
IMAGE:564772	MGC9850 Hs.50	0.890865011	1.40879173	0.67919061	-1.99533791	-1.27924793	-1.02388754
IMAGE:214075	TTC21B Hs.3106	-0.868908586	-1.09193102	-1.36593001	1.72930277	1.51344891	1.40592024
IMAGE:357892	KIAA1245 Hs.51	1.426511357	0.66408523	1.07723777	-1.23090429	-2.02681535	-1.22472246
IMAGE:1899312	LOC643008 Hs.5	1.752813397	-0.37900317	0.88711306	-1.12160068	-1.3707549	-0.67725109

IMAGE:784150	RAB31 Hs.9952	1.348120282	1.14185766	-0.03824774	-1.48216658	-1.19545831	-1.00953234
IMAGE:120707	T95670 1114	-1.487592614	-0.51552836	-0.4282172	1.16695771	1.3859632	0.98703204
IMAGE:25069	ZUBR1 Hs.1480	1.323764881	0.67182683	0.8637671	-2.0226843	-1.30550909	-0.74490305
IMAGE:755506	ANXA4 Hs.4229	1.441024816	1.04308516	0.38561964	-1.6120807	-1.63803329	-0.95807552
IMAGE:1592953	JAK1 Hs.207538	1.304133394	0.89276681	0.69766152	-1.91569163	-1.26338176	-0.98835381
IMAGE:128329	FADS2 Hs.5027	-1.615764556	-0.30138839	-0.13812679	0.76923049	1.18505426	1.09410316
IMAGE:770670	TNFAIP3 Hs.591	0.875247008	1.59998203	0.16470782	-1.61780413	-0.96218043	-1.33874378
IMAGE:712668	SOCS1 Hs.5064	0.4647304	1.69054317	0.415575	-1.26876548	-1.19687571	-1.28673792
IMAGE:74051	GAB1 Hs.80720	1.013627278	1.03066823	0.90693612	-2.14643133	-1.47294343	-0.58073865
IMAGE:1010094	Hs.634001 Tra	-0.953046223	-1.31507931	-0.51335966	2.6418374	0.94185595	0.46019453
IMAGE:301867	RNF36 Hs.4892	1.217714359	1.63933021	0.34095313	-2.247597	-1.79815878	-0.6660025
IMAGE:126519	KLF3 Hs.298658	1.651908381	0.83111058	0.94573243	-1.51962532	-1.69748681	-1.68958185
IMAGE:162077	PHLDA1 Hs.484	-1.400617282	-0.36955164	-1.03822306	0.97691866	1.42771408	1.54839947
IMAGE:447173	AA702986 21	-1.607174214	-0.90203625	-0.80174997	1.9355956	1.69819915	1.13220841
IMAGE:1573251	MGC48595 Hs.5	-1.494211402	-0.53761656	-0.24676002	0.79814211	1.40409324	1.15395661
IMAGE:487371	FBXO32 Hs.403	-1.375338085	-0.94545708	-0.26505921	0.38921227	1.76779619	1.6555083
IMAGE:469647	HBA1 Hs.44963	0.201869321	0.81653058	1.45512555	-1.28827627	-0.51375473	-1.54447579
IMAGE:813585	RBCK1 Hs.2472	0.482744079	1.04789204	1.71900523	-2.2753938	-1.15883413	-1.01048279
IMAGE:1586060	Hs.128330 Tra	0.971892252	0.96581627	1.35346413	-2.40240616	-1.42315987	-0.77282691
IMAGE:125134	CD48 Hs.24356	0.888737497	1.54313577	0.16217855	-0.85815215	-1.65934787	-1.33303306
IMAGE:470061	SIAH2 Hs.4779	1.61292363	0.49875784	0.81107518	-1.67408477	-1.47669319	-1.03058823
IMAGE:1325605	D4S234E Hs.51	-0.610602258	-1.59911108	-0.48644825	1.42596207	1.20835518	1.28831306
IMAGE:195162	RHOC Hs.50265	1.416449436	0.9584877	0.8685368	-1.55045319	-1.74086022	-1.35676329
IMAGE:1643107	RPL10L Hs.308	0.760133073	1.08072824	1.03146247	-2.04139811	-1.19707837	-0.81214358
IMAGE:36493	NAGA Hs.75372	0.868162055	1.60945556	0.79937002	-1.46454035	-1.55349557	-1.69760303
IMAGE:811048	PLXND1 Hs.301	0.328665508	1.43092561	0.91058075	-1.98001216	-1.00472757	-0.79287288
IMAGE:685381	AA243581::A	-1.135667256	-1.31179386	-0.70263418	2.58980767	1.47742912	0.48224761
IMAGE:810761	CLDN10 Hs.534	-1.448456538	-0.51358562	-0.44002119	0.98847104	1.22380748	1.2808112
IMAGE:811013	AMPD2 Hs.8292	1.035524616	1.27015811	0.7405568	-2.0499058	-1.10897761	-1.22533667
IMAGE:327165	SERPINB5 Hs.5	1.746857142	-0.11846905	0.70684981	-1.4642315	-1.21948076	-0.64243213
IMAGE:810575	ZNRD1 Hs.5781	1.065027724	0.77152669	1.15394127	-2.1498876	-0.90617699	-1.14119362
IMAGE:111120	JOSD1 Hs.3094	1.359786804	1.01453405	0.72404552	-2.4698401	-1.30014757	-0.69655051
IMAGE:253979	TRAF3IP1 Hs.63	-1.509682333	-1.53789807	-0.81933736	1.54216286	1.65189072	2.40148873
IMAGE:128795	ZNF138 Hs.184	-1.033437324	-0.8881444	-1.2658011	2.00801048	1.67756086	0.77905261
IMAGE:1941993	GPR55 Hs.1145	1.287291524	0.99924467	0.73651102	-1.43686068	-1.25864987	-1.65493252
IMAGE:154472	FGFR1 Hs.2648	0.691864448	0.51902823	1.79978892	-1.72939369	-1.51667219	-0.82000928

IMAGE:82734	ACSL1 Hs.4066	-1.258168953	-0.70370194	-0.68538473	1.00957637	1.60290557	1.18705532
IMAGE:248371	COPA Hs.16212	-0.516320031	-1.39875405	-1.1291946	1.2985965	1.51152212	1.47398575
IMAGE:1580874	CORO2A Hs.113	1.374386057	0.57459177	0.69378668	-0.39247152	-1.39068394	-2.00754464
IMAGE:586904	RALGPS1 Hs.43	-1.116906402	-1.1668927	-0.62120898	1.95995829	1.1976673	1.04458428
IMAGE:769686	THY1 Hs.64351	0.67341314	1.50783793	0.72072589	-1.8980346	-1.15608472	-1.11866464
IMAGE:263341	C2orf32 Hs.212	1.057119353	0.56577433	1.71609974	-1.69932624	-1.61261829	-1.26752067
IMAGE:469383	C8orf1 Hs.4364	-1.101024729	-1.19989767	-0.78552007	1.6620613	2.17983492	0.59138745
IMAGE:809616	KLK10 Hs.2754	1.631106813	0.28803884	0.62307628	-1.55869699	-1.37357724	-0.72528961
IMAGE:359395	NAPG Hs.4646	0.863375033	0.70045177	1.52192802	-1.15215726	-1.42483936	-1.67115361
IMAGE:248027	CCNI Hs.64272	-1.101910399	-1.13043097	-0.5866387	0.28680387	2.06220959	1.73279697
IMAGE:509610	SGK2 Hs.4727	-1.374648686	-0.72622521	-0.28546676	0.8446517	1.35353307	1.30995951
IMAGE:1455463	Hs.560695 Tra	-1.460301397	-1.54216566	0.12534631	0.83773403	1.71485108	1.79443258
IMAGE:357450	FAM89B Hs.257	0.945192927	0.93740295	0.95921489	-2.13321155	-1.28605146	-0.60364941
IMAGE:1984409	COPG2 Hs.5322	-1.014818768	-1.11616086	-0.74014675	1.42315752	1.57517066	1.1233247
IMAGE:510130	CDH17 Hs.5918	-1.35622333	-0.91720008	-1.10711586	2.25130279	0.99713786	1.54558929
IMAGE:809824	TMEPAI Hs.5171	-0.996694957	-1.24112625	-0.44742905	1.1249258	1.14789867	1.64319366
IMAGE:2017403	RGS3 Hs.4948	0.971871213	1.37586772	0.93881695	-2.15818352	-1.51546181	-1.02148426
IMAGE:841059	CAPG Hs.5161	1.213013457	0.3539181	1.13736178	-1.91354868	-1.14420956	-0.71434132
IMAGE:795965	CKMT2 Hs.8069	-1.22332555	-0.98734511	-0.40007561	1.25353049	1.0524108	1.51015922
IMAGE:687875	CTSS Hs.18130	0.857690442	1.37753828	0.48449441	-1.58838517	-1.11559724	-1.25447869
IMAGE:60738	EPHX1 Hs.8964	-1.589141801	-1.10353755	-0.4160788	1.65581345	1.28382444	1.61947964
IMAGE:950592	FRAP1 Hs.3382	-1.386841561	-0.45561086	-0.88190758	0.46465201	2.18556634	1.21584477
*mitoch. cont. IMA	149425	-1.320980028	-0.90140601	-0.70970767	1.26819503	1.13979219	1.81272643
IMAGE:809682	TNRC5 Hs.4140	0.551878854	1.36301778	1.32246084	-2.18183826	-1.45718106	-0.88640168
IMAGE:264868	APH1B Hs.5117	0.848069052	1.44642496	0.87956147	-1.67841005	-1.41899127	-1.44379155
IMAGE:713974	CSF1R Hs.4838	0.922821441	0.93621575	0.90674828	-1.46243643	-1.37644499	-1.08310971
IMAGE:731292	KLF6 Hs.4055 K	1.277718135	0.98985755	0.56958109	-2.51278894	-0.8342019	-0.76634905
IMAGE:201562	Hs.165062 CD	-1.209336772	-0.84718773	-0.49177633	1.09652095	1.33023342	1.27275279
IMAGE:825092	CTH Hs.19904 *	1.595582315	0.56673996	0.38321673	-1.74763398	-1.36330773	-0.61156261
IMAGE:1553313	Hs.127009 Tra	1.491042902	-0.0662243	1.34457735	-0.94012832	-1.72027868	-1.15754259
IMAGE:1636876	TUBB2C Hs.433	1.390958764	0.77441177	0.61202545	-2.22951807	-0.98556938	-0.79800016
IMAGE:2010309	Hs.604782 Tra	1.548558315	0.50829693	0.74505745	-1.17449703	-0.93474607	-1.90736159
IMAGE:1686766	RRAGD Hs.4859	-1.467588493	-0.92875236	-0.27716707	1.1482741	0.90396473	1.8887313
IMAGE:649743	SC5DL Hs.2877	-1.069191308	-0.80318781	-0.89223865	0.95930084	1.61617054	1.3483956
IMAGE:1606829	HDAC4 Hs.2051	1.553550636	0.48506485	1.14548	-1.78461493	-1.63968734	-1.06547095
IMAGE:78041	IL6ST Hs.53208	1.219536209	0.65232175	1.0384697	-2.26797863	-0.97931159	-0.85858384

IMAGE:84211	SOCS3 Hs.5279	1.057900387	1.24697874	-0.10342945	-1.24611652	-0.98635468	-1.09556068
IMAGE:1635970	Hs.592881 CDN	1.345309149	0.88167107	0.40703142	-2.1962178	-0.8406658	-0.81237601
IMAGE:1534173	FREQ Hs.30176	1.411853014	0.87174731	0.34933472	-0.25898261	-1.37318344	-2.22990283
IMAGE:290234	ITGAX Hs.24847	0.703196562	1.24875489	0.50386536	-1.07006077	-0.86764514	-1.62005296
IMAGE:322192	FLJ20273 Hs.51	-0.943733266	-0.6245386	-1.34161313	0.82671015	1.69760257	1.50511149
IMAGE:1656062	F12 Hs.1321 Co	1.627666025	0.58572467	0.53216302	-1.833546	-1.25971109	-0.89203273
IMAGE:285992	ADNP Hs.57035	1.047204722	0.69854009	1.12360745	-1.5083165	-1.68829521	-0.8265148
*mitoch. cont. IMAG	148100	-0.972830054	-1.09117401	-0.49816614	0.82515273	1.62868403	1.264877
IMAGE:270826	Hs.592775 CDN	-1.401593947	-0.98438957	-0.24744536	0.93124467	1.56844548	1.38859182
IMAGE:462237	C3orf52 Hs.434	1.124491947	0.652841	1.23161267	-1.47898133	-1.3710612	-1.35547273
IMAGE:502063	FLJ39575 Hs.39	-0.761020163	-1.39668232	-0.8881526	1.36617784	2.03967704	0.94088959
IMAGE:276523	FBN1 Hs.59113	0.973866776	0.75582714	1.11922653	-1.01803587	-1.49677507	-1.4787631
IMAGE:841332	ARHGDIB Hs.50	0.508245965	1.67369538	0.31702699	-1.40251201	-1.33569673	-0.93098702
IMAGE:1469115	AP2A2 Hs.1912	0.728394373	1.19485293	1.47168881	-2.28046151	-1.10902947	-1.33499098
IMAGE:1609752	TOMM40 Hs.110	-1.50888565	-0.48231564	-0.13234859	0.70983682	1.31713258	1.12526829
IMAGE:131012	NAV2 Hs.50211	-1.334587541	-1.18089398	-0.12828048	1.12788327	1.31062867	1.49506094
IMAGE:433490	CSNK1A1 Hs.52	1.74895675	0.95875117	-0.64456449	-0.88448923	-1.1307127	-1.24065435
IMAGE:2107532	ITGB7 Hs.52445	0.825958661	1.63989312	0.20327656	-1.91095154	-1.15038984	-0.89153199
IMAGE:897509	Hs.173030 **C	0.522765133	1.42785319	0.98806601	-1.79822572	-1.12921156	-1.23357272
IMAGE:731469	PLEKHB1 Hs.44	-1.696465287	-0.53265846	-0.33143251	1.57589671	0.73119821	1.45088133
IMAGE:844680	TRA@ Hs.74647	0.616756851	1.59241793	0.19406047	-0.93821989	-1.24632339	-1.37179449
IMAGE:756731	MAP3K3 Hs.292	0.831325023	1.28202878	1.31583156	-2.37184066	-1.51620775	-0.92677174
IMAGE:489495	Hs.579591 **T	1.34548201	0.27814413	1.14359234	-1.63890324	-1.42181757	-0.80420884
IMAGE:85502	UBE2R2 Hs.111	1.48295083	0.78220061	0.49862215	-1.89929595	-1.18010492	-0.94160397
IMAGE:510534	Hs.692 **Trans	-1.284803935	-0.8469424	-0.46962468	1.13870563	1.09045977	1.55548494
IMAGE:1211795	RPLP0 Hs.44822	0.963045289	0.80195727	0.8431958	-2.18735313	-1.0420173	-0.47212816
IMAGE:1899317	HIG2 Hs.433213	-1.061219343	-0.74118384	-1.17608357	1.31500133	1.72899115	1.12971676
IMAGE:428163	SLC9A9 Hs.302	1.068165931	0.3174459	1.79908436	-1.47421818	-1.37720484	-1.47585018
IMAGE:140071	FRZB Hs.12845	-1.330367978	-1.17198643	0.13712207	1.25876767	1.05027671	1.27308465
IMAGE:80463	T65992::T645	1.236200247	0.90200018	0.64770083	-1.8881862	-1.38112404	-0.74761645
IMAGE:399463	AA732944 55	1.579979133	0.37841616	1.27029795	-2.00974186	-1.2766749	-1.23904863
IMAGE:83345	GNE Hs.5920 Gl	-1.584971791	-0.67753454	-1.00075066	1.82611072	1.30508925	1.51349784
IMAGE:1625160	Hs.88045 Trans	-1.639533398	-0.84193345	-0.64196537	1.43980459	1.47632473	1.60852766
*mitoch. cont. IMAG	140317	-1.338853226	-1.03932142	-0.02780518	1.14792341	1.22346904	1.230626
IMAGE:448009	GJB7 Hs.14672	-1.342638425	-0.56888613	-1.01312577	0.62563468	1.1100219	2.39803746
IMAGE:824523	JAK3 Hs.515247	0.480043164	1.61319812	0.24264464	-1.55613814	-0.93178587	-0.95524372

IMAGE:898218	IGFBP3 Hs.4502	1.367100789	0.59401378	0.44439749	-0.89206886	-1.63324779	-0.97185206
IMAGE:1010136	AA229609::AA	-1.87767513	-1.21303644	0.04156245	1.65573834	0.98363179	1.94474415
IMAGE:681906	FGR Hs.1422 Ga	0.876368623	1.57119999	0.37609004	-1.57980423	-1.18728054	-1.3743807
IMAGE:1871243	Hs.146276 **F	-1.159935732	-0.90814005	-0.41205838	0.89893572	1.60793936	1.11031157
IMAGE:2062432	COMP Hs.1584 C	1.864992613	-0.42846325	0.39158182	-0.92990099	-0.93636143	-0.77800889
IMAGE:1750217	FCER2 Hs.46577	1.217216801	0.70869332	0.61970609	-0.37935074	-1.9358515	-1.34829555
IMAGE:138672	UNQ338 Hs.208	1.290341847	0.70442855	1.36015279	-1.96552847	-1.35779925	-1.36901887
IMAGE:235040	C13orf1 Hs.442	-1.025350627	-1.67681517	-0.18496137	1.58295052	1.21952637	1.48197352
IMAGE:276688	Hs.27688 Full-l	-1.352134113	-0.95626769	-0.4457885	1.09526295	1.29319617	1.6313792
IMAGE:68767	FCGBP Hs.1117	1.511690373	0.8349148	0.61848119	-2.31262428	-0.98654787	-0.99383709
IMAGE:1882648	TMEM25 Hs.564	-1.035093971	-1.30083279	-0.5711993	2.32889695	0.87835672	1.01063559
IMAGE:814988	DYNLL1 Hs.5120	1.684145968	0.96863853	0.79356757	-1.12345954	-1.87702588	-1.97065079
IMAGE:1708311	TARDBP Hs.3006	-0.944215357	-0.83921027	-1.24662999	1.17611727	1.41902903	1.63827964
IMAGE:986259	Hs.136418 Tran	-0.901355549	-1.96721077	0.02070382	1.67277941	1.27702548	1.32716482
IMAGE:727305	UXT Hs.172791	1.441788773	0.49452224	0.84092671	-1.99381651	-1.32666717	-0.63514122
IMAGE:244044	C1QC Hs.46775	1.094639414	0.72707356	0.88429516	-1.04864511	-1.28060957	-1.50868373
IMAGE:435371	Hs.633766 Tran	-1.012968966	-0.92597223	-0.65389655	0.82230679	1.38161028	1.52186541
IMAGE:724888	CYP4B1 Hs.4363	0.459832912	1.21732821	1.0748932	-1.60013347	-1.36220226	-0.89702245
IMAGE:810205	Hs.264606 Full	-1.364055708	-0.71125399	-0.76259484	0.85768821	1.34714163	1.86137825
IMAGE:206795	ASGR2 Hs.1624	0.624910174	1.37526482	0.55391918	-1.57652924	-1.02972825	-1.08640397
IMAGE:262060	LOX Hs.102267	1.313180882	0.47527685	1.2964228	-1.55220448	-1.55220448	-1.19880613
IMAGE:49630	CACNA1D Hs.47	-1.227484496	-0.9719762	-0.73435349	1.47250814	1.10466745	1.63995732
IMAGE:1700097	ACAD8 Hs.1479	-1.318080291	-0.92010862	-0.24117576	0.4358863	1.77266293	1.45020384
IMAGE:1869574	C2orf18 Hs.516	1.171418699	1.30053874	0.96210832	-1.53814831	-1.77693544	-1.59548781
IMAGE:148469	TYROBP Hs.515	0.751807218	0.72777299	1.21903261	-1.16576364	-1.12250203	-1.4548954
IMAGE:704532	NMI Hs.54483 N	0.899822079	1.32841304	0.24899876	-1.4927473	-1.11177755	-1.04907628
IMAGE:111750	Hs.445588 Tran	-1.446633746	-0.50162507	-0.44070559	0.75566689	1.64693352	1.07066978
IMAGE:1879740	C1orf45 Hs.149	1.227015355	0.19433218	1.09477584	-0.7934423	-1.51088008	-1.19616871
IMAGE:320254	BRP44L Hs.1727	-1.099483778	-1.00657143	-0.63359471	0.98345347	1.66847466	1.29914807
IMAGE:840942	HLA-DPB1 Hs.48	0.66909811	0.94510405	1.15838138	-1.62253534	-0.79828122	-1.44846341
IMAGE:209143	ALDH6A1 Hs.29	-1.372417646	-0.76411276	-0.48852712	0.77467176	1.73514653	1.30563622
IMAGE:175123	MAPK7 Hs.1501	1.5844017	0.61400906	0.98505889	-1.48811507	-1.24920381	-1.79162086
IMAGE:381023	ZNF540 Hs.1212	-1.55602374	-1.01939931	-0.55512873	1.82440873	1.55217734	1.18045941
IMAGE:207255	ABCF3 Hs.3613	1.293291446	1.05399204	0.71674683	-2.1590199	-1.28251626	-0.9753226
IMAGE:595001	CRISPLD1 Hs.43	-0.989952256	-1.05026139	-0.64722309	1.62461545	0.93389256	1.31084133
IMAGE:230376	CFB Hs.69771 C	0.208626282	1.27268982	0.86862693	-1.94141489	-0.70039805	-0.66594489

IMAGE:1947381	GLT25D1 Hs.418	1.146539384	1.07672044	0.62894829	-2.17847138	-1.10325967	-0.83934406
*mitoch. cont. IMAG	140615	-0.945120151	-0.92760361	-0.97104463	0.98222502	1.69689995	1.34376646
IMAGE:2490258	SLC10A3 Hs.522	1.198574256	0.46841739	1.18396013	-2.15267545	-0.87366478	-0.95409741
IMAGE:796613	Hs.634838 Tran	1.031291127	0.73766841	1.02994114	-1.81060555	-1.16812304	-0.96213714
IMAGE:2029006	Hs.442676 Tran	-0.986757949	-0.93220305	-0.56862104	1.12266883	1.36024662	1.10630236
IMAGE:199362	Hs.596145 Tran	-1.809486574	-0.51971387	-1.04009615	1.83728983	1.85965583	1.09697521
IMAGE:815276	NUP62 Hs.5744	1.109013439	1.57038776	0.27134091	-2.39775864	-1.18051574	-0.78000356
IMAGE:47475	CYFIP2 Hs.5197	0.546683486	1.82936946	0.16622729	-1.52758395	-1.46100856	-0.78327102
IMAGE:1435137	C17orf27 Hs.19	0.49515656	1.65497441	0.10319172	-2.13204141	-0.73107331	-0.49107139
IMAGE:248391	ZNF710 Hs.459	-1.088603338	-1.02577332	-0.59740686	0.88647084	1.50156679	1.53028594
IMAGE:2351095	TMPRSS2 Hs.43	-1.361016462	-1.30181669	-0.0635547	1.08763428	1.16558065	1.82047817
IMAGE:136772	AMMECR1 Hs.48	1.419637402	0.7739265	0.41124181	-1.89977183	-1.11603518	-0.7885911
IMAGE:156437	ATP1A2 Hs.3411	-1.286722467	-1.27871365	0.45383688	0.92156911	1.4527625	0.90652647
IMAGE:745397	DDA1 Hs.64260	1.100904726	1.16866393	0.73210104	-1.23316661	-1.87493446	-1.21137822
IMAGE:1526847	C18orf49 Hs.53	-1.17906775	-1.09874057	-0.56858121	1.64354756	1.17547157	1.30841986
IMAGE:1519744	Hs.129798 Tran	-0.901593377	-1.16197055	-0.67400444	1.32426983	1.50496677	1.10861485
IMAGE:595420	ECHDC1 Hs.486	-1.341286431	-0.78702627	-0.67701402	0.71682432	1.6804812	1.64143105
IMAGE:1516913	Hs.532593 **T	-1.296908053	-1.26099474	-0.8894117	1.79989666	1.32224964	1.82647251
IMAGE:283744	LOC145783 Hs.6	-1.415741659	-1.05416311	-0.13963933	0.95108048	1.26859185	1.65973398
IMAGE:1586103	GPR143 Hs.741	0.542632789	1.21323931	1.02630774	-2.16943172	-0.93966958	-0.80759153
IMAGE:232614	Hs.601468 **T	-0.883281794	-1.31296583	-0.24348199	1.24912508	1.10589064	1.24370821
IMAGE:841238	Hs.362807 MRN	0.729650247	1.40538102	-0.01715081	-0.95574974	-1.11579124	-1.10956741
IMAGE:626585	SYNJ2 Hs.43449	-1.315929659	-0.23215627	-1.64792856	1.35707949	1.53621558	1.48874452
IMAGE:45607	IGSF4B Hs.3656	0.517100858	0.01931534	2.11246266	-1.10575135	-1.41358613	-0.92586513
IMAGE:1416278	AA825324::AI	-1.143519852	-0.92826335	-0.78306248	1.90079212	1.39633867	0.78937211
IMAGE:841195	FRAS1 Hs.3694	1.28938404	1.3067472	-0.13555237	-1.43605282	-1.22769494	-1.06100863
IMAGE:490232	IRAK1 Hs.52281	-1.144853895	-0.95524501	-1.64473188	1.976367	2.09271791	1.13697829
IMAGE:2409635	COL6A2 Hs.420	0.845144035	0.45640177	1.49986786	-1.59932873	-1.07759568	-1.15022911
IMAGE:1607229	HDDC2 Hs.3282	-0.852746398	-1.09751992	-0.66219153	0.88343482	1.48014078	1.38956329
IMAGE:854897	Hs.633467 Tran	1.197922838	0.50643757	1.31689898	-2.05885838	-0.96906997	-1.17473599
IMAGE:235164	YIF1A Hs.44644	1.166791102	1.08250065	0.48436266	-1.85895236	-1.36050403	-0.75993458
IMAGE:263815	Hs.592775 CDN	-1.302246616	-1.02996859	-0.2330573	1.01261467	1.27232237	1.50470739
IMAGE:32273	NBEA Hs.49117	-1.182869113	-1.2415744	-0.66664132	1.99128795	1.11032988	1.36834909
IMAGE:742763	FCHO1 Hs.9648	1.025148653	0.72374463	1.07621004	-1.74782747	-1.22421185	-0.99656316
IMAGE:1584411	AA971543 13	0.616367801	1.29898084	0.62616315	-1.91159622	-0.99328272	-0.75084795
IMAGE:840788	TMSB10 Hs.446	0.691674723	1.44552855	0.32716066	-1.70415752	-1.1001662	-0.81043202

IMAGE:797000	UBIAD1 Hs.5229	-1.651596363	-0.47107059	-0.16696498	0.37097685	1.55963017	1.46209964
IMAGE:131362	MSN Hs.87752 M	0.826095678	1.17278877	0.35707348	-2.04145082	-0.95063596	-0.45258174
IMAGE:1857088	RGS20 Hs.3687	1.464703509	0.76346074	0.21235188	-0.74996481	-1.30229279	-1.55542863
IMAGE:505576	NFXL1 Hs.51861	1.017369924	0.15639983	1.55815596	-1.23481028	-1.3783053	-1.095234
IMAGE:127648	CD36 Hs.120949	-0.736432592	-0.95591822	-0.85032784	0.84178774	1.42016204	1.33948624
IMAGE:1901097	GPD1 Hs.52441	-1.290761441	-0.76708455	-0.1601023	0.82882202	1.21670008	1.24137477
IMAGE:1759582	TNFRSF12A Hs.1	1.355733592	0.83648853	0.05341083	-0.64055319	-0.80923767	-1.90530586
IMAGE:2405842	TAP2 Hs.502 Tra	0.903205477	1.42941676	0.14622203	-1.44049518	-1.16114356	-1.08007215
IMAGE:782444	Hs.469369 **C	-1.080800664	-1.58754869	-0.26317127	1.73649059	1.31799644	1.27700109
IMAGE:1098494	CALML5 Hs.180	1.30863975	0.15167973	0.96404227	-1.42040479	-1.2740301	-0.70109716
IMAGE:898328	PHC2 Hs.52427	0.500431913	1.14504872	1.25431913	-1.4719383	-1.18716703	-1.37701454
IMAGE:133199	R26462::R284	0.276057716	2.05307502	-1.04444425	-0.56512773	-0.68864438	-0.93437169
IMAGE:73252	HMGCS1 Hs.397	-1.105608126	-0.67775935	-0.70324363	1.05544098	1.24993809	1.24872669
IMAGE:108837	CCL2 Hs.303649	1.040864915	0.89189808	0.30957317	-1.79009748	-0.68300303	-0.81301045
IMAGE:180803	INPP1 Hs.32309	0.874636726	1.21687448	0.68281458	-1.92389629	-1.42828533	-0.63860341
IMAGE:1626279	TBC1D22A Hs.4	1.010007537	1.1827093	0.86493806	-2.21001679	-1.43372237	-0.72650866
IMAGE:752732	BLMH Hs.37191	1.289763035	-0.57564312	1.43280419	-0.98621928	-0.8981529	-0.97781294
IMAGE:49249	CXorf40B Hs.54	1.329712283	0.68149177	1.17531794	-2.0165788	-1.22005329	-1.24932142
IMAGE:1899496	AKR1B10 Hs.11	1.047148921	1.14856726	-0.08463992	-1.07171602	-1.25962005	-0.85643832
IMAGE:378458	Hs.634728 Tran	-1.566933359	-1.0599966	0.04224206	1.52189281	1.4172499	0.94844966
IMAGE:341774	HLA-A Hs.18124	0.672920198	1.2476947	0.6241735	-2.16584742	-0.92717199	-0.56811981
IMAGE:1917887	AI344419 946	-0.902186019	-1.64342876	-0.76086497	1.58384712	1.61056749	1.57508878
IMAGE:327202	TNFRSF1A Hs.2	0.886885195	0.61726005	1.67393382	-2.38296161	-1.05409484	-0.9115787
IMAGE:448101	AKAP13 Hs.459	1.755593702	0.73603556	0.67034591	-2.04448424	-1.42864375	-1.10224829
IMAGE:149406	DOPEY1 Hs.520	-0.735403152	-1.24933471	-0.72585871	0.93726053	1.88986939	1.05730026
IMAGE:1855734	KIAA0232 Hs.79	-1.03085633	-0.34501187	-1.77767207	1.89830266	1.15845506	1.22913466
IMAGE:345680	DIRAS3 Hs.194	-1.028316215	-0.99861294	-0.42465435	0.76907442	1.42026151	1.38187575
IMAGE:855385	TPP1 Hs.523454	1.055741002	0.79450646	1.01210768	-2.30414098	-1.21164514	-0.52471968
IMAGE:322676	DOPEY2 Hs.204	-1.076595089	-1.06789666	-0.75649304	1.29002895	1.75974391	1.11258107
IMAGE:1499940	MAP1A Hs.1943	0.369327439	0.88758689	1.86484421	-1.31536521	-1.60183953	-1.29922202
IMAGE:2017821	NTHL1 Hs.6619	0.896379051	0.94831191	1.53125825	-1.6538489	-1.75122301	-1.27603735
IMAGE:814251	SLAMF1 Hs.523	0.836659089	1.32218161	0.14489324	-1.17121959	-1.13520556	-1.11295245
IMAGE:1526978	WDFY4 Hs.2873	1.180710627	0.85712538	0.63484721	-1.06369529	-1.45573126	-1.33088648
IMAGE:384819	TEAD1 Hs.5681	0.676692402	0.77805257	1.43501663	-1.37810348	-1.24483363	-1.35295114
IMAGE:1417887	CHD3 Hs.19161	-1.46322323	-1.15643327	-0.64084858	1.81654415	1.25315691	1.66084441
IMAGE:2566714	DGAT1 Hs.5219	0.682953752	1.16248689	0.95562946	-2.3009647	-0.95239528	-0.7093378

IMAGE:448325	AGPAT3 Hs.2487	-1.278832947	-0.62744724	-0.48357271	0.79549683	1.5049937	1.16339565
IMAGE:1570786	AA932088::AI	-0.978485069	-1.27542557	-0.51173172	2.05077198	0.71724623	1.2525124
IMAGE:78921	ASPN Hs.43565	1.387742827	0.76015852	0.35502093	-1.16332258	-1.53964879	-0.96265682
IMAGE:84955	SEC24C Hs.819	1.063266701	1.14820619	0.55278036	-1.94507775	-1.16151094	-0.90159609
IMAGE:460159	TBX19 Hs.5079	1.741068556	0.45443426	0.61513641	-1.50781019	-0.99163082	-1.56273371
IMAGE:180147	AGPAT3 Hs.2487	-1.536087279	-0.35183681	-0.40099856	0.62269416	1.61020968	1.10023049
IMAGE:126320	JUP Hs.514174	1.474765718	0.41985462	1.07284459	-1.9027959	-1.34105574	-0.93913461
IMAGE:1989716	KALRN Hs.8004	0.738376286	0.88478418	1.44719245	-1.78261625	-1.51756747	-0.94354754
IMAGE:76480	RBM25 Hs.5311	-0.732329841	-0.64988309	-1.42344867	0.87412712	1.3419228	1.63658031
IMAGE:133456	WSB1 Hs.44601	-0.997442897	-0.38729306	-1.12691036	0.10049723	1.75253546	1.63270919
IMAGE:969854	CALM3 Hs.5154	-0.829146688	-1.22511629	-0.81677264	1.56585069	1.10801084	1.42849873
IMAGE:502518	LAMB2 Hs.4397	0.867230078	0.32136111	1.4986058	-1.95697383	-1.08091139	-0.61825881
IMAGE:2569029	EIF4G1 Hs.4337	1.420383161	0.70863519	0.61749673	-1.86212035	-1.32179948	-0.78147862
IMAGE:1659082	Hs.559426 CDN	-0.957874194	-0.95250761	-0.66575294	2.99600391	0.31002684	0.39173313
IMAGE:196012	GLS Hs.116448	-1.061838586	-0.77554476	-1.03517012	1.51147225	1.00473872	1.5338267
IMAGE:898652	Hs.404330 Tran	-0.972763999	-0.99064071	-0.480082	2.27060664	0.75287436	0.52172856
IMAGE:964748	SCGB2A2 Hs.46	-1.473802298	-0.47166288	-0.17388431	1.20206065	1.04608857	0.88740394
IMAGE:23548	Hs.593532 Clor	2.378073953	-0.02194527	0.25912494	-0.81017974	-1.62389985	-1.42401961
IMAGE:186623	GNA12 Hs.4873	1.205959311	1.09330947	0.52104826	-1.27616736	-1.81688661	-1.00715953
IMAGE:232860	THSD4 Hs.3870	-1.015303917	-1.19157356	-0.50016489	1.30295021	1.184238	1.44833412
IMAGE:754085	GTF2F2 Hs.5859	1.954044048	0.49528464	0.24110687	-1.22511211	-1.55664834	-1.19361617
IMAGE:194384	BTF3 Hs.591768	1.021035381	1.38423936	0.3394938	-2.24913571	-0.7309787	-1.05216496
IMAGE:269997	CTNS Hs.18766	1.125083976	1.0284888	0.92344155	-2.31219496	-1.28888982	-0.78357631
IMAGE:134172	UBE2Z Hs.5142	0.82301737	1.14994651	0.77691198	-2.08937638	-1.07715347	-0.76405595
IMAGE:205527	SSBP2 Hs.1027	1.332849775	1.630613	-0.10981302	-0.93275612	-1.03325121	-2.34192055
IMAGE:796505	Hs.12680 CDNA	0.732552398	1.18794504	0.84252338	-1.37751573	-1.51776985	-1.03861479
IMAGE:1913850	PSMB10 Hs.966	0.483583421	1.41803791	0.66075599	-1.9697334	-1.04228732	-0.66635628
IMAGE:154185	EIF4A1 Hs.1296	0.859141135	0.9724813	0.97467498	-0.89817982	-1.51679775	-1.5507998
IMAGE:36491	PCCB Hs.63788	-1.706749242	-0.5304682	-0.67801565	0.81151096	1.95588194	1.43595284
IMAGE:1704828	WBSCR17 Hs.48	-1.327170496	-1.11772183	-0.36273243	1.75830067	0.95777997	1.40467338
IMAGE:785933	SRPX Hs.15154	0.769137009	1.04729828	0.72196086	-1.4446929	-1.1042235	-1.07818761
IMAGE:345849	LOX Hs.102267	1.051243999	0.86437932	0.62416862	-0.82853785	-1.23554447	-1.58956344
IMAGE:754491	CTSS Hs.18130	0.955401501	0.97884754	0.65177531	-1.78697898	-0.88969701	-1.03941671
IMAGE:2312765	CFH Hs.363396	1.127359647	-0.36470023	1.63026376	-1.03196406	-1.20681482	-0.94303995
IMAGE:1703455	EDG2 Hs.12666	1.140437468	0.95113566	0.5552624	-0.90983397	-1.5923168	-1.32928692
IMAGE:484600	RERG Hs.19948	-0.887543711	-1.46670566	-0.22564434	0.95522129	1.25831042	1.59989777

IMAGE:282779	PAG1 Hs.26617!	1.062445505	0.9265711	0.51432236	-1.86372072	-0.93404103	-0.82866609
IMAGE:770854	PTP4A3 Hs.4366	0.50990916	1.35625355	1.05739388	-1.50743916	-1.28220234	-1.33134492
IMAGE:44310	ELL Hs.515260 I	1.725541441	0.85626048	0.54250438	-1.44495712	-1.91355389	-1.19232235
IMAGE:283151	N45230 9406	1.45460825	0.27986488	1.01153646	-0.64957768	-1.65486094	-1.56169164
IMAGE:899772	Hs.178393 **T	0.853444654	0.88914376	0.92889498	-2.05846382	-1.0847413	-0.63179622
IMAGE:80574	SUSD1 Hs.4948	0.648040079	1.8551619	0.22037406	-1.07555744	-1.06693514	-1.88777798
IMAGE:727292	CD163 Hs.5046	0.982472381	0.60589684	0.89159922	-1.13527887	-0.98042449	-1.38134948
IMAGE:1506399	Hs.540405 Trar	-0.717285093	-1.05704982	-0.53799386	2.05698101	0.92312514	0.35388854
IMAGE:324210	OPRS1 Hs.5220	1.140687278	0.90408697	0.7829797	-1.08235306	-1.65741212	-1.30612081
IMAGE:841008	GBP1 Hs.62661	0.990037842	1.49409564	-0.0271794	-1.35595463	-1.33138019	-1.00489116
IMAGE:897504	Hs.113577 Clor	-1.650283062	-1.40406639	0.67046695	0.93278241	1.38733628	1.42332179
IMAGE:223098	ROM1 Hs.28156	1.35440447	0.60656047	1.43585283	-2.16573798	-1.17308613	-1.39743933
IMAGE:182999	DUSP22 Hs.291	0.783165344	0.85456085	1.07065126	-2.45781673	-0.84631813	-0.4907685
IMAGE:487327	CGI-38 Hs.5344	1.668815509	-0.9886513	0.50963827	-0.10388196	-1.10348892	-0.44992327
IMAGE:51548	H20826::H20	1.054524023	0.97064954	1.63829044	-1.3849654	-1.63658885	-2.06406915
IMAGE:383851	MEGF10 Hs.438	-1.317905396	-0.93220331	-0.66351197	1.30907812	1.18482442	1.71065049
IMAGE:2509925	SEC13L1 Hs.166	1.205190137	1.13151745	0.46846327	-1.89169388	-1.31762099	-0.8813256
IMAGE:1471441	CTLA4 Hs.2478	0.701241929	1.44296591	0.25065155	-0.95402487	-1.33322084	-1.24238049
IMAGE:359135	FAM59B Hs.187	0.560048915	1.61802443	0.91390287	-1.47999089	-1.33384681	-1.59565092
IMAGE:809901	COL15A1 Hs.40	1.240445396	1.03235802	0.35437385	-1.88545235	-1.20765075	-0.75906933
IMAGE:784360	EML1 Hs.12451	1.074346806	0.40921196	1.48759626	-1.78666775	-1.28486483	-1.0133009
IMAGE:1585492	RNF20 Hs.3887	1.644052854	-1.23284182	1.67336977	-1.37824237	-0.53115402	-0.79913238
IMAGE:340734	NFKBIA Hs.8132	0.960867134	1.02990863	0.75472896	-2.40170019	-0.75949895	-0.76837571
IMAGE:1574629	UBE2D4 Hs.191	0.67787128	1.31874585	0.84906994	-1.60933386	-1.60933386	-0.8375954
IMAGE:76169	ANTXR2 Hs.162	1.073857892	0.54479433	1.42723242	-1.40350717	-1.57570239	-1.23280931
IMAGE:133085	FAM46A Hs.107	1.055499398	0.78795289	0.63445146	-1.82900329	-1.1114922	-0.61774728
IMAGE:132305	LY6G6C Hs.241	1.443781221	-0.00567617	0.96399959	-1.2805526	-1.24684429	-0.83476018
IMAGE:1010428	Hs.544119 Trar	-1.353277739	-1.19145915	-0.59273035	2.1774017	1.32785409	1.05276248
IMAGE:1585952	C1orf187 Hs.37	1.316704807	0.20559393	1.144927	-1.25522004	-1.37172919	-1.08765763
IMAGE:812976	PANX1 Hs.5919	1.199685006	1.1388351	0.53548446	-1.76626866	-0.97584975	-1.43501733
IMAGE:593183	EVI2B Hs.5509 I	0.751591834	1.72445686	0.0729629	-1.25554602	-1.11341113	-1.43631952
IMAGE:2568378	FAM50A Hs.542	0.950791248	1.06608791	0.7953914	-2.22663438	-1.2641579	-0.52876571
IMAGE:503602	CAMK2N1 Hs.19	-1.028770744	-0.87812952	-0.66268416	1.4984489	0.73955822	1.45069847
IMAGE:80344	Hs.362807 MRN	0.691937555	1.40822836	0.05862486	-1.03618377	-1.0640912	-1.12325496
IMAGE:145932	FCER1G Hs.433	1.071445192	0.74266676	0.58163242	-1.31781789	-1.07123407	-1.05915649
IMAGE:276920	N39449::N48	-1.471754653	-0.60796761	-0.71442353	1.86678226	1.05260461	1.09322595

IMAGE:134748	LOC646504 Hs.6	-1.159681405	-0.68305892	-0.88275331	0.9562441	1.53706252	1.3742455
IMAGE:772220	PDIA5 Hs.47735	0.622850798	1.65375174	0.83676274	-1.59584133	-1.77947056	-1.08554536
IMAGE:839081	ELOVL5 Hs.5201	-1.366844675	-0.42349688	-0.37725765	0.55554288	1.4288568	1.17268472
IMAGE:1517162	KLRK1 Hs.38778	0.631780594	1.57504318	0.09587977	-0.62219009	-1.49995295	-1.30794233
IMAGE:854444	HLA-DQB1 Hs.4	0.123787149	1.39705557	1.1759609	-2.13083564	-0.85756721	-0.76281236
IMAGE:2022019	NAGPA Hs.2133	0.894000628	1.04595117	0.9895124	-2.3015735	-1.1131032	-0.73214149
IMAGE:1867895	SCNN1G Hs.371	-1.140074711	-1.31450799	-0.16191934	1.29949928	1.28132915	1.30344479
IMAGE:2322367	RTN4 Hs.429581	-0.737495562	-1.03742045	-0.81202235	0.63988678	1.70780116	1.32971403
*mitoch. cont. IMAG	151937	-1.089055804	-1.04492854	-0.57129591	1.43042711	1.02224992	1.46241938
IMAGE:2061465	GGT2 Hs.45490	-1.558840753	-0.71104889	-0.64428528	1.14164128	1.46751128	1.6010385
IMAGE:1569449	B3GALT5 Hs.197	-1.026192348	-0.75486215	-0.86000625	1.21702789	0.97607265	1.55348902
IMAGE:488301	AA085748 134	0.932018398	0.43400982	1.35397415	-2.24089641	-0.90080893	-0.59980467
IMAGE:201173	NXF4 Hs.454283	-1.354988018	-1.17643007	-0.23676887	1.40168631	1.2199664	1.47143551
IMAGE:740473	ZNF397 Hs.5910	-1.14344358	-0.99148187	-0.60391456	1.60414106	1.31775168	1.03538864
IMAGE:1606837	IMPDH2 Hs.476	0.90699835	1.09775007	0.29659284	-2.3726815	-0.64999613	-0.35518606
IMAGE:1881183	FMNL1 Hs.1002	-0.965197645	-1.06267039	-0.64296422	2.67082237	0.44844382	0.72624114
IMAGE:23116	SPIRE2 Hs.4617	-1.355602258	-0.66699698	-0.93984058	1.27014262	1.68740344	1.25115351
IMAGE:1915913	CLIC2 Hs.63283	0.709631635	1.37845517	0.36236886	-1.01483075	-1.18617751	-1.38408302
IMAGE:511068	LGALS4 Hs.5302	-1.187566977	-1.12189669	-0.27912806	1.37302682	1.12785776	1.312221
IMAGE:309515	COMP Hs.1584 (	1.77493934	-0.65921235	0.58315007	-1.00875449	-0.75205749	-0.64171609
IMAGE:448218	RXRA Hs.59088	1.053050001	0.83648142	1.10662223	-2.22311975	-1.32549996	-0.6689552
IMAGE:325355	LTBP2 Hs.51277	-0.91558092	-1.15757909	-0.42441169	0.9318326	1.33524578	1.37317624
IMAGE:1422743	AA827293 22	-1.186213493	-1.1716322	-0.52033435	1.36381199	1.4269976	1.39637688
IMAGE:308579	FRG1 Hs.20377	-1.109419058	-0.83811819	-0.74838021	2.50175203	0.73307906	0.62195005
IMAGE:714213	FAS Hs.244139	0.919983079	1.15432434	0.54286237	-1.62408194	-0.95592812	-1.21002904
IMAGE:139681	NNAT Hs.50470	-0.940557805	-1.01844345	-0.46402227	1.10658852	1.26547524	1.14646597
IMAGE:2189995	HDGF Hs.50674	1.397289984	0.75348625	0.48691126	-2.22838129	-1.13441791	-0.47200422
IMAGE:1324405	MGC42367 Hs.4	-1.467012139	-1.25253412	-0.23313272	1.68351675	1.51099937	1.17621916
IMAGE:712829	LMO2 Hs.34560	0.99787227	0.73382539	0.921454	-1.69493998	-1.10875589	-0.94566811
IMAGE:1604769	Hs.434909 Tran	-1.205523204	-1.22621083	-0.2093346	2.64901855	1.32779556	-0.06754481
IMAGE:2577245	TXNRD1 Hs.567	0.903332487	1.05168056	0.48578693	-2.10469614	-1.08774293	-0.34731416
IMAGE:149966	H01178::H01	-1.262990353	-0.67411509	-0.60994278	1.96129046	1.20183955	0.50495663
IMAGE:771157	KCTD5 Hs.6196	1.56053789	0.67540849	0.2368374	-1.4268646	-1.15457294	-1.06852878
IMAGE:490525	NFXL1 Hs.51861	0.945641691	0.09325143	1.53423745	-1.63987395	-0.9048393	-0.93142324
IMAGE:2565790	CD2BP2 Hs.202	1.415559092	0.76604572	0.52297025	-2.04777417	-1.26835812	-0.60998774
IMAGE:487905	RP11-125A7.3 H	-1.439967967	-0.76037898	-0.58432438	1.66447664	1.15820564	1.20824361

IMAGE:376875	FMO1 Hs.1424 F	1.254563187	1.18902446	0.43354173	-2.18234702	-1.27970006	-0.74526157
IMAGE:366902	SLPI Hs.517070	0.891115136	0.61974627	0.96521063	-1.792133	-0.85237082	-0.82830158
IMAGE:281240	SYN2 Hs.44550	-0.963958688	-1.09654252	-0.28649808	0.95775842	1.31701783	1.17409816
IMAGE:741977	CFB Hs.69771 C	0.191453737	1.35958825	0.86928425	-1.71072902	-0.55837384	-1.14406544
IMAGE:841698	EXT1 Hs.492618	1.392809004	0.7215528	0.59623146	-1.98171022	-0.97369075	-0.96143106
IMAGE:1636360	CGNL1 Hs.1489	-1.428876866	-1.10273872	0.38487203	1.0439867	1.06108265	1.211264
IMAGE:78844	C1QTNF1 Hs.20	1.674225094	0.78630549	0.54369084	-1.72331798	-1.17514226	-1.47194919
IMAGE:687297	RAB3B Hs.1230	-1.026156841	-0.93733159	-0.4758583	1.78427404	1.06917853	0.68660296
IMAGE:629916	TIMM17B Hs.30	0.927779144	1.2178859	0.71716783	-1.95186597	-1.48728219	-0.6758092
IMAGE:295974	N73548 8290	1.316035841	1.26930565	-0.05128965	-1.61651751	-1.0671707	-1.13021196
IMAGE:1048696	APOBEC3F Hs.4	0.87841078	1.48266286	0.16780403	-1.26384798	-1.20476205	-1.28275547
IMAGE:234376	LOC646652 Hs.	-0.686002941	-1.55474972	-0.80273445	1.42946122	1.52682472	1.40826111
IMAGE:2062453	PHF19 Hs.46012	0.989175428	1.04766888	0.85059095	-1.67835083	-1.24639921	-1.1937551
IMAGE:1941633	SLC25A18 Hs.28	-1.079468525	-0.70451337	-0.60263615	1.12393443	1.18567295	1.11966067
IMAGE:2565353	SEPT9 Hs.44093	0.793134748	0.95803354	0.87723313	-2.23007844	-0.69651969	-0.79669571
IMAGE:1501598	Hs.148141 Tran	-1.040926292	-0.91476013	-0.41712476	0.75711847	1.53680602	1.1610111
IMAGE:42118	P2RX4 Hs.3217	0.763372787	1.70102884	0.39295586	-1.96269613	-1.21615728	-1.00894385
IMAGE:815740	C3orf52 Hs.434	1.209013598	0.77510588	0.78285808	-0.78114775	-1.78247324	-1.39113083
IMAGE:40227	Hs.404323 Tran	1.298792763	-0.85234766	1.4409302	-1.85875703	-0.66287082	0.05079744
IMAGE:1638479	ZNF415 Hs.147	-0.763942613	-1.39774283	-0.59010027	0.87676998	1.57274298	1.53064054
IMAGE:2306953	LY96 Hs.69328 I	0.636187527	1.39767125	0.26699759	-1.27447651	-1.07109985	-1.03895879
IMAGE:742561	AA401344::A	-1.124262829	-0.99797611	-0.95467781	2.14205289	1.0686158	1.16603698
IMAGE:898236	EEF1G Hs.4444	-1.086960997	-0.87167383	-0.64471331	2.34878302	1.00443425	0.39062661
IMAGE:868380	FCGR2B Hs.352	0.851461327	1.20442276	0.25271821	-1.05506847	-1.01130699	-1.33334843
IMAGE:1500279	Hs.553287 Tran	-1.176048075	-0.87037514	-0.84101704	1.57689241	1.82323049	0.72078323
IMAGE:767753	Hs.166891 Tran	0.459843063	1.64277989	0.42357183	-1.6086477	-0.99657063	-1.0781809
IMAGE:2548367	ALDOC Hs.1552	1.571787427	0.56065903	0.35676206	-0.63159506	-1.7534462	-1.259581
IMAGE:782748	BIN3 Hs.232256	0.983635489	0.50808359	1.04511368	-2.3998123	-0.86229244	-0.28186598
IMAGE:1689083	Hs.1987 Transc	0.46948589	1.55645152	-0.14884335	-0.53727372	-1.06124569	-1.25433253
IMAGE:489729	ETS1 Hs.369438	0.947164007	1.3838243	0.05181372	-1.50739612	-1.27493978	-0.77891371
IMAGE:1877697	LOC130576 Hs.	1.208488916	0.46014336	0.92197948	-1.26051688	-1.46175201	-0.93315388
IMAGE:2063982	KCNK6 Hs.2403	1.232074414	0.8026432	0.36728879	-1.29971877	-1.18495698	-1.02651167
IMAGE:2545528	PIN1 Hs.465849	0.98527352	0.97562442	0.89167719	-2.04140974	-1.17299022	-0.84154344
IMAGE:377641	TNFAIP8L1 Hs.4	0.221242825	1.60428826	0.4242382	-1.91535169	-0.92701949	-0.4262232
IMAGE:769571	SREBF1 Hs.592	-0.997878729	-1.54322148	-0.16563376	1.11934957	1.48550828	1.41383466
IMAGE:1741991	ZNF480 Hs.147	1.585053115	1.51340578	0.95455659	-1.4402555	-2.38361205	-2.01701653

IMAGE:2019211	RAB33A Hs.562	0.625283654	1.23472698	0.69939542	-0.54341566	-1.35674342	-1.76410114
IMAGE:288983	PI16 Hs.25391	1.397664461	-0.06742138	1.04793429	-0.85859728	-1.27262187	-1.17406334
IMAGE:730288	EHD4 Hs.14370	1.076934212	1.05311282	0.35154943	-2.33956738	-0.82465132	-0.47028864
IMAGE:489266	PRIC285 Hs.517	0.875772274	1.23873239	0.70171524	-2.12253082	-0.86115057	-1.06521965
IMAGE:687990	ARHGEF6 Hs.52	0.590493681	1.17890993	0.68711171	-0.70877003	-0.48270102	-2.32152401
IMAGE:1069393	EXT1 Hs.492618	1.213484119	0.54581631	1.32105282	-2.14247396	-1.15333646	-0.99445625
IMAGE:814731	AIP Hs.412433	0.628674705	1.33898257	0.88001441	-1.7970515	-1.47666788	-0.77778455
IMAGE:70749	Hs.593210 CDN	-1.521832526	-0.47421799	-0.85516873	0.8440303	1.50355127	1.71545512
IMAGE:454564	LOC389289 Hs.	0.834544243	1.65702957	0.358112	-0.92222349	-1.88178971	-1.38098753
IMAGE:811054	GNA12 Hs.4873	0.869383452	1.22414468	1.09744424	-1.85689322	-1.14420326	-1.51100102
IMAGE:564884	KRIT1 Hs.53198	-0.839212603	-0.73554663	-1.35754249	1.22576858	1.29112321	1.54217516
IMAGE:971372	NPTX2 Hs.3281	0.812949814	0.32206841	1.84214611	-1.54410825	-1.46137543	-0.99972629
IMAGE:358531	JUN Hs.525704	1.056548168	0.99876612	0.87954248	-1.63441021	-1.35994546	-1.18804385
IMAGE:79032	DLG2 Hs.50345	-0.912480469	-0.91000992	-0.65208413	0.83210087	1.60003124	1.11670863
IMAGE:506016	MYADM Hs.3809	1.223543909	0.46858405	0.97529134	-1.19725869	-1.20873612	-1.35131131
IMAGE:2420792	SLC7A7 Hs.513	0.82677336	0.82296056	1.1638251	-1.35453097	-1.42983382	-1.14501747
IMAGE:2309141	CD97 Hs.46603	0.870661089	0.94057871	0.89396696	-1.5382408	-1.28633173	-1.00974586
IMAGE:118447	MAPK8 Hs.1382	-0.783659971	-1.13971074	-0.83955611	1.41565999	1.19165611	1.32718511
IMAGE:866483	LOC283537 Hs.	-1.241044856	-0.21464647	-0.96943525	0.56210935	1.51775309	1.31546861
IMAGE:79254	MICA Hs.54905	0.924899535	0.92125543	0.825051	-1.75661674	-1.29745923	-0.74647023
IMAGE:159462	CDC42EP1 Hs.2	0.96802482	1.07940696	0.71608902	-1.92105999	-1.10116367	-0.94403529
IMAGE:1911663	Hs.452702 CDN	-0.766225179	-1.15288793	-0.47040488	0.9240268	1.51799669	1.02465228
IMAGE:745918	ACPP Hs.43306	0.898911159	0.8530323	0.80439508	-1.15857269	-1.38465976	-1.0901766
IMAGE:210687	AGTR1 Hs.4778	0.459172751	1.05363747	1.71860848	-0.94300363	-1.803595	-1.6708773
IMAGE:781007	RPL27A Hs.523	0.83910529	0.94127656	0.66855786	-2.26277525	-0.92472455	-0.31877031
IMAGE:768069	KLHL18 Hs.517	1.964240081	-0.01397837	0.3849083	-1.88598918	-0.87093611	-0.64960266
IMAGE:770267	LOC158381 Hs.	-1.265689732	-0.92589756	-0.6112407	1.29275915	1.06933416	1.68933851
IMAGE:2010562	ADAMTS10 Hs.4	0.714145922	1.18215518	0.65001371	-1.54510802	-0.88615966	-1.22570111
IMAGE:795544	WASPIP Hs.591	0.488346495	1.41586359	0.59676168	-1.22092601	-1.31074673	-1.07059449
IMAGE:1698046	HIST1H2BG Hs.	-1.735492871	0.0538607	-0.81666938	1.82871489	1.14534311	0.56922699
IMAGE:323028	SMAD3 Hs.3691	1.191307127	1.2483336	0.340979	-2.38393613	-1.06092188	-0.64082684
IMAGE:454698	FZD4 Hs.59196	-1.374002087	-0.86310932	-0.24130835	0.78174505	1.54496646	1.33059104
IMAGE:759163	MFAP4 Hs.2960	1.348055719	0.10559428	0.87332784	-0.99403301	-1.42143485	-0.85666694
IMAGE:810454	BACE2 Hs.5294	0.673223754	1.32895301	0.62928741	-1.88441049	-1.08796057	-0.81750334
IMAGE:1861700	AI053583::AI	-1.187684106	-0.90281825	-0.72536084	1.83873206	1.17155444	1.03216809
IMAGE:47074	ANKH Hs.15672	-0.917223186	-1.24171649	-0.97299547	0.96445771	1.61024903	1.87994712

IMAGE:842806	CDK4 Hs.95577	1.00327867	1.07311357	0.64199946	-1.994268	-1.28195203	-0.64086765
IMAGE:916090	AA579722::AI	-1.106723514	-0.70323813	-0.86884256	1.49365209	1.36734362	0.93999996
IMAGE:210494	Hs.596334 CDN	-1.213968892	-1.17809304	0.14666994	0.50155763	1.4259115	1.47728635
IMAGE:1623425	PRDM5 Hs.1325	-0.826828854	-1.47546826	-0.81665412	1.47933861	1.51749387	1.47743085
IMAGE:428100	SFRP1 Hs.21342	-1.355482972	-0.45815111	-0.4755107	1.10830101	0.95420313	1.25233535
IMAGE:1882829	AI279428 168	-0.856086397	-0.80827761	-0.44134517	2.314682	0.37394405	0.35960142
IMAGE:768597	MUC1 Hs.89603	-1.3136966	-0.50866315	-0.38068248	0.60805575	1.32284143	1.27849554
IMAGE:206638	SEC61A1 Hs.518	1.046478008	1.29379417	0.13618099	-1.905479	-0.96828092	-0.80687458
IMAGE:49356	GOLGA8A Hs.18	-0.843157549	-0.9516202	-0.96293804	0.53062412	1.30416719	2.06104787
IMAGE:1505405	Hs.602479 Tran	-1.231036086	-1.614859	-0.23761208	1.37218638	1.75036484	1.44330651
IMAGE:1877234	Hs.528324 Tran	1.248369879	1.17747913	0.10750135	-1.91341682	-0.94380853	-0.91592484
IMAGE:2547341	SLC2A1 Hs.4737	1.507697448	0.57156843	0.20851839	-1.40225361	-1.18172321	-0.79556999
IMAGE:743848	JMJD2C Hs.1571	-1.602140363	-0.34261336	-0.760246	1.37354291	1.04665439	1.4472408
IMAGE:810521	LMO2 Hs.34560	0.810266919	0.95552766	1.13256419	-1.65167568	-1.15461158	-1.25810985
IMAGE:1899292	Hs.604363 Tran	-1.250354758	-1.20377731	0.05924795	1.20815844	1.27899498	1.11998474
IMAGE:249949	H2AFV Hs.48818	1.147017827	0.99210934	0.58977461	-1.60508277	-0.9473449	-1.39348134
IMAGE:306052	Hs.596963 Tran	-1.58921585	-0.33021656	-0.56462484	1.25071603	0.92016796	1.41404567
IMAGE:298523	WDR53 Hs.3858	1.756241181	0.54496075	0.59000334	-1.33191523	-1.59669261	-1.26069924
IMAGE:32299	IMPA2 Hs.36799	1.095147825	0.67689022	0.51630373	-2.1049299	-0.69496419	-0.50354264
IMAGE:1698856	COL9A2 Hs.418	-1.177751816	-1.1093803	-0.08346744	0.80281107	1.23140862	1.50081279
IMAGE:429047	TTC3 Hs.368214	-0.74153415	-0.51273131	-1.29903179	0.79446766	1.15487863	1.55584165
IMAGE:344550	Hs.229128 Tran	-1.137354329	-0.7154127	-0.48736796	1.31805159	0.95302302	1.11728588
IMAGE:813414	SERPINA12 Hs.9	1.406134557	-0.02420414	0.95063116	-0.79406291	-1.39995013	-1.06717153
IMAGE:2421421	SNX4 Hs.50724	-0.909698059	-1.09156003	-0.79884886	0.99833711	1.58289344	1.41921766
IMAGE:754989	KIAA0409 Hs.51	-0.671187853	-1.62008636	0.14205826	1.33235789	0.9965681	0.9304125
IMAGE:324543	MRGPRF Hs.118	0.921006239	0.383733	1.40053175	-1.38957329	-1.27962111	-1.03857914
IMAGE:713205	UTP11L Hs.4720	1.797333796	0.41831079	-0.1208616	-0.98393979	-1.32412318	-0.86432692
IMAGE:704046	ULK4 Hs.563159	-1.184078636	-1.01895307	-0.04387264	0.75362785	1.10766825	1.49809225
IMAGE:66686	RPL10 Hs.53440	0.762129511	0.91587194	0.80761165	-1.97272415	-0.95898499	-0.5948076
IMAGE:197596	C9orf77 Hs.380	-1.492051343	-0.472104	-0.472104	1.55294272	1.23635192	0.74706836
IMAGE:1591736	MS4A2 Hs.3867	0.792920432	0.46493377	1.31293676	-0.46923567	-1.04934814	-2.00936843
IMAGE:2461316	CST7 Hs.143212	0.542509873	1.33819204	0.42770114	-1.05406296	-1.17042317	-1.13119316
IMAGE:453195	ANGPTL2 Hs.64	1.413939526	0.08487851	1.08299453	-1.82751707	-0.91336587	-0.86108727
IMAGE:208656	MRPL48 Hs.503	-1.418313765	-0.77588751	-0.42931546	1.60003299	1.0729295	1.15498375
IMAGE:1893670	Hs.603516 Tran	-1.426459726	-0.32159821	-1.05393716	2.15408862	1.16647064	0.61894909
IMAGE:322807	Hs.37517 Trans	-1.007919164	-0.85419921	-0.48061684	2.0225381	0.77215997	0.59925054

IMAGE:2028487	TMPRSS2 Hs.43	-1.096827529	-0.80674243	-0.55733592	1.63780808	0.43245446	1.4817623
IMAGE:365883	C21orf33 Hs.41	-1.346094434	-0.69025908	-0.33291743	0.40569499	1.44794145	1.61704062
IMAGE:252993	SEC31L1 Hs.370	-0.889834136	-0.97910873	-1.03831188	1.22057023	1.65452529	1.22620862
IMAGE:845355	CTSC Hs.12806	0.660625026	1.53661872	-0.04878626	-1.28049084	-0.97461108	-0.97978088
IMAGE:359610	BMP2 Hs.73853	1.029985074	0.94546399	0.70772371	-0.83917039	-1.29012533	-1.71853251
IMAGE:1670632	ADORA1 Hs.778	-1.239841774	-0.51931778	-1.00219526	1.58600277	1.05635443	1.2491262
IMAGE:755881	AQP5 Hs.29802	-1.147591302	-1.23946112	-0.67329806	1.4593679	1.42261997	1.54021334
IMAGE:1084673	AFAP Hs.529369	1.270086321	0.19815528	0.8479837	-1.34804569	-1.29266259	-0.62163376
IMAGE:782140	SLC35D2 Hs.49	1.014438055	1.54913434	0.41079066	-1.7185625	-1.18646814	-1.45381629
IMAGE:151055	ELOVL5 Hs.520	-1.297787285	-0.36508246	-0.47675281	0.64492405	1.41714644	1.02817516
IMAGE:490959	PHF17 Hs.1242	-1.081135709	-0.80126148	-0.45919298	-0.32903642	1.8076378	1.91898562
IMAGE:247616	LHFP Hs.507798	1.062755952	0.49842139	1.31525536	-1.58739944	-1.51955837	-0.87887741
IMAGE:121454	ALOX12 Hs.422	1.336291786	-0.86865165	1.30395262	-0.69825502	-0.94779862	-0.68534735
IMAGE:1620712	FBLN5 Hs.3327	0.921964753	-0.068664	1.69947019	-1.28629539	-1.21203112	-0.90596236
*mitoch. cont. IMA	141567	-1.347203663	-0.65714141	-0.3863809	0.43006235	1.52918213	1.53024924
IMAGE:1031372	AA609127 22	-1.346584529	-1.08483993	-0.66873314	1.03864699	1.66784073	1.77656541
IMAGE:383603	MLH3 Hs.43665	-0.437218333	-1.34195594	-1.20708823	1.22378198	1.3084757	1.64536402
IMAGE:2298441	GGPS1 Hs.498	-0.921767259	-1.04092699	-0.67897932	0.81405483	1.59045492	1.38825576
IMAGE:487824	FCHSD1 Hs.591	1.533110484	0.36331405	0.57229547	-1.87252407	-0.82417504	-0.86330702
IMAGE:868332	HLA-DPA1 Hs.34	0.955798289	0.79827716	0.62739059	-1.7085762	-0.7328203	-0.97395492
IMAGE:32496	FLJ42709 Hs.45	-1.113811127	-0.71688444	-0.43841403	0.98477921	1.17921356	1.13006812
IMAGE:854706	AA630097 26	-0.930576657	-0.94729097	-0.31214725	2.03663091	0.66570942	0.50464517
IMAGE:841686	LOC644845 Hs.	-0.772381671	-0.89336167	-0.49450574	1.89749565	0.62125119	0.59800035
IMAGE:128791	NRBP1 Hs.5158	1.188824686	0.86628296	0.74200953	-1.82006272	-1.23664166	-0.953469
IMAGE:924428	FAM3D Hs.6126	-1.209782105	-0.89201826	-0.26232875	0.93408133	1.23797122	1.30855893
IMAGE:453348	TUBGCP2 Hs.52	0.993332612	0.9133949	0.58326252	-2.535087	-0.65862699	-0.39545542
IMAGE:2005924	Hs.604701 Tran	0.552618542	1.81895429	0.25750725	-1.31990662	-1.36945889	-1.1898778
IMAGE:845054	INSR Hs.591381	-1.381237045	-1.33198719	0.20898617	1.22591758	1.48884408	1.09384197
IMAGE:714106	PLAU Hs.77274	1.405667928	0.84942711	0.27512035	-1.96909742	-0.65693959	-1.10050599
IMAGE:2307420	TUBB2B Hs.300	1.443232273	0.76591044	-0.00706824	-1.08142543	-0.86931675	-1.35413659
IMAGE:67237	Hs.632968 Tran	-1.298956309	-1.08004272	-0.22839823	2.7131649	0.74956294	0.39126848
IMAGE:773138	CAMK2N1 Hs.19	-0.893630005	-0.98361475	-0.48903608	1.18535513	0.81416804	1.42763907
IMAGE:144853	R78464::R78	-1.193315195	-0.69335163	-0.17615769	0.71000653	1.42817453	0.91201629
IMAGE:809489	NOL6 Hs.49370	1.899250827	0.4057996	0.09258969	-1.56654129	-1.06094582	-0.94582562
IMAGE:998788	AA535935::A	-1.565586318	-0.63429746	-0.46905339	1.06900597	1.35555639	1.46158005
IMAGE:79353	PPP4R1 Hs.464	1.273321516	1.01933875	0.28232693	-1.88229895	-1.09264343	-0.81695668

IMAGE:251529	KLF13 Hs.52575	1.405749352	0.59336603	0.48230406	-1.68748172	-1.29721152	-0.6168599
IMAGE:767272	CPD Hs.446079	-1.420010855	-0.582792	-0.70372361	0.85106157	1.67432677	1.35847046
IMAGE:825583	RALY Hs.136947	0.69578497	1.14763204	0.94252393	-2.06784373	-1.21491892	-0.66051779
IMAGE:1454534	ZNF681 Hs.1873	-0.740617102	-0.87147019	-0.91247484	0.60308932	1.64006486	1.31557031
IMAGE:243155	NUP85 Hs.36281	1.022702172	1.21770182	0.6023696	-1.94481329	-1.14856473	-1.02018996
IMAGE:1559703	EXOSC5 Hs.283	1.014300602	1.03231682	0.58335269	-1.31645741	-1.20836011	-1.27429947
IMAGE:712914	NOD3 Hs.59209	0.717378326	1.53567189	-0.00056484	-1.0064682	-1.17487143	-1.19752965
IMAGE:782446	C21orf56 Hs.381	0.851846628	1.28623211	0.08393299	-1.01888188	-1.23607462	-1.05707784
IMAGE:2019015	SLC7A1 Hs.1484	0.908921182	1.37092464	0.59288814	-2.22813188	-1.27152963	-0.6612174
IMAGE:595547	AA176528::AA	-1.138103702	-1.24299744	-0.04332393	1.29057471	1.08078725	1.25444465
IMAGE:2019798	COL9A2 Hs.4180	-1.274042823	-1.10028415	-0.1517452	0.81024332	1.34564808	1.59528055
IMAGE:232908	PX19 Hs.279529	0.930646696	1.12601197	0.40967265	-1.96286616	-0.75619832	-0.87801432
IMAGE:281605	FMNL1 Hs.10021	0.749903738	1.4026811	0.57212607	-1.32578939	-1.50634483	-1.11190064
IMAGE:52419	C9orf61 Hs.1180	-1.167618375	-1.1088645	-0.16235851	1.1357231	0.87543711	1.60651223
IMAGE:587525	RNF113A Hs.458	0.858899589	1.37082603	0.33141059	-1.12343316	-1.52381083	-1.11160766
IMAGE:271978	NT5E Hs.153952	0.707073766	1.11329013	0.62472583	-0.98444833	-1.31208266	-1.21492214
IMAGE:111004	ZNF432 Hs.6434	-1.127327949	-0.89903013	-0.67309401	1.31309704	1.32024775	1.24755983
IMAGE:140301	Hs.28792 CDNA	-1.043999197	-0.90418493	-0.61988393	1.31193715	0.72270276	1.66249119
IMAGE:153694	SH3BP2 Hs.1676	1.055208048	0.91345055	0.89076935	-1.08777154	-1.61621199	-1.36246607
IMAGE:366518	UBADC1 Hs.919	1.197448588	0.94100122	0.65771077	-1.58919956	-1.45474444	-0.98586918
IMAGE:611381	Hs.529890 **F	-0.237612007	-0.50245369	-1.94867845	1.24269761	1.42781654	0.87543246
IMAGE:767798	ATOX1 Hs.12521	0.778664925	0.99350156	0.86069346	-2.2966726	-0.94832862	-0.48911532
IMAGE:2306221	WNT10B Hs.919	0.83748022	1.05525707	1.03644907	-1.2808592	-1.32222266	-1.5315854
IMAGE:531402	MCM5 Hs.51758	0.351175602	1.44380712	0.58197005	-1.802842	-1.18751793	-0.42957671
IMAGE:782307	Hs.405628 CDN	-0.836308621	-1.21252264	-0.70087158	0.7687928	1.61213922	1.56840434
IMAGE:730555	DNAJB1 Hs.5152	1.439908696	0.88298444	0.08027708	-1.7720714	-1.3164061	-0.49620856
IMAGE:462802	NUDT21 Hs.528	-1.050907658	-1.27768283	-0.42840351	1.44995661	1.3669941	1.2114394
IMAGE:121798	FAM105A Hs.591	0.84090861	1.29337604	0.36989457	-1.14970312	-1.27043109	-1.24366097
IMAGE:489968	ABL1 Hs.431048	1.366273196	0.13002857	1.15458747	-1.77443734	-0.82185158	-1.09139807
IMAGE:591101	INTS8 Hs.52169	-0.651004586	-1.64010206	-0.58053139	1.12102535	1.86649133	1.17480752
IMAGE:841292	UBE2I Hs.30290	1.529136443	0.78083083	0.53004732	-1.50386083	-1.66719755	-0.95645168
IMAGE:446927	TNF Hs.241570	0.619208491	1.16515897	0.65805497	-1.40933876	-0.96050447	-1.12927667
IMAGE:40562	SGCB Hs.43895	1.128892958	1.1869916	0.30590518	-0.89152146	-1.37872656	-1.58596028
IMAGE:2012523	LOC653327 Hs.6	1.498373574	0.29297316	0.24237103	-1.39448457	-0.74344493	-0.85205438
IMAGE:823878	Hs.567506 **T	-1.6306096	-0.73128091	0.0952649	1.51766825	1.00456289	0.90152352
IMAGE:1631656	CITED4 Hs.3558	-1.554070669	-0.46448477	-0.59052335	0.61847527	1.57344052	1.57407156

IMAGE:795401	LOC642255 Hs.6	0.73864794	1.31958155	0.53961503	-2.09959162	-1.21303641	-0.449235
IMAGE:1569604	Hs.484309 Trar	-0.662151971	-1.39686891	0.13404917	0.6093917	1.26193634	1.04964182
IMAGE:1456643	AA864787 264	-1.438491999	-1.26216648	-0.159074	1.89864487	1.60241799	0.74876735
IMAGE:1535106	HAO2 Hs.35636	-1.190090619	-0.810692	-0.09815476	0.92297666	1.21800901	0.98288171
IMAGE:1588906	TNIP2 Hs.36855	1.17813091	0.77599452	0.73582602	-1.95117621	-0.96276018	-0.93703428
IMAGE:45376	ACAA2 Hs.2001	-1.346318736	-0.43089907	-0.36432309	0.59775921	1.33187823	1.19159313
IMAGE:1898089	Hs.151334 Trar	-1.848172734	-0.72792106	-0.49415581	1.41037323	1.59948527	1.47197695
IMAGE:243817	Hs.151675 Trar	-1.01001313	-0.94116302	-0.39761501	0.6490907	1.36255659	1.41213568
IMAGE:346947	ZNF211 Hs.5909	-1.451044006	-1.10068773	-0.33605054	1.44775169	1.43673171	1.36317738
IMAGE:813591	ACOT8 Hs.4447	1.272170086	0.83789978	0.74770518	-1.9756707	-1.29921119	-0.8248544
IMAGE:1857169	SESTD1 Hs.591	-0.891628462	-1.71989986	0.24846275	1.13655375	1.56801277	0.90214753
IMAGE:504420	SORBS2 Hs.481	-0.348465032	-1.71445124	0.03959186	1.0796557	0.63725138	1.3279775
IMAGE:376343	TEAD1 Hs.5681	-1.174129705	-1.26217805	-0.80139172	1.72889752	1.2886558	1.63864796
IMAGE:179193	HLA-E Hs.11835	-1.533304619	-0.98334079	0.03570985	0.61074556	1.45096808	1.66861717
IMAGE:486493	GPR124 Hs.274	0.563105512	-0.11034711	1.94980568	-1.19019449	-1.00216163	-0.92403858
IMAGE:1750155	RFX2 Hs.46570	1.75949319	0.2108415	0.05225782	0.28931308	-1.52255107	-1.78758632
IMAGE:509731	TSPAN8 Hs.170	-1.082189396	-1.24516919	0.01497668	1.24317678	1.00065765	1.22848259
IMAGE:810960	Hs.609030 Trar	0.98702563	0.78073621	0.53536037	-1.88840465	-0.88274371	-0.54969486
IMAGE:2572023	GRN Hs.514220	1.037244126	0.75099141	0.80179817	-2.02293371	-1.03158231	-0.630085
IMAGE:291374	ARFGEF2 Hs.62	-0.392256827	-0.63830727	-1.40910292	0.87870943	1.03666286	1.39185251
IMAGE:838446	CYBRD1 Hs.221	0.778759271	-0.07263216	1.73349879	-1.18924854	-0.95196975	-1.08484587
IMAGE:814460	SURF5 Hs.7835	0.660011816	0.97658725	1.22313236	-1.50733076	-1.57927973	-0.89720356
IMAGE:1873606	TMEM79 Hs.347	1.448411273	0.30626864	0.65380633	-1.92641625	-0.92296237	-0.59989917
IMAGE:788654	GRB2 Hs.44435	0.457619523	1.68189074	-0.02138495	-1.22107039	-1.06051023	-0.90095357
IMAGE:842994	CTSZ Hs.25254	0.855284641	1.5092402	-0.04310165	-1.3049896	-1.12423928	-1.06368132
IMAGE:768561	CCL2 Hs.30364	0.935380234	0.8645048	0.34693242	-1.66674764	-0.53819269	-0.92855276
IMAGE:2419934	DSCR1L1 Hs.44	-0.940488042	-1.29296354	0.05733285	1.26219729	0.86673697	1.14957706
IMAGE:1908572	SLC16A4 Hs.35	-1.327199191	-1.06069442	-0.22355588	0.64787554	1.69606234	1.51734738
IMAGE:277745	CASP7 Hs.9216	-1.132768702	-1.37162883	-0.51875768	2.08306135	1.21473455	1.10724749
IMAGE:1703854	KCNJ10 Hs.408	-1.312607594	-1.26103096	0.11915981	1.43075366	1.50786073	0.77289367
IMAGE:1574914	FLJ10916 Hs.51	-1.046598301	-1.2581804	-0.14641266	1.14096821	1.33862802	1.16058764
IMAGE:1882494	PPP1R16B Hs.4	0.702907955	1.64079369	0.12312405	-1.28109932	-1.4563608	-0.93199741
IMAGE:2577077	NPEPPS Hs.443	1.731156164	0.68451685	-0.47246584	-1.31890496	-0.85095982	-0.86306244
IMAGE:1009971	Hs.377508 Trar	-0.978353745	-0.78415855	-0.67977012	1.98363401	0.70177538	0.8080717
IMAGE:877638	CLIPR-59 Hs.46	0.770180873	1.05579782	1.40411117	-1.68786644	-1.71921465	-1.08702591
IMAGE:220069	MGC18216 Hs.5	-1.151582955	-0.72219705	-0.72258493	0.74768361	1.24223377	1.72398381

IMAGE:781342	GPX4 Hs.43395	0.893600883	1.01704662	0.59545417	-1.76540682	-1.24432041	-0.60056174
IMAGE:509823	CEACAM6 Hs.46	-1.204220875	-0.6755263	-0.49067183	1.13381096	0.81853438	1.4806152
IMAGE:1631023	PRDM10 Hs.275	-1.37086434	-1.01491395	-0.0458103	2.29079078	0.76679273	0.5783468
IMAGE:383958	AA702728 71	-1.167679986	-0.57777485	-0.71880452	1.72780555	0.84637012	0.94251224
IMAGE:2018337	GBA Hs.282997	1.354952105	0.49264228	0.8252475	-2.06272051	-1.09262195	-0.64760849
IMAGE:434864	NR2C2 Hs.5916	-0.974504629	-0.80484129	-0.84254425	0.69064681	1.60682885	1.42472353
IMAGE:881059	FAM3D Hs.6126	-0.870481883	-1.13012272	-0.26943487	0.86774895	1.05781752	1.41213402
IMAGE:703855	PHC3 Hs.52959	-1.145164065	-0.58136416	-1.23107643	1.24418415	1.33760746	1.54684626
IMAGE:415806	AMMECR1 Hs.48	1.799408634	0.33672863	1.01824107	-1.06581004	-1.88823268	-1.52296452
IMAGE:593431	UGCG Hs.59301	1.287978767	0.99332499	0.33705067	-1.47038239	-1.24269538	-1.13019121
IMAGE:303035	PLXNA2 Hs.497	1.052361118	0.19013089	1.59968987	-2.17589968	-0.98044037	-0.7070103
IMAGE:26418	EDG1 Hs.15421	0.929652275	1.00099268	0.71187629	-1.56106668	-1.1264534	-1.09829272
IMAGE:781075	RGPD5 Hs.5926	-0.746993664	-0.69141689	-1.48565952	1.16611049	1.46167788	1.38690186
IMAGE:204790	FLJ20273 Hs.51	-1.085902761	-0.19058253	-1.07808906	0.27221665	1.59077918	1.39934343
IMAGE:196849	ZC3H14 Hs.325	-1.303187589	-0.93968059	-0.38674037	0.55095408	1.71827233	1.57850133
IMAGE:490789	RIC8A Hs.5922	0.923999171	1.19209561	0.61700247	-2.08209783	-0.93086019	-0.93243723
IMAGE:321310	Hs.597566 Tra	-1.450707741	-1.2678886	-0.35629726	2.26734524	1.52156419	0.73435666
IMAGE:489025	RAC1 Hs.41381	1.079540699	0.87706379	0.51860466	-1.91955483	-0.93529206	-0.72831566
IMAGE:384397	FAM84A Hs.260	-0.634606844	-1.35577547	-0.27896918	1.24681015	1.03983215	1.04764264
IMAGE:307069	ALDH3B1 Hs.52	0.5572536	0.80262471	1.30190156	-1.43718676	-1.22915473	-1.00085292
IMAGE:1325193	FOXI1 Hs.8723	-0.895204553	-1.04594381	-0.24938673	0.8639952	1.24265948	1.11680128
IMAGE:755299	IER2 Hs.501629	1.22040258	1.02592502	0.31219237	-2.20645411	-0.99826226	-0.55501549
IMAGE:841384	DDR1 Hs.63198	-1.293133095	-0.88731538	-0.5107543	0.91125928	1.55360737	1.44424894
IMAGE:505887	B4GALT4 Hs.13	1.0437886	0.91855196	1.07408779	-1.93876242	-1.12991839	-1.21743976
IMAGE:1584980	Hs.187624 Tra	-1.431458702	-0.14273565	-0.89943139	0.92949377	0.93989509	1.6161919
IMAGE:741919	APBA3 Hs.2552	-1.226116012	-1.19439298	-0.27286475	1.16013245	1.22721888	1.58449308
IMAGE:755093	SMA3 Hs.64264	-0.733418581	-0.7730693	-0.87516988	1.76742632	0.83439545	0.7518724
IMAGE:2239277	TRPC3 Hs.1509	0.956488891	0.42226191	1.36784367	-0.97190357	-1.11742477	-1.68860245
IMAGE:201443	TERF1 Hs.4427	-1.132571331	-1.26498909	-0.75187029	2.51429637	0.72665669	1.29522542
IMAGE:415145	HSD11B2 Hs.13	-0.688979433	-1.41342239	-0.00457004	0.71946161	1.01422928	1.4256244
IMAGE:1893420	Hs.603935 Tra	-0.812694805	-0.79653118	-0.428045	2.57063168	0.48098483	-0.10272128
IMAGE:2020460	TXLNA Hs.1798	0.870049668	0.96766393	0.62575019	-2.4902554	-0.78068668	-0.26781606
IMAGE:725907	TCEA2 Hs.5050	-1.258550549	-1.1707131	-0.05710264	1.38138606	1.12870298	1.20518473
IMAGE:2012757	PXDN Hs.33219	0.310190835	1.35626217	0.75714859	-1.57358665	-1.10896796	-0.76356064
IMAGE:1637233	TFCP2L1 Hs.156	-0.78190032	-0.61192199	-1.14277739	2.21952474	0.57204246	0.72763801
IMAGE:142944	FAM46A Hs.107	0.297779488	0.99443617	1.46772206	-1.41535071	-1.48617047	-0.87145489

IMAGE:344806	SBEM Hs.34841	-1.26485284	-0.56802723	-0.32078007	0.67219643	1.48918249	0.98891627
IMAGE:342685	C9orf91 Hs.522	-1.439296836	-0.25828539	-0.95462344	0.66510861	1.6648511	1.40969294
IMAGE:1660695	ZNF595 Hs.2359	-0.757334935	-1.40947433	-0.60916887	1.53786033	1.03030956	1.44350508
IMAGE:1551288	Hs.535619 Tran	-1.116842753	-1.10075004	-0.17488268	1.21494996	1.76602219	0.56402039
IMAGE:1472689	TOMM40 Hs.110	-1.30366849	-0.48643792	-0.21070902	0.6760069	1.236038	1.03650099
IMAGE:136169	SAMSN1 Hs.570	0.566993412	1.39842044	0.01654506	-0.83859451	-0.77906512	-1.35114248
IMAGE:813748	HIPK2 Hs.39746	-0.87527324	-1.11153538	-0.90551479	0.46264549	2.09501435	1.55444658
IMAGE:212496	MAN2A1 Hs.432	1.061875568	0.82158755	0.45621808	-1.91818967	-0.89614266	-0.58113494
IMAGE:282404	CTA-221G9.4 Hs	-0.825080783	-1.370525	-0.19330727	0.80791641	1.08456257	1.64256378
IMAGE:839888	FIBP Hs.7768 Fi	0.874826449	0.9771346	0.7111334	-1.8418643	-1.30781573	-0.51717831
IMAGE:843251	KIAA1841 Hs.46	-0.622007354	-1.22349788	-1.44458629	1.82581525	0.77036194	1.97781353
IMAGE:1556290	KIAA1409 Hs.12	-0.662529493	-1.57820404	-0.61246383	2.10195126	1.11381325	0.91091558
IMAGE:223350	CP Hs.558314 C	1.326019355	0.17116202	0.90066021	-0.48888608	-1.51702745	-1.3656838
IMAGE:795825	SLC24A6 Hs.286	1.006896806	0.83541965	0.88358739	-2.01008968	-1.05004638	-0.80782285
IMAGE:1912786	MRAS Hs.52702	-1.168531525	-0.68787756	-0.56514454	0.80240163	1.13845634	1.55018634
IMAGE:294483	EIF4E2 Hs.2920	1.122750764	0.80567876	0.9033168	-1.76537259	-1.23691925	-1.01949844
IMAGE:824531	PSCDBP Hs.270	0.644225716	1.38124898	0.10231088	-1.02942133	-0.90698165	-1.22969766
IMAGE:868368	TMSB4X Hs.522	0.813966147	1.33485579	0.23976277	-1.46933883	-1.19916785	-0.85442969
IMAGE:810154	Hs.569375 **T	-1.031877639	-0.95561664	-1.01789646	0.70900655	1.69222868	1.85237677
IMAGE:214600	CPEB4 Hs.12712	-0.837313628	-0.90445059	-0.77890447	0.94064088	1.76642547	0.87921056
IMAGE:1567422	EIF2A Hs.37880	-1.23271807	-0.42881727	-0.87683085	1.04042168	1.32026056	1.22765933
IMAGE:25389	SLC2A1 Hs.4737	1.48145623	0.30090214	0.33704155	-1.32875943	-1.11041716	-0.6556629
IMAGE:1622354	Hs.131064 Tran	-0.984900191	-0.99133476	-0.37905117	1.34231874	1.34352355	0.7523241
IMAGE:431944	CD55 Hs.52765	1.384897091	0.5112276	0.50508118	-0.24598921	-1.60423794	-1.62531138
IMAGE:843287	MCL1 Hs.63248	1.102258914	1.35714243	-0.19254936	-1.34964974	-1.2165439	-0.88222168
IMAGE:1543217	AA918191 10	-1.35140127	-0.68803805	-0.65977926	1.59828998	0.89565183	1.38994124
IMAGE:71794	C10orf76 Hs.16	0.988911468	0.29486974	1.55049925	-2.27453204	-0.82382244	-0.76544139
IMAGE:1610304	PCYT1B Hs.2960	-1.026310357	-0.81431136	-0.50717699	1.10518732	1.20841105	1.08130545
IMAGE:85660	KIAA1881 Hs.59	-0.793623775	-0.96961824	-0.41112914	0.73701475	1.19900022	1.22275947
IMAGE:502625	GALNT12 Hs.47	-1.065102834	-0.68131769	-0.80859559	1.42998845	1.06409159	1.13629524
IMAGE:810703	HDLBP Hs.4718	0.641401132	0.95670568	0.92400743	-2.0030698	-0.93453772	-0.61456199
IMAGE:810737	SHKBP1 Hs.265	0.857695887	1.02423584	0.77354938	-1.85668627	-1.17299592	-0.76015214
IMAGE:1593261	AI002301 127	0.703145166	1.47229847	0.36544591	-1.58087605	-1.23270902	-0.90638777
IMAGE:595078	BHLHB3 Hs.177	-1.464454167	-0.73751516	-0.4815928	1.27725106	1.15775424	1.46993969
IMAGE:2471879	PTPRC Hs.1920	0.809841566	1.40103185	-0.08377671	-0.93803182	-1.11182394	-1.16173347
IMAGE:1896838	CPD Hs.446079	-1.486986889	-1.07338134	-0.11344657	1.01370673	1.68730432	1.2813495

IMAGE:1475115	FBXO32 Hs.403	-1.036357884	-0.83634807	-0.45374864	0.33983871	1.5328005	1.50360552
IMAGE:2569769	CA2 Hs.155097	0.834491248	1.18574463	0.0138414	-0.68865741	-1.1892173	-1.16978085
IMAGE:151261	XTP3TPA Hs.632	1.244171188	1.0939526	-0.13475843	-1.35326229	-1.39370576	-0.5917696
IMAGE:1895357	TMSB4X Hs.522	0.998377601	1.17599642	0.07068751	-1.42173295	-1.28526971	-0.64291775
IMAGE:245235	RORA Hs.56949	-0.831579161	-1.0045197	-0.5608824	0.28116024	1.38675257	1.78733849
IMAGE:2028984	CXCL12 Hs.522	0.730194383	0.5966084	1.43387818	-1.55434689	-1.2559885	-0.97221651
IMAGE:2577722	STOML2 Hs.343	1.004914285	0.77170067	0.72647137	-1.78958549	-1.08464434	-0.69878182
IMAGE:23576	PDHX Hs.50231	-1.174526022	-0.85842498	-0.81288949	1.07515677	1.22164692	1.76873469
IMAGE:191664	THBS2 Hs.3711	1.192603226	-0.03296511	1.07273995	-1.85622247	-0.62672603	-0.5974336
IMAGE:1947826	ITGAL Hs.17410	0.042821607	1.60797251	0.70452402	-1.2129242	-1.1091527	-1.03476929
IMAGE:1953022	SULT1A1 Hs.567	-1.590741064	-0.62762602	-0.02309934	1.71308751	0.6951523	0.94818498
IMAGE:724615	RCC1 Hs.46972	0.590450525	1.29920318	0.7059448	-1.65400211	-0.9685145	-1.09439495
IMAGE:384087	PPM1K Hs.2910	-1.131227858	-0.48878874	-0.6833387	0.29350209	1.54353103	1.44716516
IMAGE:391984	Hs.99913 Beta	-1.242291259	-1.12837171	0.10521424	1.34524208	1.3787229	0.70051169
IMAGE:743353	POLE4 Hs.4690	0.948772514	1.12510035	0.54840461	-2.09729085	-1.12489469	-0.57412951
IMAGE:223176	MXD1 Hs.46890	1.66982845	0.44577127	0.20991216	-1.40860648	-0.78514156	-1.24204174
IMAGE:140334	ABLIM1 Hs.438	-1.15088134	-0.72072493	-0.62370478	0.74319253	1.67821039	1.16563745
IMAGE:210698	EPB41L4B Hs.5	-0.36862258	-1.03309173	-0.99497878	1.22052434	0.81102955	1.31474105
IMAGE:153743	UBE2Q1 Hs.516	1.003278474	0.9194153	0.74079763	-2.29869786	-0.76302156	-0.74831828
IMAGE:810854	RPP30 Hs.13912	1.208615414	0.93927396	0.25330559	-1.17914774	-1.19448998	-1.16482832
IMAGE:773301	CDH3 Hs.55459	0.204497672	1.20516463	0.24128231	-2.23279661	0.19873059	-0.38203033
IMAGE:365973	PPT1 Hs.3873 *	-1.358749581	-0.56750987	-0.30257792	0.58867806	1.46882111	1.2101124
IMAGE:811900	LTBR Hs.1116 L	0.78453721	1.11535699	0.75917436	-2.30504386	-0.84943683	-0.64432856
IMAGE:2489752	PIP Hs.99949 P	-1.239476482	-0.6743973	-0.00103318	1.15310819	1.02695097	0.692043
*mitoch. cont. IMA	141855	-1.092357503	-0.92404012	-0.27355816	0.99892126	1.10263067	1.26499221
IMAGE:1127165	Hs.559628 Tra	-0.896158398	-1.64476909	0.27632781	0.9546298	1.14473308	1.36661859
IMAGE:241097	SUPT3H Hs.368	0.579852974	0.84845477	0.9287193	-0.614161	-1.99825024	-0.69094951
IMAGE:713474	AA290848 36	-1.012094386	-0.99961639	-0.48886715	1.49517131	1.40982184	0.72365695
IMAGE:684073	CSNK1A1 Hs.52	1.043071747	0.45461279	0.49190775	-2.27822498	-0.36992495	-0.21326156
IMAGE:1604717	Hs.379253 Tra	-0.975521429	-1.53293034	0.34589565	0.46428112	1.23681237	1.6292146
IMAGE:682052	THBD Hs.2030 T	1.12320899	0.50014027	0.67762945	-1.44399958	-0.56962194	-1.26843918
IMAGE:272097	DCPS Hs.50424	0.569986384	1.15768724	0.78985565	-1.5867029	-1.06295773	-0.92916936
IMAGE:214982	CDC42EP1 Hs.2	1.134817164	0.86583865	0.63436629	-1.83960763	-0.89390932	-1.06042462
IMAGE:252963	LGR4 Hs.50217	-0.5737216	-1.38360092	-0.51412671	0.8524173	1.31784323	1.40838163
IMAGE:126419	DEDD Hs.51734	1.032664766	1.16877091	0.55629326	-2.38536626	-0.97195628	-0.64019755
IMAGE:811028	TMEM49 Hs.444	0.770090517	1.34149697	-0.23455444	-1.38638339	-0.86649719	-0.62130761

IMAGE:51672	ARRB1 Hs.5032[illegible]	0.886545888	0.91488082	1.19191919	-1.68655587	-1.33880896	-1.16667423
IMAGE:969538	CENTG1 Hs.302[illegible]	1.869012993	0.26601373	-0.25003202	0.12114375	-1.23611944	-1.77502436
IMAGE:1606315	LAIR1 Hs.57253	0.631061076	1.45066491	0.18504411	-0.9272492	-1.16031945	-1.26632546
IMAGE:1540694	MGC4728 Hs.57	-1.143883872	-1.25943575	-0.39510772	1.53345307	1.31823771	1.2471733
*mitoch. cont. IMA[illegible]	151651	-0.77659729	-1.23418169	-0.64790538	1.80262778	0.86916745	1.15425496
IMAGE:855079	ZNF185 Hs.166[illegible]	1.431269272	-0.00128415	1.03141568	-1.70270413	-0.8002255	-0.93131766
IMAGE:1501802	Hs.602613 Tra[illegible]	-1.254278729	-1.50069683	-0.41102436	2.17962954	1.23134257	1.23527167
IMAGE:785975	F13A1 Hs.3355[illegible]	0.864704971	0.2407942	1.20447803	-1.18555722	-1.03653394	-0.94175514
IMAGE:323181	FAP Hs.516493	-1.131694109	-1.04824194	-0.59081976	1.12021044	1.69915985	1.18905848
IMAGE:731098	C1orf158 Hs.98[illegible]	-0.871672573	-0.88579591	-0.48532874	0.92898648	1.3021147	1.01176246
IMAGE:916018	AA570170 18[illegible]	-0.867081656	-0.82231777	-0.82487571	1.48590014	1.30284782	0.77644581
IMAGE:837953	NEDD4L Hs.185	-0.968744909	-1.18461043	-1.20533352	1.71791737	0.88899377	2.12978878
IMAGE:50506	MAPK6 Hs.411[illegible]	1.60677077	0.81832764	-0.18655086	-1.23212466	-1.2389451	-0.93338929
IMAGE:290180	Hs.542001 Tra[illegible]	-0.63749343	-1.43107827	-0.65143479	1.80572908	0.98292101	1.12850094
IMAGE:1577736	EGF Hs.419815	-1.102684341	-0.58388293	-1.29303079	1.41618598	1.69571261	1.0342408
IMAGE:796127	DNMT1 Hs.2026	0.828877881	0.58793146	1.27365834	-1.70113052	-1.15154547	-0.86461095
IMAGE:511991	OVOL2 Hs.3863[illegible]	-0.954288319	-1.15367496	-0.47006363	1.37374684	1.21516035	1.16061726
IMAGE:451470	BAT2D1 Hs.494[illegible]	-1.190265129	-1.29877593	-0.28480275	1.63493423	1.22801871	1.22661209
IMAGE:1435566	FARSLB Hs.4714	-0.803216606	-1.57568547	0.00817982	2.18416987	0.90685915	0.46709932
IMAGE:857264	Hs.600913 Tra[illegible]	-0.995665808	-0.75667921	-0.60698431	0.69339168	1.48125959	1.21259663
IMAGE:345423	PREI3 Hs.1197	1.304020496	0.9267484	0.33588225	-2.2472708	-0.78461589	-0.73411947
IMAGE:182818	PRPF31 Hs.5155	0.905269909	1.09221489	0.58314933	-2.06356018	-0.81174783	-0.84985585
IMAGE:490777	CSNK2A2 Hs.82	1.816986925	-0.05730999	0.26157492	-1.42847268	-1.1167966	-0.42121477
IMAGE:840503	BHLHB3 Hs.177[illegible]	-1.128632985	-0.95593988	-0.82692142	1.30940367	1.42638932	1.42471809
IMAGE:266343	ACAD8 Hs.1479	-1.253377379	-1.46817707	-0.57138835	1.14528314	1.80886076	1.84242322
IMAGE:511043	PSME2 Hs.4340[illegible]	0.480009592	1.46825052	0.18840892	-1.78302733	-0.67125629	-0.7036177
IMAGE:753019	FLJ21963 Hs.25	-1.557863828	-0.57373875	-0.59498691	0.96759124	1.52116159	1.45238467
IMAGE:810844	ZNF672 Hs.521[illegible]	0.62321708	0.98234573	0.74637259	-2.29093886	-0.86835791	-0.18201318
IMAGE:2291894	TMC6 Hs.63222[illegible]	0.181051168	1.7014979	0.19234861	-1.96475578	-0.7432204	-0.35628318
IMAGE:357084	Hs.512144 Tra[illegible]	-0.853232587	-1.74248374	-0.06219724	1.68279224	1.10544261	1.18308618
IMAGE:770394	FCGRT Hs.1119[illegible]	0.764728736	0.39400193	1.4490819	-1.73810334	-1.14792331	-0.66342172
IMAGE:1917941	H2AFV Hs.4881[illegible]	0.819391152	1.22831489	0.32265642	-1.9520357	-1.06746908	-0.45537481
IMAGE:1518538	CCM2 Hs.14827	0.933412606	1.17726147	0.44658577	-1.66118284	-1.23662455	-0.82643593
IMAGE:897141	CHMP7 Hs.5019	1.303753849	1.2713571	-0.08606684	-1.23048213	-1.3670113	-1.15758944
IMAGE:428796	CCDC47 Hs.202	0.834941028	1.34507184	0.92180114	-1.58369943	-1.55267796	-1.28589333
IMAGE:290566	DIXDC1 Hs.446[illegible]	-1.127344748	-1.35887438	0.1608861	1.30209563	1.08363089	1.14249454

IMAGE:453109	ACTR1A Hs.1539	1.255142935	0.86557829	0.46615125	-1.84830654	-1.26395958	-0.65150478
IMAGE:1861139	AI144039::AI	-0.758742891	-1.08848973	-0.9794504	0.92848083	1.44538901	1.62129209
IMAGE:782331	MGC72075 Hs.5	-1.070706343	-1.04612916	-0.64175259	1.19358943	1.40003776	1.38381682
IMAGE:122428	T99236::T992	1.272435766	0.77394518	0.61977283	-1.94642587	-0.88263668	-1.0152249
IMAGE:200396	L3MBTL4 Hs.128	-1.361756107	-0.24406781	-0.79982821	1.41481873	1.02641299	0.96728941
IMAGE:136862	TCTE3 Hs.58480	-1.788721342	-0.75255951	-0.29456327	1.3259912	1.87603206	0.97810211
IMAGE:2477598	SLPI Hs.517070	0.827039268	0.41817593	1.07181929	-1.78040599	-0.86853315	-0.55865777
IMAGE:210387	KATNB1 Hs.2756	1.188345283	0.58126343	0.99876534	-1.94478286	-1.06611176	-0.89197512
IMAGE:139635	AMY1A Hs.4845	-0.966483154	-1.29409133	-0.45710529	1.02600107	1.44909872	1.48714354
IMAGE:1127766	EIF4ENIF1 Hs.5	-1.498133701	-0.92004019	0.10715076	0.78509679	1.53399066	1.17423494
IMAGE:48285	TP53I11 Hs.554	1.298971439	0.43638355	0.62806974	-1.42872317	-1.25476794	-0.70462855
IMAGE:1098334	AYTL2 Hs.36885	0.935641091	1.00182183	0.43928553	-2.05152316	-0.50228323	-0.90149491
IMAGE:2018283	LASS1 Hs.41235	0.844386098	0.55192424	1.26653289	-1.86938274	-0.9772399	-0.83100897
IMAGE:2506649	CTDSP2 Hs.5245	1.083252962	1.11061141	0.62431503	-1.47903652	-1.70132389	-0.89082992
IMAGE:1574926	CYP2S1 Hs.9837	1.003735469	0.96995815	0.72290119	-0.72542201	-1.62293363	-1.51581128
IMAGE:726035	JUN Hs.525704	0.884967001	0.98201474	0.82581409	-1.75773531	-0.80805388	-1.26695104
IMAGE:811059	RAB34 Hs.30185	0.963885489	0.54819945	0.97232971	-1.89889783	-1.05255644	-0.53208528
IMAGE:1436337	Hs.602089 Tran	-1.245486228	-0.67234255	-0.25304603	0.29905776	1.44983382	1.44415913
IMAGE:2576857	EIF1 Hs.150580	1.169214497	0.61511251	0.79567333	-1.96361128	-1.20410942	-0.50336147
*mitoch. cont. IMAG	142793	-1.192992167	-0.86382583	-0.55413131	0.86383382	1.14895678	1.76510053
IMAGE:593115	PDE9A Hs.4739	-1.060112116	-0.78824987	-0.63031084	1.4389169	1.06530858	1.05620605
IMAGE:278572	FAM84B Hs.1249	-1.108726868	-0.87456893	-0.5380165	0.74925686	1.32803708	1.57017038
IMAGE:2449975	SPRR2A Hs.3555	0.711221648	0.82003015	0.74976092	-1.27643882	-1.18125646	-0.77638355
IMAGE:49481	SPRYD3 Hs.343	0.778764741	0.15778435	1.82948554	-1.27878234	-1.3581222	-1.05477603
IMAGE:248129	GBF1 Hs.29024	-0.874972777	-0.80706716	-0.73765252	2.03475611	0.74916619	0.66120326
IMAGE:208027	HS3ST3B1 Hs.4	0.848150377	1.23980139	0.13951954	-0.88592173	-0.95727269	-1.46313265
IMAGE:812053	Hs.43047 Hom	0.734269652	0.91036851	1.16057848	-2.0369417	-0.97626902	-0.90446964
IMAGE:119174	HCG18 Hs.2833	-1.238848544	-0.91723531	-0.12425992	1.62233484	0.85639545	0.91072038
IMAGE:140429	FAM109A Hs.17	1.573285712	-0.08665834	0.69696203	-1.58754093	-1.00245027	-0.51115239
IMAGE:1115504	ADCK1 Hs.4132	-1.534689435	-0.63194864	-0.20020304	1.40608684	1.33336606	0.760758
IMAGE:233299	ICMT Hs.515688	-1.905827469	0.10542318	-0.75860096	1.09809457	1.48350101	1.06726205
IMAGE:47665	C1orf2 Hs.3483	0.954443771	0.93505357	0.93246821	-2.01775128	-1.68811782	-0.29396217
IMAGE:770014	TRA@ Hs.74647	0.845727206	1.00280422	0.41058144	-1.10786502	-1.13333697	-1.04482195
IMAGE:1572710	CASD1 Hs.2600	-1.574501222	-0.48355123	-0.61189829	1.14164339	1.44513071	1.26517744
IMAGE:321574	TLE3 Hs.287362	0.945742973	0.73754221	1.13572617	-2.38449323	-1.03769457	-0.52239769
IMAGE:343737	TCF4 Hs.569908	0.652527119	0.9691882	0.97108579	-2.11369122	-0.86839485	-0.66434414

IMAGE:610113	SNX2 Hs.13482	1.326625147	0.9721979	0.1058202	-1.10776494	-1.51397065	-0.95877423
IMAGE:1055460	PANK1 Hs.3763	-0.667622389	-1.50757411	0.15736326	0.52161171	1.26494931	1.27952966
IMAGE:207550	TRAM2 Hs.5201	0.925458909	0.88824421	0.92731964	-1.36487304	-1.58816121	-0.92666999
IMAGE:843263	MRPL37 Hs.584	0.838066119	0.88616983	0.69279292	-2.19415112	-1.13346435	-0.1247296
IMAGE:52096	PDGFRA Hs.746	1.310964261	0.77173562	0.81041942	-1.52203751	-1.44291157	-1.17212501
IMAGE:461327	PIK3C2B Hs.497	1.001657722	0.65508982	1.13447786	-2.3154481	-0.91990996	-0.66786057
IMAGE:1155958	AA678653::AI	-1.027838612	-0.90252757	-0.42558615	1.9479189	0.74607209	0.73354098
IMAGE:51831	Hs.31942 Trans	-0.735593036	-0.66295579	-0.37841573	2.42580609	0.3129636	-0.16792678
IMAGE:1386780	AA825954 84	-1.379073844	-0.47983435	-0.46270597	1.66348695	0.89899956	0.80425825
IMAGE:125589	USP4 Hs.63191	0.276977274	1.4046725	0.74463912	-1.71245124	-0.99727688	-0.74354545
IMAGE:767851	FBN1 Hs.59113	1.134719411	0.97223202	-0.14870214	-0.66474265	-1.06141595	-1.24839088
IMAGE:811000	LGALS3BP Hs.5	0.551694513	0.76977965	0.87518746	-1.94551009	-0.71878121	-0.41190427
IMAGE:1456160	AZGP1 Hs.5462	-0.611025291	-1.43003571	-0.03175169	1.17927183	0.88592869	1.0360806
IMAGE:1554167	JAM3 Hs.15071	0.973430587	0.50540575	1.37567896	-2.07094446	-1.34044624	-0.52646251
IMAGE:1552744	CD2 Hs.523500	0.73886324	1.11714503	0.25167701	-1.08754795	-0.94138078	-1.06967994
IMAGE:825857	Hs.535041 Tran	-1.253398123	-0.48158252	-0.45159197	0.84737369	1.31774017	1.00184707
IMAGE:127943	R09153::R09	-1.019193625	-0.92539752	-0.80840453	0.8362421	1.56902417	1.52212612
IMAGE:26616	RPA2 Hs.79411	0.816129814	1.32027019	0.26808043	-1.48035159	-1.28520047	-0.77414849
*mitoch. cont. IMA	144995	-1.164322977	-1.21961618	-0.01698897	0.95232067	1.2262634	1.41856088
IMAGE:1526537	CDKL1 Hs.2808	-0.977615531	-1.44523912	0.1675851	0.66387279	1.37012529	1.39080252
IMAGE:916305	Hs.643025 Tran	-1.010198635	-0.9099222	-0.73193153	1.28425154	1.32812248	1.18272165
IMAGE:383706	PTK2 Hs.395482	-0.354065917	-1.18181451	-1.14257799	1.60167331	0.90173884	1.22863098
IMAGE:236399	SPIN1 Hs.63218	1.028161992	0.76286301	0.72070592	-1.60974573	-1.16818647	-0.80948771
IMAGE:306446	C11orf24 Hs.30	1.284731246	0.38563401	1.04612467	-1.92176072	-1.35982494	-0.53161807
IMAGE:742739	TIAM2 Hs.5862	-0.809045924	-1.40847773	-0.53730351	0.98097163	1.52388558	1.49305766
IMAGE:1551599	AA922508 73	-0.770609638	-1.2683945	-0.1784173	1.05360023	1.27245392	0.95547368
IMAGE:1385018	ADAMTS6 Hs.48	-1.034912465	-0.80646056	-0.64136695	1.67591737	1.00590342	0.88194744
IMAGE:755444	TMSB4X Hs.522	0.866224719	1.26934562	0.1679889	-1.27240771	-1.50629389	-0.63464002
IMAGE:295324	DMGDH Hs.631	-1.216532471	-0.88716436	-0.22225249	1.34790705	1.21307198	0.87238184
IMAGE:247089	SSBP4 Hs.5152	0.849353518	1.27772201	0.67405978	-1.79637887	-1.04880265	-1.1880065
IMAGE:85060	SQRDL Hs.5112	0.586861933	1.20572478	0.56355411	-2.00251646	-0.73733041	-0.65347583
IMAGE:502622	ACLY Hs.387567	-0.9858922	-0.60318012	-0.72433003	0.64164797	1.55787591	1.08949714
IMAGE:800675	AA581501 10	1.462938358	0.55320086	0.69530233	-1.91527224	-1.33018381	-0.64788068
IMAGE:951117	SHMT2 Hs.7506	0.900142309	1.15302067	0.56431985	-1.93386543	-1.08419415	-0.76708469
IMAGE:489201	PPAP2B Hs.4051	0.601609031	0.78689236	1.14276414	-0.98959145	-1.3369977	-1.18461813
IMAGE:2244050	EBI3 Hs.501452	0.088417935	1.83694223	-0.01897777	-0.84164412	-0.87030845	-1.15236548

IMAGE:415122	HS3ST3B1 Hs.4	0.672147957	1.25172392	0.23410215	-1.36446602	-0.78140164	-1.03256784
IMAGE:1566111	ARF1 Hs.28622	-1.4020827	-1.00436117	-0.40777886	2.31108975	0.99087521	0.81742442
IMAGE:252453	DHRS7 Hs.5971	1.045902594	1.24655869	0.23424869	-1.74246466	-0.93733208	-1.05170605
IMAGE:272155	SRGAP2 Hs.497	1.233444142	-0.03035899	1.28726444	-1.10921127	-1.32867267	-0.97582434
IMAGE:377701	CSPG2 Hs.4436	1.281114474	0.47144852	0.36111948	-1.51939112	-0.95095669	-0.60989604
IMAGE:384134	Hs.349122 Trar	-0.883716895	-1.35330535	-0.08072064	1.24410579	1.20653872	1.00578965
IMAGE:1883318	PLCL1 Hs.15332	0.459084348	1.11059109	1.18535415	-0.67952031	-1.4815513	-1.67513424
IMAGE:154152	SETD4 Hs.4737	-1.019273441	-0.62060681	-0.58963362	1.13100865	1.19078148	0.87507228
IMAGE:198509	NDEL1 Hs.3721	1.427986537	0.01354258	0.90431507	-1.64239921	-1.03029929	-0.61998901
IMAGE:277487	NECAP1 Hs.555	-1.372706321	-1.14853842	-0.23383031	1.0261479	1.47786555	1.57014156
IMAGE:1605539	IDUA Hs.89560	0.777306536	1.11290182	0.46653611	-2.1159386	-1.00571583	-0.29682825
IMAGE:913992	GTDC1 Hs.4478	-1.615305393	-0.43476466	-0.56462414	1.09269407	1.34758355	1.34060763
IMAGE:841645	C17orf81 Hs.41	-1.097724075	-0.67380225	-0.54725842	1.54118929	0.47347573	1.3266975
IMAGE:999087	ZNF539 Hs.434	-1.311960626	-0.66555818	-0.39033471	1.17517028	1.10599322	1.1730331
IMAGE:278875	SEMA3C Hs.269	-1.213598211	-0.65431343	-0.72377299	0.71525774	1.39857745	1.5927485
IMAGE:590154	PLAUR Hs.4668	1.006429263	0.98652566	0.21938408	-0.61955264	-1.3284999	-1.31560995
IMAGE:1536236	Hs.408702 Trar	0.665339488	1.47491341	0.06847715	-1.38042207	-1.3441103	-0.57144342
IMAGE:782193	LATS2 Hs.78960	0.894226764	0.89821812	1.03431777	-1.25079024	-1.47087703	-1.25989728
IMAGE:2308253	OSBP Hs.50268	1.262445735	0.85583471	0.45003691	-1.57155052	-1.05607574	-1.11234054
IMAGE:810448	Hs.525339 CDN	1.203547413	0.94718163	0.36991662	-1.57603226	-1.0986615	-1.01379558
IMAGE:724465	AA250763 10	-1.21102348	-0.6272889	-0.43283536	1.92921426	0.85785003	0.51144849
IMAGE:825809	C10orf4 Hs.303	-0.595193791	-1.18159595	-0.9365535	2.56804923	0.90020252	0.36762472
IMAGE:324437	CXCL1 Hs.789 C	0.351934204	1.41774375	-0.04669175	-1.19076351	-0.71109603	-0.69429271
IMAGE:795250	ALS2CR12 Hs.1	1.488993062	0.41490695	0.49440624	-1.58039829	-1.07295603	-0.82050351
IMAGE:286450	PHCA Hs.23862	-1.195414505	0.03571229	-1.17939299	0.46850395	1.7770279	0.96826272
IMAGE:1637296	RPS24 Hs.3567	1.305899258	0.35893195	0.69203603	-2.11246238	-0.89187386	-0.35795561
IMAGE:897822	SYK Hs.371720	0.828518659	1.16482434	0.34841958	-1.92471141	-0.64243239	-0.85839517
IMAGE:1703518	AI160214 108	1.128408885	0.24458771	0.97731338	-1.40781621	-1.06641371	-0.8069067
IMAGE:1575049	Hs.444193 Trar	-0.914328705	-1.00808526	-0.59255621	1.50796565	1.11298268	1.00336787
IMAGE:1871083	TPSG1 Hs.5920	1.060705032	0.38119552	0.96525306	-0.25330111	-1.3447093	-1.77140675
IMAGE:460571	Hs.643938 Trar	1.268993149	0.61883782	0.81918487	-1.29069965	-1.13590076	-1.42912713
IMAGE:415064	Hs.527211 CDN	-1.272918576	-0.43700118	-0.70001897	1.2581998	1.14317889	1.03852466
IMAGE:897896	RSN Hs.524809	-1.030813735	-0.75946489	-0.73632042	1.77079664	0.92110229	0.91391953
IMAGE:382195	MYO7A Hs.3704	0.475054158	0.99045313	1.1552046	-1.76869344	-0.93612586	-0.9374474
IMAGE:243614	TSPAN32 Hs.27	0.821811277	1.2923938	0.45524161	-1.14095562	-1.55984822	-1.03955578
IMAGE:145292	COL4A1 Hs.174	1.415680621	0.65489106	-0.09439283	-1.33801415	-0.8722717	-0.77758063

IMAGE:22893	HDHD1A Hs.185	0.978421404	1.13224735	0.31081681	-1.04503069	-1.03958269	-1.46991076
IMAGE:1129873	MAPK6 Hs.4118	1.575699496	0.54690448	-0.04303391	-1.29701273	-1.05570155	-0.7773993
IMAGE:247082	C20orf121 Hs.28	1.331021458	0.1030053	1.17611817	-1.58772498	-1.19558358	-0.83787929
IMAGE:1555836	TRAF1 Hs.53125	0.539928375	1.64938791	-0.06009393	-0.77251654	-1.32724631	-1.10909415
*mitoch. cont. IMAG	148609	-0.912636562	-0.82850467	-0.82741205	1.10136921	1.14689513	1.39771257
IMAGE:1323737	Hs.552449 Tran	1.315597461	0.4953308	0.81925776	-1.89937762	-1.10784642	-0.73324056
IMAGE:1564149	LOC283130 Hs.4	-1.166012961	-1.04839952	-0.41737784	1.61145403	1.28929548	0.9425915
IMAGE:683569	ITPR1 Hs.56729	-0.884196979	-0.63363583	-0.99659155	1.40181559	0.6716088	1.44906426
IMAGE:206913	FLJ21963 Hs.25	-1.209297873	-0.8586088	-0.48490575	2.64937788	0.89227948	0.16633483
IMAGE:1658252	AI038595 947	-1.159235328	-1.63110251	0.4405943	1.60301882	1.28630544	0.74543962
IMAGE:788263	ZBTB25 Hs.4359	-1.275371524	-1.13392049	0.71696628	0.81753965	0.97741299	0.92277753
IMAGE:469369	POLR2E Hs.2430	0.711085682	1.05497579	0.72392425	-2.05539558	-0.92743604	-0.57116589
IMAGE:758662	PSMD9 Hs.1311	1.019292052	0.67805876	0.89146498	-2.24869379	-0.85938681	-0.55227684
IMAGE:1703181	AI097617 237	-0.74041101	-1.62244407	0.14195552	1.50127017	1.18113719	0.68443086
IMAGE:1114740	CAMK1D Hs.156	-1.159644229	-0.6628041	-0.52332142	1.68004105	1.17749319	0.53029002
IMAGE:814522	ANKRD39 Hs.63	1.304317193	0.82421519	0.91740933	-2.42300679	-1.15883254	-0.75772091
IMAGE:230261	RALA Hs.6906 V	0.770755675	1.67747891	-0.44738664	-1.0063744	-1.22000029	-0.88674389
IMAGE:769579	MAP2K2 Hs.465	0.891717779	0.79082299	0.9339975	-1.97393453	-1.09254646	-0.62482703
IMAGE:940821	AA503283 309	-1.340232877	-0.50390385	-0.46006403	1.52479623	0.87587042	0.94061848
IMAGE:853789	TYRP1 Hs.27027	0.865328341	0.69415455	0.758178	-1.3873854	-0.95278182	-0.94677962
IMAGE:195753	DBT Hs.270570	-1.12915703	-0.57084718	-0.81323536	1.97627132	0.92263169	0.66764749
IMAGE:300973	CD8A Hs.85258	0.518069652	1.49902341	-0.06642742	-0.62836547	-1.30076997	-1.01346987
IMAGE:1635302	EDAR Hs.17197	-0.895839976	-1.20718772	-0.26257753	0.84890249	1.21119805	1.42266292
IMAGE:1658001	LOC390927 Hs.5	-0.96303342	-1.07101194	-0.57622431	1.5942317	1.02773502	1.1493817
IMAGE:234468	OSBP2 Hs.5175	1.559750438	0.45452604	0.26090402	-1.53093064	-0.82666171	-0.98995239
IMAGE:2504881	STAT5A Hs.4370	0.572786104	1.48144211	0.3910549	-1.10040177	-1.22675495	-1.24300424
IMAGE:26939	LOC643564 Hs.5	-1.205924268	-0.17953728	-0.99803914	0.9662553	1.62095899	0.73852696
IMAGE:462681	NRG3 Hs.12511	-1.001571556	-1.01392039	-0.40590881	1.95092188	0.97323118	0.60647088
IMAGE:1915185	Hs.158667 Tran	-0.822898799	-1.2396319	-0.42487616	1.42441241	1.36558784	0.834891
IMAGE:842980	DRG1 Hs.11524	1.484709657	0.59191724	0.76381309	-1.9255575	-1.27595108	-0.86819812
IMAGE:345897	Hs.642794 Full	-0.571424084	-0.94166032	-0.76944952	1.91561236	0.32087921	0.99494693
IMAGE:782701	PODN Hs.58614	0.484233906	0.27406582	1.5319375	-1.61802205	-0.85786185	-0.57648756
IMAGE:470128	MYO1E Hs.3703	1.226754516	0.69614884	0.80158745	-1.77915783	-1.09692532	-1.01025619
IMAGE:298417	TFF3 Hs.82961 T	0.91672589	0.92249172	0.64081401	-1.05942724	-1.33415203	-1.16626466
IMAGE:645670	Hs.633967 Tran	0.329943983	-0.66225519	-0.29433701	2.48677046	-1.44716518	-0.1732172
IMAGE:1010155	ALOX15B Hs.11	-1.85596128	-0.60854812	0.00546354	1.52853562	1.00223991	1.15915913

IMAGE:1704155	IER5 Hs.15725	1.032337439	1.09589962	0.23611199	-2.04994298	-0.68165793	-0.75589467
IMAGE:853988	UBE2L3 Hs.108	1.009148757	1.219725	-0.01821798	-1.22212121	-1.28394476	-0.81447218
IMAGE:712683	NCK1 Hs.47769	1.049638601	1.03116611	0.07798575	-0.56638081	-0.83446278	-1.81784567
IMAGE:823726	AA489655 10	-0.827942257	-1.58661941	0.10247939	2.72491783	0.46000253	0.3088229
IMAGE:1435192	TMEM41A Hs.53	-1.023836963	-1.03611471	-0.86913737	1.21470298	1.6382852	1.32336103
IMAGE:186132	SELE Hs.89546	0.831663825	1.13653085	0.08354667	-1.33337804	-1.29272911	-0.43061819
IMAGE:785745	PNPLA4 Hs.264	0.93514704	1.04197506	0.54085453	-1.92086367	-0.54059709	-1.18029056
IMAGE:768344	TYRP1 Hs.27027	0.797475888	0.65904936	0.83568161	-1.69022673	-1.08147961	-0.45768355
IMAGE:2144577	JPH2 Hs.441737	-1.386662448	-0.76370462	0.00974302	1.40667345	0.86596006	0.94073833
IMAGE:1739821	CRISPLD1 Hs.43	-0.962461008	-0.60073817	-0.95615647	1.68968057	0.65682002	1.19349377
IMAGE:2244196	BCAP29 Hs.303	-1.132322832	-0.63399016	-0.58665513	0.7076621	1.60357615	1.07155014
IMAGE:1591379	AA976270::A	-0.803722429	-0.46964771	-0.36862029	2.13482698	0.06094747	0.17505613
IMAGE:399101	Hs.601242 Tra	-1.632072272	-0.66707098	0.11769414	0.66687336	1.1401069	1.49461694
IMAGE:323322	Hs.171939 MR	-0.872697438	-1.43908501	0.14088272	1.30683803	1.2180758	0.76665645
IMAGE:999365	PRR5 Hs.10233	-0.901331098	-0.89486917	-0.54529081	1.12979861	1.15953815	1.08657715
IMAGE:2013094	KIF1C Hs.43512	1.496839656	0.0627702	0.77971613	-1.53268757	-1.1996993	-0.58167308
IMAGE:48182	PGM5P1 Hs.178	-0.580043677	-0.86844945	-0.91892046	1.83195442	0.74121527	0.74822058
IMAGE:242084	FCHO2 Hs.1657	-0.991239778	-1.37474848	0.087171	1.30905809	1.17139023	0.95957032
IMAGE:283309	REV1L Hs.4430	-0.997576966	-1.02301859	-0.93185277	1.18953956	1.76265759	1.24351215
IMAGE:229537	GANAB Hs.7684	1.000236163	1.0358963	0.40292883	-1.80955993	-1.13424604	-0.61405376
IMAGE:712591	Hs.598937 Tra	1.503650691	0.83187328	0.12695745	-1.81910542	-0.83950615	-1.0033712
IMAGE:785744	MFGE8 Hs.3745	0.47059289	-0.01991789	1.83049173	-1.53310135	-0.96224829	-0.46877753
IMAGE:324225	RARRES3 Hs.17	0.170740143	1.2867666	0.89495299	-1.73353018	-0.93264069	-0.63878049
IMAGE:295492	ZNF260 Hs.181	-1.415390656	-0.77889045	-0.90357094	1.6851235	1.42568733	1.31007451
IMAGE:1204881	COBLL1 Hs.470	0.633357698	0.92667251	0.93672901	-1.8822358	-1.05257391	-0.57614686
IMAGE:377252	ADORA2B Hs.16	1.142540934	0.9116687	0.35209702	-1.54907711	-0.97972271	-0.9926359
IMAGE:753321	Hs.593989 Tra	-0.972630308	-1.19024172	-0.13498639	0.70805782	1.5241006	1.18088261
IMAGE:2505791	ARD1A Hs.433	1.010731962	0.97889731	0.3241646	-2.32049927	-0.60938663	-0.45976375
IMAGE:1612722	CPSF3L Hs.644	1.273126459	0.54688516	0.65852225	-2.36018075	-0.80266325	-0.39032624
IMAGE:511882	OVOL2 Hs.3863	-0.886177452	-1.16749728	-0.43354645	1.48171523	0.97572193	1.16500799
IMAGE:210697	EPB41L4B Hs.5	-0.582958418	-0.85694153	-0.90525833	0.8394916	1.26301857	1.18891267
IMAGE:1869136	ABHD5 Hs.1938	-0.895145386	-0.91310795	-0.33212665	0.6149318	1.27505618	1.23755034
IMAGE:324891	DEGS1 Hs.2998	1.45771593	0.20329758	0.61321416	-1.42825445	-1.01602531	-0.81375821
IMAGE:1574120	ANXA4 Hs.4229	-1.3068242	-0.88106229	-0.24241944	1.81534727	0.76508741	1.00441936
IMAGE:267427	C2orf24 Hs.497	1.204944485	0.31182551	0.99992308	-1.70913116	-0.94360061	-0.87232707
IMAGE:1635163	HAAO Hs.36880	0.89326697	0.669279	1.13858713	-1.90844923	-1.25248446	-0.60611917

IMAGE:2502722	LOH11CR2A Hs.	0.735477997	0.16410118	1.54387039	-1.68800621	-0.91876371	-0.67243682
IMAGE:855687	AA663922::AI	-0.892126822	-0.94206443	-0.77527281	1.97587345	0.96696723	0.77753722
IMAGE:2014862	HOXC6 Hs.5490	1.134370684	0.90769196	0.78415206	-1.44381646	-1.2398056	-1.35966198
IMAGE:825647	NUP210 Hs.475	0.551529682	1.42039059	0.58303782	-1.37715057	-1.04862249	-1.26090462
IMAGE:1631663	LASP1 Hs.54801	0.846603984	1.15235138	0.54894991	-1.63691913	-1.01643178	-1.03126952
IMAGE:135338	TAF1B Hs.63190	0.674974973	1.3229608	0.36577859	-2.08712839	-0.52583971	-0.84115879
IMAGE:810567	ARHGEF2 Hs.56	-0.098230869	1.62520716	0.90964743	-1.64418977	-1.06858178	-0.71475217
IMAGE:753252	TMEM101 Hs.51	1.057186282	1.37603574	0.04446753	-0.95978436	-1.45275017	-1.29288292
IMAGE:379768	CRLF1 Hs.11494	1.398512464	0.26269221	0.50036771	-0.81575307	-1.36663076	-0.9348828
IMAGE:279936	ATP10B Hs.1093	1.395394195	0.20828588	0.6315457	-1.00586939	-1.19156999	-0.99751286
IMAGE:149274	CASC4 Hs.5128	-1.019111026	-0.64042146	-0.72401897	1.77158099	0.97847643	0.64426503
IMAGE:781311	LOC643988 Hs.5	1.242718746	0.34367062	0.56743371	-2.30277744	-0.69947494	-0.0866238
IMAGE:42408	AGPAT3 Hs.2487	-1.13771132	-0.53479263	-0.44695798	0.59295025	1.25586964	1.2186335
IMAGE:1871165	TMEM41A Hs.53	-0.939148906	-1.11445348	-0.78488088	1.10402588	1.54754645	1.40993236
IMAGE:280633	Hs.633606 Tran	-0.949634485	-1.19520468	-0.64057541	1.69264096	0.91300549	1.41233156
IMAGE:50238	VPS37C Hs.5237	1.109289545	0.66368583	0.82572354	-2.03724966	-1.01692938	-0.63743845
IMAGE:324712	RPS6KB2 Hs.534	1.405908278	0.56543043	0.35718404	-1.93126903	-0.92989972	-0.54231936
IMAGE:1632154	HMHA1 Hs.4655	0.125744462	1.82856064	-0.26941713	-1.331787	-0.65144647	-0.61145277
IMAGE:2562146	MAPK13 Hs.178	1.096073604	0.65240087	0.56082682	-1.82276059	-0.85377933	-0.64720531
IMAGE:2466317	RNF8 Hs.485278	1.151522296	0.84659849	0.64590318	-2.00776555	-0.94330426	-0.85349034
IMAGE:80221	CPA3 Hs.646 Ca	-1.59423447	-0.49297028	0.09712691	0.58254763	1.12942114	1.29742971
IMAGE:236129	TMEM127 Hs.35	1.399306412	0.86477353	1.10346302	-1.98099729	-1.35420609	-1.44024532
IMAGE:460386	AA676776 664	1.465237403	0.22594018	0.97521098	-1.67273432	-1.32970397	-0.75334181
IMAGE:1862098	AI053677::AI	-1.312106464	-0.57420587	-0.46577966	1.46574535	0.98588611	0.96006162
IMAGE:375834	LOX Hs.102267	0.59809473	0.63035055	1.09268391	-0.81917442	-1.0014899	-1.38785849
IMAGE:2313673	TMED5 Hs.4828	-1.045812002	-0.51340235	-0.92369287	0.73999421	1.416443	1.33700041
IMAGE:45641	MAP2K3 Hs.514	1.376535239	0.65737357	0.33610321	-2.5240767	-0.44724532	-0.49967019
IMAGE:415136	KDELR2 Hs.487	1.124777326	0.93303767	0.57028698	-1.74535799	-1.21677841	-0.83744482
IMAGE:2143529	TRAT1 Hs.13870	0.644490523	1.23350705	-0.07447943	-0.0927046	-1.10147244	-1.52972003
IMAGE:782277	DCBLD1 Hs.583	-1.126249462	-0.41675741	-1.20495874	0.79048766	1.53735475	1.49286632
IMAGE:898051	TNC Hs.143250	1.087810256	1.02073246	-0.47836054	-0.88858919	-0.98719356	-0.68908065
IMAGE:701070	AA287269::AA	-1.017146541	-0.66051996	-1.02822816	0.7951176	1.4742864	1.53238094
IMAGE:249606	RABEPK Hs.190	1.271864331	0.82251957	0.60894583	-1.72626709	-1.53729032	-0.63920073
IMAGE:180079	ARHGAP1 Hs.13	1.008786506	1.03523512	0.70727234	-2.21364614	-1.13255917	-0.60391755
IMAGE:1603560	PEPD Hs.36473	0.978530605	0.87750121	0.65528134	-1.87073357	-0.86015959	-0.87225624
IMAGE:415415	Hs.633768 Tran	0.814929976	0.89501071	0.67307267	-1.87864279	-0.90623385	-0.62137523

IMAGE:1208795	ANKH Hs.15672	-1.05587656	-0.91342813	-0.57551647	1.37796326	1.58402944	0.71135991
IMAGE:1584597	C1orf164 Hs.45	1.174988166	0.47459901	0.75282257	-1.99535956	-1.13315636	-0.28689306
IMAGE:627055	C22orf13 Hs.98	0.895347429	1.20986773	0.54536365	-1.98271683	-1.04962055	-0.80718993
IMAGE:767853	ADPRH Hs.9988	0.897687433	0.96492927	0.85527335	-1.17827495	-1.72215796	-0.96258383
IMAGE:1570436	EXOSC7 Hs.115	1.385721404	0.32416094	0.63816063	-1.46261801	-1.26334898	-0.63655731
IMAGE:323917	EHD1 Hs.52377	0.72830324	1.14316378	0.82006063	-1.3696709	-1.2305706	-1.23203481
IMAGE:789069	LOX Hs.102267	0.883355776	0.87000369	0.58226617	-0.64495768	-1.69643467	-1.01647955
IMAGE:1915445	GRHPR Hs.1557	1.130205498	0.8589519	0.45362153	-1.21749437	-1.33955849	-0.99371015
*mitoch. cont. IMA	149015	-1.232549999	-0.77539911	-0.35980739	1.82336152	0.83075351	0.80756787
IMAGE:196275	CNTN1 Hs.1434	0.95752393	0.71559898	0.97093759	-1.72448963	-0.76158041	-1.23728631
IMAGE:730018	SUGT1 Hs.2819	1.277110578	0.91020355	0.04720765	-1.02886186	-1.33843967	-0.97267923
IMAGE:2306655	AI654233 110	0.376732939	1.11145122	0.77769031	-0.6346848	-1.40938774	-1.16031658
IMAGE:510790	YARS Hs.21326	1.108974995	1.18195245	-0.14283928	-1.17436977	-1.04115696	-1.04231533
IMAGE:725931	CLCNKA Hs.591	-1.242137558	-1.05286769	0.06124359	1.44915095	0.99932775	0.91747468
IMAGE:280777	KENAE Hs.4823	-1.486910099	-0.86924114	-0.39924476	1.97345599	1.27591605	0.78391078
IMAGE:309583	RARRES1 Hs.13	-0.933523013	-0.75984552	-0.38643892	0.55340171	1.44113909	1.02856066
IMAGE:45515	BCAP29 Hs.303	-1.254288898	-0.54719177	-0.34896789	0.67218096	1.58193981	0.88431009
IMAGE:1695734	HK3 Hs.411695	0.591945032	1.26851613	0.27268805	-1.3316576	-0.91144352	-0.88845068
IMAGE:240353	C6orf71 Hs.310	-1.124878272	-0.48599441	-0.87392389	1.42525815	1.37426742	0.70918832
IMAGE:345670	APCDD1 Hs.293	0.907268528	0.36121151	1.17270287	-1.57409656	-1.0377432	-0.75675889
IMAGE:1009313	PPAP2A Hs.4351	-1.534473746	-0.82590237	0.22870422	0.92198793	1.51650843	0.81618754
IMAGE:1739623	Hs.541185 CDN	-1.020882648	-0.81472563	-0.64583994	0.86656053	1.51427921	1.17987261
IMAGE:1536968	CD3E Hs.3003 C	-0.326690544	1.82568053	0.54102939	-1.07428537	-0.81206231	-1.03842404
IMAGE:742633	NAG Hs.467759	0.441027351	0.77229342	1.27893564	-2.06709523	-0.80292545	-0.54863003
IMAGE:786308	CD4 Hs.631659	0.670417596	0.82742348	0.84799666	-1.47356988	-1.00988369	-0.82330386
IMAGE:340850	C9orf125 Hs.13	1.343845088	-0.07937106	1.3198583	-1.60777214	-1.29680056	-0.64196121
IMAGE:783681	HUWE1 Hs.1369	0.77586892	0.82068679	0.84676264	-1.92481497	-0.75140174	-0.77707015
IMAGE:714498	TRIM8 Hs.33681	0.489050589	0.91324238	0.9855478	-2.18793707	-0.81630326	-0.33113389
IMAGE:39821	ABCA5 Hs.4214	-0.796132016	-0.84863434	-0.92310686	0.86435486	1.35504963	1.40162861
IMAGE:80050	STEAP4 Hs.5210	1.158430962	0.88807405	0.04909548	-1.33352474	-1.03693504	-0.76066709
IMAGE:504678	C14orf147 Hs.2	-0.802705498	-1.13855879	-0.44304488	0.53709407	1.23218285	1.69642563
IMAGE:810213	IL1R1 Hs.55740	0.944845865	0.9232544	0.30669809	-1.16116175	-0.8300926	-1.19426866
IMAGE:1010172	AA228893::A	-0.680299571	-1.54465167	-0.31906001	1.79741042	1.03737668	0.90146476
IMAGE:2125819	BAX Hs.631546	0.769042105	1.32868181	0.13838606	-1.45542922	-1.08490914	-0.77923008
IMAGE:246869	ZNF207 Hs.500	1.30840565	1.18534017	-0.19406338	-1.39555705	-1.10662069	-0.99586176
IMAGE:502515	RBM25 Hs.5311	-0.755548868	-0.74422462	-0.9941131	0.65866143	1.49665606	1.33698411

IMAGE:1010124	CCDC9 Hs.2277	-1.275715297	-0.94875748	-0.17246622	1.50062362	0.89224854	1.15941979
IMAGE:208387	KIAA1407 Hs.47	-1.099473061	-0.93415885	-0.49838462	1.43681562	1.23458495	1.00202806
IMAGE:1550894	GCNT2 Hs.5198	-1.095004678	-0.79959398	-0.56175049	1.09652614	1.08137687	1.36618309
IMAGE:1593829	TIA1 Hs.516075	-1.384229066	-0.65498291	-0.01752743	-0.15076905	1.939011	1.2924853
IMAGE:1127373	SLC15A1 Hs.436	-0.929500724	-1.02317062	-0.39107226	1.39438012	1.17549487	0.84797236
IMAGE:855755	FBL Hs.299002	0.851463764	0.92072832	0.63273359	-1.66885465	-1.01266413	-0.76768634
IMAGE:1557288	GABPB2 Hs.596	0.450565321	0.76567843	1.27429761	-1.90324083	-0.81439929	-0.69959752
IMAGE:284619	GYPC Hs.59138	1.094941372	1.19396788	-0.04926491	-0.66435456	-1.38004614	-1.32738204
IMAGE:245426	RANBP2 Hs.590	-0.85396489	-0.93047874	-0.63754001	1.56350832	1.15780276	0.75227937
IMAGE:52339	TSPAN5 Hs.591	1.303697299	0.23474728	0.87798445	-1.7694173	-1.07623356	-0.55949658
IMAGE:841308	MYLK Hs.55660	-1.047443827	-1.01888494	0.00733121	0.49679887	1.20442472	1.38910555
IMAGE:322175	Hs.562735 Tran	-0.332541696	-1.40813987	-0.36155274	0.91725959	1.01578766	1.12991602
IMAGE:192225	SLIT3 Hs.60411	1.084325144	-0.64186788	1.29677967	-0.45336696	-1.19606484	-0.63522868
IMAGE:1073215	CYP11B1 Hs.184	-1.045227393	-0.70096601	-0.61564906	1.34218251	1.82104795	0.22562098
IMAGE:814455	COMMD8 Hs.239	1.291953012	0.73978446	0.31273785	-1.02144281	-1.06807211	-1.3490136
IMAGE:684940	TMF1 Hs.267632	1.033441715	1.26687872	0.34013381	-1.62686474	-1.17166258	-1.0771206
IMAGE:953848	Hs.430095 Tran	-1.120417415	-0.65293775	-0.66579029	1.53823144	0.98963586	0.9644033
IMAGE:284139	TRIM35 Hs.1042	1.195545831	-0.60513027	1.8030192	-1.45348653	-0.98463125	-0.70127957
IMAGE:930020	AA503162::AI	-0.887487755	-0.93378591	-0.55612524	1.42212883	1.1021755	0.90276273
IMAGE:366074	LOH12CR2 Hs.6	-0.825998643	-1.75978746	-0.01215994	1.7487532	0.78080138	1.36432449
IMAGE:127032	BIRC3 Hs.12779	0.259255166	1.267821	0.62142199	-1.02055375	-0.75847944	-1.28835854
IMAGE:229907	SLC14A1 Hs.101	-0.975856317	-0.74275438	-0.6263766	1.72444313	0.84186829	0.79457538
IMAGE:1323011	AA746175 135	-0.715679407	-1.40133335	-0.20585574	1.03210718	1.29353904	1.1071926
IMAGE:853506	PISD Hs.420559	1.00862763	1.09125603	0.4668124	-2.02727224	-0.85218286	-0.85388589
IMAGE:757368	MOSPD3 Hs.521	0.866489289	0.57399872	1.35072367	-1.11350934	-1.39137537	-1.34425189
IMAGE:1456899	CYP3A5 Hs.1502	-0.62845381	-1.63153837	0.01304944	1.20683778	1.22666404	0.94017466
IMAGE:39914	FSTL4 Hs.48339	-0.727512381	-1.15068047	-0.60673502	1.31691284	1.2457884	1.01300681
IMAGE:293729	UNQ1887 Hs.50	1.214383099	0.52172695	0.48615912	-1.55289554	-1.34045104	-0.31851739
IMAGE:1944078	SLC25A37 Hs.59	-1.281309389	-0.82404128	0.09634797	0.45685814	1.59271212	0.98802078
1239845	SYT17 Hs.25832	-0.979838675	-1.46439125	0.40956316	0.63590715	1.63332175	0.88516204
IMAGE:951449	REG4 Hs.17148	-1.242412428	-0.98572192	0.06402731	1.11052001	1.21396245	0.93768493
IMAGE:1588935	PHLDA3 Hs.268	0.713594428	0.66070913	1.19837635	-1.60636251	-1.28657171	-0.66649156
IMAGE:1655795	GNG12 Hs.4311	1.234331374	1.35474009	-0.10790894	-1.20774749	-1.09367608	-1.44729746
IMAGE:358162	SAC3D1 Hs.236	0.620480294	0.58917562	1.11587681	-2.00265601	-0.75242546	-0.45424841
IMAGE:365707	FAM3D Hs.6126	-0.703065445	-1.16107417	-0.29085759	0.84979604	1.0226646	1.28732078
IMAGE:51511	GALNTL1 Hs.21	0.653636607	0.097452	1.64441746	-0.69882855	-1.30266266	-1.18066352

IMAGE:136360	ANKDD1A Hs.20	-0.940880551	-0.89523699	-0.56368219	1.02813687	1.57736579	0.8532764
IMAGE:25499	PLXNA1 Hs.432	0.929531972	0.72208054	0.86163878	-1.96229379	-0.96511249	-0.62706096
IMAGE:449278	AA777699 24	-0.599035211	-1.61110725	0.18716668	0.73729081	1.35083234	0.9931322
IMAGE:121159	C15orf39 Hs.17	0.71649716	0.99990859	1.04678047	-1.66988154	-1.02403051	-1.18917216
IMAGE:814526	RBM38 Hs.2363	1.100999288	1.40921886	-0.00730693	-1.63990748	-1.33168791	-0.78459817
IMAGE:1637226	FLT3LG Hs.428	0.399007057	0.85394667	1.49246057	-0.96006356	-1.54103375	-1.243909
IMAGE:399898	CSPG2 Hs.4436	1.025326759	0.66901577	0.33861831	-1.58837631	-0.8498408	-0.52656956
IMAGE:42706	THRA Hs.724 Th	1.338044079	1.1321871	-0.03738552	-2.33955275	-0.97860938	-0.34045274
IMAGE:827129	POLRMT Hs.254	1.014157426	0.86407812	0.72271309	-0.91920299	-1.71075032	-1.09079136
IMAGE:123815	INOC1 Hs.2929	0.750466552	1.35255031	0.05064791	-1.87661945	-0.81167803	-0.52953771
IMAGE:383938	AA702714 93	-1.029632431	-0.71379842	-0.60685873	1.27046293	1.16732605	0.93593069
IMAGE:1646553	SLC7A2 Hs.448	1.451343529	0.67450979	0.06075604	-1.99950622	-0.99953938	-0.26567942
IMAGE:214068	GATA3 Hs.5241	0.787973801	0.47674617	0.87018977	-2.00448368	-0.69635505	-0.28397844
IMAGE:767801	ZC3HAV1 Hs.13	0.754961398	0.69002559	0.49831035	-2.14040257	-0.34539132	-0.30457453
IMAGE:1896822	COBL Hs.99141	-1.140742564	-0.76418208	-0.40521787	1.49193811	1.06357332	0.80839787
IMAGE:815501	LMNB2 Hs.5382	0.563009832	1.32691458	0.17115499	-1.72640563	-0.81979923	-0.5026255
IMAGE:289936	SLC7A11 Hs.39	1.086956591	0.88377635	0.02714976	-0.86369052	-0.85769632	-1.26864977
IMAGE:344970	ITGA4 Hs.44095	0.522255744	1.38106082	-0.07912198	-0.81881514	-0.80917768	-1.12807956
IMAGE:428828	BCL2L10 Hs.283	1.537268391	-0.27147576	0.58910567	-1.34482261	-0.68510698	-0.60514145
IMAGE:220369	SORBS1 Hs.386	-1.027518632	-0.83211684	-0.4784396	2.06972188	0.99531734	0.32246349
IMAGE:2421798	PLAUR Hs.4668	0.798112435	1.17551328	0.10684442	-0.76267084	-1.14550191	-1.18582135
IMAGE:246546	TMEM140 Hs.56	0.959535565	1.0245248	0.48511419	-1.61844792	-1.33992265	-0.6241127
IMAGE:298136	CDADC1 Hs.388	-1.375293842	-1.15803704	-0.40970804	1.89683503	1.2329948	1.18230154
IMAGE:290654	ZNF331 Hs.185	-1.205075784	-0.66911725	-0.97301126	1.38354868	1.52014751	1.12385744
IMAGE:239724	SLFN11 Hs.462	0.697603882	1.16444163	0.43722677	-0.90460301	-1.07572206	-1.35927668
IMAGE:754080	ICAM3 Hs.7551	0.457794648	1.71412373	-0.01770839	-1.40592699	-1.01551397	-0.81430111
IMAGE:744436	Hs.112954 Tra	-1.334187162	-0.71278795	-0.52463566	1.52132558	0.93562361	1.26930806
IMAGE:281901	HDAC11 Hs.404	0.322789334	0.51190603	1.5624686	-1.48674872	-0.98887006	-0.72951002
IMAGE:665508	DERL1 Hs.2415	0.591448555	1.11302336	0.69904903	-1.73859449	-1.06095793	-0.63096673
IMAGE:289978	UBL4A Hs.7648	1.253074873	0.78966239	0.59884549	-1.60836091	-1.27306835	-0.93123349
IMAGE:996847	CLN8 Hs.12767	-0.975009468	-0.94986882	-0.75307019	0.86893994	1.43723546	1.52247978
IMAGE:1492104	TUBB2C Hs.433	0.887439023	1.13717764	0.22763576	-1.53718703	-1.18350306	-0.60077961
IMAGE:234522	PKLR Hs.95990	-1.182897405	-0.71820254	-0.24131275	0.83502205	1.5197558	0.79851301
IMAGE:593780	Hs.598465 Tra	1.173449495	0.88272414	0.71460904	-1.88769894	-1.21460653	-0.87521627
IMAGE:1569429	CHD3 Hs.1916	-0.904292105	-0.39604572	-0.57275908	2.32887168	0.18315969	0.15442422
IMAGE:1558693	C19orf30 Hs.32	-0.615632722	-0.61416147	-0.45788152	1.99102194	0.23287256	0.19314869

IMAGE:767823	PALMD Hs.4839	-0.179567406	-1.67603071	-0.53136755	1.09365158	1.06672586	1.28722917
IMAGE:288667	DDX28 Hs.4583	1.11245293	0.99726202	0.56392477	-1.54160055	-1.1548882	-1.17298963
IMAGE:212634	UBE2V1 Hs.420	-0.411554967	-1.14735268	-0.48703112	0.70001161	1.35995388	0.88718489
IMAGE:745138	H2-ALPHA Hs.50	1.259940564	0.40293866	0.60647661	-1.36944842	-1.33731084	-0.54565533
IMAGE:2118808	ALOX15B Hs.11	-1.400767184	-0.31732281	-0.00750383	0.8332029	1.02683977	0.72647211
IMAGE:1897716	ERN1 Hs.13398	-1.020146382	-1.63576416	0.2849633	2.42863234	0.80457402	0.39445532
IMAGE:937364	C11orf59 Hs.53	-1.027085692	-1.28434236	-0.49542191	1.37276723	1.25788124	1.45577099
IMAGE:771016	PTPN18 Hs.5915	1.229502888	0.75423159	0.81857602	-2.25350452	-1.14575681	-0.59956041
IMAGE:842794	MICAL-L1 Hs.51	1.034784226	0.41226355	0.78345744	-1.86341469	-0.76791741	-0.51856136
IMAGE:176606	NELL2 Hs.50532	1.356872341	0.58842681	-0.14029601	-1.0489927	-0.54851792	-1.1450681
IMAGE:971279	KALRN Hs.8004	1.089560421	0.98350294	-0.17949315	-0.98719666	-1.45991003	-0.43812191
IMAGE:377246	CHP Hs.406234	-0.929975312	-0.89299891	-0.55586111	0.36485135	1.41541624	1.64902011
IMAGE:772416	UNC45A Hs.389	0.797174695	0.53212005	1.04859796	-1.82251182	-0.90239356	-0.57978419
IMAGE:188036	DST Hs.631992	1.090762807	0.52484546	0.8966791	-1.64522497	-0.47824063	-1.42079567
IMAGE:1011697	RPL21 Hs.38112	0.91002215	0.83154073	0.65493873	-1.58686916	-1.20908072	-0.63506784
IMAGE:1586056	CAPZA3 Hs.131	-0.89086584	-0.89247955	-0.78231696	1.31043409	1.3658535	0.9766267
IMAGE:855395	SCP2 Hs.476365	-0.494934817	-0.61123505	-1.37798584	0.62507793	1.5471726	1.20948658
IMAGE:2516692	CTSW Hs.41684	0.582760545	1.2384733	0.29259474	-1.0085599	-1.22225661	-0.86677769
IMAGE:1901399	Hs.210944 Tran	-0.926583871	-1.23162979	-0.14472512	1.14403087	1.35516803	0.91902799
IMAGE:345761	Hs.595153 CDN	-1.189783526	-1.0620637	-0.44283441	1.55013961	1.35946784	1.02170641
IMAGE:810859	IL32 Hs.943 Int	0.662236514	1.15843361	0.24177476	-1.13470065	-1.02940386	-0.86911914
IMAGE:244307	SERPINE1 Hs.41	0.835808914	0.76354033	0.31632532	-0.05766461	-1.43820716	-1.29855874
IMAGE:1656893	FUT8 Hs.118722	-0.815533291	-0.97840369	-0.33035095	2.1072862	0.76693477	0.22962319
IMAGE:187055	PDCD6IP Hs.475	-0.941004924	-1.21161216	-0.49675804	0.99355496	1.54698435	1.30933388
IMAGE:49164	VCAM1 Hs.1092	0.545594808	1.08019041	0.45761365	-1.13161777	-0.94815731	-0.9309198
IMAGE:299781	Hs.49943 **Tra	0.784175934	0.44614256	0.99460731	-0.64373525	-1.09878377	-1.34621786
IMAGE:491500	NFATC2IP Hs.51	0.423870522	1.38898154	0.43267985	-1.80643155	-0.59074404	-0.86295233
IMAGE:950746	Hs.606068 Tran	-1.117433064	-0.67896462	-0.51453895	1.51560423	1.0269517	0.79521427
IMAGE:2516736	NASP Hs.31933	1.024666276	0.97236109	-0.20724533	-0.49679189	-1.50179863	-0.73789387
IMAGE:1947827	Hs.577789 Tran	1.43329164	0.78034428	0.13457825	-1.82964981	-1.01968022	-0.63934667
IMAGE:1662217	ABCA12 Hs.134	1.215568794	0.06028316	0.87712741	-1.45513688	-0.68884266	-0.86620766
IMAGE:120309	C17orf75 Hs.46	-1.026053039	-1.19372173	-0.16951679	1.51069394	0.7710177	1.2598465
IMAGE:112495	T91047 11171	-1.147327463	-0.82688076	-0.26849318	0.86246267	1.60073319	0.83373296
IMAGE:281909	Hs.573828 Tran	-0.729972998	-0.56398828	-0.64745488	2.46594423	-0.11037575	0.39469204
IMAGE:428411	MYSM1 Hs.4774	-0.561440396	-1.04620611	-0.95983866	1.10696983	1.52767722	0.97662103
IMAGE:1553439	NALP9 Hs.4430	-0.90701455	-0.72209029	-0.75702042	2.20407937	0.84026291	0.34559051

IMAGE:51944	DDHD1 Hs.5132	-0.794766882	-0.80033183	-0.89243169	1.17324167	1.67162735	0.66331867
IMAGE:1605562	Hs.489491 Trar	-0.585748795	-1.34277722	-0.60418923	1.27509745	1.28763371	1.08529441
IMAGE:76415	T60688 79844	-0.906263445	-0.93248949	-0.8747922	1.20309701	1.14802232	1.50050031
IMAGE:595637	ARID5B Hs.5352	1.262003983	0.67846153	0.39919478	-1.59464474	-1.04444757	-0.77059943
IMAGE:287558	TOLLIP Hs.3685	1.131993283	0.43366025	0.68899788	-2.26661208	-0.55154307	-0.39157251
IMAGE:1891563	FZD4 Hs.591968	-1.031288507	-0.9826027	-0.57136992	1.39642926	1.09701155	1.2416084
IMAGE:213698	SRD5A2L Hs.59(	-1.045321363	-0.74650933	-0.53792988	1.16272403	1.12248602	1.07494834
IMAGE:308989	RAB25 Hs.63246	1.05833486	0.34468374	0.82538836	-1.77051759	-0.77578218	-0.58996359
IMAGE:1631863	HLA-DQB1 Hs.4(	0.634788512	0.29164276	1.2601265	-1.37746787	-0.80856833	-0.77876883
IMAGE:1493227	Hs.125407 Trar	-0.982644557	-1.09816009	-0.2986983	1.22257057	1.37851654	0.89349274
IMAGE:40364	SUSD4 Hs.4978	-0.87761377	-0.99051595	-0.21764089	0.49414767	1.37218829	1.20790972
IMAGE:968121	CCDC108 Hs.14	-0.896689975	-1.1523811	-0.17194786	1.56949015	0.94410303	0.77494827
IMAGE:1506462	ABCB9 Hs.5119	-1.228931885	-1.23869172	-0.87562575	1.51992651	1.35400926	1.92203183
IMAGE:229398	H79296::H794	-1.078189245	-0.99602364	-0.09654731	1.45780468	0.88523289	0.88896589
IMAGE:286639	ZNF256 Hs.288	-1.087130449	-1.17734444	-0.11812604	1.73329503	0.78693256	1.0241423
IMAGE:1878270	KRT75 Hs.14594	-0.505233235	-1.37464799	0.14354337	0.76343609	0.84458147	1.03237506
IMAGE:951125	PECI Hs.15250 I	-1.059477603	-0.65395105	-0.55727875	0.75770653	0.89383591	1.61519897
IMAGE:898044	CPXM Hs.29341	0.851887193	0.71983155	0.65745999	-1.03592779	-1.06496282	-1.0785125
IMAGE:240780	NINJ2 Hs.50442	0.864805335	1.22066499	0.63658846	-1.41823851	-1.1382178	-1.36748475
IMAGE:1574509	RAPGEF1 Hs.12	0.812462612	1.29687891	0.27070724	-1.51248848	-1.38792429	-0.60198356
IMAGE:190717	FSTL5 Hs.59170	-1.349381212	-0.75994348	-0.44374043	1.65234423	1.11110818	0.95521016
IMAGE:1584403	TRIM46 Hs.287	0.506438061	1.27251767	0.65390839	-0.83348306	-0.84066506	-1.81167097
IMAGE:281053	C2orf18 Hs.516	1.122817815	0.97102792	0.75314769	-0.62930239	-1.4790353	-1.97386552
IMAGE:187614	SMAD1 Hs.5190	0.502170079	0.97088517	1.08192059	-1.6357168	-0.86688754	-1.05937927
IMAGE:878231	BAT5 Hs.388188	0.724928724	1.32681639	0.37784017	-1.69942464	-1.0448718	-0.80562145
IMAGE:545749	BCAS1 Hs.4005	-0.863569409	-0.82538584	-0.78502035	2.20557143	0.75527761	0.55385927
IMAGE:452660	AA779153 10	-0.53207273	-1.30512939	-0.15978492	0.77284182	1.2877891	0.8949034
IMAGE:1881612	CYBASC3 Hs.22	0.740130146	0.53721011	0.81229589	-2.14262563	-0.54878101	-0.23997361
IMAGE:744994	KCTD17 Hs.517	0.736430184	0.72544599	1.05319731	-1.23783003	-1.24923669	-1.02224419
IMAGE:767262	KCNMB4 Hs.525	0.882100594	0.47113842	0.88062231	-1.69830086	-1.28684592	-0.14548963
IMAGE:219540	PRPF39 Hs.2743	-0.68847422	-1.18364538	-0.70580259	0.75664392	1.72795756	1.20583116
IMAGE:1553567	ANKDD1A Hs.20	-0.728837745	-0.88797528	-0.61498499	0.91589938	1.31601279	0.96203859
IMAGE:823955	MGC2749 Hs.36	1.191390374	1.16169981	0.20922637	-1.21859308	-1.52143688	-1.05113827
IMAGE:202901	VAV2 Hs.36992	0.400263935	1.66358832	0.28311931	-0.59804945	-1.45940698	-1.39222109
IMAGE:868469	ALCAM Hs.5912	-1.046261235	-0.72747299	-0.43108608	1.7147896	0.96507719	0.51959216
IMAGE:229622	ZNRF3 Hs.1344	-1.274556834	-1.1144702	-0.41148106	1.25278193	1.5245232	1.32058674

IMAGE:358227	SPTBN5 Hs.5911	-0.69214038	-0.98530406	-0.42652228	0.80643758	1.24421555	0.99866638
IMAGE:302080	NDE1 Hs.567518	0.731822982	1.10233746	0.57955676	-2.03866099	-0.59974516	-0.83728047
IMAGE:1892819	Hs.604307 Tran	-0.977478397	-1.25417363	0.30860106	1.31964545	0.87693307	0.7651482
IMAGE:148225	GALNT3 Hs.1709	-0.717863881	-1.0139415	-0.55858931	0.45752631	1.69877479	1.1396436
IMAGE:283258	ATP11B Hs.4784	1.369204203	0.76104565	0.02042516	-1.48351761	-0.7399813	-0.99740732
IMAGE:459947	MOSC2 Hs.3690	-1.076729983	-0.7742944	-0.91747646	2.3315145	1.01657718	0.57529048
IMAGE:729964	SMPD1 Hs.4981	0.896456272	0.57404153	0.76175856	-2.13617677	-0.38288542	-0.63888272
IMAGE:840514	ROS1 Hs.1041 V	-0.672870144	-1.00390805	-1.0615476	0.95985611	1.28875797	1.59348771
IMAGE:786048	E2F4 Hs.108371	1.089990676	0.90079583	0.62055095	-2.00718815	-0.93409861	-0.82058169
IMAGE:1455561	STK40 Hs.47176	1.139249226	0.7032361	0.50213616	-1.76313001	-1.15182181	-0.47644637
IMAGE:261253	PVRL3 Hs.29391	0.805902081	0.683529	0.92687661	-0.73197789	-1.30363499	-1.35712951
IMAGE:843407	TTC17 Hs.19118	1.101097842	0.53887978	0.63198596	-1.72556986	-0.81241313	-0.71196589
IMAGE:950603	HIPK2 Hs.39746	-0.798211424	-0.56840007	-1.06402655	0.60038215	1.51962756	1.25994073
IMAGE:1591477	MS4A6A Hs.523	0.54225116	1.21659492	0.22847488	-0.58847883	-1.03660778	-1.29877612
IMAGE:462849	SLC41A1 Hs.202	-0.855943389	-0.85073376	-0.55313131	0.68585583	1.33127539	1.23429865
IMAGE:35300	WWC1 Hs.48404	-0.74383058	-0.79964237	-1.06550943	1.69556736	1.32957733	0.62195152
IMAGE:953542	AA528311::AI	-1.084624311	-0.55270007	-0.63780795	1.5745457	1.00747646	0.67122436
IMAGE:418081	KIAA0831 Hs.41	-0.917891616	-0.5213341	-0.83538636	0.78584191	1.35273286	1.06449676
IMAGE:122063	SLC7A7 Hs.5131	0.527282042	0.976996	0.90575045	-1.24750485	-1.11813198	-1.02296829
IMAGE:462468	AA699895 222	-0.944961978	-0.89724293	-0.40318295	1.62757637	1.01029325	0.62941643
IMAGE:1602597	AA987596::AI	-1.111471433	-0.90394003	-0.26785627	1.32039826	1.05386997	0.98366929
IMAGE:878174	FADS2 Hs.50274	-1.449060995	-0.32089867	-0.00504068	0.59077887	1.13513799	0.93532348
IMAGE:156097	HLA-DQB2 Hs.5	0.813705802	0.65455727	0.73390738	-1.42300352	-0.79761704	-0.89915828
IMAGE:1688537	FBXO31 Hs.5675	-0.580217695	-1.26297602	-0.33643632	0.23089521	1.57055625	1.3838845
IMAGE:731293	ZNF659 Hs.2102	-0.803066253	-0.63468583	-0.86686256	0.93750756	1.27724251	1.02545626
*mitoch. cont. IMAC	150724	-1.195905972	-0.84851681	-0.41121517	1.75111207	0.73497749	1.09456358
IMAGE:435025	Hs.477083 CDN	-1.584786765	-0.44544946	-0.16380528	0.81870225	1.16169975	1.26970893
IMAGE:825229	MRPL11 Hs.4184	0.433122608	0.60729621	1.55838963	-1.65352991	-1.11834194	-0.73674341
IMAGE:2125289	WISP1 Hs.49297	1.150266614	0.78716741	0.03311482	0.18606415	-1.91978688	-1.21382184
IMAGE:795456	JMJD2C Hs.1571	-0.945194481	-0.95480785	-0.35877924	0.95472681	1.26047187	1.08327886
IMAGE:2566688	ARL3 Hs.182215	1.092614819	0.97274632	0.66156062	-1.19522011	-1.56230508	-1.1674673
IMAGE:1601661	B3GALT4 Hs.534	1.214377643	0.26604283	0.95152397	-1.41566232	-1.13648767	-0.85788568
IMAGE:135238	SRG Hs.556029	-0.717692823	-1.6233849	-0.22281883	0.94525656	1.74742138	1.09746219
IMAGE:112559	T91244 10013	-0.589171202	-0.44540527	-0.5932788	2.45722889	-0.07510531	-0.08866038
IMAGE:898035	CTSB Hs.520898	0.847729802	1.06937648	0.19157133	-1.73001052	-0.72891221	-0.65620086
IMAGE:79726	C17orf28 Hs.110	-1.184406214	-0.84879616	-0.25733281	1.13979209	0.87985881	1.35181867

IMAGE:809467	SARS2 Hs.6434	0.187179155	1.22379976	1.18209386	-1.89721175	-0.92990008	-0.76697386
IMAGE:771220	RELA Hs.502875	0.318578382	1.09150723	1.32273637	-1.85633948	-1.24254304	-0.66966636
IMAGE:785986	AA449783::A	-1.660380114	-0.58509933	-0.5420881	1.50894106	1.50643458	1.03045365
IMAGE:796367	LZTS2 Hs.52322	-1.031981113	-0.74677503	-0.33607827	0.59926805	1.24487092	1.24409308
IMAGE:346134	CARHSP1 Hs.63	0.42868696	1.45682127	0.01047593	-1.59608056	-1.01630557	-0.22897114
IMAGE:824426	PDAP1 Hs.6322	1.145031628	0.67969476	0.63011681	-1.55718393	-1.00269373	-0.96485793
IMAGE:1570219	AA931593::A	-1.024390242	-0.96369906	-0.46268617	1.25499037	1.20436205	1.10113925
IMAGE:25422	C9orf91 Hs.522	-1.884662607	-0.28421706	-0.72433958	1.26534477	1.36972165	1.52367755
IMAGE:1894503	AI291677 243	-1.004570357	-0.81218632	-0.40643089	1.6954522	0.72741069	0.81031072
IMAGE:2254132	PLCB1 Hs.4311	-1.341554318	0.00374686	-0.88326491	0.88576919	1.08534683	1.1396763
IMAGE:562080	C9orf86 Hs.567	1.042887661	0.78721447	0.53393821	-1.54720166	-1.19964591	-0.66572837
IMAGE:156211	ATP6V1B1 Hs.6	-1.133088669	-1.15714412	0.31264381	0.82953527	1.17168108	1.04332807
IMAGE:1521924	PGBD3 Hs.1334	-1.036029741	-1.46479809	0.151518	1.90414628	1.16509826	0.49259971
IMAGE:36393	ACAT2 Hs.5710	-1.069010906	-0.73347482	-0.31973941	0.61324504	1.37028101	1.1198768
IMAGE:129569	HSPC049 Hs.45	-1.279439921	-0.82182302	-0.19820783	0.73257896	1.27775622	1.38931903
IMAGE:214131	NIT2 Hs.439152	-0.407045188	-1.16604032	-0.91530085	2.38792533	0.28374687	0.83208213
IMAGE:347183	Hs.374278 CD	0.953997167	-0.63073287	1.42330988	-0.63110153	-0.94724271	-0.68568956
IMAGE:289847	ZNF141 Hs.601	-0.690199756	-0.88130303	-0.59191807	2.22935776	0.40250147	0.46529254
IMAGE:1573108	BCKDK Hs.5135	1.141639653	0.7678434	0.41564425	-1.55114706	-1.20850049	-0.62413234
IMAGE:782811	HMGA1 Hs.518	0.910878136	0.71389508	0.73522542	-1.68766619	-1.02726056	-0.64126486
IMAGE:740780	KLK11 Hs.5777	1.091225206	-0.33595938	1.2360021	-1.17711773	-1.14509756	-0.35568609
IMAGE:196445	R92500 3178	-1.070829648	-1.21647955	-0.35945801	1.59306334	1.07422463	1.21299835
IMAGE:269752	ZAK Hs.444451	1.655109256	0.12308231	1.15995815	-1.06355505	-1.51665791	-1.53702208
IMAGE:360025	SAMSN1 Hs.570	0.696630487	1.2302209	0.0332749	-1.16129125	-0.81483325	-0.95574621
IMAGE:161204	Hs.503584 Tra	1.281759036	0.72949153	0.0824522	-1.18418355	-0.72907422	-1.20668334
IMAGE:76744	TRSPAP1 Hs.53	0.889781876	0.73210418	0.91782732	-2.32079776	-0.71285921	-0.54645626
IMAGE:825451	VDP Hs.292689	-1.303313354	-0.74759842	-0.30676237	0.89456256	1.56702433	0.99823375
IMAGE:1466633	Hs.447707 Tra	-1.369840451	-1.13112441	-0.4790769	2.18815319	0.72755275	1.43458747
IMAGE:1893919	AI280322 881	-0.571497076	-1.08799928	-0.79484938	1.95276619	0.80454827	0.72549181
IMAGE:82738	DNASE1L3 Hs.4	0.764410505	1.00594263	0.2899724	-1.25917583	-0.96480855	-0.79401083
IMAGE:320763	SP3 Hs.531587	1.314706484	-0.13075069	0.79843006	-0.83406051	-1.32902008	-0.61089068
IMAGE:2542973	DIO2 Hs.20235	1.223540544	0.64434111	0.38935209	-0.9024569	-1.32758576	-1.05846992
IMAGE:1859838	ZNF213 Hs.115	0.994641344	0.56081736	0.88730345	-1.72009424	-1.16650278	-0.55572035
IMAGE:127486	LDHD Hs.38092	-1.302386556	-0.50580403	-0.02319365	1.04107027	1.12596442	0.57424325
IMAGE:1898026	MBOAT2 Hs.467	-0.104135759	-0.79637307	-1.45446415	0.87435112	1.01395943	1.28053288
IMAGE:795882	GNPDA1 Hs.278	0.640389851	1.29535407	0.49916319	-1.78425698	-0.84018746	-0.90312543

IMAGE:234562	SFRS2IP Hs.210	1.354445511	-0.81101576	0.9958706	-0.86945137	-0.6736045	-0.51692701
IMAGE:1492238	MAPBPIP Hs.632	0.615884639	0.88159041	0.7857621	-2.05576507	-0.71634665	-0.45630347
IMAGE:826204	FLII Hs.513984	0.979133517	0.7976408	0.39768462	-1.70410189	-0.8672188	-0.59094655
IMAGE:1881689	Hs.604374 Tran	-1.073290672	-0.9933204	-0.31877116	1.73891392	1.12414246	0.63532417
IMAGE:901655	PCOLCE Hs.2020	0.553114497	0.52084914	1.22100745	-1.15654627	-1.0773953	-0.90326319
IMAGE:1631807	GMPPB Hs.5674	1.124156676	0.89517263	0.17044197	-1.51976098	-1.06294937	-0.65933608
IMAGE:898109	TAX1BP1 Hs.345	-0.633582709	-0.67710409	-1.23044738	0.04296754	1.66154847	1.79957342
IMAGE:1914684	AI310519 862	0.737867157	-0.11658113	1.44375066	-1.74898691	-0.77327764	-0.21435282
IMAGE:855402	RP5-821D11.2 H	-1.093769812	-0.88851794	-0.42242516	1.35572951	0.95885578	1.18687778
IMAGE:134976	MLLT1 Hs.10095	0.834034919	0.51371702	1.04768969	-2.24238576	-0.6599606	-0.42889367
IMAGE:49435	C9orf68 Hs.1796	-1.009827567	-1.07215308	-0.23449814	1.23124963	1.22373099	0.96111303
IMAGE:809946	IFRD2 Hs.31517	1.260146172	0.77365885	0.10377011	-1.73993075	-0.84875485	-0.59173456
IMAGE:878545	RPL18 Hs.51551	0.647598426	0.65085359	0.89108484	-1.86815657	-0.72071058	-0.47266693
IMAGE:1323772	Hs.543999 Tran	-1.008533836	-0.79351903	-0.58544018	1.19755075	1.15501698	1.08231179
IMAGE:229596	PSD4 Hs.51630(	0.838714231	0.60254493	1.12393408	-1.52683921	-0.97502056	-1.06494657
IMAGE:1873533	SLC26A3 Hs.165	-1.196779229	-0.64040466	-0.18556397	0.98228477	1.35407266	0.65137338
IMAGE:32609	LAMA4 Hs.2138(	0.923513789	0.82761028	0.90094826	-1.87376579	-0.87524106	-1.00386459
IMAGE:813391	Hs.597796 Tran	0.033734294	-2.17960171	0.70658844	0.69152073	0.53190515	1.1121397
IMAGE:304886	N92502::W38	-0.677877884	-1.18752682	-0.36009678	1.28626915	1.00646189	0.955497
IMAGE:770785	MAN1C1 Hs.197	0.304762123	1.43889687	1.03006507	-1.52785023	-1.20187138	-1.17334823
IMAGE:1568233	FAM62C Hs.4777	1.254772518	-1.39294938	1.11896854	-0.83452387	-0.13676256	-0.22015896
IMAGE:1505707	Hs.42197 CDNA	-0.738264993	-1.25668207	-0.32198217	1.62482059	1.36381391	0.40626381
IMAGE:321266	MAFB Hs.64267(	1.551013117	-0.10070292	0.66350986	-1.60583503	-0.69527363	-0.70374397
IMAGE:1592276	MRPL28 Hs.513	1.049618299	0.68764353	0.52790249	-1.78853932	-0.89737318	-0.57985835
IMAGE:1374851	HNRPR Hs.3737	-0.898161206	-0.82545848	-0.731182	1.39888945	1.06632714	1.03419043
*mitoch. cont. IMAC	141632	-1.04809744	-0.91152292	-0.21399355	1.37743271	0.82280692	1.00668285
IMAGE:502664	TMEM158 Hs.35	1.561752606	-0.26580132	0.63093161	-0.02861339	-1.40297335	-1.30100471
IMAGE:1070007	ZC3H7B Hs.592	-1.267691869	-0.70481844	-0.73785666	1.63324766	1.0355224	1.21231623
IMAGE:26474	FOS Hs.25647 V	0.841846694	0.73196341	0.47232491	-1.14602041	-1.09186364	-0.71323724
IMAGE:824421	NUDT5 Hs.5559	0.898407194	1.23163199	0.18388584	-1.80481065	-0.41182177	-1.20828365
IMAGE:23185	TNC Hs.143250	1.0351759	1.03294439	-0.5001052	-0.75717552	-1.0621118	-0.65776161
IMAGE:563465	ATP11A Hs.2918	1.08970102	0.56548519	0.77694852	-1.71063329	-0.77104305	-0.97228862
IMAGE:300015	FNTA Hs.370312	0.579684951	-0.88340464	1.69435174	0.04542473	-0.93116074	-0.77662414
IMAGE:1861366	RPL14 Hs.44652	-0.875760969	-0.89483898	-0.46969617	1.96325859	0.60872003	0.67104152
IMAGE:242182	PKIB Hs.486354	-1.125901365	-0.63944894	-0.47938831	0.90119924	1.3747227	0.97133889
IMAGE:1845273	TNPO2 Hs.6434	-1.019297307	-1.23293539	0.67501699	-0.05044862	1.57796655	1.00705987

IMAGE:47853	STARD4 Hs.938	0.932339952	0.50877479	0.86626379	-1.94514998	-0.77611013	-0.52324173
IMAGE:68500	RASSF5 Hs.4975	0.731491356	1.08475781	0.55360678	-1.33194087	-1.21554904	-0.8688923
IMAGE:951008	Hs.593086 Trar	-1.02119636	-0.99742906	-0.24733295	0.36934937	1.67710565	1.29064929
IMAGE:80374	PDHA1 Hs.5303	-1.106040538	-0.63136228	-0.31585756	0.46480343	1.52643415	1.00968859
IMAGE:380880	Hs.386402 Trar	-1.097752293	-1.26020662	0.39682749	1.04152637	1.05710418	0.94227345
IMAGE:1699243	DMRT3 Hs.1891	0.608922583	-0.51395343	-0.07189236	2.04457329	-1.25540582	-0.84175575
IMAGE:730633	CROP Hs.13029	-0.3245574	-0.52739427	-1.35689032	0.66600714	1.14641025	1.16162302
IMAGE:41208	BMP1 Hs.1274 E	0.748322908	0.91648598	0.59841754	-2.14576356	-0.42009013	-0.67938157
IMAGE:647420	NOL12 Hs.6327	1.519783596	0.63665132	0.54793335	-1.94689511	-1.0289556	-0.94371835
IMAGE:195555	Hs.406337 Full	-1.119018043	-0.80169262	-0.38351857	1.71963172	0.97956799	0.66126451
IMAGE:206272	ME1 Hs.21160 M	-0.765452861	-0.67207682	-0.69560464	0.7571465	1.26281087	1.00584294
IMAGE:197913	SFPQ Hs.35593	-0.430294422	-1.23315903	-0.5702988	0.28982068	1.61208424	1.30614875
IMAGE:815051	SLFN11 Hs.4628	0.674892353	1.1560646	0.55104966	-1.03622714	-1.14271608	-1.25630428
IMAGE:1091502	COL5A2 Hs.445	0.825127196	0.76037585	0.77877887	-1.45769847	-0.99762313	-0.89640656
IMAGE:1898143	GFOD2 Hs.4610	1.580780458	0.17019278	0.55336734	-0.74263828	-1.56330368	-1.01222708
IMAGE:299603	Hs.635764 Full	-0.734923197	-0.55023322	-0.30788394	2.04927818	0.25530787	0.00800349
IMAGE:139883	KIAA1754 Hs.52	1.346155632	-0.40254438	1.28300813	-1.7617336	-0.77808985	-0.4793536
IMAGE:49920	PTDSS1 Hs.292	1.419820425	0.55095137	0.34453497	-1.53409433	-1.11646114	-0.73627093
IMAGE:949928	MYST3 Hs.5918	0.583947248	0.37902059	0.78260064	-1.97732236	-0.32431699	-0.1210632
IMAGE:239943	KIAA0256 Hs.99	-0.943462789	-0.83957108	-0.31869665	1.98043834	0.61851894	0.47396434
IMAGE:814297	PEPD Hs.36473	0.647326163	0.91471702	0.84438512	-1.64636071	-0.67099179	-1.08119367
IMAGE:869233	DLC1 Hs.13429	-1.186391817	-0.53332262	-0.99466508	1.03733874	1.6574549	1.12810937
IMAGE:1659177	Hs.562573 Trar	-0.615823612	-1.38439446	-0.11022455	1.31700022	0.97570471	0.84540288
IMAGE:1558394	LPL Hs.180878	-0.426234089	-1.15836109	-0.39411787	0.7955118	1.36777369	0.70625463
IMAGE:842879	ALDH5A1 Hs.37	-1.304709585	-0.78168298	-0.18645093	0.67237728	1.44303153	1.24724369
IMAGE:1519408	Hs.370221 CDN	-0.619232888	-1.11503127	-0.30297093	0.96168707	0.90108555	1.11733729
IMAGE:811941	ZNF688 Hs.3014	0.722378664	1.14311233	0.57836331	-1.24613154	-1.30471353	-0.97034556
IMAGE:1869329	AI269958 109	1.285942688	0.612195	0.49691153	-1.44891315	-1.13498505	-0.88444774
IMAGE:222025	STCH Hs.35234	0.619772389	0.44526082	1.50687288	-1.32094172	-1.05594267	-1.10425652
IMAGE:682749	Hs.561775 Trar	-0.645556963	-1.31428183	-0.28806254	1.49739681	0.76630068	1.03613887
IMAGE:1323636	MGAT1 Hs.5198	0.501393359	0.88744921	0.75232966	-2.04406587	-0.51528471	-0.46432534
IMAGE:148960	GPBP1 Hs.4442	-1.298799917	-1.29487245	-0.10092237	1.64994259	0.91354245	1.45317647
IMAGE:453589	SLC2A1 Hs.473	1.733248823	-0.18273954	-0.02566644	-1.30117404	-0.74659658	-0.24591025
IMAGE:242687	B3GNT2 Hs.173	-0.561309475	-0.60979518	-1.01178579	0.72113752	0.91133373	1.38891797
IMAGE:839736	CRYAB Hs.4087	-0.317773294	-1.64249511	0.3990523	0.43120404	0.89503237	1.11535082
IMAGE:160664	RET Hs.350321	0.897690193	1.24641371	0.22277606	-1.73839138	-1.08654664	-0.66968791

IMAGE:1573570	RERG Hs.19948	-0.970491143	-1.03411073	-0.60361822	1.27455116	1.22484836	1.26202605
IMAGE:2015148	GIT1 Hs.514051	0.929334021	0.03681635	1.35551121	-2.06255255	-0.62336781	-0.45769422
IMAGE:307553	KRAS Hs.50503	0.534454325	1.51425608	-0.36804819	-0.7397965	-0.6340186	-1.23919027
IMAGE:1883267	Hs.147865 Tran	-0.724621398	-1.42590393	0.22234887	0.99671038	0.78816771	1.16297382
IMAGE:1557660	POLR3D Hs.148	0.708255174	1.05904587	0.64051628	-1.35354738	-1.24528612	-0.85276342
IMAGE:878605	LOC642412 Hs.5	0.684817437	-0.05925784	1.54031705	-1.32063369	-1.08507217	-0.45802984
IMAGE:343990	COPZ2 Hs.4084	0.440185449	0.66760319	1.13257428	-1.5817103	-1.0210844	-0.47460611
IMAGE:2542220	PMM2 Hs.45985	1.453420587	0.738937	0.1684933	-1.63610172	-1.18359545	-0.67945583
IMAGE:744925	NEK9 Hs.7200 N	-0.645226955	-1.3005128	-0.64945461	2.25177064	0.78266205	0.69599521
IMAGE:32050	LONRF2 Hs.2138	-1.140434069	-0.73975535	-0.48073073	1.30471793	1.22579636	0.89068325
IMAGE:2243734	C1orf95 Hs.592	0.847359797	-0.73537966	1.69075092	-0.2835301	-0.95336311	-1.04451565
IMAGE:155575	EPB41L1 Hs.437	-1.503583669	-0.95326592	0.11735224	0.91750174	1.20829464	1.41278771
IMAGE:122946	KIAA1276 Hs.18	-0.891887603	-1.10797836	-0.605413	0.52659386	1.66552444	1.56444689
IMAGE:713660	GPM6B Hs.4957	-0.273019295	0.52025976	1.44534517	-1.93417004	-0.25290662	0.0095345
IMAGE:307157	BBS2 Hs.33373	-0.642319697	-0.66665253	-1.22728103	1.75762774	0.88894556	0.85098634
IMAGE:299600	DLX5 Hs.99348	1.364254825	-0.4503377	0.99115424	-0.74002703	-0.7297995	-1.13999195
IMAGE:278188	ABHD5 Hs.1938	-0.667503378	-0.7474805	-0.28612069	-0.1271017	1.42668897	1.18053941
IMAGE:127119	ITFG1 Hs.42217	-1.012657758	-1.15445451	-0.57437688	1.31184225	1.47619758	1.18059968
IMAGE:854450	STAU1 Hs.37018	0.904134697	0.90188588	0.45822667	-0.92334933	-1.10646718	-1.25199769
IMAGE:271926	Hs.594950 Tran	-0.352196903	-2.03527038	-0.1954872	1.25860342	1.1891236	1.37783291
IMAGE:2488470	Hs.477083 CDN	-1.215471852	-0.56138277	-0.19933563	0.19576953	1.62769427	1.09098767
IMAGE:1160966	CAPN13 Hs.445	-0.73549064	-0.79578885	-0.60361261	1.17586546	0.98913551	0.88643404
IMAGE:825404	Hs.600133 Tran	-0.632332739	-1.44408149	-0.33478661	0.65060677	1.63890945	1.24358838
IMAGE:1663707	LOC147804 Hs.4	-1.129963375	-1.35467494	0.08733125	1.52901642	1.11811528	0.9706617
IMAGE:2298710	Hs.605886 Tran	1.236093747	0.87597904	0.08912839	-1.60363583	-0.82713848	-0.84874536
IMAGE:2109825	CYP4F3 Hs.1062	-1.035006153	-0.79946058	-0.2366685	0.51591858	1.44156872	1.09004842
IMAGE:2490795	WISP2 Hs.5921	0.735142131	0.25969969	1.19113798	-0.96253587	-0.81047227	-1.20817707
IMAGE:1667445	CEBPZ Hs.1354	-1.212671619	-1.08976503	-0.09948908	0.68911276	1.50385664	1.38504693
IMAGE:701175	AA287667::AA	-1.681456748	-0.93707372	-0.65249323	2.30186236	1.36773311	1.07381677
IMAGE:61647	ACADL Hs.4712	-0.858779332	-1.0350804	-0.12432981	1.27796374	0.83850739	0.87973073
IMAGE:839941	FBXO18 Hs.498	-1.211094678	-0.72744227	-0.30931697	0.82746121	1.40667397	1.06031644
IMAGE:132789	R27412::R274	-0.812283879	-0.75837239	-0.45437499	1.8210864	0.62877031	0.47409643
IMAGE:27548	NUP153 Hs.121	0.844560048	0.52511431	1.47504507	-1.83815371	-1.20766869	-0.85249546
IMAGE:1682730	Hs.146933 Tran	-1.163009111	-1.16816782	0.06579423	1.18884414	1.23656215	0.98911631
IMAGE:280784	SLC27A3 Hs.438	0.336274702	0.69196801	1.18471518	-1.79856918	-0.3571407	-0.86754816
IMAGE:245398	Hs.498418 CDN	-0.617240107	-0.84403883	-1.15651707	1.37682465	1.38438461	0.87635547

IMAGE:897460	EEF1D Hs.3333	0.613163963	0.80123118	0.73775849	-2.0074351	-0.77912112	-0.2572346
IMAGE:954120	AA525112::AI	-0.966292212	-0.70621014	-0.58575108	1.60766474	0.84281813	0.7904595
IMAGE:342211	MBOAT2 Hs.467	0.163204599	-1.52417351	-0.38119733	0.41397476	1.20423563	0.89973964
IMAGE:510668	SEPP1 Hs.64349	-1.172658149	-0.45942856	-0.43449046	0.67687587	1.21553878	1.09882848
IMAGE:265	Hs.465730 Tran	-0.982767386	-0.87816778	-0.40746956	1.35649069	0.99767669	0.94657232
IMAGE:813286	LOC146795 Hs.1	1.510227154	0.2184843	0.17082106	-1.41409676	-0.76988031	-0.62261644
IMAGE:290229	DTNA Hs.643454	-0.861688281	-1.36843241	-0.55193201	1.08205017	1.60682858	1.34621731
IMAGE:294995	SIDT2 Hs.41097	-1.010445223	-0.53488352	-0.95134629	1.48579199	1.28494797	0.73643603
IMAGE:869187	EPAS1 Hs.46841	0.829973518	1.1219148	0.29917118	-1.6044187	-1.21958701	-0.47779074
IMAGE:843049	MCM4 Hs.46018	0.478510111	1.51231814	-0.12513871	-1.48156614	-0.79236079	-0.55589206
IMAGE:550353	PIGF Hs.468415	1.07126076	0.98821182	0.05228638	-1.28004729	-1.08566463	-0.78885494
IMAGE:1884443	ZNF626 Hs.1286	-0.923253277	-1.16965652	0.36372982	1.38172272	0.59402209	0.70895762
IMAGE:52755	LOC349114 Hs.4	-0.663278817	-1.13416815	-0.6176769	0.47007745	1.48125034	1.51693879
IMAGE:840466	MARCO Hs.6772	1.308663555	0.09990295	0.71953741	-0.98602222	-1.00896921	-1.0172801
IMAGE:236282	WAS Hs.2157 W	0.698386269	1.36116477	0.03478952	-1.43252844	-0.75747626	-0.94280877
IMAGE:241245	KIAA0146 Hs.38	-0.757900636	-0.78101034	-0.46607346	2.44306186	0.08228932	0.36560711
IMAGE:301135	TXNL2 Hs.42644	1.130769306	0.73781055	0.16700691	-1.35959862	-1.0506585	-0.6013713
IMAGE:51975	ZNF592 Hs.7934	0.944294796	0.85403218	0.77618068	-0.55237219	-1.38758345	-1.72776068
IMAGE:242011	PKN2 Hs.44083	-1.3174968	-0.96815115	0.11912736	1.1818799	1.03029738	1.06738545
IMAGE:207750	H58930::H589	-0.757954417	-0.79944849	-0.60995889	1.84674954	0.70667108	0.54513235
IMAGE:149809	GATA2 Hs.36772	0.5673074	0.80448649	0.92629858	-0.85259293	-1.1919266	-1.17104453
IMAGE:980809	Hs.573633 Tran	-0.641580449	-0.90272961	-0.73698694	1.64747898	0.716961	0.87325878
IMAGE:297305	RBP5 Hs.246046	0.254222196	1.44240693	0.21017543	-0.38277287	-1.19957424	-1.22531586
IMAGE:454317	LOC402571 Hs.5	-0.318369179	-1.3612519	-0.09105915	1.08556417	0.82102233	0.72666907
IMAGE:139331	RMND5A Hs.752	1.234394869	0.50279755	0.59304249	-1.58400715	-0.6706057	-1.09247889
IMAGE:252412	C4A Hs.534847	1.230558659	0.16647418	0.77087417	-2.04868368	-0.90745308	-0.10300521
IMAGE:83358	Hs.76704 Trans	-0.869457021	-1.10427146	0.26788777	0.15906031	1.23830358	1.22836912
IMAGE:2400801	C6orf130 Hs.22	-0.680102709	-0.95133526	-0.88108158	1.29820895	0.93906667	1.3112333
IMAGE:809600	DHX8 Hs.46310	1.030122125	0.83750431	1.08198077	-1.52836106	-1.62837416	-0.99718039
IMAGE:739511	PKMYT1 Hs.7778	0.601550884	1.26267774	0.17667505	-1.37665972	-0.83475504	-0.80577199
IMAGE:1640966	LOC645626 Hs.5	-0.706590372	-0.5818812	-0.69998326	2.22428162	0.44407885	0.13932597
IMAGE:124203	Hs.609453 Tran	-0.440139841	-1.39422598	-0.17752359	0.73112217	1.11922501	1.12310604
IMAGE:180902	BOK Hs.293753	1.362316742	0.9971312	-0.17853064	-1.48171292	-1.36685617	-0.46743952
IMAGE:771258	CD8A Hs.85258	0.669691607	1.31651497	-0.048258	-0.87203578	-1.06584541	-0.98110617
IMAGE:825677	ASNA1 Hs.4659	0.607648761	1.23636997	0.43224756	-1.38003132	-1.16378326	-0.76252297
IMAGE:1325420	Hs.621437 Vari	-0.707423766	-1.08419789	-0.24043	1.33849848	0.99075972	0.65871178

IMAGE:950574	Hs.642799 Hist	1.372517305	0.68031627	-0.33707505	-1.44621906	-0.82431969	-0.38736779
IMAGE:810964	PPFIBP1 Hs.172	-0.773967038	-0.75354729	-1.11191383	1.303657	1.04900576	1.32850102
IMAGE:1913255	AI304840 229	-1.340089389	-0.42080668	-0.82859106	1.56773146	1.25229626	0.85705521
IMAGE:700699	TMED1 Hs.5151	1.310536818	0.67089007	0.61681684	-1.97573078	-1.04593499	-0.72149561
IMAGE:366933	LOC653750 Hs.6	-0.899284659	-1.24684535	0.63584484	-0.01058215	1.35630046	1.07867065
IMAGE:50743	TXNL2 Hs.42644	1.152400494	0.93567594	-0.13074647	-1.26751836	-1.01260901	-0.6885542
IMAGE:241633	KCNH2 Hs.4388	-1.178587673	-0.35594638	-0.64446695	1.77310634	0.81452911	0.51974931
IMAGE:1032172	AA778449 751	-1.583073487	-0.21914591	-0.12394559	1.63970126	0.82965948	0.38890033
IMAGE:284383	SYNPO2 Hs.480	-0.547658408	-1.32183973	-0.0320925	0.9789217	0.67923861	1.18620251
IMAGE:767850	C6orf206 Hs.534	0.813477008	1.25106601	0.38682773	-0.20340462	-1.38027972	-1.99666485
IMAGE:73418	LYN Hs.491767	0.700938346	1.36719575	-0.24722294	-0.65953249	-1.16173656	-0.97190342
IMAGE:239924	SPON2 Hs.3029	-1.005030497	-0.56372368	-0.11101605	2.31379625	-0.17870843	0.35681351
IMAGE:813279	APEH Hs.51796	0.685196636	0.84073561	0.72587606	-1.88059825	-0.80378995	-0.51185525
IMAGE:486984	NAV1 Hs.49736	1.030044228	1.06622115	0.36542253	-1.35362717	-1.30517594	-0.94237312
IMAGE:84078	UGT1A9 Hs.124	0.788900728	1.00379311	0.2256565	-1.84013786	-0.83919179	-0.29178173
IMAGE:1290947	AA876747::AI	-0.632861223	-1.14082251	-0.3486312	1.2117802	0.92545066	0.95908373
IMAGE:823679	C18orf1 Hs.149	-0.660064527	-0.95814356	-0.66648469	0.5860587	0.68106184	1.99329746
IMAGE:285323	ITGB4BP Hs.632	0.792595113	0.8012977	0.7969464	-1.6521776	-1.2061703	-0.52867432
IMAGE:1658773	Hs.587300 CDN	-0.775941559	-1.1646417	-0.24306901	1.03153932	1.19812509	0.98504674
IMAGE:275612	R93354::R93	-0.513362857	-0.31539048	-0.64921287	2.26558907	0.17151917	-0.38246216
IMAGE:241242	PB1 Hs.189920	-0.966739214	-1.08630485	-0.55877406	1.57628361	1.38702143	0.81472864
IMAGE:824358	Hs.643726 CDN	0.576411701	0.7255635	0.7946705	-1.63687671	-0.79416904	-0.51525518
IMAGE:151449	PTPN21 Hs.4370	1.27749931	-0.19000769	0.90942974	-0.99487018	-0.86017973	-0.9129747
IMAGE:126230	ZBTB7A Hs.591	0.748923404	0.63771691	1.02569591	-1.89299834	-0.78301497	-0.68606705
IMAGE:2113499	GIT2 Hs.434996	0.704503196	1.34524759	0.4040533	-1.1119264	-1.46729724	-1.00046918
IMAGE:144855	C9orf46 Hs.584	0.474796766	1.33348074	0.12942103	-0.78972408	-0.99637888	-1.08808959
IMAGE:1553305	AA934769 271	-0.998750107	-0.80066672	-0.2250694	0.73856502	1.29567455	0.94622243
IMAGE:77301	AI821303::T5	-0.962053047	-0.80667279	-0.42903121	1.52622664	0.96381767	0.69933345
IMAGE:2338271	NFIA Hs.191911	-0.266540774	-1.23250297	-0.42400585	0.06972655	1.34995727	1.35888911
IMAGE:811133	ERICH1 Hs.389	1.010368386	0.97794874	-0.03118655	-1.29000227	-0.81549648	-0.83799375
IMAGE:795792	CUTL1 Hs.1914	1.140230091	0.47255062	0.43273487	-1.64335012	-0.65561329	-0.66112624
IMAGE:1664338	Hs.634167 CDN	1.570868651	-0.10567601	0.84897962	-1.45860969	-1.0573991	-0.74300468
IMAGE:161823	C6orf68 Hs.289	-0.754077785	-0.51342663	-0.81874453	0.40900232	1.52031338	0.99537158
IMAGE:713158	BIC Hs.388313	1.054850937	1.05485094	0.20518911	-1.37509637	-1.24679173	-0.7991511
IMAGE:705265	ZMYM6 Hs.5339	-0.766848473	-0.74591457	-0.5930971	0.91218117	1.57159904	0.52673573
IMAGE:950781	CDC26 Hs.1950	0.776190611	0.6546442	1.04011997	-1.47393738	-1.21348078	-0.75898401

IMAGE:1237871	ARID1B Hs.2915	-1.023265194	-0.78584003	-0.54515532	1.3093295	1.07464235	1.01113014
IMAGE:300965	LOC401320 Hs.5	-0.618714129	-1.42482907	-0.33696522	2.44647922	0.30988332	0.73015878
IMAGE:1570671	Hs.126893 Tran	-0.701771194	-0.87450459	-0.21221947	2.21426347	0.40582905	0.00959549
IMAGE:2149968	NCF1 Hs.520943	0.94869513	0.2653612	1.31664417	-0.82405164	-1.89108299	-0.75175507
IMAGE:810391	HYAL1 Hs.75619	1.375973703	0.10998493	0.70108329	-1.58111194	-1.04830371	-0.47587642
IMAGE:711680	ZNFN1A1 Hs.435	-1.282507261	0.11064482	-0.85189662	0.51335819	1.28866272	1.02064354
IMAGE:757143	REEP6 Hs.76277	-1.289814416	-0.53640875	-0.06176318	0.58199505	1.21342075	1.02112293
IMAGE:767202	LTBP2 Hs.51277	-0.909843952	-0.94070293	-0.18860794	0.627504	1.22323655	1.1608397
IMAGE:148763	KIAA0738 Hs.40	-1.214257913	-0.5694053	-0.59115188	0.81017099	1.37602583	1.22823786
IMAGE:328868	CD44 Hs.502328	0.935243372	0.9314487	0.41916762	-1.40170703	-0.88942595	-1.03286465
IMAGE:198580	R94726::R948	-1.111007861	-0.67801251	-0.63689982	1.34766222	1.0808671	1.05112601
IMAGE:726779	CNN1 Hs.46592	-0.36237803	-1.60112913	0.53571652	1.10241148	0.54026085	0.63294277
IMAGE:1008791	ACPP Hs.433060	0.807104665	0.51400251	0.71770032	-1.19442279	-1.11036743	-0.57399595
IMAGE:826355	VAMP5 Hs.17268	0.558999026	0.94770565	0.84557489	-1.49066626	-1.09481429	-0.73154508
IMAGE:743114	HSPBP1 Hs.5306	1.181725565	0.52637445	0.67103389	-1.92215102	-1.08378379	-0.39500758
IMAGE:745901	LOC23117 Hs.61	-0.404841723	-0.63516164	-0.2069574	2.30482437	-0.2377235	-0.24839908
IMAGE:700299	WASPIP Hs.5916	0.593777234	1.2746745	0.196059	-1.13465831	-0.96657876	-0.94651427
IMAGE:195051	Hs.613074 Tran	0.596091966	0.87294413	1.0097791	-1.61213359	-0.98751148	-0.86613294
IMAGE:77295	DEDD2 Hs.5154	1.177905387	0.31588693	0.9000312	-1.80543909	-0.71777462	-0.84251376
IMAGE:815239	ARHGEF1 Hs.63	1.352634411	0.96543917	0.0678502	-1.37445207	-1.18085445	-1.00661659
IMAGE:377205	Hs.598068 **C	-0.779402327	-1.05566868	-0.11392737	0.64248452	1.16802052	1.08451069
IMAGE:795382	RAPGEF3 Hs.85	-0.80454201	-1.50189716	0.30420743	0.74096259	0.76015459	1.57828227
*mitoch. cont. IMAG	148142	-1.104224327	-0.67425577	-0.37820047	0.43686059	1.31926636	1.38434379
IMAGE:236034	UCP2 Hs.80658	0.257902726	1.37547443	0.35757007	-1.16884415	-0.8813422	-0.84684196
IMAGE:1566550	KIAA1370 Hs.15	-1.186194657	-1.16497635	0.1993606	0.82079168	1.3495253	1.10723879
IMAGE:1460130	APOBEC3F Hs.4	0.315679013	0.92542781	1.03675265	-1.39395241	-0.98282877	-0.78081987
IMAGE:2471503	SH3BP2 Hs.1676	0.445955081	1.00976809	0.8943567	-1.894673	-0.7452891	-0.66156854
IMAGE:123436	R00486::R005	-0.550220948	-1.41267161	-0.27861709	0.63216187	1.13427709	1.52617125
IMAGE:1493596	GPR98 Hs.5917	-0.836987663	-0.86730667	-0.6581055	1.36739393	1.06609876	0.94558069
IMAGE:627401	TNFAIP8 Hs.271	0.842523786	0.89671036	0.48303459	-0.94052407	-0.99625883	-1.27586154
IMAGE:32076	F2 Hs.410092 C	1.061986854	0.60889076	0.70150507	-1.81995106	-0.81741689	-0.74582981
IMAGE:196837	EP300 Hs.51751	-0.736764705	-0.94819546	-0.543182	1.56511903	0.8340354	0.80726332
IMAGE:214231	H77640 28882	-0.99303335	-0.58687732	-0.62797892	1.63477305	0.90635361	0.61371298
IMAGE:338736	SERPINB1 Hs.38	0.611516391	1.24667919	-0.06273335	-1.50047178	-0.67705937	-0.53134555
IMAGE:1606321	TMC4 Hs.355120	-1.618069928	-0.00530031	-0.28088036	0.69793771	1.14418949	0.9440286
IMAGE:1602489	Hs.436703 Tran	-0.569531911	-0.85386389	-0.93496843	1.9288606	0.69900824	0.6759354

IMAGE:810037	ZBTB22 Hs.2067	0.899578929	0.81128418	0.70753784	-2.00504258	-0.40745936	-1.03821502
IMAGE:2448698	HLA-DRB1 Hs.53	0.531243856	0.67494643	0.9554142	-1.47030997	-0.79391684	-0.73932638
IMAGE:166335	MDS032 Hs.161	0.587664918	0.80894525	0.81686698	-2.22111662	-0.77143996	-0.12344241
IMAGE:1882185	KCTD1 Hs.5266	-0.964658112	-0.68547323	-0.96693717	1.3684349	1.62008114	0.69535243
*mitoch. cont. IMAG	142205	-0.578715473	-1.04889129	-0.74150753	1.94986983	0.59242676	0.82599797
IMAGE:2310169	ALDH3A1 Hs.53	1.16741419	0.22121352	0.58704534	-1.3083965	-1.02745517	-0.48089658
IMAGE:212456	LOC90355 Hs.48	-0.895398095	-0.4007359	-1.00414987	1.99415236	0.59299662	0.61223935
IMAGE:433225	DDX23 Hs.1300	0.978327426	0.86088568	0.35467127	-1.71615064	-0.91369956	-0.57230856
IMAGE:1507713	CHAD Hs.97220	-0.665632396	-1.36960421	-0.17655724	1.43694308	1.04404653	0.79256186
IMAGE:415698	GALC Hs.51343	1.064272463	0.97232167	0.20652252	-1.55220795	-1.12676398	-0.63407242
IMAGE:345621	CXX1 Hs.52278	0.677432983	0.27576322	1.37477632	-1.76954479	-0.86997188	-0.50874804
IMAGE:753291	C1orf21 Hs.497	1.027294494	0.58101643	0.52624594	-1.64986678	-0.93593457	-0.48447246
IMAGE:1584360	Hs.530159 CDN	-0.866095796	-1.05210809	-0.76964497	2.05109866	0.98817125	0.80009214
IMAGE:430677	MS4A7 Hs.5307	0.629536199	1.13345277	0.22789235	-1.03326436	-0.69824052	-1.19784401
IMAGE:854162	Hs.335223 **T	-1.026362135	-0.77491613	-0.44997052	1.53316479	1.05348318	0.67773258
IMAGE:855406	Hs.594613 Tra	1.676798085	0.33986675	1.05035026	-2.25191356	-0.90334918	-1.18267233
IMAGE:428413	SLC6A16 Hs.130	-0.811084127	-1.3388688	-0.20527911	1.70962039	0.74922712	1.02268077
IMAGE:383175	SART1 Hs.5028	0.881816293	0.909276	0.80822429	-1.57500336	-1.26196274	-0.85995269
IMAGE:1608958	AA991389 874	-0.569730928	1.03482823	1.55795847	-0.40771808	-1.46625088	-0.77112508
IMAGE:2412354	IGHG1 Hs.5106	-1.199112029	-0.56605961	0.09024049	0.61089928	1.27077596	0.65328161
IMAGE:1219044	TASP1 Hs.3699	-0.892850617	-1.21746306	-0.03833029	1.37570785	0.73306294	1.10461259
IMAGE:212258	TRA2A Hs.4456	-1.135561896	-0.66026682	-0.60323141	1.6432508	0.96833178	0.83619975
IMAGE:212489	WWP2 Hs.40845	0.811124709	0.33929848	1.2603539	-1.35977309	-1.35977309	-0.58153097
IMAGE:66317	HIST1H1C Hs.76	-1.438235748	0.08986603	-0.29101944	0.28875799	1.37106283	0.72650805
IMAGE:502558	ZFAND2B Hs.53	0.946132665	0.51672736	1.01227095	-1.7085299	-1.04796963	-0.7031292
IMAGE:460519	Hs.107418 CDN	-0.448542261	-0.52558703	-0.94651457	2.3191972	-0.01328628	0.33842623
IMAGE:814054	KIAA0040 Hs.51	1.175480141	0.79187357	0.82612416	-1.32036012	-1.08403107	-1.57929456
IMAGE:869466	BPI Hs.529019 I	1.043063428	0.98596591	-0.06507118	-0.77600332	-1.0079289	-1.17827282
IMAGE:1358393	MAP2K3 Hs.514	1.2904369	0.37418726	0.5142509	-2.19860672	-0.54410498	-0.39703816
IMAGE:2568869	EXT1 Hs.492618	1.268264528	0.34626723	0.731475	-1.67657384	-1.087923	-0.57164454
IMAGE:1861898	AI054063::AI	-0.923454374	-0.59995562	-0.78019064	1.65725052	0.76522196	0.83788081
IMAGE:239568	FAM63A Hs.334	1.094621312	-0.45983986	1.013807	-0.29081428	-1.01041499	-0.91820166
IMAGE:450193	IL24 Hs.58831 I	0.341366868	1.45438064	-0.29046519	-0.23017695	-1.25465554	-0.84570729
IMAGE:121558	KIAA0232 Hs.79	-0.855937961	-0.9317069	-0.65983012	1.35317225	1.16894974	0.98413296
IMAGE:279592	GNPTAB Hs.468	-1.173263688	-0.98368883	0.12228776	0.7553305	1.39081743	0.93642114
IMAGE:1696463	LOC284373 Hs.1	-0.604093079	-1.02642185	-0.79393195	1.03567156	1.16738796	1.23512782

IMAGE:378591	AA777219 10	1.372877307	0.14402188	1.03756743	-1.45422712	-1.17787282	-0.94020813
IMAGE:503581	RERG Hs.19948	-0.922009858	-1.24219015	0.0622321	1.20311677	1.03505444	0.93033868
IMAGE:273563	IMMP2L Hs.549	-1.12431915	-0.5985668	-0.37910642	1.63574328	0.98656736	0.43590132
IMAGE:1913269	C14orf72 Hs.15	-1.383833701	-0.86677355	-0.02331918	1.01914949	1.28219999	1.10371037
IMAGE:294127	HERPUD2 Hs.64	-0.655596551	-1.18561359	-0.76748904	1.63172141	0.95506633	1.13438876
IMAGE:809784	KLK6 Hs.79361	0.647450569	0.93820674	0.53240407	-1.63762291	-0.52609284	-0.8802753
IMAGE:214658	LARP5 Hs.6318	-0.849366366	-1.28813867	-0.25648867	1.02736621	1.12102894	1.37847324
IMAGE:743143	Hs.201633 Tra	1.720922342	-0.16808776	0.38552416	-0.84963372	-0.90552723	-1.05599612
IMAGE:1837653	TG Hs.584811 T	-0.883664126	-0.91117987	-0.09605184	0.81351931	0.72973077	1.26908071
IMAGE:505864	RGL1 Hs.49714	0.740609075	1.20656324	0.17652214	-1.03442921	-1.01854441	-1.08843753
IMAGE:1420801	UPF1 Hs.51526	0.859558243	0.80715417	0.62993312	-1.90046908	-1.00721781	-0.37979812
IMAGE:362279	OR2A7 Hs.5616	0.982817314	0.89086213	0.7317301	-1.06882949	-1.23814817	-1.41820413
IMAGE:117364	RAD23B Hs.521	-1.19792051	-0.82309913	-0.38402266	1.95660299	0.74847336	0.80648143
IMAGE:2124107	UPK1A Hs.1593	1.029519786	-0.03225196	0.87077232	-0.68878823	-0.94645669	-0.94912222
IMAGE:240674	LOC400721 Hs.	-1.171149176	-0.31638989	-0.85061444	1.34304514	1.33369621	0.6178353
IMAGE:49631	Hs.532778 Tra	-1.133649109	-0.36848701	-0.30158339	2.02331744	0.46838501	0.13848095
IMAGE:700792	CDKN3 Hs.8411	0.709136693	1.14369647	0.0105871	-1.38323189	-0.64715691	-0.76209482
IMAGE:730971	ACE2 Hs.17809	-0.814123442	-0.95866389	-0.53165711	1.22395813	1.01523192	1.08456234
IMAGE:760298	LGMN Hs.18069	0.952706333	0.89534934	0.42349243	-1.38497372	-1.01215324	-0.90432208
IMAGE:746157	FLJ32569 Hs.17	-1.039347853	-0.55678527	-0.16043545	0.78013243	1.09341663	0.72119494
IMAGE:770462	CPZ Hs.78068 C	0.542993498	0.50954279	1.10585749	-1.01164695	-1.11954408	-0.82993525
IMAGE:342039	PRSS23 Hs.253	-1.009521931	-1.21540193	-0.49335136	1.35184814	1.75993171	0.84229514
IMAGE:283461	RMND5A Hs.752	1.392939903	0.51175261	0.73368126	-1.30553299	-0.94938646	-1.51922091
IMAGE:137797	KIAA1856 Hs.55	-0.748058508	-0.94855062	-0.66477717	1.06723052	1.05694888	1.25170576
IMAGE:431280	RP1-93H18.5 Hs	0.215345374	1.4347601	-0.02531267	-0.70344835	-0.89089557	-0.84917345
IMAGE:2310272	DHCR7 Hs.5031	-0.928407518	-0.73076772	-0.24376789	0.47550668	1.25011559	1.06785044
IMAGE:489525	QKI Hs.510324	0.728457138	0.94163052	0.57461137	-1.34355434	-0.74336975	-1.13647162
IMAGE:854338	CHI3L2 Hs.5148	-0.04770967	1.23989694	0.43232876	-1.35334311	-0.27935022	-0.69599852
IMAGE:810321	CARS Hs.27487	1.107082322	0.3549252	0.90390222	-1.7811522	-0.73350477	-0.80823207
*mitoch. cont. IMAG	145850	-0.896961842	-0.76286799	-0.57886345	0.65778392	0.96043898	1.59510117
IMAGE:587782	FOXP1 Hs.4314	-1.154954797	-0.71505519	-0.19886531	0.93374799	1.13421506	0.98563358
IMAGE:488345	DHRS10 Hs.187	0.6766182	0.93400792	1.58986709	-2.43413583	-1.35180666	-0.61733055
IMAGE:2569053	FKBP2 Hs.2277	0.776631942	1.09171502	0.62955398	-1.42345327	-1.39964568	-0.76636397
IMAGE:854429	AA669059 11	-0.788363235	-0.72867568	-0.3529442	1.73723724	0.55312287	0.42637852
IMAGE:758371	SMC5 Hs.5341	-0.380895265	-1.43037761	-0.63714924	0.40879191	1.47115135	1.6334026
IMAGE:1635534	RALBP1 Hs.528	1.135299784	0.52961603	0.68809569	-1.587317	-1.1023146	-0.66786174

IMAGE:1894979	MYOG Hs.2830 I	-0.455423881	-0.90535739	-1.17636386	0.7182617	1.30858144	1.4847836
IMAGE:1586016	DAPK3 Hs.6318	1.316088247	0.58113899	-0.08470747	-0.53841419	-1.12695922	-1.0745831
IMAGE:1590269	SLC2A4 Hs.380	-0.836235102	-0.98881338	-0.48021912	1.17981954	0.89357225	1.26445484
IMAGE:461799	SELT Hs.369052	-0.963365091	-0.87482134	-0.29592843	1.03886855	0.97543764	1.112884
IMAGE:727263	FAM36A Hs.411	-0.83498634	-1.15621794	-0.24581728	0.44578408	1.52389011	1.32440383
IMAGE:1740334	Hs.551905 CDN	-0.934661453	-1.10111802	-0.38686802	1.04087535	1.34352366	1.15285523
IMAGE:809998	AMY1A Hs.484	-0.505690918	-1.2268037	-0.07758966	0.86737251	0.90162061	0.92673589
IMAGE:123608	FHIT Hs.196981	-1.112325032	-0.58892422	-0.59923363	0.86055803	1.43191837	1.00843953
IMAGE:145643	ATP8B4 Hs.511	-0.753852231	-0.8725628	-0.68402249	1.19914026	1.19317564	0.90233475
IMAGE:838408	TAF4B Hs.36951	-0.543346102	-0.90930177	-0.85418796	2.18561397	0.57353516	0.48755763
IMAGE:282162	LOC374676 Hs.	-0.620228676	-1.20533345	-0.53199859	1.21008122	1.41643514	0.77682507
IMAGE:135010	Hs.594441 Hon	-0.504163635	-0.84154576	-0.45911109	1.87619782	0.19048176	0.52577338
IMAGE:435743	ZNF141 Hs.601	-1.137168119	-0.62181355	-0.57854921	2.27267042	0.56334291	0.52564568
IMAGE:814109	POLS Hs.481542	-0.971997942	-0.72467559	-0.60526137	0.98372228	1.41489695	0.90296779
IMAGE:1659444	Hs.604033 Trar	-0.690419193	-1.02637444	-0.43756356	0.72631604	1.43605787	0.95977099
IMAGE:280985	CPEB4 Hs.1271	-0.638853845	-0.73552091	-0.86311846	0.72216182	1.36566488	1.05263351
IMAGE:364415	LOC284408 Hs.	-1.013464717	-0.91293668	-0.31811104	0.68737789	1.38189731	1.2179657
IMAGE:878148	TBC1D9B Hs.15	0.800741826	0.50645687	0.99513189	-1.78370106	-1.08173695	-0.3392749
IMAGE:416803	HAL Hs.190783	0.937077904	0.37125962	0.72026903	-1.11176597	-0.96135114	-0.78972547
IMAGE:742132	ISG15 Hs.4584	0.933545976	0.88677984	0.0632036	-1.39737663	-0.78683244	-0.62528416
IMAGE:2577145	SCAMP3 Hs.200	0.774977463	0.82752036	0.57149317	-2.03296246	-0.87224221	-0.21290852
IMAGE:2384055	FRY Hs.591225	0.699932971	-0.16992335	0.45271065	1.29621302	-1.26463929	-1.39247647
IMAGE:1556401	AA936454 13	-1.014573237	-0.73784253	-0.75629124	0.83106685	1.23693855	1.5059823
IMAGE:77193	MAFB Hs.64267	1.320695735	-0.01722228	0.69135469	-0.88540767	-0.67741338	-1.2565825
IMAGE:201213	FRMD4A Hs.330	-1.104684623	-0.72050591	-0.42751909	1.36073172	1.22525817	0.68619478
IMAGE:1476309	VENTX Hs.1252	0.987876743	0.70179546	0.65749901	-0.4756751	-1.36698748	-1.51371949
IMAGE:745773	GAPDHS Hs.248	-1.410838599	-0.7485166	-0.07031783	1.46407134	0.81182042	1.05103833
IMAGE:612274	TUBA1 Hs.7531	1.224826404	0.24949378	0.58151899	-1.3833298	-1.15666799	-0.39838122
IMAGE:1948644	FLJ21963 Hs.25	-1.238856602	-0.00232384	-1.1479273	0.80782048	1.25227143	1.23658788
IMAGE:276091	ITPKB Hs.52808	1.250457582	0.21975685	0.68770368	-1.6365917	-1.02991342	-0.39844614
IMAGE:298610	ZNF324 Hs.515	0.46239211	-0.3682726	1.05996461	-2.25466494	0.30740283	0.48112708
IMAGE:301315	Hs.643777 CDN	0.876578957	0.62578809	0.98943485	-1.79512755	-0.94870836	-0.74650822
IMAGE:1913608	AI310424 315	1.496578828	-0.74014989	0.46428434	-0.57748283	-0.88554859	-0.25196742
IMAGE:1851134	TNF Hs.241570	1.063519517	0.8086956	0.64544085	-1.45567914	-1.07108863	-1.08835596
IMAGE:813393	SSBP2 Hs.1027	1.379032731	0.4083616	0.44240976	-0.75353174	-1.13881351	-1.34175845
IMAGE:915113	AA579267::A	-1.295477203	-0.58548101	-0.34881561	1.15978838	1.0432486	1.05441987

IMAGE:811808	FAM65A Hs.152[illegible]	0.864619682	0.721324	0.85814826	-1.59844818	-1.05068889	-0.80246379
IMAGE:1956910	WNK2 Hs.52229	-1.475215025	-0.12595428	-0.53777611	0.60449236	1.331476	1.13800574
IMAGE:1892310	Hs.635262 Tran	-0.911600804	-1.20219763	0.08805227	1.25396824	1.1351349	0.67152918
IMAGE:1636105	Hs.586328 CDN	-0.812183617	-0.8709904	-0.26722712	0.77700306	1.10341143	0.97838043
IMAGE:417707	CTDSPL Hs.475	-0.487359374	-1.28742753	-0.53951851	1.01398399	1.2682598	1.05433471
IMAGE:2506801	DNM2 Hs.21146	0.797212712	0.27540893	1.06463715	-2.24310962	-0.18035405	-0.51626524
IMAGE:2056566	ITGAM Hs.1726	0.787471214	0.81150385	0.70782021	-1.07132412	-0.99729503	-1.21461869
IMAGE:66784	Hs.593576 Tran	1.323484884	0.57790914	0.18938806	-1.78907717	-0.6064152	-0.69333373
IMAGE:138693	LOC349114 Hs.4	-1.036999267	-0.7041005	-0.50436124	0.47853289	1.54675867	1.21680965
IMAGE:293178	PBX3 Hs.428027	-1.075463564	-0.74454334	-0.22326519	1.39540745	0.98459363	0.62909076
IMAGE:140280	R67904::R668	-1.250752486	-0.88857803	-0.65660678	1.62756169	1.40980804	0.99238451
IMAGE:1374906	AA947534::AI	-1.084514034	-0.62715055	-0.39778618	1.55437086	0.87856512	0.63179363
IMAGE:1698064	TERF1 Hs.44270	-1.067401639	-0.71945682	-0.5647023	1.6070954	0.87656301	0.90250715
IMAGE:195988	OCIAD1 Hs.518	-0.975008349	-0.70488258	-0.50055667	1.53110083	0.95708356	0.65734784
IMAGE:712916	PSMC3 Hs.2507	0.917055543	0.61864656	0.55628244	-1.75974008	-0.6683679	-0.57079823
IMAGE:753620	IGFBP6 Hs.2743	0.864240942	-0.43867442	1.30212796	-0.7573514	-0.8614503	-0.64720804
IMAGE:46931	OTUB2 Hs.2788	-0.2394048	-1.4156524	-0.07872098	0.35178214	1.2751015	0.95410337
IMAGE:1622314	SERGEF Hs.324	0.709726018	1.0767345	0.70096164	-1.72704594	-1.10258376	-0.72626312
IMAGE:344555	UBE2R2 Hs.111	0.905323271	0.69921846	0.51135898	-1.70595718	-0.7379403	-0.60211384
IMAGE:198526	SPAG9 Hs.4634	-1.005880125	-0.90058361	-0.23612629	1.3983298	0.89847392	0.85804974
IMAGE:428560	P117 Hs.356626	0.422315877	0.7821733	0.79534354	-1.99074311	-0.74150718	-0.0686629
IMAGE:136856	TMEM107 Hs.51	0.972706148	0.84449806	0.90117953	-0.98651586	-1.87722467	-0.9885402
IMAGE:1472538	PLA2G6 Hs.1704	-0.432164616	-1.19972886	-1.0020301	0.40924036	1.49729468	1.79384282
IMAGE:343930	GFRA3 Hs.5804	-1.075431323	-0.99662123	0.15826417	0.77062983	0.72020631	1.41941247
IMAGE:462625	PHKA2 Hs.5494	-1.112914186	-0.57631667	0.02674577	-0.09718996	1.53546896	1.06213507
IMAGE:49117	PHF16 Hs.37197	-1.212847565	-0.55482683	-0.53529184	0.49723524	1.78191792	1.04147323
IMAGE:810979	MRPS2 Hs.3820	0.654363976	1.00962082	0.71276236	-1.58594408	-1.32315135	-0.47783472
IMAGE:897099	GGT1 Hs.35654	-1.01641325	-0.78184667	-0.50557936	1.8721439	1.07483471	0.38238547
IMAGE:140150	Hs.28664 Trans	-0.764190193	-0.77412075	-0.55664166	0.63763131	1.35635498	1.0092822
*mitoch. cont. IMA	153263	-0.715470614	-0.40848309	-0.97424667	0.70233786	1.06083335	1.14056769
IMAGE:323522	NRBP1 Hs.5158	0.887124782	0.56696613	0.81560882	-1.74212617	-0.96875592	-0.48976531
IMAGE:841022	BTBD9 Hs.1162	-0.857388292	-1.59256054	-0.02958435	1.37588119	1.14705883	1.18896365
IMAGE:1880054	ROS1 Hs.1041 V	-0.963058254	-0.5969325	-0.47220834	0.88295986	1.08043978	0.96684692
IMAGE:321271	PRR5 Hs.10233	-1.318637147	-0.51832878	-0.20058258	0.50246059	1.47352783	1.03018869
IMAGE:562067	AGPAT3 Hs.248	-1.07039095	-0.45606633	-0.30505916	0.42179766	1.23150006	1.01771215
IMAGE:159455	PLD3 Hs.257008	0.743356752	0.72353534	1.05945604	-1.58904917	-0.68143734	-1.2541717

IMAGE:1635350	PRR13 Hs.42635	0.957934042	0.84251804	0.5656187	-1.79050243	-0.82085939	-0.79633968
IMAGE:279665	PDHX Hs.50231	-0.838723941	-0.73291226	-0.58664316	0.5808643	1.48181968	1.02807427
IMAGE:1655779	AI040138 136	-0.398962285	-0.61168638	-0.45027512	2.29141216	-0.23864525	0.02605
IMAGE:345234	ARHGAP30 Hs.3	0.815095379	0.97389345	0.23427124	-1.27848226	-0.81016252	-0.88767751
IMAGE:2253645	TAS2R49 Hs.408	-0.431109936	-1.05528308	-0.56817326	1.32316691	0.64820008	0.96843912
IMAGE:743341	C21orf91 Hs.29	1.212393606	0.36693581	0.8085104	-1.25593138	-1.02524434	-1.0984564
IMAGE:2028349	DNAH5 Hs.2123	-0.995578492	-0.94568651	-0.41229589	1.19672048	1.25834032	0.97220657
IMAGE:842836	SERPINB1 Hs.38	0.617828571	1.14828949	0.18556447	-1.55698691	-0.79769486	-0.52645091
IMAGE:594540	PTCH Hs.494538	0.873654479	0.66953863	0.82482677	-0.78468674	-1.31899	-1.24214638
IMAGE:1565868	Hs.133924 Tran	-0.872287367	-1.65188518	0.19723655	2.03824625	0.79475262	0.70671426
IMAGE:1533710	SLC25A26 Hs.37	0.908767555	0.53041782	0.9325244	-1.55864467	-1.15609815	-0.60969073
IMAGE:2336916	DIRAS3 Hs.1946	-0.892969319	-0.76024968	-0.25956788	0.52096973	1.18724668	1.09607194
IMAGE:531036	KRT18 Hs.40601	-1.259587818	-0.30859821	-0.49209902	1.11671422	0.55917981	1.29150878
IMAGE:768523	ZMYM6 Hs.5339	0.514003724	1.42601223	0.15029583	-1.06505821	-1.32797958	-0.70485593
IMAGE:2298075	AI635997 139	-1.302191658	-0.23491755	-0.68638394	1.66185522	0.89681802	0.6049705
IMAGE:1555396	AA975183::AI	-1.244874078	0.06879131	-0.53458308	1.96739004	0.28904783	0.17591513
IMAGE:593185	ABCC4 Hs.5084	0.904920961	0.93861153	0.41303864	-1.05980077	-1.09349134	-1.12830493
IMAGE:202919	VPS13D Hs.380	1.359693478	0.8850177	-0.08806766	-1.06708646	-1.19091492	-0.9989808
IMAGE:701261	CCDC51 Hs.187	1.11940536	1.00697374	0.09360071	-1.73341307	-0.89820677	-0.6749497
IMAGE:797048	BMP4 Hs.68879	1.104276676	0.7851188	0.48261264	-1.37197214	-1.04587605	-1.01951083
IMAGE:1031747	ALKBH1 Hs.945	-1.0966345	-0.33859449	-0.79532552	0.26521536	1.74553878	1.13624624
IMAGE:1926956	EHD3 Hs.36880	-0.063862585	-0.4844947	-1.01792201	2.49216776	-0.50065026	0.10342093
IMAGE:109153	COPA Hs.16212	0.192724646	1.6185259	0.11323307	-0.2723957	-0.55392836	-2.03209311
IMAGE:259819	KCTD10 Hs.524	0.861777702	0.79067694	0.86104846	-1.69953611	-1.22603238	-0.62942406
IMAGE:950613	RIN3 Hs.326822	-0.761823312	-0.7431599	-0.55652578	1.93000059	0.51780242	0.50532904
IMAGE:375827	PTP4A3 Hs.4366	0.48947072	1.17778892	0.56126305	-1.25667413	-0.95692266	-0.98887148
IMAGE:80742	AI821346::T6	-0.508570376	-0.68106439	-0.05766497	2.15797526	-0.16161531	-0.13982659
IMAGE:435824	C8orf42 Hs.289	-1.144034195	-0.97489991	-0.50262493	1.5240592	1.28743383	0.99518929
IMAGE:1690065	Hs.597349 CDN	-0.946132912	-0.95747705	-0.26334956	1.6592808	1.18337218	0.34194891
IMAGE:754108	KIAA1109 Hs.40	-0.823967148	-1.2559795	-0.39104529	1.2483658	1.10185785	1.25850294
IMAGE:1856361	Hs.469747 Tran	-0.81677424	-0.97424032	-0.32858806	1.79174852	0.73009236	0.57541603
IMAGE:1631634	ARPC5L Hs.1324	1.248144559	0.78848096	0.31470676	-1.57406522	-1.00928475	-0.86497176
IMAGE:324342	ASAHL Hs.6326	0.877119862	0.71959462	0.67898811	-1.29795368	-0.81487627	-1.13097692
IMAGE:1856268	ZNF605 Hs.2969	-1.057205189	-0.47157713	-0.82311747	1.03677619	0.98384076	1.30145337
IMAGE:53245	PPARA Hs.10311	-0.813269666	-0.55691292	-0.87767148	0.80302833	1.27713199	1.07220291
IMAGE:213577	CTBS Hs.51355	1.066256442	0.76498803	0.3179057	-1.45253579	-1.05147221	-0.64024083

IMAGE:1386594	AA831913::AI	-0.843860053	-0.57698087	-0.75012974	1.4030574	1.02473416	0.64113199
IMAGE:1864283	AI249759 112	0.896423263	0.97938166	0.14835498	-1.58727886	-0.81089652	-0.60097572
IMAGE:85394	PPAP2B Hs.4051	0.674549161	0.75180032	0.85995195	-1.10174476	-1.13071395	-0.98200546
IMAGE:2449679	PLXNB3 Hs.6328	-1.207558821	-0.88594036	-0.49243071	0.99741954	1.38446529	1.37390233
IMAGE:77133	SHC1 Hs.43379	1.019504149	1.01825721	0.17731828	-1.63881386	-1.07835417	-0.56112186
IMAGE:1686314	PRICKLE1 Hs.52	-0.758486762	-1.12065836	-0.17149544	1.04263359	0.67362857	1.31682482
IMAGE:242807	RP4-691N24.1 H	-1.219776198	-0.55696314	-0.86668887	1.23949032	1.10940551	1.39957427
IMAGE:813637	DNAH9 Hs.5672	0.735665848	0.63936457	1.12032066	-1.00518608	-0.62548391	-1.83227647
IMAGE:927208	C10orf99 Hs.298	1.307115234	0.09411159	0.22568712	-0.7945348	-0.9333719	-0.65604243
IMAGE:199239	ARMCX4 Hs.388	-1.009709361	-1.16767859	-0.04471483	1.04216285	1.08659169	1.19322092
IMAGE:144042	CNOT2 Hs.1333	0.602359251	0.67104242	1.24340212	-1.7565804	-0.91439397	-0.79338078
IMAGE:229997	C1orf112 Hs.44	-1.182217118	-0.40822673	-0.48119379	2.06711106	0.6867288	0.23331816
IMAGE:784693	GTF2B Hs.48185	0.944808028	1.02100487	0.30475453	-1.61897767	-0.94749298	-0.76319186
IMAGE:295986	EBP Hs.632801	-1.027656863	-0.29471946	-0.63792031	0.17398237	1.39990742	1.20707508
IMAGE:490961	ISG15 Hs.45848	0.919096753	0.7266055	0.47251704	-1.24876727	-0.97744626	-0.83298615
IMAGE:1575159	CD24 Hs.37510	-0.302311138	-1.23402359	-0.88300168	1.27678601	0.96776136	1.16370681
IMAGE:814489	LOC440248 Hs.5	-0.557854804	-0.78001906	-0.95351876	0.76508043	1.38925619	1.04437263
IMAGE:83029	HGD Hs.368254	-1.022150272	-0.23800727	-0.84517051	1.84566659	0.89286713	0.20816572
IMAGE:1836699	FAM79B Hs.338	0.891642313	0.06931209	1.0184351	-0.75676521	-1.12134511	-0.83636516
IMAGE:1636369	Hs.592876 Tran	-0.625579226	-1.05325808	-0.28343614	1.68658608	0.89134059	0.29462447
IMAGE:51950	AQP1 Hs.76152	0.926433765	0.01404651	1.24807331	-1.22712273	-1.46904359	-0.27464573
IMAGE:913912	LOC653269 Hs.5	-0.845827654	-0.65376328	-0.50995309	1.78481939	0.65148084	0.45052753
IMAGE:1567985	AA975922::AI	-0.618782953	-1.24571979	-0.44050265	1.97234854	0.25488386	1.12015003
IMAGE:281045	PARP9 Hs.51820	1.147565643	0.98347531	0.48609328	-0.94794868	-1.19509294	-1.6611364
IMAGE:810989	TMEM147 Hs.92	1.127305354	0.28783097	0.7506763	-1.5323537	-0.93678066	-0.5919155
IMAGE:324715	FOLR3 Hs.352 F	1.023458897	0.16726884	0.73143059	0.02747183	-1.61918416	-1.10866751
IMAGE:1009355	KCNH2 Hs.4388	-1.048961849	-0.77645223	-0.347515	1.21700538	1.08681758	0.86869191
IMAGE:417223	LOC399715 Hs.5	-1.139729473	-1.15728812	0.30242049	0.30665338	1.23546733	1.52538006
IMAGE:758266	THBS4 Hs.2114	1.428114334	0.29199716	0.60111331	-0.63023298	-0.99127698	-1.71004891
IMAGE:286378	ZNF135 Hs.8586	-1.558264103	-0.54413352	0.56788272	0.97463727	1.11220629	0.35689947
IMAGE:81409	GABARAPL1 Hs.	-1.114774098	-0.80697757	0.00065374	0.47489458	1.2705932	1.13632255
IMAGE:1470149	Hs.554362 Tran	-0.955181847	-0.70656909	-0.69133153	1.17266271	1.02469801	1.15943009
IMAGE:795334	CD209 Hs.2786	0.603444145	0.75818632	0.64791374	-0.94500418	-0.86503975	-1.04229394
IMAGE:44477	VCAM1 Hs.1092	0.772531767	0.76677933	0.36633551	-0.96050575	-1.35021014	-0.45617015
IMAGE:2029309	MSRA Hs.49098	-0.649497388	-0.949574	-0.43779609	1.99352345	1.03487605	-0.08254732
IMAGE:187616	MYCL1 Hs.4379	0.848994335	-0.09434349	1.25561518	-1.08372184	-0.92321361	-0.69455979

IMAGE:1602516	DAXX Hs.33691	0.660533092	0.71013865	0.85013654	-2.22127392	-0.62562865	-0.27177571
IMAGE:1658202	Hs.602888 Tran	-0.52561367	-0.87491285	-0.99912664	0.9297301	1.20248983	1.21747815
IMAGE:666128	CFD Hs.155597	1.029891726	-0.20239198	1.11122245	-2.23165516	-0.30929259	-0.08932995
IMAGE:290307	CECR2 Hs.2318	-0.480243614	-0.60788889	-0.82712295	2.25735281	0.30243907	0.10631056
IMAGE:1486719	AA927623 85	-1.030564946	-0.8879407	-0.31533074	1.90723049	0.83457728	0.53011412
IMAGE:257999	SERTAD4 Hs.60	-0.67487224	-1.25510569	-0.28044856	0.95563441	1.45285677	0.83703642
IMAGE:1583526	Hs.537960 Tran	-0.722844271	-1.3753381	-0.03444319	1.92241025	0.77239347	0.49552382
IMAGE:45231	GPR19 Hs.5048	0.512385897	1.117516	0.8581007	-1.1231707	-1.42332419	-0.97098384
IMAGE:1692237	AI090818 284	-1.36853586	-0.75062735	-0.11846798	1.56875626	1.07609948	0.68197405
IMAGE:279171	Hs.444781 Tran	-1.066991244	-1.08167919	0.10077377	0.8848582	1.3116479	0.90053234
IMAGE:294487	PDCD4 Hs.2325	-0.501569441	-0.5032243	-1.02020101	0.81842568	0.76133318	1.20268301
IMAGE:854263	Hs.634576 Tran	-1.172406023	-0.70343155	-0.37969916	1.38092377	1.11187591	0.79558063
IMAGE:1032007	AA610013 10	-0.509477564	-0.45513729	-0.36710604	2.55341209	-0.46247323	-0.18513404
IMAGE:38925	R51593 12816	0.778040109	0.54735217	1.0872561	-1.6500681	-0.72778715	-0.9693033
IMAGE:1461025	Hs.126020 Tran	-0.378125371	-1.35899292	-0.34800424	1.09182436	1.22119075	0.72766763
IMAGE:247081	HDAC7A Hs.200	1.163578954	0.77389677	0.65430465	-1.85209084	-1.17686568	-0.69513788
IMAGE:810801	AGRIN Hs.2733	-1.41067184	-0.64813753	0.13949286	0.31540663	1.27822685	1.3202145
IMAGE:392624	Hs.601172 Tran	-0.841382297	-0.85558078	-0.52759591	0.77316228	1.07292767	1.35884955
IMAGE:213622	DKFZP564J102	-0.645715396	-1.07849291	-0.36018385	1.81220222	0.67603753	0.54830252
IMAGE:2063468	CPT1B Hs.4397	-0.588017968	-0.92736073	-0.82993652	0.16264106	1.59937452	1.54847311
IMAGE:49303	PPP2R2B Hs.193	1.435485747	-0.08045327	0.45649488	-0.53380184	-1.16199295	-0.90737252
IMAGE:267864	N25650 1667	-0.761912718	-0.92681628	-0.55678877	1.61571514	0.81331853	0.8000458
IMAGE:2457025	PDPN Hs.46867	0.501995068	1.17470839	0.08516827	-1.31465024	-0.54933239	-0.7575329
IMAGE:796694	BIRC5 Hs.51452	0.794147382	1.1680437	-0.01538162	-1.73769588	-0.58926899	-0.59709473
IMAGE:746229	MAP4K4 Hs.431	1.016755983	1.11202771	-0.18113929	-1.52623165	-0.85639462	-0.58412517
IMAGE:1056198	C14orf58 Hs.50	1.443827901	0.47520899	0.68577832	-1.68207379	-0.91700522	-1.13669922
IMAGE:359191	HSPB8 Hs.4000	1.156877875	0.29913877	0.49661183	-0.95671815	-1.3869667	-0.46110491
IMAGE:361091	BRI3BP Hs.6327	-1.273513537	-0.4730983	-0.31984344	0.54632472	1.34054401	1.13285332
IMAGE:744846	GORASP2 Hs.43	1.124177465	0.76914163	0.20357031	-1.2291974	-1.29329031	-0.57195382
IMAGE:203130	ARPC4 Hs.3233	1.255837381	0.86656081	-0.1276605	-1.05454143	-1.33643136	-0.63304887
IMAGE:1457251	D4ST1 Hs.4424	0.307306047	1.09521668	1.18612945	-1.51307056	-0.97668524	-1.09669009
IMAGE:118581	DOCK8 Hs.1325	-0.592941117	-1.20970196	-0.30625366	1.66206414	0.80391586	0.62080169
IMAGE:726658	NME3 Hs.51406	0.431620414	1.26211891	0.38833711	-1.7927359	-0.66331205	-0.56998242
IMAGE:122665	T98915::T989	-0.989031531	-0.72821911	-0.49721383	1.43652397	1.00674237	0.75412691
IMAGE:135182	KIAA2026 Hs.53	-0.289176771	-1.55813813	-0.39899074	0.43468027	1.44130828	1.39372222
IMAGE:356992	HSPC023 Hs.23	0.863184345	0.67797372	0.7565753	-1.7945882	-0.84594839	-0.61691963

IMAGE:841620	DPYSL2 Hs.1733	1.026658305	0.67489278	0.59510206	-1.52514319	-1.04897278	-0.72208823
IMAGE:213535	H72259::H71	-1.451762327	-0.78822209	-0.21159262	2.10520365	0.71616023	0.80310352
IMAGE:1590169	AA946953::AI	-0.936655198	-0.65294171	-0.46289496	2.23571578	0.35648257	0.37081571
IMAGE:1593194	REPS1 Hs.3346	-1.088201214	-1.1843504	-0.09185533	1.00158406	1.21314446	1.30891805
IMAGE:705064	TACC3 Hs.1040	-0.170492995	1.50128628	-0.03844946	0.36064119	-0.9459592	-1.36281009
IMAGE:810290	MTCH2 Hs.2699	-1.047615559	-0.57975328	-0.53926735	0.56716169	1.52339016	1.0245856
IMAGE:486401	ZNF251 Hs.5345	-0.791435737	-0.87296118	-0.55945879	0.61172835	1.31210605	1.27208446
IMAGE:838628	SUB1 Hs.22964	-0.225583665	-1.16382244	-0.95295096	0.84207765	1.28697801	1.14624219
IMAGE:328768	HS2ST1 Hs.488	-0.691890211	-1.31687036	-0.47278798	0.99777116	1.47568542	1.13066924
IMAGE:206849	PHF21A Hs.5024	-1.059728459	-0.96313872	-0.13708647	1.18750532	0.90734258	1.11081096
IMAGE:1555209	Hs.567879 Hom	-0.831914836	-0.84958704	-0.42015249	1.67225652	0.81013412	0.56505279
IMAGE:1696319	C10orf59 Hs.14	-0.978061289	-0.64407305	-0.80871514	0.86692971	1.38892914	1.18823659
IMAGE:487878	SPARC Hs.1117	0.626373435	0.64162811	0.82947852	-1.22238406	-0.99029509	-0.72617131
IMAGE:564517	Hs.69494 Trans	-0.937291641	-0.78696709	-0.52257274	1.72367394	0.64114771	0.87478237
IMAGE:704320	RPL23AP7 Hs.24	-0.764817273	-0.9813302	-0.21943877	1.4680454	0.85800561	0.56746867
IMAGE:359287	LOC375449 Hs.4	-0.94648746	-0.66405731	-0.43979099	0.56754989	1.36510548	1.03290051
IMAGE:204686	FXYD1 Hs.44249	0.781619979	-1.05996534	1.71789263	-0.41594427	-0.93639229	-0.37751119
IMAGE:36367	ULBP2 Hs.64273	0.905050201	0.91358962	0.22925795	-0.97789253	-0.89639993	-1.14023971
IMAGE:1603855	LOC387904 Hs.1	-0.758826948	-1.09679412	-0.69348663	1.13838933	1.18814561	1.32375494
IMAGE:811025	HDAC10 Hs.265	-0.070685906	-1.04936679	-0.97852198	1.40153499	1.05020194	0.4514946
IMAGE:452668	EFTUD2 Hs.151	0.825963384	1.05891312	0.25642504	-1.7951966	-0.8610206	-0.49162887
IMAGE:33294	ZNF559 Hs.1729	-1.097502974	-0.57350182	-0.76482317	1.99258056	0.59422865	0.87572695
IMAGE:71101	PROCR Hs.3562	1.025827863	0.89164535	0.19687813	-1.1969427	-0.76644047	-1.15892432
IMAGE:1638894	Hs.131087 Tran	-0.92611485	-0.93718627	-0.35842772	0.70401355	1.3822073	1.15676549
IMAGE:277423	DCAMKL1 Hs.50	0.608429202	0.22545136	1.40997475	-0.81477836	-1.30974821	-0.88876271
IMAGE:950722	SCGB2A2 Hs.46	-0.984082248	-0.78364242	-0.26929343	1.5658776	0.72696047	0.69536572
IMAGE:1030854	Hs.584286 Tran	-0.800771936	-0.57303869	-0.36836343	2.05176489	0.46296967	0.00643567
IMAGE:767828	HPS1 Hs.40456	0.827883871	0.52462825	0.76569728	-1.78568054	-0.89894576	-0.30126348
IMAGE:34616	KCTD2 Hs.5144	0.975784628	0.76301164	0.78286043	-2.14562511	-0.7043325	-0.73681233
IMAGE:1415672	LOC441119 Hs.1	-1.025582673	-0.5791725	-0.58350657	1.44953784	1.0065588	0.68041933
IMAGE:39995	SEMA4C Hs.516	0.612840944	1.21006371	0.79187417	-1.78172839	-0.8655192	-1.07695209
IMAGE:133423	R27446::R27	-1.090562224	-1.00428281	-0.16994498	1.23458803	1.5613198	0.55879094
IMAGE:260200	MNDA Hs.15383	0.652213213	0.8836097	0.67138606	-0.89020962	-0.85496298	-1.39779434
IMAGE:1635874	CTNND1 Hs.166	0.497086965	1.4126756	0.00436055	-1.05959757	-0.91036135	-0.90013561
IMAGE:195458	UBE2D3 Hs.518	1.237358767	0.87189146	0.12555381	-1.40369666	-0.95769725	-0.9594237
IMAGE:322615	C5orf21 Hs.127	-1.332516306	-1.11421498	-0.04765708	1.67393474	1.34674263	0.70899091

IMAGE:49555	MDM4 Hs.49749	-0.131885048	-0.47557936	-1.47905755	0.96253281	0.86446444	0.9330213
IMAGE:813401	Hs.597993 Tran	0.923720744	0.93235652	0.31476519	-0.68820866	-1.03074616	-1.45861756
IMAGE:363936	ASNS Hs.48920	-0.857088977	-0.40676981	-0.51413067	1.93324965	0.27431309	0.33088878
IMAGE:1629917	CEECAM1 Hs.49	-0.961895766	-0.68035287	-0.44116091	1.91486762	0.58212682	0.51782964
IMAGE:743038	VCY Hs.170076	-1.05241943	-0.66761611	-0.21415723	0.59932807	1.41738118	0.83104058
IMAGE:263049	PCMTD1 Hs.308	-0.903071395	-1.42761186	-0.18814217	1.37155046	0.89384397	1.46580816
IMAGE:809719	CCDC80 Hs.477	0.47035154	0.21378083	1.35416945	-0.88803212	-1.18912313	-0.64175512
IMAGE:283301	FAM13A1 Hs.97	-0.778055597	-1.01407665	-0.56424829	0.85396054	1.11080698	1.42874121
IMAGE:1556889	AA935952::AI	-0.306186792	-0.99522868	-0.11954867	2.33414577	-0.55494719	0.32236047
IMAGE:884644	RPSA Hs.44990	0.418699657	1.14089921	0.64367576	-1.66014082	-0.91488924	-0.56896295
IMAGE:429574	CASP3 Hs.1411	0.666078644	1.44325161	0.24730445	-1.10247156	-1.67089972	-0.69975466
IMAGE:210501	H64973::H64	-0.783384894	-0.58477545	-0.36092606	-0.0669427	1.43411024	1.13623055
IMAGE:1540010	AI124708 310	-0.838383082	-0.68978755	-0.75301969	1.17924914	1.05840549	0.99587593
IMAGE:417424	TRPV6 Hs.3027	-0.50845071	-1.30861391	0.02967919	0.86022024	0.72208856	1.10618913
IMAGE:1900367	CINP Hs.129634	1.107045997	0.8660618	0.39561874	-1.39368893	-1.2703155	-0.79018068
IMAGE:281190	SSPN Hs.18342	-0.95713186	-1.24388425	0.92493315	0.51964297	0.71956189	0.90615288
IMAGE:1874679	Hs.150064 MRN	-0.566037482	-1.03215623	-0.25542878	2.00392382	0.36928554	0.3433672
IMAGE:770859	ITGB5 Hs.5366	1.143014406	-0.21198819	1.02565198	-1.26771659	-0.87828671	-0.53260101
IMAGE:782756	TBPL1 Hs.48650	0.497255723	0.49116461	0.78780202	-1.99660003	-0.33258289	-0.1382001
IMAGE:731423	C14orf140 Hs.4	-1.060252831	-0.94358098	-0.58128417	1.19939443	1.23521473	1.29774675
IMAGE:154482	SERPINB1 Hs.3	0.452771513	1.35741146	-0.24298401	-1.44091409	-0.56057037	-0.41005999
IMAGE:595529	ZNF83 Hs.4672	-0.438169259	-0.77413563	-1.12020042	1.15485511	1.1490291	0.91482364
IMAGE:1553319	AA934775 114	-1.069863746	-0.27329767	-0.96979263	2.32818744	0.82166228	0.07713319
IMAGE:740888	Hs.634364 **T	-1.261992934	-0.43026269	-0.45314671	0.89805087	0.72330015	1.48346581
IMAGE:1751052	CARD11 Hs.520	0.374540051	1.38778911	0.21684165	-0.75974622	-0.95180297	-1.20299662
IMAGE:197051	SGCE Hs.37111	-1.412354498	-0.32564327	-0.41687335	1.24438614	0.98981397	0.89388823
IMAGE:795440	Hs.537531 Tran	-0.645036698	-1.05546975	-0.30512877	1.36565168	0.79100929	0.77550966
IMAGE:233380	MYBPHL Hs.443	-0.656086562	-1.41290196	-0.42255959	1.83759006	1.25730319	0.53678901
IMAGE:298362	Hs.572124 Tran	-0.712462493	-1.39750936	0.06863592	1.59426974	1.00423234	0.48066081
IMAGE:308726	ETNK1 Hs.2946	-1.087481645	-0.49889622	-0.97336814	1.16465841	1.61310445	0.81851412
IMAGE:283400	Hs.632945 Full	-0.256784297	-1.41177671	-0.73927942	1.49846911	0.92668873	1.00178295
IMAGE:914079	AA572718::AI	-0.905519034	-0.84800752	-0.41429009	1.4502553	0.83576121	0.86213592
IMAGE:1899812	Hs.527860 Tran	0.368993398	1.26029271	0.42782727	-0.86528612	-0.88629822	-1.22712891
IMAGE:309676	FTSJ1 Hs.23170	0.947267762	1.00047172	-0.03111163	-1.21004127	-0.84685257	-0.82582585
IMAGE:154184	P4HA2 Hs.5195	-0.459560223	-0.77037305	-0.57471383	2.34616247	-0.03588667	0.2530164
IMAGE:365813	UBE2W Hs.1288	-1.085337154	-0.79510929	-0.07761813	0.935914	1.05541749	0.92636084

IMAGE:1128376	WDR71 Hs.5250	-1.154691934	-1.24506077	-0.13503019	1.63523084	1.17249397	0.960692
IMAGE:67009	EBF2 Hs.584959	0.646642065	0.78585178	0.91006969	-1.99432692	-0.66085844	-0.63114252
IMAGE:2302145	GNA14 Hs.1267	0.623247534	1.52705524	-0.30624214	-0.61220327	-1.12189154	-1.10855668
IMAGE:39937	CHN1 Hs.38013	1.227905152	0.91249993	-0.56917111	-0.6731448	-1.03622756	-0.78977138
IMAGE:207558	HBA1 Hs.449630	0.361904866	0.6204668	0.98335285	-0.64083453	-0.66138208	-1.40053195
IMAGE:593690	TNFSF13B Hs.52	0.472250766	0.97944953	0.56146341	-1.22675186	-0.73087451	-0.92175332
IMAGE:240966	PCBP2 Hs.54627	-0.562245515	-0.87626886	-1.47620604	1.17050477	1.25483329	1.57769105
IMAGE:131091	ALG3 Hs.478481	0.854451949	0.66310003	0.76070884	-1.82906537	-0.98827665	-0.409872
IMAGE:1631248	IFT140 Hs.3894	1.005958006	0.1583498	1.01416378	-1.51063226	-1.15512916	-0.34840502
IMAGE:811897	MKL1 Hs.474905	0.317133813	1.37241128	0.5693266	-1.32001275	-1.03471594	-0.8912472
IMAGE:321495	Hs.323090 Tran	-0.809146538	-0.90016516	-0.40736436	1.18562397	1.22463195	0.66291706
IMAGE:288743	N59220 14270	-1.062999431	-0.97978185	-0.03526226	1.18155221	1.11142405	0.81527347
IMAGE:505064	MAFG Hs.25222	0.451832642	1.30533241	0.39221017	-0.93643169	-0.9480922	-1.2414864
IMAGE:1621805	ASB6 Hs.125037	0.833453565	0.74765633	0.67043882	-2.06676107	-0.46610642	-0.67684588
IMAGE:74908	ATXN3 Hs.53261	-1.250586789	-0.38206029	-0.42944109	1.4042566	0.7048865	0.87662888
IMAGE:502917	TMEM68 Hs.420	-1.352475289	-0.55307378	-0.5714207	1.49555641	1.4857277	0.59131536
IMAGE:969636	SNN Hs.459952	1.23034298	0.16912837	0.81289837	-1.72647252	-0.57900279	-0.80985468
IMAGE:278516	C3orf15 Hs.3419	-0.778591084	-0.93721281	-0.31990993	1.52852884	0.5767985	0.86826592
IMAGE:1856915	Hs.435804 Tran	-0.909746523	-0.87237838	-0.20833258	1.19251155	0.95999865	0.78109288
IMAGE:280286	SURF2 Hs.15944	0.654824449	-0.4521729	1.72398772	-2.01002646	-0.20239787	-0.24653764
IMAGE:758284	TDRD6 Hs.4051	-0.777747384	-0.97354025	-0.37686979	1.26530137	0.92681201	0.90590531
IMAGE:1967131	KCNH2 Hs.4388	0.384312005	1.44718124	0.01285944	-0.69271656	-0.8526986	-1.217899
IMAGE:1854323	ZNF544 Hs.4389	-1.262930432	-0.67490648	-0.04322621	0.33698473	1.39943709	1.22436632
IMAGE:504207	C6orf108 Hs.109	0.677152989	0.88662384	0.61795471	-1.81304557	-1.0002076	-0.30485546
IMAGE:753969	DLG5 Hs.500245	0.41999546	0.82941809	0.86581121	-2.00628854	-0.40271657	-0.54737923
IMAGE:1323775	Hs.446264 Tran	-0.740349304	-0.70095322	-0.53031891	2.195398	0.52598873	0.10346568
IMAGE:742874	AA406213::AA	-1.022900057	-0.53593313	-0.605371	1.83194358	0.82344108	0.43957887
IMAGE:770452	TIA1 Hs.516075	0.151154063	-1.47216979	-0.73797347	0.56389279	1.18559129	1.15450636
IMAGE:415134	STAT5B Hs.6322	0.479304696	1.3994851	-0.0848059	-1.02348993	-0.95725472	-0.73143267
IMAGE:1872080	AI280873 220	-0.788765913	-0.60399845	-0.56504748	2.24316822	0.33307219	0.21921548
IMAGE:141416	GATAD2B Hs.47	-1.050322126	-0.57553185	-0.29130366	1.67738028	0.62761623	0.49791404
IMAGE:289023	MFAP3L Hs.1781	-0.480023252	-1.24090122	0.2004052	0.55249299	0.94262469	0.83576253
IMAGE:1574815	SPATA17 Hs.171	-0.613462281	-0.93761046	-0.61423406	1.67990447	0.68177982	0.7327174
IMAGE:134742	Hs.633153 Tran	-0.979808792	-0.59964275	-0.36647424	1.57617425	0.617311	0.63378486
IMAGE:49922	LOC643580 Hs.5	-0.624843141	-0.45182239	-0.54986748	2.23179413	0.28177969	-0.21124116
IMAGE:752631	LETM1 Hs.12016	0.734036214	0.55917851	0.72681069	-1.89706128	-0.50821216	-0.44306201

IMAGE:271076	Hs.594207 Trar	-1.071545294	-0.81099007	-0.4107329	1.87023403	0.63121079	0.83577436
IMAGE:77391	T55353 12070	0.275831927	1.28103652	0.5937777	-1.53242929	-0.93462039	-0.61047512
IMAGE:814667	SFRS11 Hs.4796	-0.578545404	-0.62818843	-1.08032188	0.65355642	1.35237753	1.15456915
IMAGE:549221	MGP Hs.365706	0.589672291	-0.62066143	1.58366499	-0.81489059	-1.06296	-0.05524693
IMAGE:303128	UBXD4 Hs.5915	-0.92611277	-0.59297516	-0.69249116	1.41169811	0.98110003	0.75144772
IMAGE:428733	PRKCD Hs.1553	0.775406985	0.85587929	0.49233381	-1.9836097	-0.42860073	-0.65013625
IMAGE:813823	LUM Hs.406475	0.902271496	0.86407075	0.19719488	-0.67173564	-1.22428214	-0.99998919
IMAGE:897299	NHLRC2 Hs.594	-1.328364998	-0.86344256	0.25434442	0.83870383	1.65114653	0.47993046
IMAGE:1521918	RGS7 Hs.13017	-0.64988721	-0.73666181	-0.28962635	2.04445613	0.29442036	0.10297998
IMAGE:510381	KLF6 Hs.4055 K	-0.687684944	-0.91086541	-0.30195073	0.27029669	1.29032333	1.21464393
IMAGE:256515	HNRPLL Hs.445	0.926267453	0.9239533	0.32921612	-1.72783401	-0.76699791	-0.69201937
IMAGE:1435264	TAF4B Hs.36951	-0.280042569	-0.28681672	-0.61887773	2.55247753	-0.5115002	-0.41709123
IMAGE:1674813	AI076396 565	-0.970340732	-0.94743904	-0.59589807	1.0379945	1.41630182	1.16724593
IMAGE:587992	ANP32E Hs.385	0.462226107	1.5708588	-0.0990192	-0.74712389	-1.23709995	-0.94162954
IMAGE:810999	GPX1 Hs.76686	0.528068809	0.98475586	0.42942441	-1.78952663	-0.80965889	-0.20683199
IMAGE:260015	COBRA1 Hs.521	0.850117932	0.2345851	1.04642796	-1.89065437	-0.70041019	-0.34402493
IMAGE:34773	PTPN11 Hs.506	-0.752504647	-0.66359103	-0.82606046	1.68918451	0.70103091	0.76650366
IMAGE:758309	PRICKLE1 Hs.52	-0.867052895	-0.76182281	-0.56695228	1.15239039	0.52100988	1.47860365
IMAGE:282741	N49969::N52	-0.68566931	-1.15201502	-0.44583437	1.47308305	1.07097884	0.76975758
IMAGE:2407298	JAG2 Hs.433445	1.195350131	0.42082443	0.84118171	-0.95549729	-1.66615655	-0.85408513
IMAGE:1533970	Hs.600085 CDN	-0.575718913	-1.27762961	-0.11743139	1.22608586	1.01876149	0.68196468
IMAGE:1499830	C22orf35 Hs.64	0.584529643	1.31243728	-0.29067984	-0.64939207	-0.92311903	-0.90958949
IMAGE:1472563	EFTUD2 Hs.151	1.001504809	0.7773061	0.51660992	-1.83669445	-0.8043376	-0.67294672
IMAGE:244194	COBLL1 Hs.470	-1.123463676	-0.4924551	-0.51059187	1.52488311	0.8003568	0.73687809
IMAGE:1456974	Hs.180848 Trar	-0.616668125	-0.71435272	-0.80548863	2.03091188	1.0649804	-0.09250022
IMAGE:1941469	R3HDM1 Hs.412	-0.888711354	-0.02264144	-0.8323161	1.83840181	0.5871322	-0.01810968
IMAGE:325365	KRR1 Hs.20555	0.764725608	1.01744173	0.25688656	-0.97826347	-1.1136471	-0.90244863
IMAGE:611443	MB Hs.517586 M	0.285585936	1.05512062	0.81726445	-1.95231088	-0.61811762	-0.4622119
IMAGE:230251	GCLC Hs.27126	-0.730632268	-0.37114565	-1.18630585	1.570325	1.26427558	0.3009486
IMAGE:502486	TANK Hs.55649	-0.767216572	-0.38445993	-1.04213083	0.05618517	1.778171	1.19582213
IMAGE:2322039	PPL Hs.192233	1.111951333	0.05578122	0.84204119	-1.78281235	-0.82819705	-0.19314092
IMAGE:275173	SCRG1 Hs.7122	-1.027786819	-1.05277837	0.01167656	0.68266883	1.00335516	1.4202281
IMAGE:1915867	C10orf118 Hs.1	-0.788510944	-0.91024131	-0.8328208	1.63990842	1.36311675	0.58612921
IMAGE:1456128	UBIAD1 Hs.522	-1.070800302	-0.47730131	-0.225674	0.31975564	1.20619314	1.07829614
IMAGE:936683	AA524273::AI	-0.681651378	-1.14984258	-0.33001341	1.30094879	1.15876044	0.70004846
IMAGE:23869	FAIM2 Hs.56742	-1.259413327	-1.118609	-0.00873958	2.09214384	0.43976362	1.0460505

IMAGE:180561	GSTM2 Hs.2798	-0.928819371	-1.02574471	0.5244733	0.16905941	1.07738831	1.02980678
IMAGE:1415129	SYN2 Hs.44550	-0.904643723	-0.5148825	-1.00074923	1.7617502	0.72995079	0.88852488
IMAGE:810961	ADCK1 Hs.4132	-1.021352549	-0.57422206	-0.22745809	0.28234202	1.15603024	1.23931226
IMAGE:768246	PRKCA Hs.5317	0.406591662	1.67081463	-0.28795696	-0.88867787	-1.13550235	-0.73198302
IMAGE:565097	AA126567::AI	-0.6671996	-0.40492511	-0.35820746	2.06972693	0.30913256	-0.3229131
IMAGE:51447	FCGR3A Hs.372	0.829192351	0.98961288	0.50193448	-1.39767369	-0.94391278	-1.01403947
IMAGE:2488862	FKBP9 Hs.5754	0.387998564	0.04732685	1.57157778	-1.50854331	-0.76804132	-0.34087571
IMAGE:897527	SEPT11 Hs.1281	-1.131116331	-0.37337114	-0.42580583	0.31131718	1.48630181	0.99136951
IMAGE:293917	SPARC Hs.1117	0.674679732	0.55206525	0.80045846	-1.22060402	-0.9773527	-0.64273382
IMAGE:205049	HSPB8 Hs.4000	1.259726422	0.21788499	0.41797374	-1.16083	-1.32021104	-0.25784326
IMAGE:341834	Hs.27278 Trans	1.289263499	0.80028829	0.04986102	-1.21723342	-1.22471774	-0.75470279
IMAGE:1643566	CA9 Hs.63287 C	0.576331174	1.18346468	0.71699379	-1.72205989	-0.90152797	-0.91234817
IMAGE:452068	C20orf44 Hs.18	-1.364426637	-0.59280815	-0.35573116	0.9149232	1.50202422	0.96356871
IMAGE:784146	GPATC3 Hs.109	0.84365257	0.74444341	1.07419577	-1.51752552	-1.18682831	-1.02053485
IMAGE:376941	MAL2 Hs.20108	1.628499208	-0.2350135	0.28242867	-0.97733476	-0.87320553	-0.59272411
IMAGE:429811	PCMTD1 Hs.308	-0.739186638	-0.63463141	-0.72760326	1.44530888	1.06964927	0.455273
IMAGE:1934935	GPC4 Hs.58367	-0.991658567	-0.8599758	-0.3577819	0.84426638	1.20094128	1.17947127
IMAGE:2116188	HDAC5 Hs.4387	0.636941214	-0.03482746	1.46962192	-1.37582615	-0.77240603	-0.59196584
IMAGE:1916839	DDX19B Hs.221	1.400958359	0.70949721	0.39731277	-1.56924381	-1.00443644	-1.08864408
IMAGE:324861	EGFR Hs.48829	-0.842827852	-1.18491716	0.53696036	0.18676535	1.31933205	0.86431966
IMAGE:841470	CTSH Hs.14864	0.576104171	0.53136601	0.9438519	-2.05382896	-0.30232973	-0.48486145
IMAGE:123761	R01415::R014	-0.612147719	-0.69767907	-1.16055463	1.45745946	1.01370267	0.94427134
IMAGE:796181	GAS6 Hs.36920	0.694512221	0.35663687	1.06835214	-1.75014224	-0.77439653	-0.38762504
IMAGE:25621	CDC37 Hs.1609	0.75791757	1.06752979	0.31683924	-1.73731875	-0.93709024	-0.4598111
IMAGE:1588700	SPIRE1 Hs.5152	-0.389801008	-1.32223675	-0.31598318	0.72776207	0.86762743	1.3676461
IMAGE:878447	PPP1R14C Hs.48	0.944142118	0.53106642	0.97865224	-1.02371914	-1.567515	-0.84489397
IMAGE:239877	HDAC3 Hs.5196	1.10395108	0.51320695	0.43912951	-1.89360656	-0.7754123	-0.30563006
IMAGE:1126178	AA653120::AI	-0.874797899	-0.70647686	-0.64242691	1.07450766	1.31415524	0.78628287
IMAGE:757220	TUSC2 Hs.5179	1.262706418	0.15765478	0.82422526	-1.21007436	-1.18899353	-0.76175548
IMAGE:36348	PTPN13 Hs.4361	1.389602186	0.07057708	0.54074053	-1.3015353	-0.66301697	-0.9016423
IMAGE:1500480	MRPS16 Hs.180	0.562251414	1.08656552	0.29746124	-1.83973083	-0.69848149	-0.30683964
IMAGE:2030301	Hs.552087 CDN	0.779417379	-0.88791423	1.36378762	-0.57723299	-0.60317734	-0.36157892
IMAGE:25384	LOC646201 Hs.1	-0.966095709	-0.47117156	-0.6316875	0.44456352	1.53187788	0.96906887
IMAGE:138917	MYCL1 Hs.4379	0.857936573	0.20106048	0.93384858	-1.06299307	-1.1752005	-0.51761273
IMAGE:593658	BRWD1 Hs.1905	-0.325689161	-1.34609261	-0.61669773	0.94489959	1.15258443	1.18106081
IMAGE:502151	SLC16A3 Hs.500	0.489641626	1.04414716	0.48697573	-0.78425487	-0.88022698	-1.24492101

IMAGE:1925280	TUSC4 Hs.4370	0.81417138	0.88853844	0.64255508	-1.93083128	-0.95261839	-0.47380892
IMAGE:1020543	Hs.634750 Tran	-0.567158586	-0.9574134	-0.55621686	1.68738357	0.62603638	0.6687091
IMAGE:2578773	CSNK1D Hs.631	0.440630074	1.27887842	0.36755201	-1.86106018	-0.27411993	-0.90352265
IMAGE:2516260	LTBP1 Hs.49787	0.627600585	0.63191409	1.02961956	-1.64712802	-0.63776708	-0.89140137
IMAGE:1553065	TNFRSF19L Hs.5	0.489459126	1.40942417	-0.38972909	-1.70030631	0.06005923	-0.7209165
IMAGE:202560	MAGI2 Hs.5832	-0.990730812	-0.79648457	-0.39807314	1.4999606	0.90987861	0.76857529
IMAGE:292222	LOC644823 Hs.6	-1.03660095	-0.6485324	-0.4826242	1.01108369	1.23271	0.88718658
IMAGE:826973	USP9Y Hs.16585	-1.031232048	-0.38645965	-0.56991619	0.741115	1.62505676	0.47276102
IMAGE:754538	DRAP1 Hs.3567	0.724859573	0.64438129	1.00795377	-1.57752943	-1.05915461	-0.67711946
IMAGE:234664	Hs.633489 Tran	-0.162363729	0.0657915	0.95551554	1.26467997	-0.73595037	-1.57826569
IMAGE:126458	MT1M Hs.64353	0.777705804	0.7701824	0.22073117	-1.65514484	-0.56667807	-0.37592336
IMAGE:416227	MUS81 Hs.2887	0.947142806	0.94084069	0.67867267	-1.58536242	-1.24504816	-0.84990551
IMAGE:121574	PSME4 Hs.4138	-1.140192752	-0.53427014	-0.34024246	0.6742877	0.86713286	1.39557685
IMAGE:505997	KIAA0672 Hs.49	-1.405954168	-0.69073545	-0.15359867	1.1880869	0.78913709	1.35980877
IMAGE:1492114	RBP7 Hs.42268	-0.621770121	-1.18026063	0.04170702	0.76043397	0.92582386	0.96465453
IMAGE:51997	RP11-487F23.3	-0.919251776	-1.30567598	0.10753253	1.26556305	1.14066523	0.79674769
IMAGE:79043	C11orf67 Hs.50	-1.00174627	-0.68092486	-0.7903197	1.27897841	1.27897841	0.9539495
IMAGE:1607473	Hs.157101 CDN	0.844898261	0.80715635	0.48823719	-0.93321759	-0.98511272	-1.17004809
IMAGE:84264	ECHDC2 Hs.476	-0.842512271	-0.89052457	-0.24142324	0.46426402	1.3143519	1.12271839
IMAGE:202872	PTPLB Hs.47736	-0.771149596	-0.93136024	-0.74634279	1.24130278	1.37386417	0.87152629
IMAGE:187582	ABI3BP Hs.4770	-0.442643758	-1.02079049	-0.32985765	2.08416127	0.29864338	0.22466878
IMAGE:276412	MS4A7 Hs.5307	1.296929427	0.64634149	0.47277392	-1.3306907	-1.21741869	-0.9577644
IMAGE:113048	PTGFRN Hs.418	0.797950819	0.87476782	0.79795082	-1.06129502	-1.62096177	-0.8242597
IMAGE:2284803	SCAMP1 Hs.482	-1.499266532	-0.45183521	-0.52076295	1.02909753	1.47799667	1.0705121
*mitoch. cont. IMA	144621	-1.018295226	-0.45585008	-0.42444081	-0.01520718	1.46942888	1.28754726
IMAGE:2422534	AGPAT1 Hs.409	-1.300626803	-0.51683051	0.16865638	0.11002387	1.32651144	1.07883019
IMAGE:645284	FLJ23861 Hs.59	-1.120043097	-1.11308958	-0.32456121	1.27648515	1.21494656	1.26396883
IMAGE:724588	ISGF3G Hs.170	0.896509852	0.77729624	0.51758087	-1.67658823	-0.69520475	-0.78589225
IMAGE:866866	RASSF1 Hs.476	0.868348301	0.78918109	0.63480502	-1.70384392	-1.08782404	-0.48813241
IMAGE:645939	ZC3H13 Hs.136	0.923945914	0.82549596	0.21145994	-0.25508346	-1.25599134	-1.37741295
IMAGE:162365	TOMM70A Hs.22	-0.464325622	-1.16304846	-0.54942148	1.13863825	1.19719045	0.79200927
IMAGE:780977	TRAPPC3 Hs.52	1.15712079	0.7632343	0.06999408	-1.62568725	-0.74600743	-0.59633056
IMAGE:856977	KIF3B Hs.36967	-0.933518535	-0.66565404	-0.49577156	1.5782398	0.73537295	0.70486056
IMAGE:743116	TAF7L Hs.22380	-0.136883821	-1.12949605	-0.7362689	1.30069956	0.97550998	0.54369639
IMAGE:296345	FRMD4A Hs.330	-0.703780324	-0.7520981	-0.8809455	1.19732279	1.04255492	1.0451218
IMAGE:1202584	DNAJA2 Hs.368	-0.542780337	-1.45482075	-0.39263491	1.31857094	1.16434836	1.00427596

IMAGE:488017	MFGE8 Hs.3745	0.764132987	0.63808745	0.9069846	-1.82426473	-1.08374717	-0.32904949
IMAGE:383999	CAND1 Hs.5464	-0.462735665	-1.05999346	-0.55010595	1.49873205	0.68694901	0.78604505
IMAGE:234419	LOC54103 Hs.18	-1.148932084	-0.97566562	-0.17170924	0.74590168	1.22375958	1.43187185
IMAGE:137931	R63068::R631	-0.803067848	-0.74721901	-0.60309296	0.99625577	1.09984636	0.98319435
IMAGE:322169	CASR Hs.43561	1.374795069	0.51735263	-0.55678791	0.46434684	-1.50841702	-1.09816647
IMAGE:208181	H62537::H62	-1.139763742	-0.70590818	-0.15961922	1.59079211	0.97329738	0.40394242
IMAGE:488207	PDPN Hs.46867	0.547834426	1.20939217	-0.03250705	-1.29293619	-0.6581877	-0.64408218
IMAGE:142067	LOC389541 Hs.4	1.01285968	0.10531161	0.87191472	-1.52535288	-0.87563097	-0.36616648
IMAGE:1610125	AI000670::AI	-0.82794572	-0.76319011	-0.38356035	1.90627315	0.47106192	0.48881912
*mitoch. cont. IMA(	148009	-0.79491673	-0.83615017	-0.83615017	1.07424662	0.99693392	1.42060751
IMAGE:50542	APBA2BP Hs.51	-0.949919781	-0.58902784	-0.51833003	0.53575205	1.37217824	1.04840368
IMAGE:826133	BTBD2 Hs.4655	1.00599858	0.70069654	0.097725	-0.42583865	-0.6918037	-1.56455657
IMAGE:196636	NIP30 Hs.39674	-1.190926186	-0.06713358	-0.40969657	2.62338093	-0.16672486	-0.05744571
IMAGE:22411	DARC Hs.15338	0.579540863	1.08315405	0.27389226	-1.22520901	-0.98772403	-0.62347465
IMAGE:838155	SCD5 Hs.37919	-1.266882278	-0.81926624	0.03351153	1.08494776	1.05726017	0.94512543
IMAGE:757257	C6orf105 Hs.12	-0.874724911	-0.48600664	-0.80404886	0.73646815	1.19554696	1.11414329
IMAGE:296024	N73572 2534	-0.853509062	-0.66882737	-0.67685701	1.63025893	0.86995252	0.62936447
IMAGE:868356	Hs.116806 Tra	-0.535712004	-0.62149845	-0.60669337	2.03840016	0.29672782	0.15905441
IMAGE:345833	HNRPAB Hs.591	1.22752005	0.47632193	0.09963417	-1.62431108	-0.68803516	-0.36795944
IMAGE:869164	Hs.178949 Tra	-0.785532601	-0.87270478	-0.49601915	1.90229755	0.25111644	0.95396602
IMAGE:2315045	NCF1 Hs.52094	1.18017152	0.50311256	0.22730012	-1.55960101	-0.80058229	-0.44886797
IMAGE:81057	LOC400590 Hs.5	-0.436455717	-1.08853939	-0.45217559	1.36230545	0.91763679	0.57276991
IMAGE:127682	ZDHHC2 Hs.443	-1.319542024	0.16946418	-0.79431546	0.87748293	0.91081889	0.92970927
IMAGE:81315	RAB40B Hs.484	-1.016791123	-1.12646969	0.31510042	0.75008843	1.07406205	0.99686521
IMAGE:712209	ARHGAP15 Hs.1	0.301626623	1.29752725	0.32396458	-0.85203584	-0.81480591	-1.1368448
IMAGE:810509	RRBP1 Hs.4722	0.904505897	1.00339627	0.14799456	-1.32609004	-1.18393513	-0.53682127
IMAGE:666774	MOSPD2 Hs.190	-0.70105151	-0.67372833	-0.82400581	0.64171193	1.30976363	1.14070147
IMAGE:744985	C11orf66 Hs.50	-0.64629419	-1.47108417	-0.12064446	1.47200036	0.76787696	1.08699579
IMAGE:392127	DAP Hs.75189 D	-1.172107602	-0.19457809	-0.94697959	1.15285749	1.26906255	0.81183299
IMAGE:786573	GRB2 Hs.63306	-0.748558583	-0.7923183	-0.67217799	0.51709165	1.4144144	1.22003176
IMAGE:377348	CALML3 Hs.239	-0.6780387	-1.05101318	-0.07207029	0.59203148	1.15840013	0.93323406
IMAGE:86035	ISOC1 Hs.48329	-1.049332882	-0.39360324	-0.77984399	0.69974598	1.24012319	1.19934
IMAGE:1950530	TMEM107 Hs.51	-1.608713022	-1.00177993	0.36889267	1.99460631	0.75491319	0.70510409
IMAGE:813310	SFRS1 Hs.68714	1.347139875	0.43655435	0.44830384	-1.08280166	-1.2230612	-0.93005828
IMAGE:512410	RNASEH2A Hs.5	0.876039617	1.08181464	0.03335951	-1.10295945	-0.91750333	-0.95801798
IMAGE:151492	CHD4 Hs.16223	0.946254633	0.87474614	0.1292701	-1.82492295	-0.6886083	-0.37955753

IMAGE:179211	GPR160 Hs.231	-1.280449938	0.06776284	-0.63027287	1.08519657	0.63433321	0.88734197
IMAGE:357785	CDSN Hs.55603	0.878234594	0.26714116	0.77826193	-1.09709971	-0.71973189	-0.87405944
IMAGE:825284	TMEM109 Hs.13	0.93236514	0.56686143	0.61245893	-1.47199786	-1.09907551	-0.44334015
IMAGE:1686218	FGL2 Hs.52098	0.600399409	0.54230855	0.78647458	-1.39377685	-0.82983446	-0.47354385
IMAGE:471855	LUM Hs.406475	0.881243998	0.73412574	0.4284467	-0.77342774	-1.03905792	-1.14612732
IMAGE:1534734	ZDHHC14 Hs.14	0.872116718	0.66544641	0.54060067	-1.80342026	-0.86075058	-0.31792468
IMAGE:824622	LAS1L Hs.52267	1.185823205	0.9115886	0.18945579	-1.28036475	-1.12373777	-0.97883492
IMAGE:183005	FBXW8 Hs.4354	-0.713199505	-1.3572151	0.20903082	0.90771874	1.11126089	0.82535375
IMAGE:771010	HIP1 Hs.329266	0.711885742	0.71188574	0.8596052	-1.98479722	-0.55398796	-0.67137855
IMAGE:1156220	AA679634::AI	-0.769686847	-1.1524688	0.27830418	1.16587086	0.76731887	0.60216351
IMAGE:825853	Hs.535041 Tran	-0.374861263	-0.89907034	-0.42457074	2.3893813	0.12809418	-0.07586465
IMAGE:309993	ABR Hs.159306	0.616979149	0.73343451	0.7237299	-2.08502778	-0.36003278	-0.48522229
IMAGE:462019	Hs.634742 Tran	-0.796308849	-0.66922925	-0.3166482	1.77967891	0.60022138	0.2142171
IMAGE:711857	FGFR1 Hs.2648	0.684259457	0.26323609	1.47940739	-1.06032282	-1.02834637	-1.18183337
IMAGE:2504022	GBAS Hs.5910	-0.830000825	-0.76475993	-0.53061761	0.47294441	1.28362293	1.29884581
IMAGE:123405	KCTD5 Hs.6196	1.256522923	0.5761651	0.12552809	-1.44429754	-0.56243374	-0.89921086
IMAGE:345925	KRT5 Hs.433845	1.164262934	0.6238234	0.24774894	-1.66902484	-0.87176486	-0.45102598
IMAGE:1020434	Hs.584100 Tran	-0.923711589	-1.00766396	-0.13342097	1.49553959	0.77518557	0.79311438
IMAGE:432047	ABCC11 Hs.642	-0.689820449	-1.34360423	-0.12837302	1.44413718	0.93406353	0.83240258
IMAGE:2028590	GCGR Hs.208 G	0.825994038	0.80076886	0.15500436	0.59203053	-1.20053354	-2.02539679
IMAGE:841221	ASL Hs.632015	1.114991613	0.47717861	1.09136891	-1.95477873	-1.17522951	-0.62245824
IMAGE:824530	ARTS-1 Hs.4361	0.268765676	1.36438013	0.56260251	-1.28511333	-1.29375391	-0.57410461
IMAGE:564608	TMEM163 Hs.36	-1.168310105	-0.64574612	-0.33163662	1.57515084	1.05401462	0.50646465
IMAGE:785540	CAPNS2 Hs.460	1.298155741	-0.75371652	0.96177074	-0.50333471	-0.69355242	-0.82198513
IMAGE:1575061	GALT Hs.52209	0.225076892	1.11629983	0.75024631	-1.70235575	-0.71114194	-0.53637529
IMAGE:1646628	STXBP5 Hs.935	1.047839559	0.70301665	-0.41951541	0.14312661	-1.27276636	-0.9722503
IMAGE:233987	C13orf24 Hs.44	-0.889767771	-0.63275668	-0.66677286	1.44765629	1.04027212	0.62932435
IMAGE:2455026	SCTR Hs.42091	0.829824335	0.73718285	-0.18090142	0.93338171	-1.52377202	-1.5339937
IMAGE:128143	PON1 Hs.37099	-0.918090677	-1.07974599	-0.31188326	1.53358531	1.28244224	0.57058153
IMAGE:1914863	DYSF Hs.25218	0.27004105	1.29706402	0.43391575	-1.87856958	-0.96098348	-0.05349925
IMAGE:1557243	FPRL2 Hs.44546	0.744525286	0.86524578	0.34972455	-0.89976984	-0.87424714	-1.07779531
IMAGE:855451	ZNF521 Hs.116	-0.829986049	-1.11542828	-0.10577834	1.27015514	0.78803448	0.9921548
IMAGE:592777	FER1L4 Hs.7222	-1.027563142	-0.45665032	-0.54533018	0.71748842	1.3260757	0.86441878
IMAGE:230202	ZNF347 Hs.467	-0.528420306	-1.43192724	0.13538071	0.52526503	1.44984501	0.7961854
IMAGE:123400	T99639 8299	1.109475292	1.06144065	0.21324999	-1.09011105	-1.49988027	-0.93294507
IMAGE:683151	C10orf128 Hs.3	0.670664994	0.79376468	0.87761688	-1.73149672	-1.11046637	-0.45170252

IMAGE:845658	HIG2 Hs.433213	-0.848862507	-0.57727625	-0.56684734	0.68136309	1.11774788	1.04865634
IMAGE:981201	ITCH Hs.632272	-1.134170683	-0.27488783	-0.78646088	1.16251255	1.07858259	0.85556872
IMAGE:1010364	AA229894::AI	-1.40779987	-0.92824645	0.0568127	1.72046009	0.83090864	0.88168488
IMAGE:1899498	LYPLAL1 Hs.591	-0.983680257	-0.60254911	-0.26629006	1.70444428	0.66454766	0.34321468
IMAGE:813552	CD47 Hs.44641	0.658688626	1.01400985	0.23427717	-1.1755338	-0.91644541	-0.70991495
IMAGE:297800	RGS18 Hs.4408	0.841982866	1.09858604	-0.18164998	-0.55495415	-1.10194659	-1.02689016
IMAGE:841287	GNPAT Hs.4980	-1.184622389	-0.25557171	-0.42465296	0.33934932	1.29635261	1.05540542
IMAGE:347429	W81196::W81	0.684632438	0.80151107	0.73054905	-1.24115179	-1.45403787	-0.44721192
IMAGE:71956	CLIC2 Hs.63283	0.577596605	1.12631563	0.3352457	-0.83617144	-1.04684035	-1.0919137
IMAGE:2243166	MLN Hs.2813 M	0.774994649	0.90502819	0.57827724	-0.63989412	-1.68097899	-0.92200771
IMAGE:24176	RGS6 Hs.50987	0.723983565	0.81540979	0.39891256	-1.75417491	-0.52500018	-0.52855564
IMAGE:214713	VAPA Hs.165195	-0.2269284	-0.99091898	-1.01830646	0.77294485	1.25672103	1.06998826
IMAGE:782222	CIP29 Hs.50567	0.46083207	1.20154365	0.53532649	-1.20619226	-1.07286417	-0.88366527
IMAGE:704020	CSF2RB Hs.592	0.56733342	0.95501041	0.3844411	-1.34961525	-0.72078038	-0.69367149
IMAGE:450965	ANKRD39 Hs.63	0.931757201	0.99023944	0.60382348	-1.69247067	-1.12714239	-0.81816125
IMAGE:2029229	C21orf51 Hs.30	-0.767316997	-1.07782157	0.21241626	1.66155937	0.48177246	0.3588557
IMAGE:282980	Hs.596757 Tran	-0.986650182	-0.69832756	-0.90366953	1.95599858	0.81466295	0.88639199
IMAGE:212467	SPTLC2 Hs.4356	-1.136257359	-0.98500129	-0.35498688	1.50842212	1.17302823	0.94417122
IMAGE:233583	IL1R2 Hs.25333	0.970296488	0.72475105	0.18773407	-0.83912332	-1.0744377	-0.86367787
IMAGE:432492	TNRC6A Hs.407	-0.883772925	-0.96033044	-0.37645179	0.41591851	1.35757595	1.46322532
IMAGE:198169	R94923::R95	-0.687633717	-0.47419902	-0.2699448	2.0933569	0.09064246	-0.10381925
IMAGE:51408	DSCR1L1 Hs.44	-1.329672892	-0.51726615	0.22587564	1.25368345	0.77303644	0.46134413
IMAGE:2018501	MOSPD3 Hs.521	0.790170704	0.5188273	1.1345681	-1.38501195	-1.23629489	-0.7604003
IMAGE:435714	AA699972 14	-0.977484245	-0.63676792	-0.23930892	1.38978711	0.92300488	0.40772241
IMAGE:435835	SCAND2 Hs.513	-1.181330052	-0.86215342	-0.25627779	1.30600782	1.28227956	0.79728507
IMAGE:2449845	ITGB1BP1 Hs.46	1.193432952	0.40551905	0.40963527	-0.78458426	-1.35056508	-0.77532275
IMAGE:1191176	EIF4A1 Hs.1296	1.215990887	0.61245935	0.01867428	-1.50683604	-0.71962099	-0.53956116
IMAGE:857249	PLEC1 Hs.43424	0.688895097	-0.27983503	1.32675302	-1.78313611	-0.3632166	-0.12567866
IMAGE:1115508	LOC401101 Hs.5	-0.809984767	-0.52580998	-0.52248873	2.05679111	0.27744911	0.32256281
IMAGE:2018680	HIP2 Hs.50308	1.102245676	0.99317202	-0.05767471	-1.27330057	-0.89465917	-0.90307343
IMAGE:283686	Hs.586700 CDN	-0.575906434	-1.28531176	0.02336744	0.83967966	1.03191362	0.89102473
IMAGE:504543	TEAD3 Hs.4852	1.176868134	-0.37293498	0.74257192	-1.92029822	-0.02606731	-0.18774912
IMAGE:795260	Hs.634293 CDN	-0.712615022	-1.39897591	-0.08738932	1.17360847	1.01569471	1.08731988
IMAGE:376290	TEAD1 Hs.5681	1.614975397	0.25969226	0.31462004	-1.03499121	-1.12454737	-1.04573795
IMAGE:1699812	AI049745::AI	-1.078325042	-0.61011342	-0.25239421	1.45108812	0.99427103	0.4027913
IMAGE:665261	Hs.633963 Tran	-0.759350119	-0.84463567	-0.57965382	1.17932931	0.85622025	1.09499639

IMAGE:796090	MAN2C1 Hs.598	0.479843414	1.23761315	0.7672346	-2.36474	-0.43242716	-0.73806095
IMAGE:648046	AA206914::AA	-0.580518701	-0.77155576	-0.42006727	2.12716081	0.45380173	-0.02776676
IMAGE:32310	DAD1 Hs.82890	-0.905854407	-0.7003241	-0.49096997	1.93882845	0.53767167	0.54648011
IMAGE:200899	AKR1C2 Hs.460	-0.391748086	-1.1997913	-0.48371478	1.07533125	0.96870956	0.94791175
IMAGE:119851	KLRK1 Hs.3877	0.449252603	1.07788072	0.19480045	0.03865935	-1.3070612	-1.26579869
IMAGE:1894160	LOC644450 Hs.5	-0.254020459	-0.40480971	-0.51147914	2.0918344	-0.12574987	-0.33849036
IMAGE:77577	FOSL2 Hs.2209	0.909958222	1.0760103	-0.15900202	-0.18157473	-0.79648632	-1.80213921
IMAGE:1553979	Hs.602361 Tran	-0.945500741	-0.86771656	-0.61282379	0.76964041	0.94615066	1.77006462
IMAGE:878835	NDP Hs.522615	1.151886078	0.58053546	0.44276297	-1.01404605	-1.12142755	-1.0166124
IMAGE:510466	KRT17 Hs.2785	-0.744091875	-0.73003806	-0.52942678	0.60934537	0.79121823	1.47241478
IMAGE:810613	DDX49 Hs.1431	0.734981376	0.59386962	0.89678952	-1.80902834	-0.84946842	-0.45576663
IMAGE:137045	Hs.633162 Tran	-0.921257418	-0.5265464	-0.79578086	1.50579984	0.89269937	0.76793261
IMAGE:76252	CHMP4A Hs.279	0.498823799	1.14591665	0.64273725	-1.60215728	-1.05083417	-0.61754079
IMAGE:83746	METTL7B Hs.514	0.525464152	0.92588937	0.66434581	-2.00811236	-0.64717349	-0.35217669
IMAGE:193182	TACC1 Hs.2792	0.761375442	0.52408516	0.59813703	-1.69841671	-0.5835848	-0.39386068
IMAGE:782843	C14orf8 Hs.406	1.793736272	-0.88363096	0.39783769	-0.20420318	-0.67284432	-0.98540759
IMAGE:869448	AA680243::AI	-0.800943255	-0.7901609	-0.57235728	1.3938056	0.77921123	0.929086
IMAGE:1190928	ZNF10 Hs.5073	0.335749551	0.70895966	1.00482984	-1.90283663	-0.73599297	-0.18427152
IMAGE:1470305	AA866057 24	-0.543291438	-0.98991728	-0.53517097	0.85212431	1.05658614	1.06006634
IMAGE:80770	SHANK2 Hs.268	-0.925264864	-0.76269111	-0.42411358	1.35129789	0.91187751	0.79890053
IMAGE:1541632	AA928058 83	-1.215212352	-1.00039455	0.17632961	0.41956601	1.37841421	1.30501813
IMAGE:1669248	SLC1A3 Hs.481	-1.41914443	-0.78259432	0.25207304	1.13735529	1.15193834	0.69822321
IMAGE:23539	R38110::T770	0.964672735	0.60219133	0.69788642	-1.66927826	-0.98475226	-0.56862361
IMAGE:48283	EPHA5 Hs.4798	1.383334534	0.79531786	0.32992284	-2.23711361	-1.42709064	-0.01617789
IMAGE:714493	SRGAP2 Hs.497	0.86716374	0.31295319	1.06339533	-1.38806267	-0.85779949	-0.85355739
IMAGE:2029080	ERBB4 Hs.3907	1.271397041	0.36490359	0.42195016	-0.974714	-1.25091123	-0.75626341
IMAGE:46050	MLSTD1 Hs.298	-0.899962247	-0.96241102	-0.3347116	0.78570867	1.34635372	1.07988702
IMAGE:564981	C18orf24 Hs.13	-0.03601476	1.55165607	0.13361718	-0.79778133	-0.92623196	-0.71647015
IMAGE:2114193	CILP Hs.442180	1.136366395	-0.80294703	0.81739787	-0.37559391	-0.448675	-0.69760748
IMAGE:117546	ITGBL1 Hs.6434	-0.403930708	-0.76331235	-0.64896832	1.86043068	-0.25164338	0.95328768
IMAGE:753420	DCUN1D4 Hs.22	-0.483641956	-1.47027335	-0.10119515	0.88857245	1.26225243	0.90654202
IMAGE:245534	ZNF704 Hs.632	-0.59763977	-0.21121066	-0.63231312	2.14986059	0.20853964	-0.35473319
*mitoch. cont. IMA	144076	-0.773765796	-0.85961337	-0.3125231	0.83486478	0.95811182	1.04774603
IMAGE:308275	Hs.597449 **T	-0.894628004	-1.09411733	-0.30723615	0.95249428	1.41072424	1.00394466
IMAGE:809456	IRF7 Hs.166120	0.940904156	0.62703399	0.50809371	-1.37165821	-0.98807582	-0.62729032
IMAGE:2310335	FCGR2B Hs.352	1.081810547	0.39445775	0.44283589	-1.15831292	-0.75236763	-0.85726677

IMAGE:449061	AA777417 934	-0.537614275	-0.59517251	-0.5391699	1.95341269	0.4600099	-0.04028003
IMAGE:810959	ARHGDIA Hs.15	0.673816792	0.69988355	0.64045134	-2.14738879	-0.58703245	-0.12669345
IMAGE:282320	BCL2L13 Hs.631	0.762034168	1.03260807	0.6007509	-1.23198939	-1.31156995	-0.89934265
IMAGE:362875	Hs.130643 Full-	-0.301915174	-1.09558722	-0.85225261	0.85654076	1.22957389	1.0754547
IMAGE:324651	MECR Hs.18364	-1.026613181	-0.50596445	-0.32257177	0.34533507	1.24675675	1.10998933
IMAGE:1942064	GRK5 Hs.52462	-0.769470198	-0.28269079	-0.66899893	2.03162542	0.4519567	-0.06909198
IMAGE:1693563	POU6F1 Hs.5948	-1.184948439	-0.72913909	-0.05931174	1.33772757	0.96537436	0.64216904
IMAGE:1009152	AA225452::AA	-1.190714907	-0.42242417	-0.1770055	1.31668201	0.80236158	0.52192189
IMAGE:1861415	LRRC16 Hs.1454	0.519845722	0.95695548	0.62025113	-1.76026591	-0.84493211	-0.3853177
IMAGE:1697156	AI095492 161	-1.125785595	-0.96058791	-0.22545822	0.9931697	1.5064625	0.91175084
IMAGE:1118167	PDE4DIP Hs.584	0.411718105	1.6481766	0.29243623	-0.87819903	-1.45678887	-1.12039943
IMAGE:723955	EFNB2 Hs.1492	-0.392096996	-1.11088885	-0.37172943	0.43068798	1.09276855	1.19568403
IMAGE:1912788	Hs.543417 Tran	0.736014272	1.21516002	0.28949121	-1.03707986	-1.28939597	-0.96214962
IMAGE:151745	FBXO45 Hs.518	1.079816029	0.57848742	0.70657557	-1.52840512	-0.89761037	-0.94465914
IMAGE:1010100	AA229499::AA	-1.397229579	-0.86323834	-0.81073292	1.47101837	0.77280806	2.16029159
IMAGE:809587	ARHGEF18 Hs.4	0.720994703	0.88659059	0.72572601	-1.97200841	-0.7743953	-0.57213175
IMAGE:1601279	Hs.341780 Tran	-1.188398387	-0.99478603	0.09276226	1.40337964	0.90344487	0.8519993
IMAGE:844906	AA773755 285	-0.607161284	-0.72050084	-0.72201203	1.775425	0.70436624	0.41421698
IMAGE:1169174	AA640825::AI	-0.683707619	-0.65551362	-0.76400699	1.58127	0.44637221	0.93619838
IMAGE:1560382	LOC441212 Hs.1	-0.818050163	-0.91293059	-0.35369421	2.05989635	0.60320278	0.37548976
IMAGE:810083	COMMD5 Hs.631	0.828159233	1.21443375	0.5915661	-1.85041308	-0.63123416	-1.32169985
IMAGE:771058	CISH Hs.8257 C	0.203673688	1.16379709	0.44853056	-1.32884075	-0.55921802	-0.7238106
IMAGE:2011066	MMP19 Hs.5910	0.559490977	0.87266328	0.57405713	-1.1990079	-0.72560791	-0.94118698
IMAGE:290391	Hs.146317 Tran	-1.296885491	-0.47508935	-0.57156107	0.63344219	1.24085674	1.49811466
IMAGE:257926	NPLOC4 Hs.464	-1.037659254	-0.51183158	-0.71955524	1.25751337	1.12062434	0.84554259
IMAGE:252953	FAM3C Hs.4340	-0.69074284	-0.49674175	-0.84647522	0.50289535	1.33603701	1.00038855
IMAGE:399577	PKP1 Hs.497350	0.980527795	0.15057803	0.87072899	-1.39726213	-0.87749398	-0.51031387
IMAGE:194353	NUP214 Hs.4618	0.94407733	0.60764196	0.82686759	-1.55395533	-1.24465184	-0.56255625
IMAGE:815284	PEPD Hs.36473	1.257136689	0.49221404	0.29517497	-1.24300315	-0.72359744	-1.02639422
IMAGE:2249736	SELP Hs.73800	0.388786066	1.07679934	0.50517929	-0.88382294	-0.89891095	-1.04711832
IMAGE:625786	RPL22L1 Hs.380	1.021567103	0.83297235	0.21165099	-1.21090882	-1.04314049	-0.7923236
IMAGE:248599	AMDHD1 Hs.424	0.982001382	0.40824299	0.73488819	-0.79664962	-0.86270089	-1.34462629
IMAGE:916140	AA570243::AI	-0.972700607	-0.53922304	-0.48213559	1.51419723	0.82107891	0.53527883
IMAGE:487499	Hs.642841 **C	-0.69105432	-0.91772594	-0.66824695	0.60190864	1.18377702	1.46279342
IMAGE:742776	YPEL1 Hs.51743	1.01680224	0.66149791	0.70341584	-1.06686801	-1.18663351	-1.14321851
IMAGE:1436413	AA877935::AI	-0.903197684	-0.80326127	-0.30183029	1.2837424	0.88296502	0.77026886

IMAGE:71672	ETFA Hs.39925	-0.922348596	-0.28991877	-0.91990048	0.39412548	1.09795867	1.4761925
IMAGE:243238	YWHAZ Hs.4924	1.276676796	0.10886767	0.51177643	-1.62906779	-0.6645919	-0.42437755
IMAGE:381107	PVRL1 Hs.33484	1.03862386	0.19592402	0.70440053	-1.96724362	-0.52465576	-0.2404231
IMAGE:565644	ZNF182 Hs.1896	-1.392643096	-0.68391197	-0.44136842	0.54967394	1.70726813	1.40960105
IMAGE:124829	KIAA1641 Hs.54	-0.844392497	0.09291608	-1.07082475	1.61360685	0.4293729	0.42276582
IMAGE:243770	HMBOX1 Hs.591	-0.964976652	-0.73431123	-0.23200797	1.67966101	0.80731428	0.3519665
IMAGE:340903	PPARA Hs.10311	-0.893680442	-1.28669639	-0.291056	1.32904306	1.5528438	0.75249837
IMAGE:1555604	AA975065::AI	-0.598621124	-0.94072521	-0.5284308	1.07400241	1.08137534	0.81418026
IMAGE:79000	MOSC1 Hs.4978	-1.020502843	-0.4970185	-0.58116138	0.92618448	1.10261958	0.97392968
IMAGE:277621	CCDC82 Hs.525	-1.152851075	-0.55181978	-0.45851282	1.0020878	1.28342719	0.84463231
IMAGE:1856927	VIT Hs.137415 \	0.320225507	-1.01553697	1.64601961	-0.2674103	-0.45880313	-0.28834389
IMAGE:2455836	ADAMDEC1 Hs.5	0.704636718	0.1812163	1.11283881	-1.47414193	-0.88652844	-0.35915767
IMAGE:452395	LGR4 Hs.502176	-0.574464503	-0.70101772	-0.56442923	1.259828	0.14173217	1.21719971
IMAGE:1020181	PRSS12 Hs.4458	0.894173971	0.87974191	0.28802743	-0.73670899	-1.26738788	-1.01681123
IMAGE:812161	PARD6G Hs.352	1.120007197	0.05246976	0.80345724	-1.45550875	-0.62592955	-0.68159868
IMAGE:489563	ARHGDIA Hs.15	0.468748459	0.42094294	0.90951538	-2.19330208	-0.23464152	-0.04348772
IMAGE:192242	RPS19 Hs.43842	0.508548253	0.66273949	0.87729495	-1.60534794	-0.78354147	-0.46466088
IMAGE:214635	RORA Hs.56949	-0.861460991	-1.07971069	0.60402153	0.14341036	0.99093331	1.02238694
IMAGE:2244016	SEZ6L Hs.19476	0.706628869	1.06578367	0.41930503	-1.51677342	-0.81010949	-0.85586719
IMAGE:241447	WHDC1L1 Hs.55	-0.903946336	-0.66498064	-0.55027711	1.65059695	1.0200301	0.37060979
IMAGE:949971	ATF4 Hs.496487	1.231943524	0.58279211	0.36815334	-1.64133675	-0.80895711	-0.73200126
IMAGE:754093	NID2 Hs.369840	0.132253973	1.65137915	-0.13347679	-0.72099574	-0.63464486	-1.15296309
IMAGE:430465	CCL19 Hs.50002	0.65339016	0.56376681	0.70220628	-1.31279357	-0.48949823	-0.90120151
IMAGE:124597	R02373::R024	-1.23728453	-0.31999307	-0.45878326	0.67486934	1.46633235	0.76819378
IMAGE:502153	MARCH9 Hs.632	0.370109857	0.82939338	1.14062552	-1.58508713	-1.17173196	-0.46821766
IMAGE:1879640	IGSF10 Hs.6435	0.08075978	0.55782289	1.29698217	-0.73161811	-0.87468895	-0.97279467
IMAGE:234856	VHL Hs.517792	0.516654456	0.76304819	0.75856831	-1.76752749	-0.50755952	-0.59267735
IMAGE:360878	PRKAG1 Hs.530	-0.88264872	-0.5189296	-0.65586941	1.60481499	0.64136637	0.6760229
IMAGE:795901	Hs.642795 CDN	-0.783802212	-1.187425	-0.26335021	1.19548219	1.0230484	1.067498
IMAGE:727207	Hs.599366 Tran	0.339520964	1.26545959	0.55028223	-0.85251478	-1.03182353	-1.21098531
IMAGE:1627623	STK32C Hs.4690	0.755441355	1.11845377	0.46389701	-1.55507595	-0.72978991	-1.10584809
IMAGE:1687041	GIPC1 Hs.63163	-0.660536525	-0.85223428	-0.46565266	1.89999017	0.55727144	0.39396043
IMAGE:1384997	AA848149::AI	-1.114271896	-0.46004959	-0.48353449	1.13239461	0.81283217	1.02067357
IMAGE:128679	C1QTNF6 Hs.220	0.473501125	0.85507879	0.84126304	-0.98488211	-0.9947505	-1.06481605
IMAGE:143523	COL5A1 Hs.2102	0.935037772	0.33157496	0.93353849	-1.14348868	-1.0155808	-0.90777273
IMAGE:561918	EIF2B4 Hs.1694	1.393147601	0.90178174	0.18478067	-0.90133443	-1.09001892	-1.68201651

IMAGE:244147	RPS3A Hs.3565	-0.063205533	0.30580631	1.52402933	-1.3104353	-0.44386242	-0.5146401
IMAGE:261834	PLXNC1 Hs.584	0.509301107	0.92331494	0.81888206	-1.41501827	-0.96287715	-0.79463122
IMAGE:280843	C4orf12 Hs.467	-0.506053615	-1.50482386	-0.10245848	0.86642985	0.93293547	1.34502399
IMAGE:35789	NRBP1 Hs.5158	1.055139746	0.31239229	0.6987638	-1.43467114	-0.7886951	-0.70138657
IMAGE:83920	SLC43A1 Hs.59	0.073577702	0.4007482	1.28899731	-1.72153539	0.06097976	-0.66217987
IMAGE:1535451	Hs.599739 **T	-0.177883181	-0.86817182	0.09121239	2.10416427	-0.35948091	-0.28961634
IMAGE:260931	RANGAP1 Hs.18	0.788468371	0.65976213	0.60460232	-1.94929721	-0.57374927	-0.40505217
IMAGE:844552	Hs.102746 Tran	-1.145866056	-0.28857716	-0.65408437	1.30884088	1.20001524	0.46041416
IMAGE:1609611	Hs.129636 Tran	-0.651188328	-1.10935929	-0.41531513	0.97387922	0.95516754	1.23091858
IMAGE:1683035	Hs.536967 Tran	-0.913974134	-1.03303815	0.02172892	1.15738948	1.11203176	0.62393602
IMAGE:155048	CCDC55 Hs.462	-0.895991406	-0.67446062	-0.48889931	1.41400569	0.80489359	0.74790288
IMAGE:913913	TCF12 Hs.5115	-0.891885619	-0.81616476	-0.51748804	1.28448812	1.05435079	0.8700967
IMAGE:2511682	GCNT3 Hs.1947	-0.261790956	1.42408327	0.49859046	-0.05194379	-1.0910412	-1.22369155
IMAGE:210565	SC4MOL Hs.105	-0.887448162	-0.6814032	-0.54025709	1.36574746	1.03380584	0.62904511
IMAGE:324815	PLCB4 Hs.4721	-1.04733314	-0.51118731	-0.76379448	1.54910768	0.69849171	1.0449244
IMAGE:823577	CCDC12 Hs.631	1.050849832	0.11425704	1.01076492	-1.42151812	-1.04506847	-0.54452986
IMAGE:78869	ADRM1 Hs.9010	0.911521319	0.80179333	0.61977396	-2.03402975	-0.8625221	-0.44813757
IMAGE:360079	OS9 Hs.527861	1.197666884	0.43330117	0.51466913	-1.13659368	-1.0050899	-0.94810493
IMAGE:814148	AA465395::A	-0.794943894	-0.77677723	-0.65705888	1.61849806	0.9390322	0.62137502
IMAGE:1020315	VAV2 Hs.36992	1.170904037	0.71247425	0.32379256	-1.04776668	-1.19313909	-0.98890236
IMAGE:50276	C17orf27 Hs.19	0.443132969	1.50011979	-0.3191227	-1.76937333	-0.27577988	-0.47082255
IMAGE:489800	SCARA5 Hs.591	0.643833779	-0.52363386	1.32494154	-0.64646194	-0.56416385	-0.62585101
IMAGE:729956	ZMYM5 Hs.5309	-0.798211486	-1.00754225	-0.49456157	1.06459173	1.16588858	1.09635226
IMAGE:292424	DKFZp547E087	-1.005606026	-0.42077599	-0.62831567	1.09929475	0.77282713	1.05284575
*mitoch. cont. IMA	146407	-0.931236275	-0.81344132	-0.1845975	0.63679576	1.01328655	1.19768096
IMAGE:178805	SRXN1 Hs.5168	-0.677614086	-0.66447749	-0.80666187	0.38896194	1.30466025	1.32784249
IMAGE:200926	ATG16L1 Hs.529	0.02163613	-0.61436289	-0.84203986	2.30455196	-0.33297341	-0.02993858
IMAGE:884599	FAM82C Hs.511	-1.013032777	-0.62540209	-0.13507155	-0.18168748	1.60525657	1.20292265
IMAGE:789253	PSEN2 Hs.2536	0.771067514	0.58204386	1.10050874	-1.36949032	-0.85912646	-1.1766862
IMAGE:1604622	Hs.634940 Tran	-0.743875653	-0.98557898	-0.37053697	1.27281326	0.88724996	0.89728995
IMAGE:377521	TAF5L Hs.27062	0.959128409	0.52921519	1.09008659	-1.59654033	-0.84353079	-1.15505252
IMAGE:289818	ALDH6A1 Hs.29	-0.985416717	-0.72392705	-0.50192766	0.28814468	1.41601898	1.48726157
IMAGE:711552	CBR1 Hs.88778	1.17190796	0.29202474	0.60944293	-1.47853393	-0.96431646	-0.51485231
IMAGE:795888	JARID1A Hs.762	1.29478243	0.68636541	0.2499432	-1.47439254	-1.17603419	-0.63372402
IMAGE:741885	TFE3 Hs.274184	1.205342407	0.2747053	0.66096974	-1.67462919	-0.77251161	-0.59914293
IMAGE:356949	RPH3AL Hs.461	-0.336407987	-1.12301646	-0.86151238	0.69572085	1.28921458	1.28109171

IMAGE:230180	FCHO2 Hs.1657	-0.843590695	-1.19407967	-0.55819253	1.93653789	0.48451216	1.33319616
IMAGE:292833	C1QC Hs.46775	1.071020147	0.76679107	0.17067844	-1.60244799	-0.75589751	-0.61171938
IMAGE:471477	LOC646324 Hs.1	-0.902827209	-1.018319	0.00213347	1.47774734	0.67342951	0.72787564
IMAGE:204608	MGC40579 Hs.1	-0.724869528	-0.80889207	-0.48893422	1.50175191	0.91983577	0.4902225
IMAGE:44409	MRPS25 Hs.555	-1.110970526	-0.7572242	-0.33365951	1.05642393	1.191697	0.97124554
IMAGE:782585	C1orf150 Hs.38	0.821676709	0.26084565	0.98524644	-0.60286298	-0.90658647	-1.34589214
IMAGE:285367	Hs.597217 Tran	0.712941049	0.32123567	1.50445606	-0.72117338	-1.22768896	-1.48297281
IMAGE:1882431	LOC94431 Hs.6	-0.890588362	-0.88832723	-0.37912108	0.73674585	1.5466821	0.8588468
IMAGE:769911	CAPZB Hs.4327	0.287066342	0.86436229	1.02727046	-1.28408808	-1.10022327	-0.62691968
IMAGE:1323649	WDR66 Hs.6206	-0.983668877	-0.55476291	-0.56721502	2.33101328	0.10001047	0.58564271
IMAGE:1574487	Hs.543584 Tran	-0.920958029	-0.61235044	-0.21305	2.37075133	0.26403215	-0.06850828
IMAGE:756828	PEX1 Hs.164682	-0.693919015	-1.23567544	0.06537861	0.80467486	1.01871477	0.98927878
IMAGE:452544	ABCB11 Hs.158	-0.797940757	-0.79532901	-0.39531439	1.64772192	0.51582496	0.72050076
IMAGE:112565	T91080 11517	0.296607304	1.15224102	0.47197497	-0.68553756	-0.91334359	-1.16436005
IMAGE:1558571	Hs.434889 Tran	-0.398826232	-1.4530775	-0.52637762	1.09024945	1.00456442	1.34101375
IMAGE:2568621	C22orf5 Hs.182	0.577731692	1.15536124	0.93171493	-1.6020793	-1.11331584	-1.04888792
IMAGE:287646	RNF14 Hs.4836	-0.99259081	-0.80822174	-0.42309523	0.53178292	1.19243876	1.50586618
IMAGE:1602284	PLA2G12A Hs.3	-0.534452555	-1.20426131	-0.0305012	0.1219999	1.17541087	1.34878654
IMAGE:234985	TOPBP1 Hs.5345	-1.20549424	-0.37460694	-0.42065611	1.83850644	0.45477876	0.60268671
IMAGE:124502	PAXIP1 Hs.4438	-1.235089907	-0.48000731	-0.49347376	1.12466343	1.2328759	0.83194869
IMAGE:491180	ACO1 Hs.64274	-1.093719445	-0.22018792	-0.43703444	0.32445241	1.14462279	1.0480789
IMAGE:151184	CHPT1 Hs.2930	-1.1090235	-0.58045831	-0.41282764	1.06809876	0.99447718	0.98768134
IMAGE:258120	COX7B Hs.5226	0.215550865	1.45798585	-0.53837562	-0.42641538	-0.64136202	-0.76955816
IMAGE:788668	LOC441124 Hs.5	-0.787921137	-0.65129649	-0.94883461	0.78402132	1.20148551	1.35936288
IMAGE:49693	EIF4EBP2 Hs.59	1.561297421	-0.08964587	1.40327856	-1.50297126	-2.01341597	-0.4451883
IMAGE:700862	LOC643338 Hs.6	0.940982227	0.40221928	0.70178276	-1.11971555	-0.95131687	-0.82099829
IMAGE:50882	ECE1 Hs.195080	0.862964499	0.78985141	0.96177364	-1.58490421	-1.43583869	-0.66069802
IMAGE:594684	C1orf106 Hs.51	0.977260737	-0.03308836	1.0738092	-1.14361994	-0.95855378	-0.65634634
IMAGE:1856212	AI240278 219	-0.978917294	-1.04361722	0.06232016	1.17623719	0.8807742	0.89889018
IMAGE:2028332	Hs.560366 Tran	0.665392928	0.78801039	0.47948904	-1.0095467	-0.952729	-0.81719057
IMAGE:26186	Hs.137567 Clor	-1.447298771	-0.46753629	-0.07626238	1.42914293	0.91024922	0.62818842
IMAGE:71116	TFPI Hs.516578	0.361380123	1.11277034	0.56605746	-0.7124959	-1.15109019	-1.05521144
IMAGE:34466	HSMPP8 Hs.269	-1.079934115	-0.94297374	-0.22834982	1.32115254	1.15754563	0.84110088
IMAGE:302591	RHOH Hs.16067	0.377493966	1.39836514	-0.26105661	-0.728257	-0.7716368	-0.83757409
IMAGE:187605	ABI3BP Hs.4770	-0.621012477	-0.81153103	-0.24464307	2.06906785	0.08557322	0.29997804
IMAGE:1836821	CD300LG Hs.14	-0.366857005	-1.03142438	-0.2624067	0.2727672	1.30846374	0.8441995

IMAGE:2149720	KIF1C Hs.43512	1.183947925	-0.13050288	1.04425721	-1.09984972	-1.15077863	-0.63486073
IMAGE:2545711	Hs.592971 Trar	0.468732542	0.58061477	1.24027242	-0.88437118	-1.34756362	-0.8924267
IMAGE:1031552	EPHB6 Hs.38008	0.778980502	-0.46512242	1.25187708	-1.3839371	-0.54880724	-0.10288914
IMAGE:51328	CDC34 Hs.5149	0.799099267	0.56510311	0.73922378	-1.48184715	-0.7437269	-0.74475924
IMAGE:758435	Hs.25766 **CD	0.666312772	0.32334291	0.99107559	-0.78927145	-1.05245318	-0.88160338
IMAGE:52232	Hs.633382 **C	-0.872469064	-1.02736226	-0.27118627	1.81579945	0.80306375	0.56986662
IMAGE:796341	CLCN3 Hs.48118	0.840000302	1.24150568	-0.17682417	-0.64284857	-0.98370487	-1.27467533
IMAGE:1950245	TMEM56 Hs.483	-0.814639528	-0.44498502	-0.94324459	1.82712285	0.83961967	0.40175005
IMAGE:1636232	PLEKHA5 Hs.188	-0.370794019	-1.36142197	-0.10054598	1.47872603	0.59287951	0.65240094
IMAGE:2340213	POU3F1 Hs.1837	0.731837461	-0.23841231	0.7716626	-1.67787643	0.00297881	-0.02981837
IMAGE:73550	C12orf44 Hs.991	1.035035619	0.4562347	0.46789399	-1.57518999	-1.106736	-0.13984698
IMAGE:307130	C6orf160 Hs.292	-1.165958204	-0.79874166	-0.1684446	1.58350717	1.02446108	0.5496373
IMAGE:2304929	PCDHA6 Hs.199	0.008052541	-0.46407287	-0.3970821	2.25793976	-0.53760647	-0.53995017
IMAGE:1635664	MGC15523 Hs.3	0.519233888	0.77869526	0.84760139	-2.0495324	-0.40993555	-0.54692751
IMAGE:2549448	FYN Hs.390567	0.853843003	0.74226289	0.65122473	-0.81448972	-1.58481266	-0.80888738
IMAGE:700778	ATP1A1 Hs.3718	-0.549783593	-0.75393806	-0.29902865	2.14483347	0.28747163	-0.10293681
IMAGE:1909426	GPR111 Hs.1501	-0.233281113	-1.44895457	0.23194244	0.71775689	0.77322636	0.74244222
IMAGE:460332	AA676696 134	0.419375077	0.69878869	0.99224151	-1.78213011	-0.84453375	-0.29088367
IMAGE:1635062	LETMD1 Hs.2887	-0.798357874	-0.52886255	-0.93270569	1.11284262	1.04952435	0.99434579
IMAGE:376947	Hs.547925 Trar	-1.179160221	-0.99079382	-0.10871944	0.83538594	1.54663149	1.00881294
IMAGE:204614	EIF4A1 Hs.1296	1.032477162	0.92924775	-0.46700375	-0.9771045	-0.70457886	-0.67714933
IMAGE:756575	FAM51A1 Hs.592	1.04755125	0.3739499	0.68163361	-1.62664611	-0.80528704	-0.5523606
IMAGE:257422	BST1 Hs.169998	0.71115011	0.48717615	1.0830314	-1.2008691	-0.86490816	-1.08550138
IMAGE:2566856	FSCN1 Hs.11840	0.725541816	0.95734682	0.09392929	-1.5503473	-0.73277778	-0.35861949
IMAGE:120273	USP46 Hs.33147	-0.753261296	-0.88800086	-0.25831795	1.90627321	0.40392703	0.47459044
IMAGE:136508	OAS2 Hs.414332	1.075553328	0.50450255	0.25786769	-1.10806537	-0.98453696	-0.59981611
IMAGE:853151	RPS16 Hs.39760	0.884933074	0.60054468	0.42766647	-1.71703365	-0.8133521	-0.23241397
IMAGE:416808	DLG1 Hs.292549	-0.929727738	-0.51045365	-0.64546658	1.29331465	0.99253832	0.68125233
IMAGE:731339	Hs.593232 Full	0.878185809	0.73484401	0.74275253	-1.33545636	-1.00181598	-1.01071305
IMAGE:144912	Hs.633129 CDN	-1.18316359	-0.92276434	-0.06936497	0.6279428	1.36142535	1.25622997
IMAGE:758365	CTDSP2 Hs.5245	1.002119667	0.29752993	0.81645075	-1.76077666	-0.71817427	-0.4910869
IMAGE:1559108	AA934093::AI	-0.898888682	-0.38434764	-0.39473287	1.81118484	0.53401109	0.07307463
IMAGE:504630	RBM25 Hs.5311	-0.415141727	-0.37876922	-1.21112544	0.47689413	1.13590662	1.09197246
IMAGE:1636130	VISA Hs.570362	0.438657551	0.70846933	0.51963947	-2.04856186	-0.3041884	-0.01748939
IMAGE:1420942	Hs.634840 Trar	-0.459866556	-0.69970684	-1.11459396	1.77132739	0.63003946	0.73123569
IMAGE:2436850	WNT7A Hs.7229	1.560967261	0.16653805	0.16084651	-0.25918972	-1.37837161	-1.15475477

IMAGE:626990	C20orf3 Hs.472	-1.154187843	-0.31344283	-0.12681259	0.47829659	0.96936237	0.9123028
IMAGE:2577230	TNC Hs.143250	0.817331846	1.00241392	-0.41875198	-0.67737113	-0.74898026	-0.77982728
IMAGE:811053	RNF187 Hs.356	0.698761096	0.63357769	1.32307326	-1.46840602	-0.77040041	-1.41354332
IMAGE:433257	LOC653071 Hs.6	-0.342757178	-0.37575184	-0.41791474	1.97384154	-0.14089132	-0.23279327
IMAGE:280518	Hs.595334 Trar	-0.858283574	-1.02232048	-0.21619626	0.55079345	1.28092507	1.25943289
IMAGE:1055760	AA628230 752	-0.879026167	-0.73095179	0.0777156	1.71726154	0.36192774	0.23863316
IMAGE:1470657	DIO2 Hs.202354	1.385686961	0.24431531	-0.22502183	-0.07448888	-1.04090388	-1.04833337
IMAGE:197300	LOC646858 Hs.4	-0.857192514	-0.33504222	-0.84321948	1.6227303	0.55322615	0.66642
IMAGE:192477	KNDC1 Hs.5306	0.748391803	0.65186135	0.32981681	0.67466149	-1.45285786	-1.73445437
IMAGE:1637328	CARHSP1 Hs.63	0.216288217	1.49344438	0.09548861	-1.31606865	-0.90802417	-0.45986684
IMAGE:770289	UBE2R2 Hs.111	1.127551036	0.31442913	0.68272552	-1.62677985	-0.88062092	-0.50897638
IMAGE:66815	PARP14 Hs.583	0.478738308	1.04892386	0.33370725	-1.69985641	-0.55566627	-0.45310465
IMAGE:796159	TTC18 Hs.59136	-0.703478142	-1.1676748	0.04121482	0.68245858	1.15878417	0.91396813
IMAGE:825223	HOP Hs.121443	0.576409596	0.49295076	0.95549819	-0.85966202	-1.06204971	-0.87670154
IMAGE:143450	Hs.590931 **F	0.542385718	0.58998204	1.03010807	-1.6415157	-0.72808621	-0.61658482
IMAGE:486036	SH3KBP1 Hs.44	0.271860887	1.21757154	0.90059542	-1.70663101	-0.71754812	-0.93571378
IMAGE:50649	PKN1 Hs.46604	0.36223342	1.05380631	0.82536417	-1.04255191	-1.20986793	-0.90334498
IMAGE:856646	AA669312::AI	-0.894137541	-0.61695088	-0.70032934	1.25477093	1.03853864	0.84873474
IMAGE:179500	NCF1 Hs.52094	1.018706864	0.31876073	0.49772423	-0.26525682	-0.52773662	-1.83536325
IMAGE:2062341	PHLDA2 Hs.154	1.509720735	0.18163606	-0.12045644	-1.53388459	-0.55185014	-0.30072991
IMAGE:263836	Hs.514802 **T	1.097938371	0.74815933	0.12523965	-1.14690715	-1.05155323	-0.72723574
IMAGE:810117	ANXA11 Hs.530	-0.206437355	1.29789442	0.78078037	-1.67081031	-0.62700604	-0.31534471
IMAGE:429122	MYO5C Hs.4870	-1.00895292	-0.37514577	-0.37127519	2.08097791	0.25938712	0.19987698
IMAGE:2490241	HOXD9 Hs.2366	0.827291358	0.20192841	1.01679528	-0.50056263	-1.53967582	-0.77458531
IMAGE:1492587	RAB37 Hs.5920	0.408878098	0.90597133	0.79043074	-0.70012122	-1.14369856	-1.11649278
IMAGE:1623764	MGC39606 Hs.3	-0.365315379	-1.28132378	-0.44131015	1.84218004	-0.06574671	1.24516309
IMAGE:202769	H53950::H53	-0.468143913	-0.55405094	-0.95495039	2.03103462	0.36300655	0.33293909
IMAGE:85800	SLC43A1 Hs.59	0.318855627	0.51084997	1.08569017	-1.68628527	-0.26689854	-0.64848729
IMAGE:340630	MAP3K8 Hs.432	0.952793375	0.97296746	-0.36140438	-1.01374794	-0.66790643	-0.75523141
IMAGE:1127964	Hs.116530 Trar	-1.193996381	-0.96111328	-0.0026279	0.93771151	1.38101691	0.91722095
IMAGE:450938	LETMD1 Hs.288	-1.061695498	-0.65640076	0.01946314	0.33955281	1.22654267	0.98671999
IMAGE:366540	MAGI1 Hs.4766	0.948903835	0.38919088	0.68014622	-0.93290395	-1.07324495	-0.85117595
IMAGE:1584573	Hs.346736 Trar	-0.752611048	-0.87596262	-0.60351654	1.82516871	0.6747812	0.69730627
IMAGE:448551	AA777753 62	-0.84173467	-0.7184228	-0.41248707	0.58365301	1.39005732	0.88213471
IMAGE:461661	LOC221442 Hs.	-1.366636354	-1.0765179	-0.09998545	1.40255177	1.1544331	1.23272833
IMAGE:936292	Hs.105551 Trar	-0.632643708	-1.17198572	-0.38757956	1.22165831	1.2944745	0.67528577

IMAGE:66336	PPFIBP2 Hs.160	0.787858881	0.84390726	0.46019141	-1.62804198	-0.88432305	-0.51052346
*mitoch. cont. IMAG	146492	-0.93778745	-0.50563522	-0.66786942	0.67800142	1.11369588	1.20827346
IMAGE:1553306	PSMD11 Hs.443	0.334507749	0.31696829	0.46500133	-2.12224446	0.5507693	0.01300944
IMAGE:40449	PTD004 Hs.1573	0.826585063	1.06140022	0.07744895	-0.92591244	-1.01538838	-0.9874883
IMAGE:768997	RNF150 Hs.4808	-0.858621905	-0.98515891	-0.13381797	1.03629076	0.22782478	1.66882814
IMAGE:1553560	FLJ36031 Hs.29	0.780075923	0.8498797	0.26449076	-1.05371753	-0.85152727	-0.87030208
IMAGE:31237	C1orf31 Hs.2319	-0.934607306	-0.57276805	-0.26890722	1.81509759	0.60398793	0.17811153
IMAGE:460435	Hs.117067 Tran	-1.033337435	-0.36482387	-0.74198526	1.78084275	0.66862825	0.57525254
IMAGE:758301	C14orf138 Hs.55	-0.969228752	-0.34635491	-1.14151301	1.09486913	0.87800783	1.42738979
IMAGE:293683	Hs.597181 Tran	-0.606550359	-1.03775598	-0.87902232	1.12025108	1.33390096	1.11108536
IMAGE:878496	POGZ Hs.59147	-0.948173726	-0.94255245	-0.12409453	0.09260569	1.47543981	1.42316193
IMAGE:814838	AUP1 Hs.41148(	0.894872629	0.79655981	0.41641693	-1.89803061	-0.84936058	-0.31027864
IMAGE:470348	Hs.31841 CDNA	-0.816274015	-1.19588682	0.43012136	1.17850089	1.14206483	0.16002384
IMAGE:1627154	C11orf17 Hs.13	0.062236793	1.43874322	0.52038744	-0.77933253	-1.40338302	-0.71923878
IMAGE:193586	H47383::H47	-0.964447787	-0.7490794	-0.3931548	1.12037489	1.00206397	0.93929542
IMAGE:143661	NTN4 Hs.20103	-0.814395242	-0.65762994	-0.61644062	0.94337419	0.92124262	1.11397173
IMAGE:66894	PARL Hs.478469	0.531230987	0.9703954	0.52650879	-1.84568736	-0.73360974	-0.33127847
IMAGE:1325631	Hs.125341 Tran	1.005567054	0.5945789	0.28816503	-0.54571844	-0.99329153	-1.22141525
IMAGE:1618835	Hs.541326 Tran	-0.85423318	-0.42912832	-0.73725482	0.84316045	1.19731513	0.80613519
IMAGE:1685642	Hs.2868 Transc	-0.568063579	-1.25542255	-0.07468322	1.41874119	0.62490484	0.78493718
IMAGE:2017754	DGCR14 Hs.517	1.061653837	0.58480412	0.84675356	-1.53463395	-1.12454319	-0.86895174
IMAGE:785459	SMTN Hs.14909	1.009987377	0.05453059	0.88429173	-1.58397164	-0.90878217	-0.20938781
IMAGE:392350	CCDC73 Hs.632	-0.177961914	-1.5442123	-0.16746453	0.93293166	0.9885996	0.87106072
IMAGE:809567	FLOT1 Hs.17998	0.601419062	0.86174773	0.68125319	-1.48533213	-1.16382623	-0.39715831
IMAGE:1585400	AUH Hs.175905	-0.569688777	-0.91474165	-0.71398361	1.18753217	1.02755311	0.90403987
IMAGE:435948	AKR1B1 Hs.521	0.811354298	0.56589083	1.00345615	-1.20491468	-1.42102926	-0.69424393
IMAGE:257003	KLRK1 Hs.38778	0.023807489	1.27787492	0.3241308	-1.17136502	-0.91184651	-0.27447558
IMAGE:856289	CCNB2 Hs.1946	0.459367074	1.36356618	-0.2028258	-1.00114717	-0.72689561	-0.75282485
IMAGE:280390	NHSL1 Hs.9229(	-0.530076836	-1.31778281	-0.16324863	1.03891118	0.95252048	0.98441859
IMAGE:768638	SLC15A2 Hs.518	0.623881329	0.95937742	0.25888062	-1.23095401	-0.69514177	-0.77239312
IMAGE:299332	GREB1 Hs.4677	-1.181652426	-0.14793513	-0.68900352	1.280055	0.75378145	0.82179928
IMAGE:22040	MMP9 Hs.29741	0.375608855	1.1452813	0.1976623	-1.14212824	-0.65744593	-0.72883894
IMAGE:342814	FLJ16636 Hs.49	-0.746385753	-0.85050856	-1.18690533	1.46942772	1.41107318	0.99847224
IMAGE:783116	Hs.634309 Tran	-0.681108254	-0.76129786	-0.62127447	1.30558925	0.86197623	0.77263678
IMAGE:138304	ITPR2 Hs.51223	-0.422487845	-0.9229646	-0.84670147	0.78759344	1.07953821	1.20942385
IMAGE:1897302	STK17B Hs.882	0.730144206	1.11217566	-0.1317597	-0.67409987	-0.789072	-1.13560832

IMAGE:858204	Hs.600940 Trar	1.113500375	0.24971722	0.83960492	-1.68060632	-0.64272992	-0.7709963
IMAGE:809939	C6orf48 Hs.109	0.7823247	0.41274361	0.94766361	-1.8161708	-0.74389933	-0.41711184
IMAGE:417466	GPR108 Hs.1676	1.070451658	0.21741661	0.78733624	-1.49026733	-1.12369281	-0.30201257
IMAGE:32996	Hs.527484 Trar	0.856646954	0.65527256	0.50633149	-1.637166	-0.74237976	-0.52124787
IMAGE:231438	Hs.234478 CDN	-0.639959228	-1.22202978	-0.07234009	0.83388354	1.19516871	0.85435637
IMAGE:211951	Hs.406106 Trar	0.872977108	1.24461084	-0.06741781	-1.03285762	-0.88743572	-1.17181632
IMAGE:1947258	TRIM56 Hs.5210	0.333386013	0.42461999	0.91728344	-2.07714587	-0.3437004	0.13723297
IMAGE:1083850	EEF1G Hs.44446	0.868998322	0.42759545	0.78229419	-1.62611026	-0.62408632	-0.67256181
IMAGE:1667310	C6orf170 Hs.12	-0.82400915	-1.17002058	-0.33243429	1.31708573	1.12170858	0.96779315
IMAGE:1635288	TXN2 Hs.211929	0.441580596	0.91937961	0.48524154	-1.77174094	-0.88698898	0.0107377
IMAGE:1891757	Hs.603854 Trar	-0.720595136	-1.19049377	-0.1202198	1.12318673	1.1878168	0.70590458
IMAGE:280483	MAGI1 Hs.47663	-1.198271811	-0.49628335	-1.21779457	1.10795758	1.58460359	1.37151479
IMAGE:309603	CNTROB Hs.348	0.353624599	0.75929007	1.13590111	-1.81367057	-0.64354497	-0.63203285
IMAGE:2029216	TINAG Hs.12701	-0.691599215	-1.33515503	0.13215223	1.16112647	0.92038892	0.79342569
IMAGE:2210984	NBR1 Hs.373818	-1.001941575	-0.70666756	-0.62479612	2.00549142	0.84385659	0.49456084
IMAGE:1896918	ADRBK2 Hs.571	-0.693087841	-1.01538213	-0.09485042	1.29833213	0.64918816	0.7337477
IMAGE:266732	TFDP2 Hs.37901	1.276313409	0.69090887	0.24653361	-1.72820886	-1.1396999	-0.39109167
IMAGE:1741841	DKFZp586C072	-0.816986649	-0.96550357	-0.13282341	1.26123837	0.55787997	1.02064625
IMAGE:162838	SH3PX3 Hs.870	0.773706068	0.53345631	0.88688853	-1.52970425	-0.72817048	-0.8114795
IMAGE:664121	LITAF Hs.45994	0.112866319	1.08151052	0.09093025	-1.79417057	-0.05508168	-0.05597582
IMAGE:1574661	MGC52110 Hs.5	-0.96709571	-0.89471418	-0.11092559	0.31328189	1.74798725	0.87010267
IMAGE:399302	MFSD2 Hs.7566	0.876978115	-0.55585508	1.22797986	-1.47511508	-0.40049018	-0.14105411
IMAGE:1013695	RBP2 Hs.97661	-1.154991338	-0.84171326	-0.35814042	1.16537508	1.16411996	1.11323737
IMAGE:1951711	SH2D1A Hs.349	0.000847284	1.40896798	-0.21982129	-0.07912736	-0.98282898	-0.77798994
IMAGE:815214	CHI3L2 Hs.5148	-0.167347899	1.07875446	0.56198974	-1.37197444	-0.19899511	-0.49862746
IMAGE:1636156	NOD27 Hs.5288	0.795142894	0.653838	0.60652157	-1.67621658	-0.36255449	-0.89285223
IMAGE:39947	R53531::R53	-0.759945058	-0.57682082	-0.40418538	1.86449183	0.48608583	0.15980289
IMAGE:529861	PSMB6 Hs.7706	1.075857553	0.67686916	0.38427768	-1.62677726	-0.99760327	-0.48505664
IMAGE:196612	Hs.643717 Trar	0.581890825	0.82945475	0.32607477	-0.95680923	-0.50809961	-1.05970298
IMAGE:293438	APAF1 Hs.55256	-0.783417056	-1.16607866	-0.65736382	1.00721416	1.5874592	1.151275
IMAGE:51405	PAQR8 Hs.23938	0.727048858	1.0686379	0.30358218	-1.09060682	-1.09998478	-0.88241627
IMAGE:123614	C20orf55 Hs.574	1.20020265	-0.66765767	0.87441469	-1.33007455	-0.51175785	-0.05000342
IMAGE:344834	SCARF2 Hs.4742	0.612741874	0.10904017	1.31792426	-0.8691215	-1.03645864	-0.82449826
IMAGE:970649	CENPN Hs.55028	0.263608468	0.76379591	0.81929616	0.06798722	-1.30495879	-1.32825519
IMAGE:32483	R43456::R179	-0.776127445	-1.02149391	-0.50517061	2.23186972	0.51849785	0.57752773
IMAGE:756968	EFNB1 Hs.1447C	0.959487044	0.42064534	0.9895035	-1.72655619	-0.75366593	-0.82685584

IMAGE:290181	N62214::N76	-0.850349221	-0.97278699	-0.29837654	1.69148786	0.94637224	0.4698149
IMAGE:755363	ZNF672 Hs.521	0.816818326	0.64376089	0.60677152	-1.77970368	-0.68983818	-0.47979137
IMAGE:506637	Hs.367827 CDN	-0.743183999	-0.800307	-0.52902467	1.85599919	0.1637735	0.95674464
IMAGE:81050	PPARA Hs.1031	-0.814231703	-0.49302714	0.11210338	-0.41991545	1.24849193	0.99218256
IMAGE:292528	FOSL2 Hs.2209	1.009006535	0.65690344	0.5046811	-1.68431537	-0.36499576	-1.08040522
IMAGE:131979	EPAS1 Hs.4684	0.818357491	0.83182608	0.34903384	-1.69643818	-0.99550626	-0.21962321
IMAGE:302004	SLC25A21 Hs.6	-1.187547296	-0.70887553	-0.46303917	1.13253338	1.04756281	1.24333702
IMAGE:914733	AA569693::A	-0.615533914	-0.56511861	-0.54207656	1.85187089	0.32609321	0.27061039
IMAGE:771130	Hs.181895 Trar	-1.204241396	-0.7245598	-0.35389675	2.03742502	0.77008444	0.52806327
IMAGE:810457	ZNF503 Hs.195	1.222908652	0.10974081	0.57876458	-0.68729415	-0.89375059	-1.14138518
IMAGE:110664	T90522::T830	-0.871243345	-0.42655575	0.18196412	2.02176153	-0.08660378	-0.21591426
IMAGE:141931	Hs.595334 Trar	-0.885673706	-1.10300406	0.01383753	0.80863917	0.90241404	1.25466653
IMAGE:1627058	Hs.165387 Trar	-0.825519669	-0.88115285	-0.27189004	2.36214238	0.38795922	0.14976973
IMAGE:256619	HSD17B7 Hs.49	-0.921970499	-0.71962568	-0.49082038	0.83283579	1.22390608	1.01917786
IMAGE:1762245	FLJ38482 Hs.64	0.3029184	1.18551001	0.48441909	-0.66277206	-1.16990635	-1.00548808
IMAGE:795735	BAT4 Hs.247478	0.370611963	0.55402061	1.44171849	-1.66430702	-0.72066951	-0.80412045
IMAGE:769959	COL4A2 Hs.508	0.859175107	0.57743961	0.60865895	-1.53690914	-0.95440197	-0.42443466
IMAGE:1846982	HNRPA3 Hs.516	0.725943641	0.95567264	0.34229621	-1.45016432	-0.77820699	-0.72192338
IMAGE:1604182	C21orf66 Hs.47	-0.67166463	-0.66901946	-0.45114695	2.08882133	0.593472	-0.10733351
IMAGE:1581839	AKAP11 Hs.105	1.112338386	0.63615927	0.19541673	-0.60121433	-1.11296846	-1.15283461
IMAGE:745007	PLD1 Hs.38286	1.044264676	0.87036271	-0.03238175	-0.67292067	-1.09608213	-1.06246109
IMAGE:624867	FLJ20186 Hs.62	1.070433282	0.38238088	0.57503556	-1.51212225	-0.73364011	-0.65225334
IMAGE:489722	COBLL1 Hs.470	-1.202611821	-0.73243378	0.12076735	0.99403705	0.90802887	0.84954331
IMAGE:1660748	COX18 Hs.3566	-0.923597237	-0.96635334	-0.42626917	1.91086057	0.50499936	0.95190238
IMAGE:842839	METTL9 Hs.279	1.104818493	0.74060398	0.10558527	-1.45046767	-0.82701812	-0.62262951
IMAGE:1592452	PEX19 Hs.5172	-1.277853067	-0.52017508	-0.67753897	0.6392908	1.63374316	1.27093197
IMAGE:772373	DGKH Hs.32647	-0.727525421	-1.16602975	-0.37200443	0.75758647	1.23184866	1.31590316
IMAGE:771579	AA471001::A	-1.271388243	-0.45145293	-0.09865915	1.21558398	0.97290766	0.51909405
IMAGE:589869	WWTR1 Hs.477	0.838961548	0.74381462	0.32707108	-0.36009362	-1.15864819	-1.26426128
IMAGE:264117	CTSD Hs.12157	0.985830244	0.74060585	0.38830014	-1.25838165	-0.90587159	-0.91077608
IMAGE:281082	Hs.322859 Trar	-0.872915614	-0.82237632	-0.62122995	0.89419066	1.27485982	1.15042486
IMAGE:358083	KIAA0226 Hs.47	0.919743918	0.38921322	0.75978606	-2.06862713	-0.51126282	-0.33327832
IMAGE:1879066	C6orf50 Hs.150	-0.666925874	-1.14149427	-0.83716787	1.19140578	1.51448803	1.05319625
IMAGE:430970	IKIP Hs.252543	0.344927397	1.6167765	-0.4221566	-0.62560277	-0.83426552	-0.95499182
IMAGE:2498291	DMXL1 Hs.1810	1.098410345	0.85038847	-0.25067832	-0.53834612	-0.85635901	-1.2151452
IMAGE:786259	PCDHGC3 Hs.36	0.477738421	0.00291082	1.37840347	-1.46434415	-0.75210275	-0.2275313

IMAGE:178324	IL17RE Hs.3908	-0.79291126	-1.22170967	-0.21001342	1.82108406	0.65528838	0.80807573
IMAGE:2403355	MDFIC Hs.42723	0.572351487	1.05291007	0.04613984	0.53721064	-1.36174664	-1.67103114
IMAGE:703479	HDHD1A Hs.185	0.569174476	1.09656811	0.19642626	-1.40719065	-0.76985245	-0.56710362
IMAGE:234425	LOC253981 Hs.2	1.29038344	0.45641888	0.05231084	-0.86980649	-1.15947685	-0.6563087
IMAGE:1127807	PSD3 Hs.434255	-0.842946066	-0.66179797	-0.40345608	1.63541576	0.64586487	0.48015553
IMAGE:325513	W52248 1297	0.431414708	0.58462447	1.17593135	-1.22532115	-1.14768106	-0.62097074
IMAGE:771461	LOC23117 Hs.61	-1.063921594	-0.13864665	-0.89354693	1.49347349	1.02255497	0.40475756
IMAGE:916369	AA573663 724	-0.925110981	-0.4561304	-0.43749541	1.71973764	0.66091247	0.23808122
IMAGE:121543	WWC1 Hs.48404	-0.408410178	-1.21048291	-0.27655325	1.19146531	0.87377892	0.70878696
IMAGE:433673	DUS3L Hs.28429	1.012805135	0.60226792	0.3643966	-1.48619002	-0.76698349	-0.62493182
IMAGE:731476	C4orf22 Hs.5271	-1.144774038	-0.9460707	0.12588151	0.9941628	1.31060658	0.67414585
IMAGE:470079	FADS1 Hs.50354	0.69023203	0.96534456	0.65469666	-1.6932168	-0.6443503	-0.96416861
IMAGE:160672	C8orf1 Hs.43644	-0.78159556	-0.73335987	-0.51662085	0.64827101	1.27533495	0.99460325
IMAGE:1631355	KIAA0100 Hs.59	0.947511231	1.16937382	0.33666878	-1.08634922	-0.98910426	-1.52071007
IMAGE:362985	NR3C1 Hs.12292	-0.982021573	-0.63339731	-0.50890814	1.85720501	0.36661105	0.83544744
IMAGE:322194	KRTAP4-7 Hs.38	-0.893748176	-0.1199636	-0.80996259	2.02329294	0.23203856	0.27768939
IMAGE:341295	ICOS Hs.56247	0.476179142	1.24244827	-0.1642722	-0.55502789	-0.62777496	-1.18979803
IMAGE:1626196	Hs.130639 Tran	-0.860267047	-1.14627445	0.48805354	1.0926868	1.00347973	0.30357877
IMAGE:1542291	BRI3BP Hs.6327	-0.99805902	-0.57003395	-0.31821774	0.40296806	1.23346428	1.11347927
IMAGE:470393	MMP7 Hs.2256 M	-0.784367771	-0.93504365	0.20945862	0.77496899	0.70597529	0.83634958
IMAGE:143443	TBXAS1 Hs.5207	0.779495515	0.89621745	0.34395596	-1.05420605	-0.88412552	-1.00518283
IMAGE:859574	Hs.600897 Tran	0.987609905	-0.20964592	1.28293301	-1.21226452	-0.911454	-0.64689372
IMAGE:320871	NPHP1 Hs.28038	-1.466267373	-0.93501523	-0.01528495	1.21614093	1.29084826	1.1140409
IMAGE:133179	FXR2 Hs.52788	0.450781969	0.64149236	1.25176561	-1.32204448	-1.11139618	-0.76967784
IMAGE:1203584	SLC7A8 Hs.6323	-1.015694391	-0.59060791	-0.31765764	1.56586701	0.65165141	0.58900708
IMAGE:1555047	Hs.127481 Tran	-0.906397036	-0.5305227	-0.18777399	1.79976295	0.49163915	0.09869499
IMAGE:214441	LOC652651 Hs.6	-0.007028511	1.06694687	1.00936351	-1.38651561	-0.63244779	-0.83261852
IMAGE:79229	JOSD1 Hs.3094	1.307944188	0.48514969	0.16314796	-1.11974433	-1.13975825	-0.6340732
IMAGE:1550580	BCKDHB Hs.436	-0.985396487	-0.61308923	-0.7371004	1.51481098	1.01383872	0.79045437
IMAGE:824447	DOCK11 Hs.368	0.467323016	0.86029622	0.74874865	-1.7998483	-0.78214239	-0.34537398
IMAGE:1470128	LOC399959 Hs.4	0.59982664	0.47331496	1.03073309	-0.42702649	-1.38161462	-1.08948765
IMAGE:139957	CYP19A1 Hs.511	0.304610816	1.25329209	0.17058296	-1.16691017	-1.05483798	-0.32833491
IMAGE:950422	MRPL32 Hs.5025	-0.893332556	-0.16336372	-1.17132069	0.62953994	1.30432363	1.1153317
IMAGE:1897944	TSC22D1 Hs.507	1.244782767	0.2429629	0.29104189	0.1954065	-1.34240267	-1.44842469
IMAGE:131446	ANK3 Hs.49972	-0.733536807	-0.97079337	-0.31654042	0.77052602	1.06440063	1.11724414
IMAGE:701460	CHFR Hs.56000	0.674615793	1.17158981	-0.31721746	-1.85175691	-0.44410444	-0.07692523

IMAGE:153149	G3BP Hs.58705	0.617522976	0.48925017	0.78748444	-1.67487239	-0.6887752	-0.28086768
IMAGE:255277	N23708 7439	-1.295485551	-0.40164112	-0.70394948	0.51909132	1.73232164	1.17421389
IMAGE:1523043	HLA-DQB1 Hs.4	-0.865862363	-1.16745679	0.31572677	1.01166625	0.74601526	0.89763876
IMAGE:814119	DHX8 Hs.46310	0.724051588	0.90355024	0.78559398	-1.68860264	-1.18857067	-0.54622192
IMAGE:244313	N52814::N72	-0.889102841	-0.8371134	-0.33801477	1.62307266	0.74039478	0.64837538
IMAGE:1010483	AA228330::A	-1.159355833	-0.49212803	-0.14510131	1.16387679	0.9666777	0.52804794
IMAGE:136218	TIA1 Hs.516075	0.534991122	1.31176696	0.13576447	-0.9027216	-1.11272205	-0.92439907
IMAGE:712950	ACAD8 Hs.1479	-1.021344439	-0.51639467	-0.1746665	0.19503861	1.17764357	1.15225961
IMAGE:235923	TBC1D10B Hs.6	0.301687744	0.31268994	1.4228113	-1.7466457	-0.88022293	-0.07321201
IMAGE:461442	Hs.601138 Tran	-0.054377627	0.32009627	0.61010549	-2.03024752	0.65868308	0.21035461
IMAGE:1032796	LSM1 Hs.42531	0.988947573	0.79346427	0.25858869	-1.35414853	-0.75522097	-0.88748414
IMAGE:783536	AA468491::A	-1.098480341	-0.57751763	-0.253658	1.14846172	0.83378254	0.8488252
IMAGE:827011	RUVBL1 Hs.272	1.242719258	0.52085406	-0.00223666	-1.22051496	-0.77065694	-0.65139225
IMAGE:135766	LOC285500 Hs.	-0.707931369	-0.41422938	-0.422896	2.21265805	0.06749003	-0.06828696
IMAGE:1662723	CAPS2 Hs.4071	-0.674468026	-0.87079194	-0.44887401	1.49152014	0.8077775	0.57968481
IMAGE:1460075	PIN1 Hs.465849	1.006801043	0.84732557	0.15856391	-1.34251699	-0.89311222	-0.74376559
IMAGE:591465	FAM38A Hs.513	0.890323807	0.7740086	0.38370648	-1.59688296	-0.75036676	-0.62888199
IMAGE:814981	ITSN2 Hs.43256	1.022713818	0.80895375	0.29084006	-1.56200169	-0.90545291	-0.64359682
IMAGE:108395	DLG5 Hs.50024	0.543313503	0.84539175	0.51000744	-1.78753	-0.38751351	-0.54552367
IMAGE:2271170	LOC400451 Hs.	-1.227525971	-0.60792618	-0.05298027	0.84970479	0.52419041	1.44550836
IMAGE:462953	MAOB Hs.46732	0.29125972	1.33158985	0.22944044	-1.49825066	-0.6614195	-0.56140474
IMAGE:76886	FBXL15 Hs.380	0.535125843	0.44746401	1.2026081	-2.00299736	-0.78199323	-0.19215431
IMAGE:31448	C6orf152 Hs.21	-1.454713699	-0.08642159	-0.40857644	1.6529548	0.67653116	0.49293753
IMAGE:855486	NFATC1 Hs.534	0.704356335	0.68868295	0.66517288	-1.5500636	-0.96583832	-0.40512312
IMAGE:1950323	MGC13017 Hs.1	-0.864714982	-0.5985898	-0.66826621	1.76823085	0.67106928	0.59098979
IMAGE:773724	FADD Hs.86131	1.025475885	0.80794947	0.22004024	-1.90436978	-0.68739767	-0.43342088
IMAGE:208678	RUFY2 Hs.4998	-0.930159886	-0.81690673	-0.54170156	1.5084637	1.21082025	0.57844294
IMAGE:471826	SETD7 Hs.4807	1.130492268	1.05050961	-0.02273459	-0.95633728	-1.08143836	-1.20530894
IMAGE:2060307	AI376502 836	0.899203504	1.04453439	0.08625889	-0.92129286	-1.13928918	-0.96284841
IMAGE:724229	CCDC107 Hs.53	-0.65332229	-1.31505888	-0.30316671	1.42208309	0.88775296	1.0216941
IMAGE:153505	DPT Hs.80552 D	0.931874439	-0.38654603	0.99551278	-0.49272523	-1.12466189	-0.44499647
IMAGE:2049599	ADCY8 Hs.5918	-1.042750191	-0.48303788	-0.50732343	1.21750221	0.62692191	1.07841227
IMAGE:130421	LOC441127 Hs.	-1.27043344	0.13604376	-0.90221946	1.69667403	0.87859967	0.25408515
IMAGE:897518	RECK Hs.38891	0.023062851	-0.02020664	1.66544908	-0.54361294	-0.74759768	-0.79488505
IMAGE:1631868	RNF135 Hs.298	1.200526142	0.42856889	0.40509104	-1.2492537	-1.00695388	-0.69379876
IMAGE:208969	PVRL1 Hs.33484	0.931485711	0.43435928	0.51779308	-1.65096444	-0.5871834	-0.457861

IMAGE:294682	SLC2A9 Hs.4446	-0.598641994	-0.98973678	-0.65171914	1.16098525	1.1549991	0.88123275
IMAGE:1374473	AA829097::AI	-0.796312429	-0.8726996	-0.33333973	1.09144712	0.8852571	0.94348849
IMAGE:856454	SLC3A2 Hs.5027	0.906592313	0.72932248	0.07548539	-1.73328526	-0.76242375	-0.05251992
IMAGE:341680	LOX Hs.102267	0.53643441	0.93811713	0.46027119	-0.89384299	-0.80855062	-1.0847727
IMAGE:359504	TMEM130 Hs.27	1.178358239	-0.23631551	1.05149856	-1.54720972	-0.91615746	-0.26407012
IMAGE:417487	C6orf108 Hs.10	0.598430636	1.00785165	0.32796463	-1.59073114	-0.88975273	-0.33889536
IMAGE:1590058	SLC35E3 Hs.50	-0.662276957	-0.775137	-0.74838499	1.41073504	0.84733056	0.83353656
IMAGE:1589928	NDUFA11 Hs.40	0.622925316	0.46704793	0.80387864	-1.65881471	-0.79471178	-0.18628166
IMAGE:1220017	OTUB2 Hs.2788	0.816434798	0.66192136	0.60269121	-1.63582222	-0.83750279	-0.49757323
IMAGE:121420	COX18 Hs.3566	-1.163869101	-0.51739924	-0.65317037	0.75841339	1.65525019	0.92470188
IMAGE:399513	ABHD5 Hs.1938	-0.887552641	-0.76161357	-0.04513117	0.18600048	1.1079944	1.2361684
IMAGE:969843	HAPLN3 Hs.447	0.949483482	0.44843501	0.47985254	-1.76400206	-0.69218471	-0.24050664
IMAGE:1556536	SIRT7 Hs.52864	1.1300778	0.36440956	0.37599805	-1.6298458	-0.76070888	-0.32117392
IMAGE:1722146	ST3GAL5 Hs.41	-0.785722977	-0.0101248	-1.1562096	0.49522588	1.21265419	0.9311536
IMAGE:1588477	CALCRL Hs.4708	0.931398138	0.72343617	0.39960968	-1.24421827	-0.85985999	-0.8776853
1276380	LOC161527 Hs.	-0.108429916	-0.76308499	-1.11199845	1.30934066	0.45475038	0.93317939
IMAGE:1882370	VKORC1L1 Hs.4	-1.089771538	-0.40780398	-0.56498919	1.40918214	0.64072114	0.90269648
IMAGE:1953214	ADCK4 Hs.1307	0.42196885	0.78391752	1.29969437	-1.37630201	-1.06589871	-0.9912468
IMAGE:154610	DCTN5 Hs.4359	0.723683695	0.73739878	0.82106082	-2.12802591	-0.70851434	-0.38140949
IMAGE:365326	FBP2 Hs.61255	-0.742686251	-0.5628116	-0.66493056	0.75721911	1.07908471	0.95310618
IMAGE:130819	TMCC1 Hs.4775	-0.533012447	-0.74978372	-0.62771303	2.14351063	0.43515958	0.13016532
IMAGE:1688967	USP49 Hs.5917	-1.167413505	-0.19079038	-0.43015879	1.37515777	0.7687578	0.43108875
IMAGE:857630	AA782345::AI	-0.885824969	-0.49069917	-0.58912142	1.58142774	0.46994401	0.74981623
IMAGE:1507723	ARHGAP22 Hs.4	0.578015791	0.89219315	0.62258514	-0.67997226	-1.15671814	-1.14685443
IMAGE:897974	AA598859 86	0.881845519	0.37970579	0.85536906	-1.60488738	-0.92060787	-0.43604303
IMAGE:80030	RBMS1 Hs.4704	0.782637922	0.45940217	0.42150557	-1.62792055	-0.41745839	-0.34456316
IMAGE:244784	RRAGD Hs.4859	-0.833284488	-0.64922589	-0.35260839	1.4717713	0.08982541	1.10292934
IMAGE:1558642	MLPH Hs.10240	-0.711720021	-0.96196252	-0.22884245	1.0015366	0.73290278	1.06213749
IMAGE:162208	ARPC2 Hs.5293	0.551920835	0.8897235	0.26676275	-1.94365116	-0.52224775	-0.03002102
IMAGE:79592	AKR7A2 Hs.571	0.48600093	0.57129568	1.02380675	-1.75022852	-0.64235878	-0.47311605
IMAGE:200418	IFT122 Hs.4775	-0.406525237	-0.7215	0.25144646	2.0632038	-0.32898348	-0.35649054
IMAGE:161484	DGAT2 Hs.3343	-0.800100012	-0.70747777	-0.19599718	0.45313036	1.1246416	0.92859119
IMAGE:711450	THOC1 Hs.5923	-1.30943754	-0.4400149	-0.22464524	0.78092811	1.01345453	1.11060258
IMAGE:810574	Hs.559734 Tra	-0.42132534	-1.06136844	-0.49292338	1.39086449	0.81349981	0.6358306
IMAGE:182328	CASP3 Hs.1411	-0.869539714	-0.64069905	-0.55774431	1.75640687	0.81172402	0.39440765
IMAGE:51239	MIR16 Hs.5126	0.968843723	0.378421	0.65482631	-1.93270128	-0.79285741	-0.11387128

IMAGE:1155182	POLR2J2 Hs.530	-0.737698081	-0.87701174	-0.30154978	1.38122587	0.67316306	0.74461303
IMAGE:487797	DR1 Hs.348418	1.182407594	0.64593772	-0.24499965	-0.13404406	-1.2626532	-1.03957115
IMAGE:292726	PIM1 Hs.81170	1.027943497	0.71176071	-0.1449785	-0.9242606	-0.81251201	-0.69156059
IMAGE:1460832	Hs.634877 Trar	-0.868617438	-0.47865483	-0.30130197	2.18030256	0.30138486	-0.08415156
IMAGE:22161	LOC401152 Hs.:	0.940438265	0.78711471	0.64893846	-1.65340797	-0.82967084	-0.91942372
IMAGE:877632	FLOT1 Hs.17998	0.450501691	0.97841375	0.62889956	-1.25465424	-1.00344092	-0.67140244
IMAGE:343687	Hs.582517 Trar	-0.749874531	-0.53355905	-0.64920464	1.84237528	0.54448241	0.34979848
IMAGE:1671606	SNRPE Hs.5155	-0.891736897	-0.4825563	-0.7839756	1.18793911	0.91853586	0.93493432
IMAGE:76220	KIAA1961 Hs.59	-0.746357931	-0.66217581	-0.59284935	1.91567189	0.23191147	0.70627893
IMAGE:1744499	Hs.603521 Trar	-0.856491997	-1.2186081	-0.15398675	1.86770746	0.69807244	0.73935368
IMAGE:201890	BIRC3 Hs.12779	0.276484399	1.23511436	0.35280948	-0.89236571	-0.82876148	-0.9872828
IMAGE:292996	YWHAH Hs.2267	0.903154033	0.77529098	0.27625977	-1.38687318	-0.87176775	-0.60435132
IMAGE:1683411	Hs.633996 Trar	-1.154748299	-0.31279008	-0.61762213	0.95511264	1.27165654	0.74656606
IMAGE:415787	ZKSCAN1 Hs.61	0.506968885	1.03226714	0.52813475	-1.63980291	-0.93447455	-0.39474502
IMAGE:154428	Hs.299119 Trar	-0.634577055	-0.99913513	-0.39781031	1.38088028	0.83992314	0.72702774
IMAGE:856420	RAC1 Hs.41381	1.075025226	0.2581282	0.60747253	-1.59156878	-0.69586617	-0.47163585
IMAGE:2062533	ZNF467 Hs.112	-0.637544247	-1.08205584	-0.42495175	0.63390385	1.24269146	1.2339945
IMAGE:1568655	SCUBE3 Hs.129	-1.29498437	-0.51786154	-0.25448099	1.23899831	1.01259976	0.78577203
IMAGE:1699439	CAST1 Hs.4763	-0.757886282	-0.69390117	-0.40724788	1.86705712	0.56724533	0.25243859
IMAGE:1470278	NES Hs.527971	0.560286579	0.60721154	0.91836016	-1.43528782	-0.82345847	-0.6404511
IMAGE:245990	MT1F Hs.51362	0.610569552	0.90064741	0.04924243	-1.66871866	-0.42867996	-0.23097984
IMAGE:191787	ROBO1 Hs.1364	0.158863327	0.82489334	0.83180032	-1.80477405	0.23144827	-0.94205962
IMAGE:251	DICER1 Hs.8788	-0.5335334	-1.14723452	-0.28223543	0.26494564	1.42368202	1.1853185
IMAGE:1522799	FOXJ2 Hs.12084	0.409708181	0.05822193	1.60996865	-1.8316676	-0.57217518	-0.31051319
IMAGE:727164	MGC13114 Hs.2	0.735926545	0.67581118	0.66477155	-1.66577139	-0.8132131	-0.46958655
IMAGE:504544	HCK Hs.126521	0.442579615	0.99314364	0.38064116	-1.07249302	-0.76038128	-0.79651204
IMAGE:173878	MGC14327 Hs.5	0.83285147	0.26660953	1.0021743	-1.90132745	-0.53245131	-0.46813062
IMAGE:1709743	RALGDS Hs.106	1.471458712	0.23282188	-0.03849856	-1.10418967	-0.90744022	-0.4966678
IMAGE:1684602	STYXL1 Hs.1161	-0.904183342	-0.59054537	-0.28126348	1.90458784	0.37770297	0.31138161
IMAGE:838818	AOX1 Hs.40623	-0.839378991	-0.86061979	0.07397526	2.02581481	0.15143779	0.2802765
IMAGE:1607142	DNAJC4 Hs.172	1.349223087	0.01017171	0.39312746	-0.33849916	-1.44467204	-0.74733031
IMAGE:666829	SGCD Hs.59172	0.439632221	1.05135723	0.47576095	-0.35006781	-1.00284818	-1.47826936
IMAGE:1671875	IGHG1 Hs.5106	-0.593254008	-1.2339474	-0.06090552	1.65813959	0.76574522	0.39304919
IMAGE:430395	AA680099 64	-1.078496113	-0.66773088	-0.17639246	1.32631429	0.95941856	0.55409822
IMAGE:951746	ARHGEF11 Hs.5	-0.956992561	-0.63010732	-0.20878856	1.87442387	0.33108286	0.43612879
IMAGE:201317	R99584::R99	-1.162100001	-0.67111952	-0.46895109	1.225798	1.12442497	0.98579519

IMAGE:2310212	SNF8 Hs.127249	0.666667379	0.66666738	0.7946382	-1.8268657	-0.9809569	-0.18547728
IMAGE:26578	PES1 Hs.517543	1.04509849	0.71248374	0.3941239	-0.9522531	-1.32288096	-0.85389416
IMAGE:853996	PACSIN2 Hs.162	-0.920558399	-0.60160746	-0.44017923	1.35001428	0.68607559	0.79738296
IMAGE:129541	BEAN Hs.97805	-0.788271761	-0.90370498	-0.07463797	0.79782338	1.11163072	0.72180847
IMAGE:1566919	AI091219 175	-0.421250412	-1.54098791	-0.34857871	0.83493192	1.43892277	1.10522617
IMAGE:1671363	BLK Hs.146591	0.959299291	0.85504196	0.44218292	-1.04426602	-1.15373622	-1.07623827
IMAGE:486374	WDR25 Hs.4976	0.216730525	1.22315134	0.80894809	-1.4083773	-1.06805581	-0.69457479
IMAGE:376296	TACSTD2 Hs.235	0.624467604	0.39318815	0.98314033	-1.6289905	-0.65079215	-0.4756264
IMAGE:1499828	FUT1 Hs.69747	1.371516351	-0.25219668	0.4422268	-1.44629348	-0.21340563	-0.57206391
IMAGE:219937	HS3ST5 Hs.6320	1.036143619	0.57741716	0.60652256	-1.50187916	-1.07478901	-0.60182621
IMAGE:1553996	Hs.595398 Tran	-0.29686915	-1.1878705	-0.49867715	0.98404048	1.11826184	0.74815359
IMAGE:1880989	TLE1 Hs.197320	-0.462595756	-0.8115023	-0.80143078	0.55768612	1.21053704	1.1447124
IMAGE:153614	IFRD1 Hs.7879	-0.82528374	-0.95174426	-0.55051952	0.80232065	1.38863761	1.16273314
IMAGE:111200	PCMTD1 Hs.308	-1.069727764	-0.45610714	-0.55225407	1.14106526	0.93391945	0.90408524
IMAGE:110321	PTPRS Hs.40845	1.204518972	0.62442957	0.22522826	-0.12469664	-1.4567971	-1.4434644
IMAGE:1637732	PPAN Hs.14468	0.655447753	0.94266534	0.45082869	-1.69978339	-0.54528133	-0.71564078
IMAGE:344243	UCK2 Hs.458360	0.717781541	1.13957069	-0.12875669	-1.19321931	-0.90407175	-0.52779143
IMAGE:309039	IL17D Hs.58562	0.204225758	-1.01747993	1.55355319	0.03796476	-0.4795767	-0.2804483
IMAGE:78353	MT1F Hs.513626	0.652220778	0.88038881	0.21915163	-1.55095102	-0.73016933	-0.29173356
IMAGE:809569	WASPIP Hs.5916	0.692197346	1.15269095	0.23170375	-0.89982872	-1.22739633	-0.92973707
IMAGE:744050	PSCD3 Hs.48747	0.813354717	-0.63965166	1.34777316	-1.26426446	-0.54225975	-0.13874683
IMAGE:267202	ZNF462 Hs.3703	-0.977113308	-1.00760473	-0.31849865	1.06672654	1.30151047	1.00696336
IMAGE:1622066	COPG Hs.51825	-0.123334395	-0.70379446	-0.44325208	2.1548606	-0.08101841	-0.2790838
IMAGE:1700833	Hs.133024 Tran	-0.853111455	-1.83613778	0.20281345	1.5522656	1.20848252	1.01960892
IMAGE:66811	C2orf11 Hs.5916	-0.617789174	-0.82663317	-0.34145291	1.81014964	0.42620796	0.35709206
IMAGE:268240	FXC1 Hs.54943	-1.530430635	-0.74527401	-0.01473697	0.99411709	1.27919591	1.15866517
IMAGE:1882030	LOC441119 Hs.1	-0.952637111	-0.56270309	-0.57861674	1.33023139	0.78871519	0.87733464
IMAGE:234320	PARD3 Hs.13148	-0.701752632	-0.62791717	-0.38514616	1.72492378	0.41360591	0.33740771
IMAGE:232869	LOC642775 Hs.1	-0.886601849	-0.72287455	-0.31864829	1.60201398	0.57793496	0.63257601
IMAGE:40932	KIAA1411 Hs.21	1.267236132	0.32142008	0.28862162	-1.07361092	-0.6041194	-1.06603104
IMAGE:773330	GPNMB Hs.1904	0.726609256	-0.07749325	1.44261901	-1.25223409	-0.56520447	-0.9595092
IMAGE:1667259	Hs.149995 Tran	-0.876419742	-1.06192975	-0.08263219	1.33578707	0.83133004	0.84369737
IMAGE:810551	LRP1 Hs.162757	0.860718855	0.04969114	1.27188849	-0.90065131	-1.13230488	-0.92251941
IMAGE:203352	RORA Hs.56949	-0.744520734	-1.04760053	0.07367347	0.67245306	1.08956663	0.83407036
IMAGE:43933	MAOA Hs.18310	-0.892027168	-0.90902793	0.04301487	0.41543786	1.26016333	0.97221289
IMAGE:248098	NOS1AP Hs.129	-0.936502719	-0.74197481	-0.1330059	1.68665779	0.38442453	0.61289135

IMAGE:756666	PPP1CA Hs.1839	1.035602145	0.59322959	0.16012579	-1.68330385	-0.72272344	-0.23737755
IMAGE:377679	Hs.349024 Tran	-0.555544931	-0.75038252	-0.56370092	1.9531023	0.46463374	0.24578129
IMAGE:859761	TOMM40 Hs.110	0.440181837	1.02784475	0.68861875	-1.75199993	-0.83132195	-0.47949144
IMAGE:744374	TANC2 Hs.41088	-0.262574259	-1.03571903	-0.53152994	-0.00287164	1.42209997	1.19262402
IMAGE:731125	WDR33 Hs.6204	-0.866141957	-1.19351024	0.08591111	0.42722733	1.39737679	1.15748528
IMAGE:586820	PTD004 Hs.1573	-0.292726541	-0.24513075	-0.70291571	2.1670383	-0.20121282	-0.28039491
IMAGE:796757	AP3S1 Hs.40619	1.025464948	0.48722318	0.61489914	-0.99544513	-1.20197976	-0.84023122
IMAGE:1694766	Hs.603142 Tran	-0.663895544	-0.5281353	-0.71500528	1.81033498	0.59761948	0.27384841
*mitoch. cont. IMAG	149977	-0.926057519	-0.70359263	-0.34813774	0.541294	1.12434758	1.21400582
IMAGE:450744	ITGB2 Hs.37595	0.308112509	1.3275976	-0.00059139	-0.58226956	-0.82523096	-1.0453254
IMAGE:813730	MAL2 Hs.201083	1.460581595	-0.93695822	0.63207093	-0.88060367	-0.47648227	-0.21843778
*mitoch. cont. IMAG	144801	-0.88431516	-0.79846171	-0.27160774	0.60077059	1.19637887	1.06652553
IMAGE:1642145	SATB2 Hs.51661	-0.736472268	-1.32049613	0.0165216	1.03431075	0.91084357	1.11964627
IMAGE:1030649	SPAG17 Hs.5288	-0.77000102	-0.85196657	-0.57137286	1.26900508	1.03140557	0.8467568
IMAGE:1901295	Hs.554323 CDN	-0.638240306	-1.20498099	-0.19266488	1.16384848	1.18363555	0.658179
IMAGE:288770	TMEM144 Hs.17	-0.49962669	-1.09623318	-0.72042685	0.99830166	1.30775642	0.98826528
IMAGE:160488	Hs.180284 5.5	0.577177355	1.02325334	0.16858232	-0.52705866	-0.9823729	-1.10194238
IMAGE:825318	Hs.445080 **C	0.558960759	1.19294054	-0.04081748	-1.54672801	-0.57073282	-0.45936927
IMAGE:415988	Hs.586834 MRN	-1.033207489	-0.68551069	-0.35313612	1.90861191	0.46247806	0.64840745
IMAGE:745116	HMOX2 Hs.2842	1.203561002	0.36359443	0.31149524	-1.8519503	-0.3950336	-0.49311831
IMAGE:1456060	FRG1 Hs.203772	-0.44789081	-0.82164543	-0.58380158	1.60050072	0.49192334	0.54163227
IMAGE:954052	SEMA3C Hs.269	-0.890812762	-0.50824718	-0.25438285	1.97135274	0.05410211	0.39111364
IMAGE:44300	H06377::H063	-0.842882258	-0.64439684	-0.44697856	2.01087914	0.23411454	0.54464817
IMAGE:136984	R35849 26248	-1.264242376	-0.82158582	-0.51172624	2.01556601	1.05417131	0.69866277
IMAGE:1915416	CDK7 Hs.18429	0.915472144	0.95004796	-0.09206704	-1.04272902	-0.74191945	-0.89854788
IMAGE:855438	ATP6V1F Hs.780	0.930613075	0.76276073	0.38964896	-1.42099824	-1.1764134	-0.42971027
IMAGE:796239	PDZD8 Hs.5011	0.00728543	-1.53817023	-0.61480938	1.09399508	0.84946095	1.1213829
IMAGE:126459	R06607::R066	-0.295407669	-0.20257601	-0.43871922	2.18003204	-0.54026722	-0.34439027
IMAGE:2010543	DDX28 Hs.4583	0.896176061	0.82659176	0.62416468	-1.70912362	-0.96899964	-0.68623432
IMAGE:1203836	RORA Hs.56949	-0.766456384	-0.46261677	-0.75709727	1.4817394	0.67700289	0.63123903
IMAGE:1623179	AA992653 670	-0.780206117	-0.6846938	-0.49366915	1.63555518	0.57855216	0.60032897
IMAGE:795729	BAD Hs.370254	0.670169456	0.50345674	0.8611313	-1.53441682	-0.85809363	-0.44434297
IMAGE:1117944	FLJ39378 Hs.53	-1.028741618	-1.42583621	0.1387365	1.56197554	1.2851596	0.66131099
IMAGE:462689	Hs.119871 Tran	-0.478495709	-0.44373563	-0.25420578	2.11530569	-0.05338515	-0.36081632
IMAGE:85224	RBM25 Hs.5311	-0.851925637	-0.17435435	-1.00497703	0.4759148	0.96017899	1.35954748
IMAGE:460229	AMID Hs.53365	-0.684618619	-1.34791177	-0.37027534	1.13021943	1.1673623	1.21405804

IMAGE:2565992	DNM1 Hs.52241	0.776390995	-0.36848371	1.41342245	-0.83302028	-0.4475558	-1.09806291
IMAGE:433468	CDC2L6 Hs.5848	-0.563043152	-0.78302219	-0.42475656	1.75635772	0.50587287	0.28781312
IMAGE:2547636	SF1 Hs.502829	0.551233105	0.58060019	0.63597811	-1.95482761	-0.48857128	-0.04932367
IMAGE:340835	Hs.597201 Tran	-0.550659203	-0.99943904	-0.18129479	0.85079543	0.99659717	0.70437325
IMAGE:2282990	CHRNB4 Hs.624	-0.642491759	-0.93082607	-0.59362154	1.51830513	0.59465792	0.98904062
IMAGE:490060	PCGF5 Hs.50051	1.052464843	1.10491736	-0.63876107	-0.85126728	-0.89538622	-0.69096847
IMAGE:246300	TIAL1 Hs.64351	1.095330043	0.75489809	0.36567088	-1.15350673	-0.69392359	-1.38500047
IMAGE:1698127	SYNPO2 Hs.480	-0.501277368	-1.11740909	-0.39571487	1.37508555	0.7793814	0.76820632
IMAGE:205745	LOC646621 Hs.4	0.811325004	0.80605595	0.41719991	-1.80923804	-0.85290509	-0.28542819
IMAGE:136706	SERTAD4 Hs.600	-0.389970698	-1.03516099	-0.59467922	1.42870921	0.64611728	0.80622006
IMAGE:81331	LOC653327 Hs.6	1.308600254	0.14651263	0.04901322	-1.14825324	-0.45653442	-0.63914819
IMAGE:898098	TACC1 Hs.27924	0.860539984	0.65652053	0.68916364	-1.24698097	-1.11776865	-0.77229571
IMAGE:1475363	Hs.642765 CDN	0.968686779	0.35864866	0.76635747	-1.73530719	-0.76068657	-0.45295623
IMAGE:295798	PGRMC1 Hs.900	-0.598030034	-0.30510393	-0.87581614	0.34928969	1.02565278	1.07452865
*mitoch. cont. IMAG	152032	-0.490398301	-0.60958923	-0.88010082	0.67339748	0.94935039	1.12735945
IMAGE:2253659	KIAA0984 Hs.19	0.378844521	1.14232188	0.57033869	-0.35288575	-1.57218826	-1.06959896
IMAGE:485165	PTDSS1 Hs.2925	0.682131209	0.5871982	0.96022908	-1.80483438	-0.72427338	-0.57517271
IMAGE:1917477	ZDHHC5 Hs.272	1.111577517	0.19011736	0.57692238	-1.53508253	-0.58043418	-0.55817858
IMAGE:951015	AMPH Hs.59218	0.553558335	0.9018852	0.44583503	-0.79266048	-1.44359631	-0.5042023
IMAGE:769773	ZNF451 Hs.4856	0.589627632	0.63231161	0.8457315	-1.81419169	-0.54078637	-0.53509517
IMAGE:795830	CENPC1 Hs.4798	0.782378301	0.46369921	0.66551444	-1.59848879	-0.82563628	-0.27688426
IMAGE:769921	UBE2C Hs.93002	0.273281748	1.47102181	-0.5614166	-0.44703615	-1.03177136	-0.43587708
IMAGE:826995	SLC39A8 Hs.288	-0.89966168	0.36278637	-1.10284213	0.39872202	0.68226488	1.10287873
IMAGE:1593948	COL9A2 Hs.4180	-1.071500705	-0.66535764	-0.07289315	0.41549483	0.9575208	1.32338832
IMAGE:756372	KCNH2 Hs.4388	0.441203954	0.65692938	1.05961684	-1.06393032	-0.49667731	-1.41111342
IMAGE:2010404	MNDA Hs.15383	0.646521684	0.80553287	0.50626242	-1.03963036	-0.67696133	-1.09431817
IMAGE:132323	LOC342892 Hs.4	-1.30516288	-0.07554303	-0.66695533	1.18303346	0.99900533	0.72271423
IMAGE:122295	LOC441383 Hs.1	-0.674224267	-1.04016187	-0.60675452	0.90188044	1.08199036	1.34615157
IMAGE:257155	ATF4 Hs.496487	1.20990145	0.22127438	0.32819637	-1.00127264	-0.95443406	-0.60130251
IMAGE:781014	ST5 Hs.117715	0.1594609	0.67180404	1.00375111	-1.89105629	-0.42133201	-0.18919799
IMAGE:1630129	SLC26A5 Hs.585	-0.748256437	-1.16137358	-0.34189005	1.20520798	1.15611821	0.93048141
IMAGE:2461050	LGALS1 Hs.4453	0.509132517	0.42889482	0.86273175	-1.59876712	-0.60686318	-0.27982539
IMAGE:243731	KIAA0182 Hs.46	-0.926672671	-0.67641004	-0.14283121	1.88693254	0.50687352	0.18935702
IMAGE:295741	N72720::W02	-0.655266557	-0.625244	-0.22954081	1.71627731	0.20623208	0.28518248
IMAGE:884539	ENDOG Hs.5919	0.643786241	0.73422079	0.78601512	-1.26232735	-0.73883465	-1.04836744
IMAGE:1870935	Hs.643042 **Tr	1.13894589	0.01118733	0.66716151	-0.90281413	-1.13547492	-0.52086266

IMAGE:757255	C19orf15 Hs.32	-1.148567812	-0.98025539	-0.24136388	1.54639453	1.62564162	0.32290351
IMAGE:878846	BRI3 Hs.567438	0.978402196	0.51096993	0.71054775	-1.47089035	-1.16364555	-0.48770698
IMAGE:953015	LOC113230 Hs.	-0.52862067	-1.28843922	-0.04058661	0.81490546	0.94242339	1.01899425
IMAGE:139759	LOC285419 Hs.	-0.524969694	-1.11754329	-0.18529316	1.95124105	0.09878172	0.64536315
IMAGE:392444	MTX2 Hs.47072	-0.192971459	-0.89938245	-0.52824596	1.6516477	0.50259053	0.14460008
IMAGE:1203805	ASTN2 Hs.6435	-0.842865872	-0.53705816	-0.5180548	1.46856295	0.65019229	0.59869931
IMAGE:1600239	GINS2 Hs.4331	0.531864709	1.16177825	0.15323926	-0.87646619	-0.89170603	-0.96384129
IMAGE:1592048	SSNA1 Hs.5303	0.659547629	0.77464777	0.62363638	-1.68089866	-0.86598966	-0.38395026
IMAGE:856796	SRM Hs.76244 S	1.125858679	0.74962672	-0.17583358	-0.98356023	-0.83646642	-0.77340948
IMAGE:141731	SLC16A5 Hs.59	1.21347991	0.55837341	0.39342695	-1.59909117	-0.78313262	-0.76733988
IMAGE:214334	SCC-112 Hs.331	-0.816152971	-0.4673176	-0.67117102	1.51265265	0.83189647	0.4196205
IMAGE:852605	CECR2 Hs.2318	-0.732310717	-0.29507045	-0.17809933	2.24312533	0.16683466	-0.64626407
IMAGE:810901	TMEM23 Hs.386	0.653643935	0.66051891	0.76020601	-1.26052028	-0.89958422	-0.76139728
IMAGE:2283783	MTMR11 Hs.425	0.998220192	-1.08935741	1.14960573	-0.92230299	-0.35099134	-0.02700701
IMAGE:810958	CPNE1 Hs.2464	0.853818844	0.42378982	0.64286121	-1.71154768	-0.69529985	-0.31314199
IMAGE:703633	PSRC1 Hs.4059	0.74134983	1.19967234	0.35941441	-1.24375954	-1.24375954	-0.87328218
IMAGE:1881014	Hs.194081 Tra	-1.246376654	-0.5843367	-0.23241944	1.38676873	0.90472637	0.74509923
IMAGE:1602122	C16orf72 Hs.22	1.228163798	0.6973455	0.0692932	-0.8514533	-1.1473721	-0.97605504
IMAGE:1554725	AA913303 11	-0.498928203	-0.11590545	-0.94641088	2.06849197	-0.0124426	0.04921472
IMAGE:160723	LAMC1 Hs.4970	0.953206619	0.27634665	0.5281478	0.17693525	-0.94962463	-1.73182527
IMAGE:145388	GOSR2 Hs.4632	0.657780336	1.02232503	0.28881692	-0.99868867	-1.10363336	-0.77885717
IMAGE:144924	TRAF3IP2 Hs.57	0.457066181	0.42371927	1.11542941	-1.81219086	-0.34016309	-0.56311099
IMAGE:811032	PAWR Hs.40607	0.996036028	0.30347936	0.69185428	-1.67098612	-0.67866105	-0.46444377
IMAGE:209655	TGFBR3 Hs.482	0.717520712	0.3763101	0.90428863	-1.49801367	-0.73298356	-0.54010977
IMAGE:613056	RCN1 Hs.97887	1.119406104	0.79380625	0.01708545	-1.33132263	-0.80753156	-0.75232116
IMAGE:1901002	Hs.560776 Tra	-0.238319165	-1.20632189	-0.20586235	1.72765766	0.49481022	0.20182163
IMAGE:51543	FEZ1 Hs.224008	0.642235563	0.41262922	0.944481	-0.71807763	-1.67616227	-0.36865853
IMAGE:342089	LOC643980 Hs.	-0.430997315	-0.22144613	-0.80415962	2.04593797	-0.08785725	0.02578397
IMAGE:2050110	TRPC2 Hs.1319	-0.989157926	-0.88902103	-0.14709765	0.91185004	1.09004821	0.99924225
IMAGE:365755	MYO5A Hs.2121	0.91671454	0.93551165	0.07381249	-0.30534503	-1.26251373	-1.30274614
IMAGE:135688	GATA2 Hs.3677	0.501201468	0.80387185	0.66614294	-1.00201514	-1.07698854	-0.71128497
IMAGE:744055	Hs.553293 Tra	-0.639340147	-0.6157173	-0.67997145	1.93153434	0.42292025	0.37809589
IMAGE:415076	EPB41L4B Hs.5	-0.652489995	-0.86170204	-0.32139853	0.27496308	1.14399771	1.25407543
IMAGE:246808	NALP2 Hs.3692	0.638665344	0.88391554	-0.16788792	-0.78687809	0.03652279	-1.32365613
IMAGE:345601	PXDN Hs.33219	0.518621306	1.09978269	0.14525253	-0.9242763	-0.96539621	-0.71949915
IMAGE:1938312	DHTKD1 Hs.104	-0.786316686	-0.53330547	-0.38559373	1.71783619	0.78641124	-0.04282203

IMAGE:1951140	MNS1 Hs.44448	-1.429178138	-0.32296374	-0.43046028	1.22792384	0.81543712	1.12292722
IMAGE:840697	FKBP9 Hs.57546	0.467051063	0.20838454	1.29829129	-1.43283266	-0.99222273	-0.21096213
IMAGE:161336	ZNF678 Hs.3032	-0.598968473	-0.97854371	-0.40876374	1.51956187	0.79412825	0.56353282
IMAGE:2577105	ARL2 Hs.502836	0.597909748	0.85272112	0.59552089	-1.47382611	-0.60454775	-0.84197355
IMAGE:2562848	PSMC3 Hs.2507	1.051584842	0.56505295	0.08583346	-1.09685278	-0.89159212	-0.5438036
IMAGE:281865	KATNAL2 Hs.404	-0.368056071	-0.61199475	-1.31449628	1.0356383	1.01208199	1.0654763
IMAGE:1690915	LIX1L Hs.63243	-0.253726126	1.34210814	0.52032169	-0.55328604	-0.78345445	-0.94623466
IMAGE:51078	TBX19 Hs.50797	0.946307565	0.51485655	0.4885084	-1.31913962	-0.74606728	-0.73717478
IMAGE:897427	MTG1 Hs.50157	0.470540225	0.80352607	0.57028014	-2.06113922	-0.36010622	-0.20270417
IMAGE:434868	FLJ10120 Hs.59	-1.047630363	-0.8072788	-0.13772803	1.19693093	0.74122678	1.01636607
IMAGE:756763	AA425787::AI	-0.635017557	-0.73558973	-0.62639708	1.49357199	0.76971531	0.57561999
IMAGE:288903	FBXW9 Hs.5151	0.6733475	0.91084762	0.60666477	-0.92452593	-1.02386493	-1.1862328
IMAGE:451169	ARIH1 Hs.26878	-0.714867644	-1.2649431	0.02542083	1.10577931	1.00553191	0.82662887
IMAGE:1584638	ZNF141 Hs.6010	-0.855896363	-0.66654057	-0.22392139	2.17172531	0.12135707	0.27047476
IMAGE:624361	AHR Hs.171189	0.646652219	1.08854832	0.26023404	-0.39214778	-1.30880688	-1.2271387
IMAGE:289520	N62741::N79	-0.825234828	-0.83723551	-0.04039046	2.12783211	0.23443936	0.18192211
IMAGE:296444	CGN Hs.591464	1.310693126	-0.53795285	0.68027596	-0.5417956	-0.96888234	-0.49877744
IMAGE:291370	ZFYVE27 Hs.523	0.654997639	0.54763973	1.2376107	-1.58915369	-0.92460385	-0.83721189
IMAGE:1553989	Hs.150064 MRN	-0.459127284	-0.92479223	-0.4649481	0.81379151	0.99417271	0.84910017
IMAGE:274529	AK3L1 Hs.59260	-0.858576523	-1.0120567	0.06865546	0.07103705	1.42854278	1.22055068
IMAGE:415305	TMPIT Hs.48883	1.302461537	0.51321726	0.01810129	-1.44641255	-0.5155986	-0.78413366
IMAGE:1576307	AGPAT4 Hs.3531	-0.798669876	-0.78371255	-0.31668486	1.18661661	0.88877877	0.69403432
IMAGE:1642467	Hs.538411 Tran	-0.847425486	-0.96560715	-0.36825257	1.15508217	1.33396623	0.69081626
IMAGE:1883883	AI216134 111	-0.85426159	-1.20016807	0.12859105	1.42897503	0.91879573	0.57313492
IMAGE:141153	CPEB4 Hs.12712	-0.68496056	-0.64406716	-0.75559462	0.96646913	1.31379753	0.65776819
IMAGE:1985471	PLB1 Hs.444933	-0.458618695	-0.99039756	-0.34912161	0.85343907	0.99863382	0.7578535
IMAGE:24254	PCSK2 Hs.31518	-0.550843217	-1.46475393	0.3454203	1.18526627	0.71200063	0.69435344
IMAGE:2114195	IVL Hs.516439 I	0.89763794	0.04084742	0.83859118	-1.11661955	-0.91921367	-0.42013381
IMAGE:898265	SFRS5 Hs.63232	-0.446421529	-0.75487952	-0.70649667	0.16928141	1.2714122	1.2443788
IMAGE:1604300	C21orf66 Hs.47	-0.489875926	-0.98981943	-0.26611048	1.49845301	0.56176045	0.49196767
IMAGE:814409	AGPAT1 Hs.4092	-1.026975097	-0.59046976	0.07142502	-0.01571007	1.18924382	1.16335225
IMAGE:788511	RPS6KA1 Hs.14	-0.083168374	1.03574635	0.58541771	-1.88379226	0.04875305	-0.3255999
IMAGE:1934959	CDS1 Hs.44492	-0.795900919	-0.70953239	-0.4262436	2.07671648	0.37093796	0.34330003
IMAGE:1558675	SOX10 Hs.3769	-1.278620445	-0.5952703	0.55228668	0.24772459	0.7366734	1.13607978
IMAGE:2029034	PRPF31 Hs.515	0.892691903	0.87507714	0.12398364	-1.00212818	-0.95985274	-0.84465219
IMAGE:121580	IGFBP7 Hs.4798	-0.993353845	-0.6397293	-0.30444085	1.59694594	0.72105994	0.51216991

IMAGE:149539	DUSP16 Hs.536	-0.94818034	-0.73507456	-0.2660993	0.76141742	1.01566629	1.08042276
IMAGE:592458	CCDC96 Hs.381	0.803382682	0.53101686	0.39669947	-1.80778472	-0.48140048	-0.20828845
IMAGE:842842	CRTAP Hs.5178	0.381097306	1.01679675	0.78401857	-0.90362323	-1.20511305	-0.96812803
IMAGE:2315876	TPSAB1 Hs.405	0.402284723	-0.62193632	1.63286734	-0.44687039	-0.70861576	-0.55612063
IMAGE:180298	R85257 1139	0.807437232	0.46706061	1.07862254	-1.26746519	-0.91592867	-1.07663108
IMAGE:1554906	Hs.527515 Tra	-0.66857953	-1.47528295	-0.11474661	0.71716629	1.19420726	1.44785343
IMAGE:154465	BPHL Hs.10136	1.190378481	0.22705402	0.70417702	-1.21608337	-1.09865193	-0.69163473
IMAGE:810239	TSNARE1 Hs.37	1.115595121	-0.22505413	0.65155548	-1.58599327	-0.20021325	-0.36404931
IMAGE:25321	ARL6IP6 Hs.516	0.590205185	0.4507393	1.2875346	-1.4573283	-0.97791433	-0.73559236
IMAGE:1132291	Hs.116465 Tra	-1.31145527	-0.65371345	-0.16451798	1.17719825	1.29069175	0.68551055
IMAGE:209179	RYBP Hs.64263	-0.609570708	-0.88576463	-0.3887523	1.4955534	0.57462213	0.65876785
IMAGE:2541203	SLC7A5 Hs.513	1.294197749	0.61446447	-0.64284101	-1.12247529	-0.53726113	-0.39970564
IMAGE:1636117	SUSD2 Hs.1318	-0.100788108	0.21740373	1.44557413	-1.12012945	-0.58646912	-0.27529253
IMAGE:293193	C8orf72 Hs.154	-0.873631599	-0.32542941	-0.70485639	1.74174967	0.60917918	0.32873315
IMAGE:813195	SPIRE1 Hs.5152	-0.60869862	-1.22009921	-0.30461579	0.84297341	1.11614475	1.16484832
IMAGE:241897	H93027::H93	-0.682669822	-0.72689096	-0.91451494	1.52165919	0.68967005	1.0461556
IMAGE:432732	GMDS Hs.1444	-0.751499221	-0.63678644	-0.64361458	2.23634927	0.51273122	0.13786624
IMAGE:1836679	GPR156 Hs.333	-0.760884513	-1.22015273	0.18473697	1.23226385	1.37221028	0.13616053
IMAGE:117544	ITGBL1 Hs.6434	-0.539259158	-0.85630675	-0.94923449	0.98841305	1.14245966	1.14901926
IMAGE:1607286	CYR61 Hs.8867	0.685212588	0.97486549	-0.2078296	-0.85149366	-0.76118054	-0.6176559
IMAGE:291871	FCRLM1 Hs.266	0.19679195	1.32630446	0.34070021	-0.64692601	-1.09817323	-0.96542065
IMAGE:241900	RNF149 Hs.142	1.019053264	0.88352337	-0.12140571	-1.59039918	-0.72451374	-0.38719489
IMAGE:46843	SHC2 Hs.30965	0.044376918	0.36031862	1.40032621	-1.65516883	-0.5915247	-0.11075755
IMAGE:1553993	AA933082 77	-0.188008964	-1.01019722	-0.51172076	0.71292268	1.14189046	0.58214708
IMAGE:843374	STOM Hs.25390	0.758847499	0.61438551	0.73798077	-0.86599879	-0.75780278	-1.3585239
IMAGE:292362	SLC22A9 Hs.50	-0.633230107	-1.25299395	-0.18867855	1.30483809	0.61395248	1.1463937
1293021	SFRS16 Hs.466	-0.75038178	-0.69170943	-0.58714855	0.86508586	1.40569183	0.6262948
IMAGE:415696	HRBL Hs.52108	-0.337614751	-1.45160087	0.5405585	0.95112554	0.51471861	0.54228115
IMAGE:294259	CYP39A1 Hs.38	-0.088104688	-1.33295635	0.09156007	0.90766628	0.72557362	0.38390157
IMAGE:111437	SMAP1L Hs.152	-0.532527362	-1.29590793	0.10641172	0.79110134	1.55971055	0.2588264
IMAGE:377256	VPS41 Hs.5921	-0.412592653	-0.40247496	-0.70499406	2.20055521	-0.07289102	-0.0238202
IMAGE:486072	MAP2K1 Hs.145	0.690091532	0.85473949	0.76278056	-1.68114397	-0.97681659	-0.61276166
IMAGE:627125	BID Hs.591054	-0.077260942	1.45884493	0.08791173	-1.32369524	-0.33885317	-0.51971724
IMAGE:773183	GTL3 Hs.53275	1.170042239	0.53357897	0.43602759	-1.61879704	-1.01207505	-0.46959421
IMAGE:1526201	Hs.367445 Tra	-0.62200537	-1.34157278	-0.39114416	1.3909095	1.36979719	0.67359074
IMAGE:586990	SLC11A2 Hs.50	-0.590037648	-0.70612713	-0.67229534	0.44365625	1.34604465	0.99491544

IMAGE:428184	MGC18216 Hs.5	-0.353606667	-0.89487599	-0.73249519	0.48167031	1.13013908	1.17653359
IMAGE:810898	TMEM77 Hs.485	-1.2880826	-0.35684163	-0.82021274	1.08163325	1.46209885	0.94892017
IMAGE:1374241	AA828839::AI	-0.867244612	-0.4259902	-0.60477962	1.56920124	0.56417063	0.56245488
IMAGE:391967	AI003617 582	-0.833747989	-0.67511668	-0.54508476	1.10395639	1.17259494	0.66810161
IMAGE:262894	SSX4 Hs.55840	-0.013185359	0.69386036	-0.11210835	1.28565445	-1.07803426	-1.08849726
IMAGE:586658	VPS41 Hs.5921	0.303276442	1.02116381	0.82749561	-1.49369844	-1.09134379	-0.43598765
IMAGE:753069	MERTK Hs.3061	-0.654929487	-0.98392933	-0.36623213	0.70358282	1.35053016	0.8619654
IMAGE:897563	M-RIP Hs.46234	0.886308451	0.75953443	0.53134119	-1.34613329	-0.98775289	-0.79905463
IMAGE:726088	AA401756::AI	-0.883480268	-0.90092549	-0.22492307	1.19424587	0.72431516	1.03920145
IMAGE:823909	LDLRAP1 Hs.590	0.313457029	0.74625764	0.88296691	-1.84021836	-0.60522351	-0.24783877
IMAGE:505924	ELAC2 Hs.43423	0.928368299	0.56998915	0.46822717	-1.65483747	-0.71906969	-0.45891297
IMAGE:363124	LAMB1 Hs.4896	-0.480099598	-0.52383562	-1.24795208	1.47746786	1.13946444	0.44891063
IMAGE:781459	Hs.469244 CDN	0.763456601	0.17839757	0.97604294	-1.72572393	-0.60026687	-0.30684413
IMAGE:731459	MOV10 Hs.5149	0.192371103	0.58825578	1.07624266	-1.62006867	-0.6855022	-0.21067278
IMAGE:324236	Hs.632945 Full	-0.072699949	-1.33192261	-0.73828907	1.25937774	0.67473865	1.09567879
IMAGE:396214	CAMTA1 Hs.397	-1.10070072	-0.49516597	-0.52171177	1.11566433	0.87994105	1.05033437
IMAGE:1626903	AA994902 29	-0.539927608	-0.38350791	-0.39807711	1.91263365	0.13725405	-0.16713804
IMAGE:530958	SMO Hs.437846	0.322847993	0.51284615	1.40920381	-1.34710076	-1.10270874	-0.56523648
IMAGE:1601603	ELAVL1 Hs.1844	-0.990870546	-0.69136475	-0.57985521	1.64641079	0.71347441	0.88828676
IMAGE:86160	SERPINA1 Hs.52	0.281257749	0.93678838	0.79173479	-1.65674184	-0.69436709	-0.46562874
IMAGE:823616	C3orf1 Hs.4772	0.447601521	0.81471171	0.68407769	-1.78533427	-0.15908382	-0.80414886
IMAGE:1631397	LOC389541 Hs.4	0.96735114	0.08999477	0.8032281	-1.47696782	-0.65561466	-0.45747151
IMAGE:121275	HLA-DQB2 Hs.5	0.755693465	0.62158136	0.50654297	-1.43137698	-0.64011555	-0.62759842
IMAGE:810217	AA464698 81	-1.009143697	-0.9837713	0.46158057	0.39063992	1.14597917	0.8757777
IMAGE:412894	AA707694 23	-0.82020633	-0.70952511	-0.45248425	1.02089983	1.02093859	0.81836405
IMAGE:610341	ATAD3B Hs.234	0.452146053	0.72166785	1.34156799	-1.61306473	-1.11894144	-0.70567468
IMAGE:1456962	ARPC4 Hs.3233	0.494356985	0.62429652	0.74990474	-1.54863281	-0.69938514	-0.36734322
IMAGE:254749	KIAA1632 Hs.51	-1.091927892	-0.8796659	-0.25864794	1.00647688	1.14942884	1.12479489
IMAGE:202802	KCNT2 Hs.4200	-0.860342241	-0.62056855	-0.45386273	1.21816966	0.94379705	0.62672789
IMAGE:50182	KAL1 Hs.52186	0.900294487	0.15614781	0.9249526	-1.56705811	-0.70182248	-0.47197362
IMAGE:240748	DUSP22 Hs.291	0.485222458	0.85053991	0.75632646	-1.61222844	-0.90562759	-0.43119559
IMAGE:31022	KIAA1045 Hs.79	-0.911753854	-0.82785368	-0.25011704	1.27419539	0.72283138	0.92503082
IMAGE:135118	GATA3 Hs.5241	0.362510003	0.23684911	0.87709135	-1.85650424	-0.06583656	-0.07306206
IMAGE:284101	NOPE Hs.59110	0.055227659	0.86831537	1.24407031	-0.60798089	-1.24218931	-1.09023225
IMAGE:2418897	APOBEC3B Hs.2	0.694652368	1.0060619	0.11198875	-1.5358184	-0.52168868	-0.63355026
IMAGE:280787	ATP5J2 Hs.5676	-0.916187532	-0.84819277	-0.30617739	0.76255454	1.43278861	0.83394904

IMAGE:824694	PTP4A1 Hs.2277	1.388775571	0.26391802	-0.05976548	-1.14158001	-0.71688889	-0.54586463
IMAGE:120631	Hs.17529 Clone	-0.900226057	-0.64980103	-0.45857761	1.52478859	0.97779281	0.39568124
IMAGE:854404	AA668953::AI	-0.717060187	-0.60985676	-0.26790129	1.82797795	0.62608478	-0.12881071
IMAGE:305122	EIF1AY Hs.4611	-0.751863524	-0.51005005	-0.50418	0.87843705	0.64999425	0.99466406
IMAGE:810408	ERGIC3 Hs.4725	0.520028117	0.47684435	0.75381612	-1.84335269	-0.3859004	-0.20832575
IMAGE:951242	ROD1 Hs.26998	1.190104394	0.25156659	-0.01231215	-1.53998646	-0.49433293	-0.11279691
IMAGE:489623	EBF Hs.308048	0.513963114	0.00070794	1.08330081	-1.50237862	-0.31938803	-0.30436593
IMAGE:243648	VGLL4 Hs.37395	-0.588309817	-0.37821602	-0.87384195	1.78389349	0.35330128	0.40489641
IMAGE:609743	AA169372 106	-0.214084624	-0.07570786	-0.06247182	1.79997416	-0.16569036	-1.12150531
IMAGE:291129	N72150::W00	-0.951092507	-0.79429594	-0.54111361	1.83628886	0.6113864	0.84679942
IMAGE:1623417	Hs.557807 **C	-0.549230724	-0.69267351	-0.21148817	2.348721	-0.3982898	0.17678536
IMAGE:1468431	GPR175 Hs.6418	0.598030867	-0.02369176	1.16707977	-1.72846384	-0.20848154	-0.38341299
IMAGE:30148	LOC124512 Hs.7	0.895469315	0.57214991	0.7570482	-1.28365293	-1.16240815	-0.70167801
IMAGE:284408	Hs.248158 MRN	-1.159068442	-1.15906844	-0.10331255	1.72419733	0.9644811	0.9176676
IMAGE:2229965	ADAMTS2 Hs.59	0.699708257	-0.02785837	1.2184949	-0.30998815	-0.99038721	-1.23051809
IMAGE:243068	C20orf3 Hs.4723	-1.052899563	-0.29598462	-0.26758129	0.51325669	0.87886504	0.96568117
IMAGE:2461206	HBB Hs.523443	0.366281223	0.51558057	0.89888836	-0.5495939	-0.9449666	-0.95184264
IMAGE:1613757	RP11-50D16.3 H	-1.179521728	-0.59328746	-0.39714553	0.87503761	1.08131537	1.19929273
IMAGE:773617	UBE2D2 Hs.108	0.851619836	0.665068	0.42006326	-2.12170545	-0.36625271	-0.31215268
IMAGE:1015340	GCNT2 Hs.5198	-1.002328045	-0.90865812	0.42492412	0.95051649	0.83863297	0.54617464
IMAGE:249688	CCND2 Hs.3760	-0.019699627	1.51395396	-0.69497078	0.08956192	-0.71568669	-0.74654326
IMAGE:743890	AA634469 135	-0.708658458	-0.32325378	-0.91539348	0.1931885	1.07757695	1.42134475
IMAGE:565149	TMEM163 Hs.36	-0.848516532	-0.8335836	-0.28703837	1.08716449	0.99937131	0.79541237
IMAGE:1253597	AA879036::AI	-0.684577171	-0.84853008	-0.45771988	1.01520346	0.95569285	0.90091442
IMAGE:1574335	Hs.527119 CDN	-1.071102066	0.37031225	-1.19761469	0.59585729	0.99950515	0.95284066
IMAGE:2556648	DDX3X Hs.3807	0.956784166	0.48964035	0.18639729	-1.47730204	-0.53634093	-0.38899042
IMAGE:840691	STAT1 Hs.47094	0.628887452	0.96469188	0.33733019	-1.45645806	-0.65052743	-0.70504626
IMAGE:1868692	C6orf170 Hs.121	-0.85872918	-0.93008777	-0.09857242	1.00917579	0.87447081	0.92279434
IMAGE:2330085	FNTB Hs.632345	0.52488485	1.11565328	0.28542671	-0.28494052	-1.28215763	-1.25049244
IMAGE:780938	Hs.537965 Tran	-0.635750281	-0.62083356	-0.2967694	1.69882252	0.50289755	0.05411745
IMAGE:1455835	GAL3ST1 Hs.179	1.160603074	-0.18124391	0.91672289	-1.16207738	-1.15448775	-0.29837723
IMAGE:453107	CDC45L Hs.4742	0.22997246	1.49682263	-0.33716851	-0.70486883	-0.70486883	-0.75899432
IMAGE:1502795	Hs.635653 Tran	-1.126132362	-0.73781086	0.29071637	1.04295809	0.62183917	0.76772211
IMAGE:586958	SDF2L1 Hs.3031	0.509482878	1.14684668	0.28549503	-1.38143895	-0.97170507	-0.4882191
IMAGE:212394	DCTD Hs.18385	0.760990613	0.63766718	0.65231184	-1.38888362	-0.7067509	-0.81774198
IMAGE:1504457	CPEB3 Hs.13168	-0.777209025	-0.92401051	-0.10296151	0.96406929	1.11448398	0.60197791

IMAGE:951297	AA620525 283	-0.930284302	-0.59760491	-0.46124744	1.48431091	0.64337134	0.74071087
IMAGE:785733	Hs.608192 Trar	-0.670404294	-0.89902517	-0.47581071	0.21264385	1.14252383	1.59373992
IMAGE:788209	UBTD1 Hs.5007	1.316534208	-0.72011412	0.84045674	-0.96000611	-0.63212823	-0.35306671
IMAGE:269748	TM2D2 Hs.7471	1.229419191	0.44994485	0.37686913	-1.09852966	-1.02545394	-0.86614887
IMAGE:813499	FAM89B Hs.2572	1.274345941	-0.16820973	0.76848975	-0.97451821	-1.13496245	-0.51033585
IMAGE:49404	TATDN2 Hs.4754	0.83973671	0.36572679	0.66523394	-1.72893564	-0.67814373	-0.2326583
IMAGE:1155867	SUZ12P Hs.6288	-0.65123226	-0.51593919	-0.6194678	1.61727952	0.4212594	0.48655301
IMAGE:1870871	Hs.105081 Trar	-0.798425559	-1.01289207	-0.05583525	0.82414767	1.42063267	0.54199016
IMAGE:202682	C8A Hs.93210 C	1.154767322	0.29213269	0.59976007	-1.14604812	-0.6779319	-1.09607008
IMAGE:950470	CLDN12 Hs.2585	-1.071759525	-0.88186323	0.08261006	0.52561813	1.23772923	1.0638242
IMAGE:286661	Hs.90250 CDNA	-1.047620736	-1.05848441	0.16080332	0.90790462	1.2087725	0.84147645
IMAGE:461440	C17orf71 Hs.729	-1.091818079	-0.54085611	-0.44768221	1.19022932	1.1617067	0.65667803
IMAGE:25838	APBA2BP Hs.516	-0.778360286	-0.55631456	-0.61392643	0.58287003	1.12748218	1.05906809
IMAGE:396106	AA757720 153	-1.075270745	-0.45967294	-0.51567777	1.1364647	1.24232748	0.56822057
IMAGE:1861822	AI053806::AI	-1.116962838	-0.71536793	-0.06863949	1.4407148	0.80368488	0.58989583
IMAGE:914479	AA570804::AI	-0.921355402	-0.68709524	-0.28622702	1.0924838	0.88256755	0.7954084
IMAGE:51906	THAP8 Hs.35020	0.657702585	0.65097096	1.08314103	-0.59000337	-1.70072084	-1.02621241
IMAGE:1416092	Hs.596392 Trar	-1.276260238	0.07943127	-0.49154521	0.31936115	1.02709361	1.06322021
IMAGE:297403	GPC6 Hs.444329	-0.899601804	-0.7406085	-0.21293732	1.05554937	0.33249913	1.33843861
IMAGE:460002	GALNT5 Hs.2690	0.572640459	0.96434069	0.18649985	-0.48709818	-0.91923198	-1.13226636
IMAGE:454538	TTC9C Hs.31704	0.227595947	1.38497897	0.25808311	-1.53303768	-0.87624634	-0.33218223
IMAGE:127199	BMP2K Hs.1465	1.251162478	0.75691958	0.23834064	-0.94339546	-1.16241984	-1.20423359
IMAGE:1010486	AKAP12 Hs.3712	-1.127341988	-0.36205787	-0.2076521	1.4863464	0.32632447	0.68099404
IMAGE:1506051	ARHGAP28 Hs.1	-1.190428911	-0.92092321	-0.34713688	1.85989127	0.7593579	0.98170011
IMAGE:1700537	Hs.454670 Trar	-1.286933104	-0.67367266	-0.0982395	1.03648337	0.92788687	1.09933779
IMAGE:280266	ETV6 Hs.504765	-0.328241615	-1.07841704	-0.36112602	1.88708808	0.50748464	0.16682279
IMAGE:795263	RPP21 Hs.18323	0.803458108	0.43546537	0.76538989	-1.57517083	-0.82649594	-0.41345581
IMAGE:1630663	Hs.543284 Trar	-0.19735235	1.12033401	0.70870895	-1.71943651	-0.26039726	-0.29052491
IMAGE:1031765	Hs.176247 Trar	-0.634855721	-0.53096559	-0.42160755	1.95165711	0.20144033	0.12264396
IMAGE:1588602	TATDN3 Hs.5305	-1.018459834	-0.28563866	-0.83353161	0.64810531	1.49320434	0.8567526
IMAGE:839583	PRPF6 Hs.31334	0.659926416	0.4658121	0.87300074	-1.63716825	-0.91262528	-0.23006516
IMAGE:869119	AA680221::AI	-0.879345477	-0.43621673	-0.29119278	1.68980519	0.28936785	0.35816126
IMAGE:25930	SLC30A10 Hs.28	-1.12652392	-0.08654883	-0.75903865	0.91499239	1.08147062	0.77194444
IMAGE:212686	MAGI2 Hs.58323	-0.421347878	-0.6975858	-0.73507523	1.82783307	0.5504485	0.21896299
IMAGE:46694	LIFR Hs.133421	-0.94794376	-0.49280431	-0.81874634	0.81103082	1.30094904	1.07257518
IMAGE:594500	Hs.626848 Trar	-0.253787296	-1.18340233	-0.49500931	0.0552073	1.17088282	1.54845596

IMAGE:841149	TGFBR2 Hs.8202	0.851455266	0.59917996	0.5487249	-1.11301257	-0.8786488	-0.87019758
IMAGE:1602248	SLC29A1 Hs.254	-0.995507493	-0.86207432	0.11948208	0.82975916	0.84385973	0.96340123
IMAGE:271568	Hs.634689 Tran	0.739180341	1.04523853	-0.09464159	-1.32801875	-0.66178233	-0.56852523
IMAGE:1486056	DFNA5 Hs.5207(	0.997692193	0.72827902	0.03350997	-0.72195628	-0.8607833	-1.0481047
IMAGE:241537	PLGLB2 Hs.5285	-0.759015346	-0.25789335	-0.78136268	1.60929398	0.62634995	0.26642247
IMAGE:815294	PTPRCAP Hs.155	0.745514985	1.24317751	-0.11909071	-0.53098939	-1.31895505	-0.98423092
IMAGE:308467	NHSL1 Hs.9229(	-0.518564528	-1.25304392	0.10668459	0.68918325	1.1185712	0.71630248
IMAGE:731376	RALGPS2 Hs.49(	1.074106013	0.4806747	0.26737239	-1.32198997	-0.74451298	-0.5998836
IMAGE:142326	LOC402110 Hs.1	-0.424761695	-0.03421025	-0.90499533	1.78108254	0.11087239	-0.07225285
IMAGE:340904	LOC643774 Hs.5	-0.709558362	-0.68757157	-0.66045453	0.75164706	0.98580637	1.18380963
IMAGE:814158	ATF5 Hs.9754 A	0.364569396	1.1881515	0.286133	-1.09309963	-0.83639871	-0.75724926
IMAGE:360254	CYR61 Hs.8867	0.623580812	0.97404878	-0.16777889	-0.96315911	-0.61269114	-0.61087053
IMAGE:49502	NCF1 Hs.520941	0.307127209	0.54874045	0.88699899	-2.18473032	-0.24003839	0.03221849
IMAGE:788256	KIF23 Hs.27084	0.03296102	1.45177278	-0.29907019	-0.98306178	-0.03414	-0.83606118
IMAGE:1680549	MXI1 Hs.501023	0.850722812	0.00896755	0.94272862	-1.44842232	-0.85173572	-0.16778829
IMAGE:2164126	PRKCQ Hs.4985	0.101939788	1.33803264	0.02444523	-0.50478261	-0.77082909	-0.91490348
IMAGE:300000	MAK Hs.446125	-0.802777951	-0.71054239	-0.73877361	1.17837559	1.14206739	0.87300455
IMAGE:245324	LOC90624 Hs.11	-1.055748244	0.02720726	-0.85959298	0.99377203	0.84266196	0.78086873
IMAGE:1876207	PRTN3 Hs.928 P	0.675354203	0.77754817	0.39132546	-0.35332496	-0.78931688	-1.52586855
IMAGE:745493	AA625990 114	-1.003076265	-0.39105024	-0.20838401	1.83653189	0.37826219	0.1368757
IMAGE:121600	PACSIN2 Hs.162	-0.674024052	-0.59036475	-0.69075591	1.95052516	0.49164136	0.31786157
IMAGE:814210	C20orf4 Hs.1131	0.928059397	0.59066506	0.59284921	-1.64731702	-0.66962064	-0.70221052
IMAGE:1188706	HDAC11 Hs.404	-0.527772634	-1.27679804	0.10987336	0.68339518	1.04810677	0.83801235
IMAGE:545366	GALNACT-2 Hs.1	0.738662587	0.74461114	0.76146535	-1.18222252	-0.97513872	-1.01938104
IMAGE:2276858	MAGEA11 Hs.61	-1.068608419	-0.06577544	-0.75321362	1.01298768	1.17302949	0.45707564
IMAGE:1881297	Hs.604377 Tran	-0.32387189	-0.14628542	-0.06002913	2.26172744	-0.89005935	-0.59139571
IMAGE:1875921	KCNH2 Hs.4388	0.675001122	-0.28512724	1.16389735	-0.06817242	-1.03494856	-0.93656152
IMAGE:344942	PPFIA3 Hs.4137	1.072622552	0.2796515	0.59019723	-0.44419407	-1.34718466	-0.97477889
IMAGE:744911	Hs.600685 Tran	-0.788956957	-0.53385133	-1.2774571	1.42541088	1.04989717	1.10572575
IMAGE:1659193	RYK Hs.245869	-0.879360651	-0.75898799	-0.53048395	0.97193011	1.55135107	0.59734671
IMAGE:1573305	SPG20 Hs.44041	-0.73946161	-0.59057069	-0.59503742	0.38568845	1.54285005	0.81030673
IMAGE:741735	TMPRSS13 Hs.2	1.444800342	-0.60712582	0.64236758	-0.72507601	-0.6388216	-0.69557364
IMAGE:362680	AA018569::AA	1.250458049	0.45209762	0.23348747	-1.20072034	-1.09623046	-0.54874204
IMAGE:256680	CEP70 Hs.53196	-0.723186186	-1.05290477	-0.30535118	0.61513234	1.21999935	1.21069371
IMAGE:949990	LOC653198 Hs.5	-0.763399806	-0.83261206	-0.02464491	1.83574117	0.65790571	-0.06882294
IMAGE:1469292	PIM2 Hs.496096	0.324260768	1.31761515	-0.19782781	-0.68165492	-0.85491441	-0.67895978

IMAGE:755821	NFE2L1 Hs.5142	0.37523792	0.64997215	0.97157281	-1.53315494	-0.75743475	-0.46169143
IMAGE:741710	SPON1 Hs.4458	-0.879661476	-0.72576505	-0.24680934	1.45890734	0.75933548	0.49840864
IMAGE:190021	H30547 9197	0.989837365	0.57224266	0.46339283	-1.41755888	-0.89155823	-0.61324396
IMAGE:50536	FLJ12529 Hs.44	-1.129214327	-0.64966482	0.35996737	-0.26886126	1.2942295	1.19299127
IMAGE:971367	RPS8 Hs.512675	0.810333335	0.49772085	0.66135395	-1.59470686	-0.50789002	-0.68617683
*mitoch. cont. IMAC	149323	-0.800267069	-0.71024883	-0.51533978	0.9758971	0.64648254	1.28756894
IMAGE:726767	Hs.43818 CDNA	-1.01255825	-0.74855144	-0.15282841	1.8221121	0.82367273	0.18691521
IMAGE:1470659	COG8 Hs.13084	1.010656745	0.54166374	0.10475974	-1.48796517	-1.00032216	0.02885693
IMAGE:869450	RPL11 Hs.38866	0.435346118	0.63591599	0.83314302	-1.71597464	-0.69432187	-0.23802543
IMAGE:1566793	EBI2 Hs.784 Eps	0.554655878	0.9635376	0.16082751	-0.77021753	-0.71338602	-0.99472106
IMAGE:2306860	MAD2L2 Hs.194	0.635559071	1.16372811	0.10644688	-1.24533575	-1.04019867	-0.54645494
IMAGE:154289	Hs.538136 Tran	-0.605084181	-0.71307368	-0.39478884	2.13493625	-0.0614423	0.39722889
IMAGE:223047	H86481 6755	-0.608675176	-0.83029258	-0.51190224	1.45415195	0.68095344	0.66322405
IMAGE:32683	Hs.100912 CDN	0.215230882	1.33718995	0.06397816	-0.72858609	-0.97311132	-0.70690654
IMAGE:267495	GNPNAT1 Hs.47	-1.279711506	0.25089163	-0.4870691	1.47994708	0.53922861	0.1328905
IMAGE:1202753	QKI Hs.510324	-1.033940181	-0.33238669	-0.50820981	1.41769869	0.70165344	0.56540043
IMAGE:40150	THNSL1 Hs.534	-0.921108952	-0.58279989	-0.34922761	1.78142432	0.42686397	0.48410948
IMAGE:292496	USP32 Hs.1328	-1.222587831	-0.58621886	-0.50756651	1.64929529	0.73764311	0.96072977
IMAGE:824487	DDA1 Hs.64260	0.878070893	0.76815482	0.26392096	-1.72861603	-0.42736738	-0.64325636
IMAGE:2466969	CTDSPL Hs.475	0.441909065	-0.00632805	0.83372104	-1.76987744	-0.02966275	0.10401738
IMAGE:713109	C6orf129 Hs.28	0.677915417	1.09940833	0.23334364	-1.74445417	-0.86988674	-0.34332426
IMAGE:588681	EIF3S5 Hs.5160	0.949752981	0.7157388	0.66640608	-1.40050835	-1.25092901	-0.67980792
IMAGE:1895373	AI299363 186	-0.932266795	-0.57469685	-0.3336107	1.46752626	0.65432483	0.55560775
IMAGE:1557007	PWCR1 Hs.5559	-0.558047134	-0.20290385	-0.5914586	2.1811356	-0.12684953	-0.17353634
IMAGE:813546	FAM116A Hs.91	1.094783578	-0.20490316	0.76032291	-0.53996002	-1.10022635	-0.6450379
IMAGE:815514	AA457023::A	-1.012650721	-0.35645403	-0.63548542	1.84357838	0.84450414	0.15993138
IMAGE:1894405	PPP1R1A Hs.505	-0.485734442	-0.91723651	-0.56858284	1.21317551	0.85071377	0.7512957
IMAGE:267634	RAF1 Hs.159130	1.070668255	0.56243112	0.20163861	-1.86342382	-0.61517108	-0.22310243
IMAGE:1612075	Hs.634961 Tran	-1.019649071	-0.68921319	-0.34481522	1.26501256	0.79030185	0.938998
IMAGE:566474	PSMD11 Hs.443	1.006938615	0.27019348	-0.04408894	-1.9626572	-0.09514817	0.19721841
IMAGE:813815	TOMM40L Hs.32	0.891659756	0.73166592	0.66833227	-1.52423288	-0.80596774	-0.94020323
IMAGE:284479	Hs.595184 **M	0.92310858	0.36061825	1.0707339	-1.93741593	-0.7813961	-0.54519559
IMAGE:770588	LOC126208 Hs.	0.5787924	0.89203693	0.36644311	-1.9382441	-0.42481545	-0.30123833
IMAGE:320425	MGC75360 Hs.5	-0.737637334	-0.75467013	-0.63969878	1.0344117	1.08054218	0.9231308
IMAGE:1422194	LILRB3 Hs.6315	0.526501774	0.87733317	0.25322259	-0.0177485	-1.12563711	-1.27889503
IMAGE:301191	ATOH8 Hs.1355	-0.496123362	-1.32766467	0.60579404	0.25973575	0.83040135	0.8883024

IMAGE:813280	ADSL Hs.75527	0.702906441	1.04061624	0.02254476	-1.44834614	-0.64669722	-0.54842161
IMAGE:1626800	MTM1 Hs.43428	0.117768015	-1.08819851	-1.34673251	0.87978741	1.58305675	0.67621722
IMAGE:132899	TRDN Hs.14474	-0.898262396	-1.00837031	-0.09936832	0.65618168	1.71990276	0.60807501
IMAGE:2449452	AI924452 219	-0.62990046	-1.1966925	-0.1147235	0.74185557	0.89980503	1.24163322
IMAGE:154254	Hs.101120 Tran	-1.244170155	-0.94961531	0.50415882	1.61593089	0.6684019	0.37614688
IMAGE:810939	ATG9A Hs.32336	0.499708823	0.42687321	1.01502079	-1.76206461	-0.51930694	-0.37727749
IMAGE:2116508	WASPIP Hs.5916	0.915960258	0.58510742	0.04039899	0.40361786	-1.29539935	-1.41031877
IMAGE:726893	PPP1R9A Hs.218	-1.028199155	-0.43114485	-0.4321462	1.76828235	0.64680729	0.31410911
IMAGE:1558451	AA976113::AI	-0.729240815	-0.89993693	-0.31313247	1.51126844	0.81602231	0.50789144
IMAGE:51608	ERO1LB Hs.5585	-0.688763497	-0.9422076	-0.820722	1.5295124	0.9753798	0.96746694
IMAGE:123735	HSDL2 Hs.59486	-0.822110421	-0.79184904	-0.51138762	1.36626954	0.98144117	0.71246301
IMAGE:260696	KRR1 Hs.205558	0.573759784	1.29565423	-0.26718392	-0.59710235	-0.83140142	-1.04163735
IMAGE:548503	TRSPAP1 Hs.533	1.125190811	0.47334458	0.63994135	-1.24164667	-1.40710095	-0.54898215
IMAGE:754649	AHSA1 Hs.2040	1.11424906	0.44781088	0.29560499	-1.47991531	-0.78982932	-0.44285151
IMAGE:191836	CXorf52 Hs.391	-1.086681646	-0.75055352	-0.19067443	1.04873532	1.18418994	0.76127052
IMAGE:489384	IGFBP5 Hs.3699	0.257353674	-0.27681672	1.56519832	-1.0553788	-0.52068471	-0.35123981
IMAGE:588070	Hs.549823 Tran	-1.007584126	-0.46893798	-0.26165604	2.27306497	0.36341735	-0.09462912
IMAGE:196115	CXCR6 Hs.3452	0.735155373	0.62991143	0.54186226	-1.07292506	-0.74065383	-0.91134913
IMAGE:925325	Hs.377419 Tran	-0.53169406	-1.04537025	-0.45590577	0.9483881	0.87733658	1.109754
IMAGE:307544	TSEN2 Hs.33555	-1.000235579	-0.74393883	-0.52817273	1.36097274	0.88712177	1.028383
IMAGE:136605	POMP Hs.26874	-0.987110899	-0.9233896	-0.23126039	0.7869481	1.30808699	1.05979114
IMAGE:196550	DECR1 Hs.4922	-0.767079591	-0.4672858	-0.95152125	1.09400071	1.04606414	0.90088481
IMAGE:431869	DOCK6 Hs.5910	1.104008159	0.12709621	0.78228072	-1.42649542	-0.93619622	-0.46181581
IMAGE:41128	Hs.594857 Tran	0.965297426	0.85630999	0.40098469	-1.44453593	-1.05208024	-0.73702581
IMAGE:435075	PPP2R1B Hs.584	-0.826974392	-1.14007026	-0.12162257	1.30503124	0.99607061	0.80149334
IMAGE:490306	TM9SF1 Hs.9158	0.67150844	0.85226721	0.37024382	-1.34111641	-0.90516879	-0.50218306
IMAGE:856887	Hs.634574 Tran	0.63323544	0.11608921	1.35410594	-0.65199047	-1.38755263	-0.7770763
IMAGE:841348	EIF3S12 Hs.314	0.218312244	0.81891891	0.79559437	-1.86738671	-0.92663065	0.24367767
IMAGE:2392841	NCLN Hs.73797	0.443663294	1.18343535	0.15175324	-0.42946767	-0.94605849	-1.25481336
IMAGE:279232	Hs.44439 CDNA	-1.048764164	-0.981958	-0.04889849	0.81699373	1.22073972	1.0694729
IMAGE:194986	RBBP6 Hs.18855	-0.309269013	-0.74597023	-0.93427259	0.38845133	1.36802425	0.99422403
IMAGE:260273	Hs.543038 Tran	-0.86068254	-0.41869277	-0.29240998	2.09196697	0.03592528	0.1566832
IMAGE:126763	Hs.245931 Tran	-0.639519314	-1.33962046	-0.1372965	0.98012205	1.04725287	1.11295536
IMAGE:1604331	RAB2 Hs.369017	-0.483326175	-1.31650393	-0.08982535	1.18629178	0.9378442	0.68789087
IMAGE:490600	FOSL2 Hs.22097	1.141954073	0.14912858	0.03036955	0.52987003	-1.51516047	-0.98929548
IMAGE:305606	EPHA1 Hs.89839	1.070350074	-0.13504626	0.7647106	-1.51348847	-0.44001445	-0.40534106

IMAGE:293078	Hs.173030 **C	0.595430745	0.7541362	0.40768556	-0.9449408	-0.80058637	-0.78843021
IMAGE:246035	BLVRB Hs.5157	-1.016371776	-0.53320519	-0.02574531	0.27022797	1.23791078	0.84840833
IMAGE:782439	ATP5I Hs.85539	0.757625119	0.36744448	1.0245313	-1.7296351	-0.67784381	-0.56078961
IMAGE:434990	TMEM23 Hs.386	-0.466886912	-0.57427572	-0.22417075	2.08272417	0.0331695	-0.27393629
IMAGE:489169	C10orf83 Hs.21	-1.551545777	-0.4128573	0.05413292	0.93789592	0.8707261	1.07031644
IMAGE:194464	ATAD2 Hs.3708	-0.689378101	-0.69799814	-0.55217588	1.43266415	0.51428408	0.82433597
IMAGE:126449	R06706::R06	-1.336206177	-0.02309188	-0.64628496	1.47084374	0.8184571	0.55750244
IMAGE:1584623	CCNC Hs.43064	-0.677193589	-0.62821542	-0.77106842	0.58868806	1.28050473	1.05275624
IMAGE:1017145	NSMCE1 Hs.284	0.392097646	0.78560259	0.89191443	-1.61875797	-0.91060022	-0.3520852
IMAGE:344036	BNC2 Hs.43530	-1.158102605	-0.53135016	-0.15004722	1.00014055	0.87215164	0.84944598
IMAGE:76005	Hs.8610 Transc	-0.335148204	-0.63659005	-0.22473243	1.92786717	0.14969899	-0.33904324
IMAGE:182177	ADAM17 Hs.404	0.610118679	0.4606401	0.48474955	-2.08941621	0.07320127	-0.19586017
IMAGE:156045	SCAMP3 Hs.200	0.412021423	1.01925443	0.74487507	-1.5578312	-0.85913841	-0.66103801
IMAGE:109488	MGC33302 Hs.4	-0.83184519	-0.74946115	-0.63961577	0.63632988	1.33842495	1.1967244
IMAGE:756710	BAZ2B Hs.4703	-0.652592472	-0.79142556	-0.46095322	1.85086568	0.4464202	0.4449327
IMAGE:265045	GNG12 Hs.4311	0.947227599	0.72475953	0.28645009	-1.53471143	-0.81050685	-0.52082502
IMAGE:1601605	CTDSP1 Hs.444	0.7200208	0.20944496	0.96322639	-1.49513783	-0.82281468	-0.28027912
IMAGE:2466685	ABI2 Hs.471156	0.610248112	0.54119128	0.32573395	0.32418017	-0.74620078	-1.71230591
IMAGE:809751	AA454724::AI	-0.932041734	-0.85465171	-0.62992298	1.4188663	1.46072389	0.58785371
IMAGE:810904	CCDC8 Hs.9787	-0.963064826	-0.60405592	-0.36164102	1.47988734	0.60403422	0.71881083
IMAGE:81328	AI821352::T6	-0.500025981	-0.73261561	-0.19053388	1.95923964	0.07460821	0.05328189
IMAGE:810156	DTYMK Hs.4718	0.527757497	1.23000562	0.0427516	-1.47971224	-0.83556377	-0.37480817
IMAGE:1554564	CPNE2 Hs.3398	0.262070534	0.80843666	1.05291236	-1.26057779	-0.88552985	-0.7757936
IMAGE:938231	Hs.185118 Tran	-0.904411806	-0.5210522	-0.43811609	1.69052378	0.24196899	0.75334834
IMAGE:187266	EIF2B2 Hs.4091	1.107393215	0.62648269	-0.07404364	-1.41878969	-0.61927594	-0.47019368
IMAGE:279163	N46324::N48	-0.885458011	-0.90766337	-0.13713726	1.71895356	0.59064353	0.55150657
IMAGE:1459519	AA865075::AI	-0.118733741	-1.05331583	-0.721964	1.28564964	0.56926543	0.80561429
IMAGE:291426	ACSS1 Hs.5293	0.300868212	0.66037695	0.85497343	-1.53905794	-0.68811065	-0.28341595
IMAGE:1912972	RP13-297E16.1	0.972118979	0.08320814	0.80666477	-1.06199451	-0.59920936	-0.93011777
IMAGE:2490935	PSMB4 Hs.8954	0.718726108	0.8032511	0.26873117	-1.63730732	-0.67130745	-0.310265
IMAGE:783559	BRAP Hs.57744	-0.66978146	-0.72454924	-0.53962744	1.29042189	0.76218126	0.7134272
IMAGE:814260	FVT1 Hs.74050	0.457898936	-0.21084451	0.69874777	-2.08897633	0.3852901	0.45966988
IMAGE:1704337	Hs.135282 CDN	-0.555103021	-1.04256209	-0.16235029	1.65482749	0.21212274	0.7324853
IMAGE:137971	NKIRAS2 Hs.63	0.688960395	0.50654708	0.56032459	-1.83965822	-0.59417104	-0.05983768
IMAGE:291091	ARMCX6 Hs.835	-0.476149194	-0.95094122	-0.15129149	2.17597037	-0.06507925	0.21885888
IMAGE:825461	GADD45B Hs.11	1.074843107	0.51405182	0.35641262	-1.05539173	-1.20067808	-0.57278836

IMAGE:251195	Hs.539412 Trar	-0.742686376	-0.6727159	-0.44646299	1.4006898	0.67705968	0.60343268
IMAGE:175968	Hs.593276 CDN	-1.767420578	0.64985157	-0.86671087	1.50329408	0.85258648	0.40386153
IMAGE:2307039	CARM1 Hs.3714	0.745583218	0.7507858	0.51979134	-1.68792255	-0.92626516	-0.28010499
IMAGE:244073	ARFGEF2 Hs.625	-0.395516432	-0.37225194	-1.11555248	1.0415022	1.05022639	0.45436457
IMAGE:79051	MGC11271 Hs.1	-0.907748805	-0.7256834	-0.30620471	0.94076124	0.91587897	0.97626399
IMAGE:2551147	MALL Hs.185055	1.206699237	-0.20415222	0.55977223	-0.71558588	-0.97796984	-0.50998009
IMAGE:730346	HEBP1 Hs.2941	0.946490735	0.38048299	0.78926636	-1.50542339	-0.99444417	-0.47717598
IMAGE:140108	SRPK2 Hs.2851	-0.660202079	-1.02762134	-0.31625683	0.39281131	1.31065778	1.22358707
IMAGE:240050	H82232::H82	-0.724379902	-0.7998556	-0.37544915	1.1323852	0.86171193	0.76156755
IMAGE:770212	CHI3L1 Hs.3822	-0.836265091	-0.61914381	-0.14572833	0.85614213	0.9882369	0.52089473
IMAGE:767475	CART1 Hs.4168	-0.890934354	-0.38334377	-0.9968663	1.17060066	0.9374952	1.04940421
IMAGE:432729	AA701596 134	-0.928916913	-0.55653271	-0.21662713	1.9983589	0.0042835	0.49631596
IMAGE:322123	TTC8 Hs.303055	-1.131881171	-0.24029828	-0.61504916	0.56141115	1.40531582	0.86035365
IMAGE:129817	N4BP1 Hs.5118	1.139798372	0.84472964	-0.30419424	-0.74748891	-0.86505536	-0.98400494
IMAGE:1756945	HOXC6 Hs.5490	0.670090793	1.25324569	-0.16945949	-1.11865252	-0.68618614	-0.86834172
IMAGE:194656	GAS2L3 Hs.205	-0.804229898	-0.35618699	-0.82873225	1.19561164	0.67226985	0.90865915
IMAGE:1055713	Hs.538323 Trar	-0.909418096	-0.06620527	-0.48938377	2.21079094	0.22738286	-0.36300904
IMAGE:814616	BTBD15 Hs.178	-0.568521163	-0.77269402	-0.59779878	0.4850236	1.51506004	0.75898761
IMAGE:1896952	Hs.593163 CDN	-0.64380182	-0.91582275	-0.36024061	0.39063333	1.30836052	1.09074377
IMAGE:1635111	AMDHD2 Hs.433	0.287982485	1.12697708	0.55715992	-1.25388858	-0.91501342	-0.64998726
IMAGE:1700880	NXF2 Hs.530358	-0.171443388	-1.15356878	-0.31815595	2.02484968	0.29021617	0.07014733
IMAGE:1631830	MGC2463 Hs.52	0.538880556	1.19163647	-0.22204634	-0.26065219	-0.87960467	-1.17796075
IMAGE:263159	TMEM115 Hs.91	0.723490919	0.18115255	0.93627871	-1.74363022	-0.56022355	-0.22345982
IMAGE:195943	R91385::R926	-0.708687901	-1.03532732	-0.1673114	1.25821795	0.4607482	1.10619593
IMAGE:1473071	LOC388963 Hs.4	-0.884892018	-0.81657228	-0.25927844	0.81895338	1.53997058	0.51737055
IMAGE:770580	CIZ1 Hs.212395	0.492878466	0.45731497	1.02328255	-1.73161796	-0.83236966	-0.14040572
IMAGE:302369	NCK1 Hs.47769	0.662599904	1.13780082	0.02077009	-0.87347164	-0.87347164	-0.97962042
IMAGE:1914166	C19orf24 Hs.59	0.435709467	0.84836431	0.46905531	-1.79366872	-0.4056479	-0.31311318
IMAGE:109314	T80848::T809	0.033529736	-0.92004761	0.96711221	-1.71275853	0.72662446	1.10702063
IMAGE:1895722	ZNF577 Hs.148	-0.741048447	-0.93334759	-0.17030117	1.34306616	0.7234016	0.65800276
IMAGE:187147	RIN2 Hs.472270	0.682514186	0.56289162	0.60300036	-0.4061266	-0.61961268	-1.59611987
IMAGE:815542	MX1 Hs.517307	0.953587855	0.39642953	0.32760408	-1.17909367	-1.05054343	-0.20489407
IMAGE:510532	Hs.63224 Trans	-0.621405492	-0.61047388	-0.30657512	2.07186995	-0.35822698	0.51739499
IMAGE:1566137	Hs.603046 Trar	-0.652905179	-1.09830629	0.00969675	1.19028081	0.87753177	0.54688369
IMAGE:80095	GFM2 Hs.27715	-1.122725105	-0.64766455	-0.23343493	0.81879523	1.10122452	1.0273584
IMAGE:252349	RAB22A Hs.529	1.05085961	0.71365622	-0.04057831	-0.90818888	-0.79928595	-0.88857603

IMAGE:253534	RGNEF Hs.4825	-0.401889556	-1.17208263	-0.12321389	1.09743027	0.73013903	0.68740633
IMAGE:223121	PEX14 Hs.14998	-0.30333101	-0.63434448	-0.53403737	2.1223457	0.12121884	-0.16950461
IMAGE:756488	TARBP2 Hs.326	0.489963726	1.04552159	0.27551839	-1.88865729	-0.53698499	-0.22198368
IMAGE:1031640	Hs.501423 Tran	-0.913013151	-0.1844527	-0.74210273	1.75828386	0.92412715	-0.10858381
IMAGE:183315	C10orf116 Hs.6	-0.418637795	-1.20369837	-0.05870748	1.05052323	0.69624802	0.76011735
ATCC:186006	IFNG Hs.856 Int	0.407107106	1.13855702	0.32135091	-1.65280719	-0.39673113	-0.67064651
IMAGE:726699	MVP Hs.632177	1.010693985	-0.40797079	1.1129401	-0.86104887	-0.89779357	-0.53641747
IMAGE:915928	DYNLL1 Hs.5120	-0.652412085	-0.91856102	-0.43746514	1.26702869	0.98454469	0.6517177
IMAGE:770334	Hs.540231 Tran	0.291709662	1.3146823	0.29218436	-1.10508708	-0.94060447	-0.72912684
IMAGE:62108	AI821283::T4	-0.938755645	-0.62642136	-0.19160304	1.25722997	0.77801119	0.55202815
IMAGE:645315	LOC441601 Hs.	0.757371806	0.95044457	0.27117948	-1.24071192	-0.74705997	-0.91292702
IMAGE:256664	H2AFX Hs.4778	0.494043113	1.12833527	0.15434443	-0.82193692	-1.13168292	-0.67287826
IMAGE:249753	FLJ10120 Hs.59	-1.102399117	-0.44420876	-0.17228891	0.34585757	0.910573	1.2788424
IMAGE:364448	IKBKE Hs.32104	-0.335980995	0.82404336	0.92304218	-1.56912089	0.06322608	-0.38000146
IMAGE:633045	CDH13 Hs.4360	1.289845548	0.19658236	0.17897181	-0.55855819	-0.87554816	-1.01925028
IMAGE:1632161	GGT1 Hs.35654	0.665314723	0.80938296	0.11107574	-1.74295537	-0.66244359	0.05450777
IMAGE:1870049	DDO Hs.591348	-1.115389799	-0.38614821	-0.47243452	0.770231	1.26949222	0.80312693
IMAGE:80854	Hs.570098 Tran	-0.778183457	-0.88464211	-0.49751973	2.00221135	0.93137661	0.18255006
IMAGE:302540	UGCG Hs.30424	1.095095514	0.50066363	0.18604167	-1.00462839	-0.69920207	-0.92236034
IMAGE:2506797	RNASEH1 Hs.56	0.800938506	0.87402874	0.23599399	-1.39757276	-0.86229427	-0.54757632
IMAGE:79760	ADAMTS7 Hs.16	-0.554569187	-0.67230929	-0.51855457	1.6713866	0.59305051	0.22407381
IMAGE:730150	LOC144363 Hs.	-0.555405757	-0.9298366	-0.4563376	0.51191717	1.27247983	1.01388853
IMAGE:154720	ARD1A Hs.4332	0.633974938	1.00990148	0.39498426	-1.83635549	-0.72706404	-0.39612543
IMAGE:795758	CRISPLD2 Hs.51	0.555789376	-0.10794709	1.12944732	-1.26320412	-0.26404369	-0.55632478
IMAGE:940585	KCTD1 Hs.5266	-0.738330644	-0.92959918	-0.17317079	0.90251972	1.01892061	0.7969179
IMAGE:1948395	ASXL1 Hs.37404	-0.645694788	-0.17745128	-1.05580853	0.18330414	1.09553432	1.27564131
IMAGE:897636	SEC13L1 Hs.166	0.935164906	0.72513835	0.25484358	-1.33332783	-0.81443869	-0.66124284
IMAGE:1657538	AI040814 284	-0.827062017	-0.47904715	-0.55481559	1.57171496	0.59675335	0.48421493
IMAGE:753931	CXCR4 Hs.4219	0.315833758	1.0965741	0.20635536	-1.21339449	-0.62359887	-0.53956263
IMAGE:196070	FALZ Hs.444200	-0.71071172	-0.36080821	-0.94946941	1.65043863	0.92380078	0.21987725
IMAGE:784212	FLJ20152 Hs.48	-0.821144807	-0.95268491	-0.17620977	0.23589373	1.37021901	1.27489406
IMAGE:841486	ASRGL1 Hs.535	-0.337589262	-0.69792331	-0.68479569	1.78480694	0.44340801	0.18104853
IMAGE:240586	ST3GAL1 Hs.584	-0.850781458	-0.42105892	-0.77142571	1.40373578	0.91139584	0.55691108
IMAGE:257206	ZC3H6 Hs.1904	-0.664256849	-1.20301662	0.1225182	1.03167532	0.52310467	1.09298247
IMAGE:970480	DKFZp547E087	-0.906833457	-0.81113898	-0.13489804	1.29872478	0.74442784	0.7024286
IMAGE:1638475	PER2 Hs.58756	-0.548162071	-1.0813	-0.11195831	0.36267292	0.65536375	1.56610433

IMAGE:504927	PDZK1IP1 Hs.43	0.354072999	0.53565038	0.62089088	-1.68230475	-0.44667486	0.01828094
IMAGE:745560	F25965 Hs.5291	1.079045927	0.44456917	0.82325949	-1.59209409	-1.31304786	-0.40935505
IMAGE:268960	Hs.633550 Tran	-1.111158886	-0.70817692	-0.23109828	1.69200315	0.97882257	0.34705085
IMAGE:1862615	AI053439::AI	-0.836011131	-0.60835175	-0.41641739	1.42987099	0.75462743	0.50256764
IMAGE:30170	CASP3 Hs.1411	0.167612625	1.24409859	0.32733419	-0.9588447	-0.92586127	-0.64202858
IMAGE:344372	Hs.633756 Tran	-0.701184778	-0.83349177	-0.58582962	0.94999094	0.81786853	1.26644238
IMAGE:295514	N74930::W23	-0.84629108	-0.53438204	-0.40513539	1.63601852	0.60830466	0.33310573
IMAGE:207293	H59625::H59	-0.853143953	-0.82824709	0.12560153	1.81654667	0.25219378	0.29634421
IMAGE:1892001	STAB2 Hs.40824	-0.768667168	-1.03200039	-0.30153694	0.77784303	1.49056012	0.80951935
IMAGE:327480	COL23A1 Hs.41	0.813410769	0.18712235	0.73343856	-1.9078124	-0.23048355	-0.27930193
IMAGE:2407433	DPYSL3 Hs.519	0.665205176	0.38157553	0.93691276	-1.5623211	-0.77056174	-0.40842918
IMAGE:840786	RG9MTD3 Hs.55	0.894930823	0.27044998	0.57717879	-1.66879057	-0.50831808	-0.29243606
IMAGE:322643	SQRDL Hs.5112	1.132311372	0.59707407	0.12464974	-1.30987471	-0.64967498	-0.79034065
IMAGE:770709	SMG5 Hs.51683	0.714809861	0.65016076	0.78144817	-1.5048922	-0.79623858	-0.72313536
IMAGE:711768	GALE Hs.63238	-0.974902769	-0.38805418	-0.48473434	0.49221872	1.12547379	1.03266083
IMAGE:1587791	KCNH2 Hs.4388	0.450970974	1.08357332	0.73504523	-1.16544737	-1.14754353	-0.90763208
IMAGE:897531	MCAM Hs.59903	0.509059398	0.7044005	1.05095007	-1.97358125	-0.43255704	-0.72773914
IMAGE:491727	ALPP Hs.284255	-0.979837756	-0.96336107	0.34735905	0.91907052	0.74396653	0.81756238
IMAGE:1324551	SLCO4A1 Hs.23	0.878376662	0.49438361	0.58225229	-0.9459363	-1.1638783	-0.67714116
IMAGE:121994	TMCO4 Hs.4667	-1.440168892	-0.45278913	-0.08315466	0.78554964	0.84251386	1.31531686
IMAGE:1693166	Hs.545194 Tran	-0.768499238	-0.65396755	-0.77335816	1.36022833	1.08561259	0.65455697
IMAGE:796986	TEX264 Hs.517	0.730024427	0.9814071	0.46227265	-1.21176058	-1.35810817	-0.57511935
IMAGE:840687	MUC1 Hs.89603	-1.047443726	0.07685796	-0.37805907	-0.10347909	1.26809221	0.76383936
IMAGE:282481	ZNF589 Hs.172	-0.757761276	-0.94854475	-0.44614826	0.83289598	1.2621588	1.02208959
IMAGE:222518	CCDC64 Hs.369	0.813319784	0.63264763	1.25263838	-1.4389012	-1.69564584	-0.60020206
IMAGE:855657	NMT2 Hs.60339	-0.853770758	-0.40081769	-0.96310426	0.94320341	0.91196526	1.23059432
IMAGE:69893	Hs.632958 Tran	-0.980309735	-0.34245646	-0.63404653	1.26133664	0.80119491	0.71417589
IMAGE:194704	Hs.418344 Tran	-0.797378516	-0.50866329	-0.61781173	1.26816766	0.42314749	1.04001222
IMAGE:884567	BMPR2 Hs.4711	-0.606975978	-0.68682706	-0.59308883	1.50482479	0.5626688	0.61457201
IMAGE:701778	C10orf78 Hs.93	-1.156707929	0.2355211	-1.24753784	0.52699578	1.35339911	1.06080766
IMAGE:469229	XPO6 Hs.46046	0.763247722	0.95138553	0.12541466	-1.54098158	-0.555323	-0.63241362
IMAGE:502536	NRP2 Hs.47120	0.584541208	0.9696966	0.32083121	-1.12037863	-0.64674159	-0.96527551
IMAGE:506504	PAPPA Hs.49492	0.993121879	0.19533403	0.79740841	-0.80425878	-0.98110831	-0.99407727
IMAGE:593023	DTNB Hs.30772	-0.861014773	-0.11938882	-1.07943886	0.42235371	1.16905929	1.22849096
IMAGE:287807	Hs.524453 Tran	-0.677778912	-0.86323294	-0.30771703	1.60824167	0.48240174	0.60552065
IMAGE:781089	PTTG1 Hs.3509	0.568679742	1.01161131	-0.00744995	-1.42084778	-0.53657792	-0.40369845

IMAGE:123331	SLC36A1 Hs.269	0.985161574	0.55358855	0.76240043	-1.55334574	-0.77822771	-0.92955228
IMAGE:1127918	ATRX Hs.533526	-1.148084384	-0.3502976	-0.3542346	1.05234208	0.99542313	0.642601
IMAGE:214424	NRG3 Hs.12511	-0.659487437	-0.92345694	-0.76851469	1.50352644	0.68564315	1.14589034
IMAGE:1324438	UGT2B7 Hs.631	-0.802098133	-0.42380731	-0.61405454	0.99606875	0.60584156	1.00451602
IMAGE:124474	WNK1 Hs.35660	0.016172168	-0.47335552	-1.29117511	1.74230524	-0.13832003	0.6957587
IMAGE:565677	LOC375748 Hs.4	-0.968048678	-0.69749262	-0.47892577	1.33095783	1.00276651	0.76324483
IMAGE:753330	C12orf23 Hs.25	-0.467392983	1.82514727	-0.65106047	-0.04357034	-0.36069406	-0.81854144
IMAGE:1655864	AI033961 136	-0.66816713	-1.14341922	0.01356913	1.50892917	0.5169166	0.67457234
IMAGE:770804	TRAF2 Hs.52250	-0.063262944	1.21670824	0.41811752	-1.64629977	-0.37560374	-0.23091134
IMAGE:277871	TTC6 Hs.509182	-0.78672923	-0.75715294	-0.51019095	1.94648938	1.13683853	-0.12976596
IMAGE:1589620	Hs.529249 CDN	-0.434558497	-0.78925484	-0.43280547	2.15671153	0.09272779	0.12728751
IMAGE:1323462	DNAH5 Hs.2123	-1.058980328	-1.26296383	0.0952473	1.26920172	0.86994389	1.2247115
IMAGE:265625	FDX1 Hs.744 Fe	-0.899829452	-0.90671841	-0.30655751	0.63625001	1.36148657	1.09528209
IMAGE:2139152	RAPGEF2 Hs.11	-1.097085366	-0.99083795	0.06788628	1.00067607	0.91630313	1.13004793
IMAGE:357037	CDON Hs.38034	0.373603378	-0.00725143	1.35755299	-1.46726956	-0.0162874	-0.76291219
IMAGE:129342	C4orf29 Hs.445	-1.109363785	-0.31989117	-0.65514667	1.40180801	0.53798438	1.02302338
IMAGE:293457	MOBKL2C Hs.63	0.543839542	0.52503009	0.74773397	-1.69956343	-0.33964027	-0.49876821
IMAGE:1869514	SLC7A13 Hs.14	-0.530594174	-0.54607564	-0.36649064	2.48650381	0.06660335	-0.47998913
IMAGE:813462	SIAH1 Hs.29592	-0.452385873	-0.76922004	-0.69170333	0.86719255	1.22452817	0.60531732
IMAGE:1502650	VPS13A Hs.459	-0.342726405	-0.96755424	-0.77649118	0.54253242	1.49767557	0.89582695
IMAGE:852829	KPNA3 Hs.5279	1.053188296	0.02264662	0.62437816	-1.64259519	-0.51598607	-0.2356438
IMAGE:117402	PAPSS2 Hs.524	-0.715104116	-0.67398503	-0.51407747	1.7367356	0.57222514	0.41726982
IMAGE:1423061	Hs.600128 Tran	-0.836869556	-0.74094032	-0.23388575	0.43984587	1.18427636	1.03494977
IMAGE:1688460	CCNH Hs.29252	-0.764908834	-0.55454818	-0.40778493	1.94482991	0.16742216	0.37666462
IMAGE:208275	H65318::H62	-0.65856983	-0.35700158	-0.5376907	2.11494519	0.44058034	-0.36005504
IMAGE:1613940	RNF7 Hs.13462	1.034151448	0.47341788	0.28200958	-1.50327333	-0.83924674	-0.27134591
IMAGE:282500	PLXNB3 Hs.632	-0.976992708	-0.8821102	-0.23101991	0.8745794	1.1226917	1.08015816
IMAGE:1693368	AI140869 284	-0.357739528	-0.82028947	-0.72464355	1.55380689	0.56598838	0.55305266
IMAGE:743362	ALKBH7 Hs.111	0.444443808	0.68255337	0.3807495	-1.73232402	-0.41528052	-0.0188281
IMAGE:241894	AEBP2 Hs.1264	-0.676266342	-0.42273131	-0.68688325	1.66779519	0.62188632	0.21741903
IMAGE:1544114	Hs.512544 CDN	-1.242417387	-0.40098018	-0.08810775	0.56252468	0.95065241	1.06205395
IMAGE:119004	SYK Hs.371720	-0.423284011	-1.18890323	-0.16423991	1.81226655	0.50150342	0.30981079
IMAGE:1623955	Hs.482077 Tran	-0.542787063	-0.74858812	-0.49352866	0.1961447	0.90166864	1.45616027
IMAGE:594037	FGF12 Hs.58475	-0.824779108	-0.43311869	-0.160753	1.80778977	0.31301238	-0.03301421
IMAGE:704905	RAP1A Hs.1903	-0.742415208	-0.22785512	-0.90671334	0.84427403	1.18744843	0.55707471
IMAGE:530035	S100A13 Hs.516	0.722749072	0.33141742	1.13214218	-1.33234413	-1.34137486	-0.32270848

IMAGE:128290	EVI1 Hs.554762	-1.280471247	-0.4757111	0.03437442	0.56427587	1.30795631	0.71907331
IMAGE:504859	Hs.444781 Trar	-0.5209317	-0.65701735	0.00224202	-0.55835525	1.28258119	1.03989511
IMAGE:343387	Hs.549644 CDN	-0.364722716	-1.21742448	0.17813847	0.48964348	0.95680498	0.70409925
IMAGE:347268	NES Hs.527971	0.410127558	0.48375604	1.08014672	-1.23982531	-0.94592497	-0.50525852
IMAGE:843075	H2AFY Hs.42027	1.047829649	0.4413832	0.14448487	-1.56156433	-0.38401647	-0.46884456
IMAGE:1476307	YWHAB Hs.6435	0.188698287	0.7358574	0.22884526	-2.11456139	0.1251652	0.31650608
IMAGE:809974	LOC150223 Hs.1	0.586536676	0.8291413	0.59590036	-1.394096	-0.92165541	-0.55264101
IMAGE:138059	RPP25 Hs.8562	0.631492972	0.79501397	0.68381969	-0.36202893	-1.34315489	-1.2893512
IMAGE:2568611	CTNNA1 Hs.534	1.082936035	0.64007336	0.38210586	-1.39820208	-0.86676688	-0.79717747
IMAGE:296375	ATP6V0A2 Hs.20	1.29465062	0.66168025	0.14574968	-1.52327733	-0.58103401	-1.01237206
IMAGE:1551969	DKFZp434K191	-0.71736854	-0.74515921	-0.36155229	1.49027511	0.70691318	0.4485437
IMAGE:433465	PDZK1IP1 Hs.43	-0.591700722	-0.67738458	-0.5026346	2.06449902	0.05769276	0.40972942
IMAGE:128208	DKFZp586I1420	0.630337376	0.19913276	0.40255601	-1.89385542	-0.10155839	0.24801359
IMAGE:288840	COL4A3BP Hs.2	0.800380367	0.39871418	0.80125145	-1.51768117	-0.73860604	-0.54391893
IMAGE:324897	Hs.56044 Trans	-0.486234375	-1.08637138	-0.58660331	1.87728096	0.70400274	0.51087907
IMAGE:813648	DLD Hs.131711	-0.788302791	-0.25364669	-0.828402	0.35001346	1.06233133	1.18608194
IMAGE:199327	EXOC4 Hs.3212	-0.775481447	-0.56150865	-0.51103542	1.58636617	0.54918338	0.50872988
IMAGE:345128	RTKN Hs.19285	0.837046563	0.79722208	0.36977262	-1.31944923	-0.78845612	-0.80571339
IMAGE:2516566	RPP38 Hs.94986	1.220943791	0.4709033	0.45911227	-1.4790928	-1.14938068	-0.48318751
IMAGE:46977	Hs.539223 Trar	-0.558088953	-0.99278536	-0.44155332	1.44174574	0.7883138	0.64819358
IMAGE:2261214	SURF2 Hs.1594	0.71357664	0.19164417	0.90795149	-2.05716487	-0.11341636	-0.32218935
IMAGE:824962	KPNA2 Hs.5942	0.897597279	0.79259554	-0.2488662	-1.52463738	-0.17128158	-0.52828751
IMAGE:461436	Hs.531664 Trar	-1.069150983	-0.75411471	-0.46231515	1.41192724	1.09018807	0.81067717
IMAGE:1417886	CERK Hs.20066	0.940777329	0.43034622	0.77655167	-1.55783841	-0.61742458	-0.85211193
IMAGE:46896	CYFIP2 Hs.5197	0.579140622	1.31823862	-0.40663133	-0.83369139	-0.47107143	-1.03301688
IMAGE:1573505	Hs.560445 Trar	-0.751926035	-0.8507262	0.13068877	2.13732012	-0.12520366	0.22850093
IMAGE:126221	TPD52L2 Hs.473	0.730573852	0.40477959	0.50817537	-1.82642342	-0.3749807	-0.13684525
IMAGE:1555741	CH25H Hs.4735	0.699109793	0.66379881	0.40706716	-0.72820704	-0.76550859	-1.05948122
IMAGE:136801	R36086::R361	-0.484096016	-1.17131725	-0.44652792	1.43525563	0.79098572	0.81503846
IMAGE:1626299	Hs.634037 Trar	-0.501718309	-1.32843312	-0.05681521	1.15563509	0.63481059	1.02580046
IMAGE:2213824	PI3 Hs.112341 F	0.049754819	0.77714906	0.46688399	-1.4463986	-0.1313641	-0.2461981
IMAGE:713251	RBM6 Hs.18887	0.914001315	0.96604016	-0.01600716	-0.97445115	-1.16657007	-0.65903204
IMAGE:251826	WBP5 Hs.53328	1.057296529	0.42379241	0.4326837	-1.02048584	-0.93157298	-0.81042921
IMAGE:430399	DCUN1D1 Hs.10	-1.154155794	-0.41315698	-0.34081	0.71100993	1.45678938	0.60918235
IMAGE:772481	PHYHIP Hs.3346	-0.632194649	-1.18337276	0.74707876	0.74956902	0.41587384	0.62405979
IMAGE:824851	AYTL2 Hs.36885	1.16411696	0.51848641	-0.07517876	-1.36073156	-0.62258687	-0.44661317

IMAGE:327228	KIAA0738 Hs.40	-0.814601634	-0.1880241	-0.93475709	1.06549955	1.09148409	0.51540133
IMAGE:953521	MCF2L Hs.1704	-0.829374125	-0.46603515	-0.54553496	1.35015508	0.76626366	0.50861388
IMAGE:2028876	SFRS5 Hs.6323	-0.463338584	-0.76158424	-0.57560677	0.18795537	1.18015476	1.18878258
IMAGE:428412	GZMK Hs.27793	0.4780426	0.93481019	0.20756041	-0.63028536	-1.00303156	-0.74541277
1293118	HDAC11 Hs.404	0.642242624	0.81289583	0.71646322	-1.49146414	-0.97137819	-0.61544437
IMAGE:486626	TMEM64 Hs.567	-0.811245675	-0.89222698	-0.20696672	0.88093345	0.88687502	1.0461089
IMAGE:450198	PTPRC Hs.1920	-0.903836289	-0.81082821	-0.19639656	1.51027769	0.7540047	0.55321005
IMAGE:305485	Hs.524270 Tran	-0.888107361	-0.78464468	0.0282764	1.12443889	0.79138759	0.55795607
IMAGE:2017415	CENPA Hs.1594	0.576433652	1.04347375	0.06268955	-0.96263645	-0.69884528	-0.84674131
IMAGE:1700703	SUZ12P Hs.628	-0.865514702	-0.53865903	-0.41827259	1.26956477	0.95694836	0.40258821
IMAGE:268812	DIRC2 Hs.4773	0.89213552	0.59545061	0.64050649	-1.40767541	-0.76653428	-0.85780261
IMAGE:1118429	GAB2 Hs.42943	-0.592663025	-1.08275722	-0.03594665	1.32881434	0.60443033	0.62481901
IMAGE:241043	TncRNA Hs.523	-0.424223006	-0.56571462	-0.78799337	0.37486227	1.24435102	0.85068485
IMAGE:588608	B2M Hs.534255	-0.945640823	-0.51838412	-0.50219334	1.43867645	0.93946072	0.44564192
IMAGE:1941742	ATG7 Hs.38032	0.78194866	1.12424595	0.38625299	-1.23315551	-1.1201974	-0.98875524
IMAGE:1585094	C4orf21 Hs.380	-0.776591649	-0.77046756	-0.40404306	1.70863919	0.71113608	0.40586738
IMAGE:588430	PIGO Hs.52209	0.192094497	0.74192083	0.95685294	-1.53798404	-0.69450046	-0.36460466
IMAGE:1892010	AI277315 292	-0.723838609	-0.53670232	-0.6007889	1.63322571	0.44583761	0.56273421
IMAGE:1861970	AI053937::AI	-0.700238133	-0.86732451	-0.4102014	0.98363	1.11495437	0.76551135
IMAGE:1607112	AP3S1 Hs.4061	1.190223594	0.21256312	0.37405814	-1.05061043	-0.92568715	-0.59545516
IMAGE:417804	KIAA0376 Hs.47	-0.683064127	-0.70423775	-0.25574191	1.75223942	0.39654319	0.25184759
IMAGE:139189	R68654::R68	-0.498295463	-0.6650976	-0.67803328	1.48568547	0.30197183	0.80497389
IMAGE:510336	FXYD3 Hs.3013	0.409119467	0.7527701	0.41499384	-1.4834556	-0.87545833	0.09733729
IMAGE:1412238	AMY1A Hs.4845	-0.490155009	-0.92711207	-0.19520899	1.00333957	0.79455162	0.57202067
IMAGE:32551	PGK1 Hs.78771	-0.625324159	-0.57857425	-0.5774055	2.08746447	-0.00890714	0.44904716
IMAGE:1836893	Hs.635128 Tran	-0.697174294	-0.81912711	-0.3585759	1.59977105	0.53304263	0.58985829
IMAGE:1126251	SFXN1 Hs.3694	-0.74845955	-0.37182292	-0.31999219	1.77707908	-0.16916477	0.47249965
IMAGE:969906	Hs.116928 Tran	-0.608092871	-0.58776964	-0.62525471	1.13033372	0.86003475	0.58499369
IMAGE:72063	RBM4B Hs.5238	-0.777314134	-0.66011079	-0.641605	1.06555424	1.2175661	0.6750233
IMAGE:1503971	ARL6 Hs.37380	-0.998779209	-1.03412258	-0.21981138	1.28899701	1.0769368	0.95818309
IMAGE:1555233	C1orf117 Hs.12	-0.873641207	0.37996101	-1.09089698	0.4393178	1.01916255	0.64566118
IMAGE:1879938	Hs.101139 Tran	-1.060069164	-0.58230454	-0.01830309	2.01293842	0.27156596	0.20193504
IMAGE:341095	TPCN2 Hs.1318	-0.341783925	-1.35102699	-0.11294159	1.60848764	0.76040116	0.31150455
IMAGE:137275	INPP5E Hs.1209	-0.674579284	-0.7047901	-0.61726505	0.38344671	1.31760836	1.13958032
IMAGE:1914797	AI309311 544	-1.059367596	-0.75256858	-0.29918781	0.75351631	1.17429968	1.16407305
IMAGE:754040	SYNCRIP Hs.571	0.475293117	1.1914526	0.02368096	-0.62689514	-1.09356894	-0.80925569

IMAGE:344505	PYDC1 Hs.58314	0.910534153	-0.04113707	0.71948705	-0.81521647	-0.96224433	-0.42599363
IMAGE:796984	CYBB Hs.292356	0.237225695	1.31745935	0.22488017	-1.4826606	-0.4950184	-0.63544878
IMAGE:434999	Hs.573560 Trar	-0.709436954	-0.95761707	-0.21384272	1.03663269	0.98971593	0.74153581
IMAGE:127209	ACSM2 Hs.2982	-0.503461422	-0.41707704	-0.37201254	1.9840602	0.09851765	-0.23675448
IMAGE:489213	GLT28D1 Hs.110	-0.540337711	-0.33727926	-1.16516838	0.47715815	1.35710005	0.93862773
IMAGE:156430	AQP7 Hs.45532	-0.760317278	-0.69780763	-0.4727729	1.33246893	0.75412337	0.6915612
IMAGE:757138	Hs.599735 Trar	-0.943050986	-1.03103168	0.16270052	1.12972306	0.83645409	0.79157119
IMAGE:591143	ARL6IP4 Hs.103	0.362601074	0.62481019	0.90816519	-1.50817634	-0.95626846	-0.15187858
IMAGE:868526	ARL13B Hs.5330	-1.114365792	-0.82245117	-0.17207794	1.3694196	0.79769741	0.95320585
IMAGE:1523456	KIAA0773 Hs.13	1.095476449	0.44675498	0.28438927	-0.69870178	-1.19302754	-0.77710441
IMAGE:79828	HD Hs.518450 H	0.814643059	-0.02335944	0.8921606	-1.66917511	-0.35950747	-0.2734436
IMAGE:1558669	C19orf30 Hs.32	-0.32428928	-1.07846279	-0.21442818	1.73682754	0.33871547	0.29662032
IMAGE:46919	CBWD1 Hs.5313	-0.641650742	-0.31689895	-0.44517591	1.76191843	0.12344417	0.10893183
IMAGE:131016	RAB8B Hs.3897	0.29355805	1.00767072	0.45767597	-1.01636769	-0.85105438	-0.65651605
IMAGE:1912713	BTNL9 Hs.54650	-0.652569621	-0.8522052	-0.2203761	0.66920304	1.10825122	0.7551781
IMAGE:1698590	Hs.65745 Trans	-0.385913127	-1.13914263	-0.26224858	1.2335303	0.72200877	0.65985529
IMAGE:183440	ARSA Hs.88251	0.414431917	0.83299332	0.65394205	-1.86353038	-0.84328696	0.01825192
IMAGE:2308994	TPX2 Hs.244580	0.605562633	0.51324839	0.26818089	0.61034426	-0.65178709	-1.97199982
IMAGE:1325580	AA875853 21	-0.848332037	-0.78793539	-0.04883147	1.47939236	0.45241349	0.58363464
IMAGE:813187	DDAH1 Hs.3798	-0.719226293	-0.41851172	-1.01091943	1.36397388	0.92710244	0.67917998
IMAGE:824568	MRPL4 Hs.2796	0.777136391	0.81701916	0.90157063	-1.5787388	-1.30354769	-0.63591013
IMAGE:1691661	DZIP3 Hs.40921	-0.504951452	-0.5164459	0.03101801	2.09927884	-0.48697086	-0.11898447
IMAGE:1878424	ARHGAP24 Hs.4	-0.405240058	-0.9598618	-0.15390981	0.29030965	1.11610027	0.83363013
IMAGE:1155286	Hs.122193 Trar	-0.561313763	-0.64406434	-0.16402613	1.97064167	-0.04083386	0.083292
IMAGE:277808	KIAA1370 Hs.15	-0.496763692	-1.02401015	-0.46029992	0.54122197	1.51687429	0.7984394
IMAGE:449346	AA777915 24	-0.108221213	-1.10705047	-0.25720689	1.64087368	0.19679205	0.30675041
IMAGE:343646	MRPL27 Hs.773	0.417704175	0.41013423	0.51535643	0.50911122	-0.57528296	-1.81978141
IMAGE:1743745	ODZ4 Hs.21308	-0.562019953	-0.94330071	-0.23654911	0.87013002	0.96675418	0.71678318
IMAGE:588728	Hs.387576 **T	0.562064724	0.65553526	0.73308119	-1.27523717	-0.91563524	-0.55187906
IMAGE:235008	GNA12 Hs.4873	0.94030608	0.84764935	0.24846915	-1.07420571	-1.18230523	-0.73600864
IMAGE:1917346	ATP6V0D2 Hs.4	-0.504869318	-0.82411706	-0.39660269	1.58290679	0.61012654	0.2961996
IMAGE:324345	UNC84A Hs.438	0.886030416	0.45201454	0.92029483	-0.98052339	-0.91199457	-1.26491801
IMAGE:950676	KIF1A Hs.51680	-0.256734948	-0.53956976	-1.04767123	0.70227529	0.91627862	0.88549218
IMAGE:1469381	KIAA1107 Hs.21	-0.325679582	-1.17918469	-0.18606411	1.51620691	0.7137998	0.25986985
IMAGE:2577055	GNB1 Hs.43042	0.488596152	1.16483749	0.05328889	-1.14232797	-0.72660583	-0.67782777
IMAGE:270997	GOLPH3 Hs.408	-0.775136193	0.05330319	-0.01918525	1.8426852	0.23287684	-0.96883097

IMAGE:246246	C6 Hs.481992 C	1.145516462	0.37108545	0.47403922	-1.00844571	-0.92881977	-0.93018642
IMAGE:109863	EMP2 Hs.53156:	0.517505579	0.30290603	0.3483807	-1.82482295	-0.00839105	0.16712072
IMAGE:255295	N23717 66319	-0.354075235	-1.00792217	-0.4252719	1.43198638	0.63995373	0.50264588
IMAGE:1568169	DENND1A Hs.43	0.093899257	1.04337002	0.75908296	-1.14426881	-1.0195815	-0.49090731
IMAGE:1873420	ZCCHC11 Hs.47	-0.850218591	-0.36833989	-0.698771	1.41096686	0.35335012	0.93698449
IMAGE:66599	NAT1 Hs.591847	0.828291366	0.756549	0.06682888	-0.42678623	-0.93588114	-1.09812927
IMAGE:911356	AA484178::AI	-0.779560868	-1.00252508	-0.06346411	1.48711638	0.4783791	0.78696357
IMAGE:415417	C14orf102 Hs.52	-0.749030136	-0.75546968	-0.17934504	0.90196193	0.91376777	0.66520132
IMAGE:565285	Hs.598400 Tran	-0.578781599	-0.34979697	-0.10629137	2.16568295	0.03088998	-0.67084087
IMAGE:825327	KRTCAP2 Hs.516	0.521138276	0.83948134	0.43479317	-1.65318848	-0.14345739	-0.78777503
IMAGE:1898791	C9orf114 Hs.224	0.372251407	0.46700739	1.03985039	-1.65358846	-0.52943792	-0.3756748
IMAGE:454150	EHBP1 Hs.27166	-1.060340835	-0.65050176	-0.15698721	1.20508734	0.81339218	0.74401838
IMAGE:1569217	ITCH Hs.632272	-0.936923355	-0.71279971	-0.60580451	1.2521567	0.89025491	1.08942862
IMAGE:685026	AMMECR1 Hs.48	0.964203061	0.09007647	0.29312239	-1.63492383	-0.18877994	-0.12411852
IMAGE:856936	NDST1 Hs.2220	1.090258763	0.21267191	0.33943446	0.2878356	-1.55387792	-1.11264676
IMAGE:301740	XIST Hs.529901	0.585646757	0.79870514	0.36108323	-0.77043939	-1.02937127	-0.72807121
IMAGE:2018976	PTTG1 Hs.35096	0.431746292	1.05198224	0.04986921	-1.33101005	-0.64775116	-0.3091681
IMAGE:1703568	ZNF284 Hs.445:	-1.243985234	-0.80552491	0.07266665	0.80402393	1.19074049	0.98866747
IMAGE:269300	DDAH1 Hs.3798	-0.750775697	-0.52814635	-0.60410225	0.43816323	1.23209876	1.00324889
IMAGE:1466893	CCDC73 Hs.632	-0.592719011	-1.23625914	-0.10320652	0.7980134	1.11450855	0.95995342
IMAGE:896949	HMGCR Hs.6434	-0.391541979	-0.73572075	-0.73094774	0.42367322	1.13067533	1.05023022
IMAGE:2409751	ESPL1 Hs.15347	0.710077167	1.0078031	0.18078662	-1.40637616	-0.78256944	-0.61385808
IMAGE:1629264	TIMM44 Hs.4657	0.825840797	0.5822906	0.18253233	0.23229216	-1.4116717	-1.16098296
IMAGE:811740	ITGA2 Hs.59177	1.326747187	-0.2224756	0.45666044	-1.32742272	-0.38843159	-0.51137863
IMAGE:897575	PCQAP Hs.51742	0.64476606	0.63594982	0.68262405	-1.81366943	-0.54568615	-0.4149983
IMAGE:1654630	KCNH2 Hs.4388	0.709502223	1.07873008	-0.3455322	-0.40963426	-0.91219441	-0.92860454
IMAGE:812964	FAM120B Hs.369	-0.804614048	-0.73191191	-0.6284288	0.92393523	1.0254386	1.14095111
IMAGE:2030270	PHF13 Hs.51607	1.112422086	0.35385057	0.60670774	-0.04241274	-1.75461702	-1.16076389
IMAGE:296334	CEP152 Hs.5973	-0.944186936	-0.10753777	-0.92389868	1.44765392	0.68394587	0.60086062
*mitoch. cont. IMAG	148361	-0.859842581	-0.97677006	-0.31677939	1.56088107	0.96649971	0.62351243
IMAGE:299272	Hs.597274 Tran	0.403958992	0.22519789	1.18762257	-1.17823355	-0.84282128	-0.4072087
IMAGE:1456508	LOC150159 Hs.4	-0.669550632	-0.60510491	-0.23009554	1.52482004	0.62360031	0.05118239
IMAGE:2325804	RNF40 Hs.65238	0.428286056	0.47222993	0.80180896	-1.88030519	-0.32469217	-0.14803781
IMAGE:563592	RBM7 Hs.53373	0.651052805	0.98528723	0.58791964	-1.50197396	-0.91706371	-0.77037193
IMAGE:504201	SAMD1 Hs.1403	0.489505394	0.43170054	1.0584269	-1.72000928	-0.42700586	-0.55782738
IMAGE:435536	YPEL2 Hs.46361	-0.76553196	-0.50434774	-0.49461417	1.71221287	0.5572201	0.25365429

IMAGE:327	COL5A2 Hs.445[illegible]	-0.84131356	-0.74565297	-0.23828187	1.31679073	0.81480385	0.54670755
IMAGE:857696	DIXDC1 Hs.446[illegible]	-0.472039516	-1.58917007	0.38299503	1.10805572	0.43133241	1.07368247
IMAGE:590150	MT2A Hs.53433[illegible]	0.866559659	0.44551992	0.00554347	-1.42468218	-0.53324638	-0.01712015
IMAGE:41905	KCNK2 Hs.4977[illegible]	-0.11165647	-0.2925777	1.6260919	0.22915389	-0.45981675	-1.19560076
IMAGE:1604327	FLJ10159 Hs.44[illegible]	-0.817376386	-0.48154974	-0.24864943	1.83588101	0.57653109	-0.15321113
IMAGE:253725	Hs.596678 Full-[illegible]	-0.655346384	-1.22185801	-0.4161858	1.46692352	1.1418146	0.72730073
IMAGE:178856	H49517::H49[illegible]	-0.546109704	-0.73476634	-0.57427617	1.22280294	0.87585665	0.54049968
IMAGE:305408	BXDC1 Hs.3722[illegible]	-0.810520079	-0.87867321	-0.46180322	0.82357297	1.42518663	0.86228436
IMAGE:131653	MRPS12 Hs.411[illegible]	0.680314875	0.80201511	0.30207233	-1.80827262	-0.56338528	-0.22942748
IMAGE:137794	ACVR2B Hs.517[illegible]	0.475559408	1.02992948	0.59035817	-1.06334901	-0.89981802	-1.03301401
IMAGE:238461	ABHD6 Hs.4764[illegible]	-1.011366009	-0.84002441	-0.33524466	1.0875686	1.18926416	0.91930869
