# Supplementary material for: Global Expression Profiling in Atopic Eczema Reveals Reciprocal Expression of Inflammatory and Lipid Genes
Source: PLoS One. 2008 Dec 24;3(12):e4017. doi: 10.1371/journal.pone.0004017 (PMC2603322; doi:10.1371/journal.pone.0004017)
Supplement: Table S2 — Enriched GO terms. Shown are Gene Ontology (GO) terms significantly enriched in 2181 genes induced (Gene Set 1) or 1896 genes repressed (Gene Set 2) in AE (P<0.05; FDR<0.05). (0.05 MB PDF) [file pone.0004017.s002.pdf]

**Supplementary Table S2. Enriched GO terms.** Shown are Gene Ontology (GO) terms significantly enriched in 2181 genes induced (Gene Set 1) or 1896 genes repressed (Gene Set 2) in AE (*P*<0.05; FDR<0.05).

| Gene Set 1 | Enriched GO Term | *P*-value |
|---|---|---|
| Gene_Set_1_4102 | immune response | 3.39E-74 |
| Gene_Set_1_4102 | response to stress | 1.18E-42 |
| Gene_Set_1_4102 | intracellular signaling cascade | 2.62E-38 |
| Gene_Set_1_4102 | response to other organism | 8.91E-38 |
| Gene_Set_1_4102 | extracellular region | 1.20E-34 |
| Gene_Set_1_4102 | cell adhesion | 4.93E-33 |
| Gene_Set_1_4102 | positive regulation of cellular process | 1.88E-31 |
| Gene_Set_1_4102 | death | 2.19E-31 |
| Gene_Set_1_4102 | enzyme regulator activity | 3.82E-28 |
| Gene_Set_1_4102 | extracellular region part | 6.02E-28 |
| Gene_Set_1_4102 | ATP binding | 2.57E-26 |
| Gene_Set_1_4102 | cell cycle | 1.18E-25 |
| Gene_Set_1_4102 | regulation of apoptosis | 3.70E-25 |
| Gene_Set_1_4102 | negative regulation of cellular process | 8.23E-25 |
| Gene_Set_1_4102 | peptidase activity | 9.37E-25 |
| Gene_Set_1_4102 | phosphorylation | 5.76E-24 |
| Gene_Set_1_4102 | protein kinase activity | 1.87E-23 |
| Gene_Set_1_4102 | proteolysis | 1.92E-23 |
| Gene_Set_1_4102 | extracellular matrix (sensu Metazoa) | 1.31E-21 |
| Gene_Set_1_4102 | humoral immune response | 1.44E-21 |
| Gene_Set_1_4102 | kinase activity | 2.75E-21 |
| Gene_Set_1_4102 | endopeptidase activity | 3.08E-21 |
| Gene_Set_1_4102 | cytoskeleton | 1.73E-20 |
| Gene_Set_1_4102 | regulation of signal transduction | 3.11E-20 |
| Gene_Set_1_4102 | protein kinase cascade | 9.46E-19 |
| Gene_Set_1_4102 | cell differentiation | 1.96E-18 |
| Gene_Set_1_4102 | GTPase regulator activity | 4.96E-18 |
| Gene_Set_1_4102 | cytoskeleton organization and biogenesis | 1.19E-17 |
| Gene_Set_1_4102 | membrane fraction | 1.55E-17 |
| Gene_Set_1_4102 | regulation of cell cycle | 6.49E-17 |
| Gene_Set_1_4102 | small GTPase mediated signal transduction | 7.64E-17 |
| Gene_Set_1_4102 | pyrophosphatase activity | 1.71E-16 |
| Gene_Set_1_4102 | cellular biosynthesis | 2.11E-16 |
| Gene_Set_1_4102 | locomotion | 1.26E-15 |
| Gene_Set_1_4102 | calcium ion binding | 1.32E-15 |
| Gene_Set_1_4102 | protein biosynthesis | 1.45E-15 |
| Gene_Set_1_4102 | receptor binding | 1.65E-15 |
| Gene_Set_1_4102 | GTP binding | 4.04E-15 |
| Gene_Set_1_4102 | organ development | 5.10E-15 |
| Gene_Set_1_4102 | cell activation | 1.19E-14 |
| Gene_Set_1_4102 | induction of apoptosis | 1.78E-14 |
| Gene_Set_1_4102 | transcription factor binding | 5.50E-14 |
| Gene_Set_1_4102 | transcription from RNA polymerase II promoter | 5.74E-14 |
| Gene_Set_1_4102 | humoral defense mechanism (sensu Vertebrata) | 6.76E-14 |
| Gene_Set_1_4102 | enzyme linked receptor protein signaling pathway | 1.11E-13 |
| Gene_Set_1_4102 | protein-tyrosine kinase activity | 2.21E-13 |
| Gene_Set_1_4102 | extracellular space | 3.68E-13 |

| | | |
|---|---|---|
| Gene_Set_1_4102 | GTPase activity | 4.51E-13 |
| Gene_Set_1_4102 | transmembrane receptor protein tyrosine kinase signaling | 8.11E-13 |
| Gene_Set_1_4102 | complement activation | 9.17E-13 |
| Gene_Set_1_4102 | protein serine/threonine kinase activity | 1.19E-12 |
| Gene_Set_1_4102 | regulation of cell proliferation | 1.35E-12 |
| Gene_Set_1_4102 | antigen processing | 3.57E-12 |
| Gene_Set_1_4102 | immunological synapse | 4.11E-12 |
| Gene_Set_1_4102 | enzyme inhibitor activity | 5.06E-12 |
| Gene_Set_1_4102 | guanyl-nucleotide exchange factor activity | 5.57E-12 |
| Gene_Set_1_4102 | antigen presentation | 6.57E-12 |
| Gene_Set_1_4102 | morphogenesis | 7.70E-12 |
| Gene_Set_1_4102 | small GTPase regulator activity | 7.83E-12 |
| Gene_Set_1_4102 | hemopoiesis | 8.88E-12 |
| Gene_Set_1_4102 | serine-type peptidase activity | 1.05E-11 |
| Gene_Set_1_4102 | transmembrane receptor activity | 1.87E-11 |
| Gene_Set_1_4102 | T cell activation | 2.41E-11 |
| Gene_Set_1_4102 | cytoskeletal part | 2.42E-11 |
| Gene_Set_1_4102 | enzyme activator activity | 2.59E-11 |
| Gene_Set_1_4102 | nuclear part | 4.77E-11 |
| Gene_Set_1_4102 | mitotic cell cycle | 4.89E-11 |
| Gene_Set_1_4102 | receptor complex | 5.23E-11 |
| Gene_Set_1_4102 | cellular localization | 5.41E-11 |
| Gene_Set_1_4102 | vacuole | 7.78E-11 |
| Gene_Set_1_4102 | extracellular matrix structural constituent | 8.43E-11 |
| Gene_Set_1_4102 | inflammatory response | 8.77E-11 |
| Gene_Set_1_4102 | regulation of protein metabolism | 8.77E-11 |
| Gene_Set_1_4102 | I-kappaB kinase/NF-kappaB cascade | 1.13E-10 |
| Gene_Set_1_4102 | lymphocyte differentiation | 1.74E-10 |
| Gene_Set_1_4102 | response to abiotic stimulus | 1.76E-10 |
| Gene_Set_1_4102 | organelle lumen | 3.73E-10 |
| Gene_Set_1_4102 | regulation of catalytic activity | 5.04E-10 |
| Gene_Set_1_4102 | cell division | 6.01E-10 |
| Gene_Set_1_4102 | Rho protein signal transduction | 6.08E-10 |
| Gene_Set_1_4102 | endomembrane system | 6.66E-10 |
| Gene_Set_1_4102 | lipid binding | 6.77E-10 |
| Gene_Set_1_4102 | hydrolase activity, acting on ester bonds | 7.10E-10 |
| Gene_Set_1_4102 | growth factor binding | 8.29E-10 |
| Gene_Set_1_4102 | endoplasmic reticulum | 9.08E-10 |

| **Gene Set 2** | **Enriched GO Term** | ***P*-value** |
|---|---|---|
| Gene_Set_2_4102 | lipid metabolism | 5.24E-34 |
| Gene_Set_2_4102 | cellular lipid metabolism | 1.07E-26 |
| Gene_Set_2_4102 | organic acid metabolism | 7.63E-19 |
| Gene_Set_2_4102 | oxidoreductase activity | 4.23E-13 |
| Gene_Set_2_4102 | fatty acid metabolism | 5.51E-13 |
| Gene_Set_2_4102 | lipid biosynthesis | 1.23E-12 |
| Gene_Set_2_4102 | generation of precursor metabolites and energy | 2.10E-12 |
| Gene_Set_2_4102 | steroid metabolism | 6.95E-12 |
| Gene_Set_2_4102 | coenzyme binding | 1.30E-11 |
| Gene_Set_2_4102 | catabolism | 7.64E-11 |
