# Supplementary material for: Global Expression Profiling in Atopic Eczema Reveals Reciprocal Expression of Inflammatory and Lipid Genes
Source: PLoS One. 2008 Dec 24;3(12):e4017. doi: 10.1371/journal.pone.0004017 (PMC2603322; doi:10.1371/journal.pone.0004017)
Supplement: Table S3 — Genes located in enriched AE-linked chromosomal regions. Shown are differentially expressed AE genes located within disease susceptibility chromosomal regions (cytobands) that are enriched in 2194 genes induced (Gene Set 1) or 1908 genes repressed (Gene Set 2) in AE (P<0.05; FDR<0.05). (0.06 MB PDF) [file pone.0004017.s003.pdf]

Supplementary Table S3. Genes located in enriched AE-linked chromosomal regions. Shown are differentially expressed AE genes located within disease suceptibility chromosomal regions (cytobands) that are enriched in 2194 genes induced (Gene Set 1) or 1908 genes repressed (Gene Set 2) in AE (*P*<0.05; FDR<0.05).

Gene Set #	Cytoband	Entrez Gene	Symbol
1	11q12.1	221	ALDH3B1
1	11q12.1	572	BAD
1	11q12.1	823	CAPN1
1	11q12.1	921	CD5
1	11q12.1	923	CD6
1	11q12.1	1521	CTSW
1	11q12.1	2197	FAU
1	11q12.1	2206	MS4A2
1	11q12.1	2286	FKBP2
1	11q12.1	2950	GSTP1
1	11q12.1	3338	DNAJC4
1	11q12.1	3992	FADS1
1	11q12.1	5007	OSBP
1	11q12.1	5499	PPP1CA
1	11q12.1	5790	PTPRCAP
1	11q12.1	5837	PYGM
1	11q12.1	5920	RARRES3
1	11q12.1	6094	ROM1
1	11q12.1	6520	SLC3A2
1	11q12.1	9049	AIP
1	11q12.1	9092	SART1
1	11q12.1	9158	FIBP
1	11q12.1	10312	TCIRG1
1	11q12.1	10589	DRAP1
1	11q12.1	10897	YIF1A
1	11q12.1	10938	EHD1
1	11q12.1	29901	SAC3D1
1	11q12.1	30008	EFEMP2
1	11q12.1	51338	MS4A4A
1	11q12.1	58475	MS4A7
1	11q12.1	64231	MS4A6A
1	11q12.1	65003	MRPL11
1	11q12.1	79073	TMEM109
1	11q12.1	80198	MUS81
1	11q12.1	219928	MRGPRF
1	11q12.1	220002	CYBASC3
1	11q12.1	245802	MS4A6E
1	11q12.1	283237	TTC9C
1	19p13.3	997	CDC34
1	19p13.3	1613	DAPK3
1	19p13.3	1675	CFD
1	19p13.3	2208	FCER2

1	19p13.3	2769	GNA15
1	19p13.3	4542	MYO1F
1	19p13.3	4616	GADD45B
1	19p13.3	5434	POLR2E
1	19p13.3	5442	POLRMT
1	19p13.3	5605	MAP2K2
1	19p13.3	5657	PRTN3
1	19p13.3	5802	PTPRS
1	19p13.3	5990	RFX2
1	19p13.3	7409	VAV1
1	19p13.3	8570	KHSRP
1	19p13.3	8698	S1PR4
1	19p13.3	10148	EBI3
1	19p13.3	10469	TIMM44
1	19p13.3	23370	ARHGEF18
1	19p13.3	23526	HMHA1
1	19p13.3	30835	CD209
1	19p13.3	51341	ZBTB7A
1	19p13.3	55009	C19orf24
1	19p13.3	55643	BTBD2
1	19p13.3	56927	GPR108
1	19p13.3	56931	DUS3L
1	19p13.3	84266	ALKBH7
1	19p13.3	84330	ZNF414
1	19p13.3	84823	LMNB2
1	19p13.3	91304	C19orf6
1	19p13.3	125988	P117
1	19p13.3	126282	TNFAIP8L1
1	19p13.3	126308	MOBKL2A
1	19p13.3	126328	NDUFA11
1	3p21.31	327	APEH
1	3p21.31	1154	CISH
1	3p21.31	2876	GPX1
1	3p21.31	3373	HYAL1
1	3p21.31	3615	IMPDH2
1	3p21.31	3913	LAMB2
1	3p21.31	4134	MAP4
1	3p21.31	5580	PRKCD
1	3p21.31	7375	USP4
1	3p21.31	7866	IFRD2
1	3p21.31	7867	MAPKAPK3
1	3p21.31	10641	TUSC4
1	3p21.31	11070	TMEM115
1	3p21.31	11186	RASSF1
1	3p21.31	11334	TUSC2
1	3p21.31	11344	TWF2
1	3p21.31	29925	GMPPB
1	3p21.31	51246	SHISA5
1	3p21.31	51368	TEX264
1	3p21.31	79714	CCDC51

1	16p11.2	3683	ITGAL
1	16p11.2	3684	ITGAM
1	16p11.2	3687	ITGAX
1	16p11.2	8479	HIRIP3
1	16p11.2	10295	BCKDK
1	16p11.2	10421	CD2BP2
1	16p11.2	10423	CDIPT
1	16p11.2	26000	TBC1D10B
1	16p11.2	27040	LAT
1	16p11.2	79077	XTP3TPA
1	16p11.2	83985	SPNS1
1	16p11.2	112476	PRRT2
1	16p11.2	146542	ZNF688
1	16p11.2	260434	PYDC1
1	19q13.2	945	CD33
1	19q13.2	951	CD37
1	19q13.2	2217	FCGRT
1	19q13.2	2323	FLT3LG
1	19q13.2	2359	FPR3
1	19q13.2	3903	LAIR1
1	19q13.2	5653	KLK6
1	19q13.2	5655	KLK10
1	19q13.2	8541	PPFIA3
1	19q13.2	11012	KLK11
1	19q13.2	11025	LILRB3
1	19q13.2	11202	KLK8
1	19q13.2	22809	ATF5
1	19q13.2	23636	NUP62
1	19q13.2	26121	PRPF31
1	19q13.2	79042	TSEN34
1	19q13.2	91663	MYADM
1	19q13.2	147657	ZNF480
1	19p13.2	439	ASNA1
1	19p13.2	976	CD97
1	19p13.2	1032	CDKN2D
1	19p13.2	1785	DNM2
1	19p13.2	1936	EEF1D
1	19p13.2	3337	DNAJB1
1	19p13.2	3385	ICAM3
1	19p13.2	5585	PKN1
1	19p13.2	8666	EIF3G
1	19p13.2	9592	IER2
1	19p13.2	10498	CARM1
1	19p13.2	11140	CDC37
1	19p13.2	28974	C19orf53
1	19p13.2	51073	MRPL4
1	19p13.2	56342	PPAN
1	19p13.2	57572	DOCK6

1	19p13.2	84261	FBXW9
1	19p13.2	90378	SAMD1
1	3p25.3	2199	FBLN2
1	3p25.3	5894	RAF1
1	3p25.3	6396	SEC13
1	3p25.3	7428	VHL
1	3p25.3	7476	WNT7A
1	3p25.3	9797	TATDN2
1	3p25.3	10533	ATG7
1	3p25.3	23225	NUP210
1	3p25.3	79885	HDAC11
1	12q13.12	3225	HOXC9
1	12q13.12	3489	IGFBP6
1	12q13.12	3695	ITGB7
1	12q13.12	3852	KRT5
1	12q13.12	4778	NFE2
1	12q13.12	6895	TARBP2
1	12q13.12	9700	ESPL1
1	12q13.12	54458	PRR13
1	12q13.12	60314	C12orf10
1	12q13.12	84926	SPRYD3
1	19p13.12	684	BST2
1	19p13.12	1311	COMP
1	19p13.12	3718	JAK3
1	19p13.12	8178	ELL
1	19p13.12	9244	CRLF1
1	19p13.12	10437	IFI30
1	19p13.12	23149	FCHO1
1	19p13.12	54555	DDX49
1	19p13.12	79016	DDA1
1	19p13.12	79036	C19orf50
1	19p13.12	79709	GLT25D1
1	19p13.12	170436	LOC696405
1	1q21.3	1513	CTSK
1	1q21.3	1520	CTSS
1	1q21.3	3713	IVL
1	1q21.3	4170	MCL1
1	1q21.3	5692	PSMB4
1	1q21.3	6281	S100A10
1	1q21.3	6700	SPRR2A
1	1q21.3	11000	SLC27A3
1	1q21.3	27173	SLC39A1
1	1q21.3	54544	CRCT1
1	1q21.3	57530	CGN
1	1q21.3	163778	SPRR4

1	19q13.13	581	BAX
1	19q13.13	2512	FTL
1	19q13.13	2523	FUT1
1	19q13.13	2769	GNA15
1	19q13.13	6141	RPL18
1	19q13.13	51171	HSD17B14
1	19q13.13	57664	PLEKHA4
1	5q32	475	ATOX1
1	5q32	1452	CSNK1A1
1	5q32	3340	NDST1
1	5q32	5159	PDGFRB
1	5q32	6678	SPARC
1	5q32	206358	SLC36A1
1	17q11.2	230	ALDOC
1	17q11.2	2124	EVI2B
1	17q11.2	2319	FLOT2
1	17q11.2	3965	LGALS9
1	17q11.2	5717	PSMD11
1	17q11.2	7756	ZNF207
1	17q11.2	9703	KIAA0100
1	17q11.2	28964	GIT1
1	17q11.2	83871	RAB34
1	17q11.2	84282	RNF135
1	14q11.2	5721	PSME2
1	14q11.2	6035	RNASE1
1	14q11.2	6039	RNASE6
1	14q11.2	7051	TGM1
1	14q11.2	9056	SLC7A7
1	14q11.2	9878	TOX4
1	14q11.2	10379	IRF9
1	14q11.2	10548	TM9SF1
1	14q11.2	26277	TINF2
1	14q11.2	29082	CHMP4A
1	14q11.2	122664	TPPP2
1	12q13.13	1019	CDK4
1	12q13.13	2065	ERBB3
1	12q13.13	4035	LRP1
1	12q13.13	4327	MMP19
1	12q13.13	10106	CTDSP2
1	12q13.13	10956	OS9
1	12q13.13	23344	FAM62A
1	12q13.13	79888	LPCAT1
1	12q13.13	92979	MARCH9

1	12q13.13	116986	CENTG1
1	2q33.3	1493	CTLA4
1	2q33.3	8828	NRP2
1	2q33.3	10152	ABI2
1	2q33.3	29851	ICOS
1	16q12.2	1459	CSNK2A2
1	16q12.2	4313	MMP2
1	16q12.2	4494	MT1F
1	16q12.2	4499	MT1M
1	16q12.2	10300	KATNB1
1	16q12.2	29105	C16orf80
1	16q12.2	84166	NLRC5
2	12q13.11	362	AQP5
2	12q13.11	2819	GPD1
2	12q13.11	3855	KRT7
2	12q13.11	4891	SLC11A2
2	12q13.11	23017	FAIM2
2	12q13.11	25840	METTL7A
2	12q13.11	25875	LETMD1
2	15q21.3	55329	MNS1
2	15q21.3	55930	MYO5C
2	15q21.3	84952	CGNL1
2	15q21.3	145783	LOC145783
2	3q22.1	5096	PCCB
2	3q22.1	22808	MRAS
2	3q22.1	80321	CEP70
