# Supplementary material for: Global Expression Profiling in Atopic Eczema Reveals Reciprocal Expression of Inflammatory and Lipid Genes
Source: PLoS One. 2008 Dec 24;3(12):e4017. doi: 10.1371/journal.pone.0004017 (PMC2603322; doi:10.1371/journal.pone.0004017)
Supplement: Table S4 — Overview of skin biopsy samples that were used in the SAM approach to identify differentially expressed genes in AE and healthy skin. Shown are six groups of samples representing skin from atopic eczema patients (SAM group 1-3: AE-L, AE-M, AE-P) and healthy control individuals (SAM group 4-6: H-N, H-M, H-P). Corresponding microarray slide name are given for each sample using the same nomenclature as is used in the Stanford Microarray Database (SMD). (0.05 MB PDF) [file pone.0004017.s004.pdf]

Supplementary Table S4. Overview of skin biopsy samples that were used in the SAM approach to identify differentially expressed genes in AE and healthy skin. Shown are six groups of samples representing skin from atopic eczema patients (SAM group 1-3: AE-L, AE-M, AE-P) and healthy control individuals (SAM group 4-6: H-N, H-M, H-P). Corresponding microarray slide name are given for each sample using the same nomenclature as is used in the Stanford Microarray Database (SMD).

SAM GROUP	ARRAY NAME	INDIVIDE #	SKIN TYPE	BODY POSITION
1	SHDD222	AE-1L	Lesional	leg
1	SHDD225	AE-2L	Lesional	arm
1	SHDD227	AE-3L	Lesional	leg
1	SHDD208	AE-4L	Lesional	back
1	SHDD215	AE-6L	Lesional	back
1	SHDD219	AE-7L	Lesional	back
2	SHDD223	AE-1M	M sympodialis	back
2	SHDD226	AE-2M	M sympodialis	back
2	SHDD228	AE-3M	M sympodialis	back
2	SHDD213	AE-5M	M sympodialis	back
2	SHDD216	AE-6M	M sympodialis	back
2	SHDD220	AE-7M	M sympodialis	back
3	SHDD221	AE-1P	PBS	back
3	SHDD224	AE-2P	PBS	back
3	SHDD207	AE-4P	PBS	back
3	SHDD211	AE-5P	PBS	back
3	SHDD214	AE-6P	PBS	back
4	SHDD237	H-1N	normal	back
4	SHDD241	H-2N	normal	back
4	SHDD244	H-3N	normal	back
4	SHDD247	H-4N	normal	back
5	SHDD239	H-1M	M sympodialis	back
5	SHDD243	H-2M	M sympodialis	back
5	SHDD246	H-3M	M sympodialis	back
5	SHDD249	H-4M	M sympodialis	back
6	SHDD238	H-1P	PBS	back
6	SHDD242	H-2P	PBS	back
6	SHDD245	H-3P	PBS	back
6	SHDD248	H-4P	PBS	back
